# Development of Multitaxon Indices of Biotic Integrity for Aquatic Ecosystem Health Assessment in Dongjiang Lake

**DOI:** 10.3390/biology15100765

**Published:** 2026-05-11

**Authors:** Yu Wang, Meiyu Hou, Hanbing Li, Rui Wang, Xin Zhou, Liangjing Zhang, Qiang Zhou, Rui Meng

**Affiliations:** 1 State Key Laboratory of Environmental Criteria and Risk Assessment, Chinese Research Academy of Environmental Sciences, Beijing 100012, China; wangyucraes@163.com (Y.W.); wang.rui@craes.org.cn (R.W.); zhouxin_1991@126.com (X.Z.); 18286808172@163.com (L.Z.); zhouqiang20577@163.com (Q.Z.); 2 Dongjiang Lake Water Environment Monitoring Station of Chenzhou City, Chenzhou 423000, China; houmin823@163.com (M.H.); binglove201@163.com (H.L.)

**Keywords:** index of biotic integrity (IBI), macroinvertebrates, zooplankton, phytoplankton, aquatic ecosystem health assessment

## Abstract

Three locally calibrated Indices of Biotic Integrity (IBIs) based on macroinvertebrates (B-IBI), zooplankton (Z-IBI), and phytoplankton (P-IBI) were developed to characterize relative aquatic ecological condition at impaired sites in Dongjiang Lake, a deep reservoir-type lake in China, during 2021–2023. Using synchronous monitoring data, candidate metrics for the three biotic groups were screened and assembled by integrating taxonomic diversity, community composition, pollution-tolerance attributes, trophic indicators, and functional feeding groups. Metric values were standardized using a linear transformation, and site conditions were classified using a unified five-class grading scheme under the present local calibration framework. A total of 327 taxonomic units (species or morphospecies) were recorded across the three biotic groups, indicating relatively high biodiversity in the study area. Under the present locally calibrated framework, most impaired sites were classified within the moderate-to-good range, with clear interannual variation and spatial heterogeneity. Upstream and downstream sections showed greater fluctuations in IBI classes than the lake area. The macroinvertebrate-based IBI was more sensitive to long-term and cumulative habitat disturbance, whereas the zooplankton- and phytoplankton-based IBIs responded more rapidly to short-term variation in nutrients and water quality. Together, these results indicate that multitaxon IBIs can provide complementary information on relative ecological condition within Dongjiang Lake and may support ecological zoning, pollutant management, and restoration prioritization in similar deep reservoir-type lake systems.

## Simple Summary

Dongjiang Lake (Hunan Province, China) is a representative large, deep reservoir-type lake. In this study, we developed and applied three Indices of Biotic Integrity (IBI) based on Macroinvertebrates (B-IBI), Zooplankton (Z-IBI), and Phytoplankton (P-IBI) to evaluate aquatic ecosystem health during 2021–2023. Using synchronous monitoring data and based on complete taxonomic inventories and abundance/biomass information, candidate metrics were screened and assembled for each biotic group by integrating taxonomic diversity, community composition, pollution tolerance and trophic indicator attributes, and functional feeding groups. Linear standardization and a unified five-class grading scheme were then used to assess site-level health status. A total of 327 taxonomic units (species or morphospecies) were recorded across the three biotic groups, indicating relatively high biodiversity. The macroinvertebrate-based IBI was most sensitive to long-term and cumulative habitat disturbance, facilitating the identification of persistently stressed river sections, whereas the zooplankton- and phytoplankton-based IBIs responded rapidly to short-term changes in nutrients and water quality, showing synchronous deterioration in 2023 and partial seasonal recovery at some sites. The integration of multitaxon IBIs provides a locally calibrated framework for relative ecological condition assessment in Dongjiang Lake, supporting ecological zoning, pollutant regulation, and prioritization of restoration efforts within the study system.

## 1. Introduction

### 1.1. Index of Biotic Integrity

The Index of Biotic Integrity (IBI) was originally proposed by Karr in 1981 as a multimetric framework for assessing aquatic ecological condition through the structure and function of biological communities [1]. The original fish-based IBI linked community composition, trophic structure, abundance, and organism condition to the broader concept of biological integrity advanced in freshwater management [2]. Its central premise is that anthropogenic disturbance alters aquatic assemblages in predictable ways across multiple ecological dimensions, so a set of disturbance-responsive metrics can be integrated into a single index to provide a more comprehensive assessment than any single descriptor alone [2,3,4].

Subsequent studies extended the IBI concept beyond fish to other biological groups and habitat types, including macroinvertebrates, algae, amphibians, birds, wetlands, and lakes [5,6,7,8,9]. At the same time, methodological development moved from expert-driven metric choice toward more standardized procedures involving reference-condition calibration, redundancy screening, and percentile-based scoring [10,11]. Despite this progress, current IBI applications still face several recurring challenges, including region-specific metric behavior, limited transferability across ecosystem types, and the difficulty of integrating complementary signals from different biological groups within one assessment framework [10,11]. These limitations are particularly relevant when IBI is applied to complex freshwater systems such as deep reservoir-type lakes.

### 1.2. Applications of IBI Across Biological Groups

Although the IBI was originally developed for fish, it has since been established and applied for multiple aquatic biological groups—including macroinvertebrates, zooplankton, and phytoplankton—to evaluate ecosystem health in diverse settings. While the construction of IBIs across groups generally follows a similar multimetric framework, differences in organismal ecology lead to distinct emphases in metric selection and assessment focus. Below, we review major advances in large-scale applications, index construction, and comparative strengths and limitations for the three biological groups used in this study.

#### 1.2.1. Macroinvertebrate IBI (B-IBI)

Macroinvertebrates are key indicator taxa for assessing aquatic ecological quality, and the development of B-IBI closely followed fish-based IBI. In the early 1990s, Karr and colleagues incorporated macroinvertebrate metrics into the fish-IBI framework to develop B-IBI. The B-IBI proposed by Kerans and Karr (1994) for the Tennessee Valley included 13 metrics—such as richness of aquatic insects (at the order level), the proportion of sensitive families, functional feeding groups, and the proportion of pollution-tolerant taxa—to capture macroinvertebrate responses to pollution and habitat alteration [12]. Subsequently, localized B-IBIs were developed in many countries for river and stream health assessment [13,14,15]. For example, in three Portuguese estuaries (Mondego, Tejo, and Mira), researchers sampled benthic macroinvertebrates and sediment physicochemical variables at 41 stations in summer to evaluate ecological status within the framework of the EU Water Framework Directive. Building on the Chesapeake Bay B-IBI approach, a set of metrics (e.g., diversity, richness, biomass, and proportions of sensitive/tolerant groups) was used; B-IBI scores were calculated based on abundance and biomass thresholds, and sites were classified into high/good, moderate, and poor/bad categories. Most stations were classified as below “good” status, particularly in the Tejo and Mira estuaries, which showed extensive poor/bad conditions. These classifications were highly consistent with “moderate–poor” water quality indicated by nutrient and heavy metal measurements, suggesting that B-IBI can effectively identify reaches under strong human pressure [15]. Compared with fish, macroinvertebrates generally have shorter life cycles and smaller dispersal ranges, making them sensitive indicators of local water quality and habitat conditions. Therefore, B-IBI is often used to track relatively fine spatiotemporal changes at reach or microhabitat scales [16]. Common B-IBI metric categories include richness and diversity indices, dominance and compositional attributes, proportions of tolerant/sensitive taxa, and functional feeding group structure [17]. B-IBI is advantageous for reflecting cumulative impacts of disturbances over short-to-intermediate timescales, but it may respond less rapidly than plankton to instantaneous disturbances or strong seasonal shifts. In addition, macroinvertebrate sampling and taxonomic identification require specialized expertise, and standardization and labor costs should be considered for large-scale applications.

#### 1.2.2. Zooplankton IBI (Z-IBI)

Zooplankton—primarily rotifers, cladocerans, and copepods in freshwater ecosystems—have increasingly been used in IBI development in recent years due to their sensitive responses to water quality and trophic conditions. Early work was limited until Lougheed et al. [18] developed the Wetland Zooplankton Index (WZI), which was among the first to systematically use zooplankton for wetland health assessment. Based on samples from 70 wetlands in the Laurentian Great Lakes region spanning gradients from minimally disturbed, vegetation-rich wetlands to severely degraded sites, the study found that high-quality wetlands were dominated by epiphytic crustaceans, whereas degraded wetlands were characterized by pollution-tolerant zooplankton. By integrating the indicative value of different zooplankton groups, the WZI distinguished disturbance levels more effectively than conventional diversity indices [18]. The concept of zooplankton-based integrity assessment has since expanded to lakes, reservoirs, estuaries, and bays. For example, a zooplankton IBI for Chesapeake Bay salt marshes used metrics such as the relative proportions of large crustacean zooplankton and microzooplankton to evaluate eutrophication effects [19]. In China, zooplankton IBI research has also increased in recent years. Mutethya et al. [20] developed a Z-IBI for urban rivers in Haikou (Hainan Island) using five metrics (e.g., zooplankton abundance, protozoan abundance, copepod abundance, cladoceran biomass proportion, and Shannon diversity) and successfully applied it to assess reach-scale ecological status. The Z-IBI scores were significantly correlated with nutrients (total nitrogen and total phosphorus) and COD, indicating sensitivity to human impacts on urban river ecosystems [20]. Overall, Z-IBI has the advantages of rapid responsiveness to water quality changes and relatively straightforward sampling and analysis. However, zooplankton communities often show strong seasonal and hydrological variability; thus, temporal and spatial heterogeneity should be explicitly considered to ensure stable and comparable assessments.

#### 1.2.3. Phytoplankton IBI (P-IBI)

Phytoplankton community structure directly reflects nutrient regimes and light conditions and is therefore a central component of aquatic ecosystem health assessment. The development of phytoplankton-based IBI began in the early 2000s, with early work conducted in North America and Europe. For instance, Wu et al. developed a P-IBI for German lowland rivers and demonstrated that screened phytoplankton metrics could effectively discriminate ecological conditions in reaches influenced by dams [21]. P-IBI metrics are typically selected from species composition, density/biomass, and dominance structure. In large rivers, candidate metrics may include total phytoplankton richness, cyanobacterial richness, diatom density and biomass, and dominance ratios of key taxa [22]. These metrics can represent algal diversity (richness), community structure (relative contributions of major algal groups), and overall primary production (total density/biomass), and they are often strongly indicative of nutrient enrichment and bloom risk. Seasonal effects are frequently important for P-IBI construction because phytoplankton communities can shift substantially between dry and wet seasons. Accordingly, some studies screen metrics and establish assessment criteria separately by season; for example, Zhu et al. developed two P-IBI systems for Lake Gehu (Taihu region) for wet and dry seasons to enhance year-round applicability [23]. Phytoplankton-based indices are now widely used in eutrophication management. Under the EU Water Framework Directive, Padisák et al. proposed an assemblage index based on phytoplankton functional groups to assess lake ecological status [24]. In China, phytoplankton integrity assessments have also been applied in lowland rivers and drinking-water reservoirs; for example, phytoplankton assessments in the middle–lower Yangtze River suggested generally good ecological health with distinct spatial variation [25]. P-IBI is advantageous for its sensitivity to nutrient fluctuations and its capacity to provide early warning for algal blooms, but because phytoplankton can respond rapidly to short-term disturbances, single-snapshot sampling may be affected by stochasticity. Robustness can be improved through repeated monitoring, averaging across surveys, or incorporating functional-group metrics.

### 1.3. Current Status of IBI Applications in Chinese Freshwater Ecosystems

Over the past two decades, the IBI concept has gained increasing attention and application in freshwater ecological protection and assessment in China. IBI-related studies now span multiple waterbody types, including rivers [25], lakes [26], reservoirs [27], and wetlands [28], and cover broad geographic regions from Northeast China and the North China Plain to the Yangtze and Pearl River basins. Among these, the Yangtze River Basin has attracted particularly intensive research given its ecological significance; this includes the development and application of fish-based IBI, macroinvertebrate IBI, and plankton-based IBI in the middle–lower mainstem and key tributaries. For example, new fish-based IBIs have been developed to evaluate dam impacts in the upper Yangtze River [29], and diatom-based IBIs have also been established [30]. In general, research has been more concentrated in the Yangtze Basin and eastern regions, whereas studies in western plateau and remote areas are still emerging; nevertheless, nationwide IBI applications continue to expand and mature.

In terms of research scope and methodology, early Chinese IBI studies largely adapted international metric systems and conducted validation-oriented case studies in local waters. More recently, research has increasingly emphasized alignment with China’s ecological characteristics. For example, some studies targeting southern rivers and mountain streams have incorporated functional feeding groups and benthic algae into macroinvertebrate IBIs to better reflect the ecological status of rural rivers and small catchments. To overcome limitations of single-taxon indices, other studies have attempted to develop integrated multitaxon IBIs (m-IBI) by combining metrics from fish, macroinvertebrates, zooplankton, and phytoplankton, thereby improving robustness and comprehensiveness. In parallel, some researchers have used statistical models to quantify relationships between IBI scores and environmental drivers (e.g., nutrients and land-use patterns), providing more operational evidence for watershed management [31]. Overall, Chinese IBI research is transitioning from single-site applications toward more systematic, localized, and integrative frameworks. However, multitaxon IBI studies focusing on large, deep reservoir-type lakes remain limited, particularly for systems with pronounced upstream–lake–downstream gradients and synchronous monitoring of macroinvertebrates, zooplankton, and phytoplankton. Therefore, this study aims not only to apply IBI in such a system, but also to fill this methodological gap by developing a locally calibrated multitaxon framework for integrated relative ecological condition assessment in Dongjiang Lake.

### 1.4. Overall Study Design and Objectives

This study focuses on the Dongjiang Lake Basin (Hunan Province, China) to evaluate the applicability of the Index of Biotic Integrity (IBI) for freshwater ecosystem assessment in this region and to characterize biodiversity responses across different biological groups. We selected three major aquatic biotic groups—macroinvertebrates, zooplankton, and phytoplankton—as assessment targets. Based on species composition, community structure, and functional attributes, we screened a suite of candidate metrics that are sensitive to environmental disturbance and ecologically informative, and then developed group-specific IBI assessment systems. Although established single-group indices such as BMWP, ASPT, and other regional tools provide valuable references, most were originally developed primarily for riverine systems and individual taxonomic groups rather than for integrated assessment in deep reservoir-type lakes. The scientific novelty of the present study lies in integrating three major aquatic biotic groups within one locally calibrated assessment framework for a deep reservoir-type lake with marked upstream–lake–downstream gradients. Through field sampling and monitoring at representative transects in these three spatial units, we calculated IBI scores for each biotic group, classified relative ecological condition, and compared spatiotemporal patterns among biological groups. Accordingly, the present study developed a locally calibrated multitaxon framework as a complementary tool, rather than directly applying or replacing any single pre-existing index. By integrating multitaxon IBI applications, this study provides quantitative support for ecological zoning management and conservation planning in the Dongjiang Lake Basin and offers a methodological reference for multitaxon biological integrity assessment in similar reservoir-type lake systems.

## 2. Methods

### 2.1. Study Area

**Figure 1:**
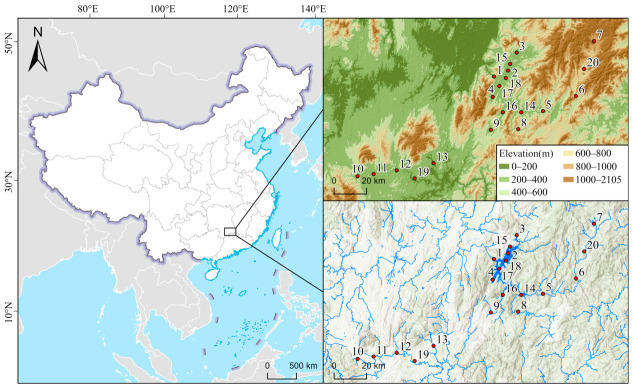
Sampling sites in Dongjiang Lake area.

This study was conducted in the Dongjiang Lake Basin, located in Zixing City, Hunan Province, China. Dongjiang Lake is situated in the upper reaches of the Leishui River, a tributary of the Xiangjiang River, and is a large, deep reservoir-type lake primarily used for hydropower generation and water supply, with additional flood-control functions. The reservoir has a surface area of approximately 160 km^2^, a length of about 103 km, and a maximum width of 6.2 km. It is a deep-water body, with an average depth of 61 m and a maximum depth of 141 m. The total storage capacity is 9.15 × 10^9^ m^3^, with a normal storage volume of 8.12 × 10^9^ m^3^. Its catchment area is about 4719 km^2^, and the reservoir is characterized by a regulated hydrological regime with an average annual inflow of 4.48 × 10^9^ m^3^. Along the hydrological continuum from the upstream inflow reaches to nearshore embayments, the open-lake area, and the downstream discharge reaches, Dongjiang Lake exhibits pronounced gradients in hydrology and habitat conditions. Based on the hydrological setting and the intensity of human activities, a total of 20 fixed sampling sites (Sample1–20) were established across the basin. These sites broadly cover representative upstream, lake-area, and downstream waterbody types and collectively capture the major environmental characteristics of Dongjiang Lake. Field sampling was conducted annually from 2021 to 2023, with seasonal surveys mainly carried out in May and September each year. At each site, samples of macroinvertebrates, zooplankton, and phytoplankton were collected synchronously, and corresponding physicochemical water-quality variables were measured. These datasets provided the basis for developing multitaxon IBIs and assessing aquatic ecosystem health in Dongjiang Lake. During the first sampling campaign, sites were assigned to operational reference-calibration and impaired-calibration groups based on expert evaluation of habitat condition, surrounding anthropogenic disturbance, and hydrological setting. The expert assessment emphasized whether sites retained relatively complete habitat features and lower visible human disturbance within the same hydromorphological context, whereas impaired-calibration sites were characterized by stronger surrounding disturbance and/or less favorable habitat condition. These groups were then used for metric screening and score standardization within the present dataset and should therefore be understood as operational calibration groups for local assessment, rather than as independently validated ecological endpoints. The spatial distribution of sampling sites is shown in Figure 1, and site coordinates are provided in Supplementary Table S1.

### 2.2. Sampling Methods

#### 2.2.1. Macroinvertebrates Sampling and Identification

Macroinvertebrates sampling followed the methods recommended by the China National Environmental Monitoring Centre [32]. Quantitative sampling was conducted as follows. In rivers and shallow-water areas, a standard Surber net or a benthic sampler (sampling area approximately 0.1 m^2^; mesh size 500 µm) was used. At each site, multiple replicate samples were collected along representative reaches or shoreline sections by disturbing the substrate and rinsing the material through the mesh to retain organisms. In deep-water zones or areas with soft sediments, a grab sampler was used; the surface sediment was washed through a 500 µm sieve, and retained macroinvertebrates were collected. After removing coarse debris in the field, all samples were preserved in 75% ethanol and transported to the laboratory. In the laboratory, individuals were sorted under a stereomicroscope, identified, and counted. Organisms were generally identified to species or morphospecies level, while some difficult taxa were identified to genus or family level, following published taxonomic keys and monographs for freshwater benthic macroinvertebrates [33,34,35]. Abundance was converted to areal density (ind/m^2^), and biomass was estimated using body length–weight relationships to provide abundance- and biomass-based data for subsequent IBI metric calculations.

#### 2.2.2. Zooplankton Sampling and Identification

Zooplankton sampling differed among major groups. For cladocerans and copepods, sampling followed the China National Environmental Monitoring Centre protocol [36]. A depth-integrated water sampler was used to collect a composite 20 L water sample by mixing water from the surface, mid-water, and bottom layers. The sample was slowly filtered through a 112 µm plankton net, the concentrate was adjusted to a final volume of 10 mL, and Lugol’s solution was immediately added for fixation and preservation. In the laboratory, the sample was gently homogenized and subsampled using a 5 mL counting chamber. Depending on organism concentration, either diagonal transect counts or full-chamber counts were performed to ensure counting precision. Rotifers and protozoa were handled differently: for both groups, 1 L water samples were collected, and identification/counting were conducted using a 1 mL counting chamber for rotifers and a 0.1 mL counting chamber for protozoa. Zooplankton taxa (e.g., rotifers, cladocerans, copepods) were identified to species or morphospecies level based on morphological characteristics, using published atlases and identification manuals for freshwater zooplankton [37,38,39]. Zooplankton density was calculated as ind/m^3^ based on counted individuals and the filtered water volume. Biomass was estimated using group-specific average individual mass or length–weight relationships and empirical conversion coefficients, yielding biomass data by species and by major group for subsequent development of richness-, structure-, and trophic status-related metrics.

#### 2.2.3. Phytoplankton Sampling and Identification

Phytoplankton sampling and identification followed the approach used for the protozoan component of the zooplankton procedure [40]. A depth-integrated water sampler was used to collect a composite 20 L water sample by mixing water from the surface, mid-water, and bottom layers. The sample was slowly filtered through a 112 µm plankton net, the concentrate was adjusted to 10 mL, and Lugol’s solution was immediately added for fixation and preservation. Phytoplankton counting was conducted using a 0.1 mL counting chamber. Taxonomic identification of phytoplankton followed published atlases and identification manuals for freshwater algae [41,42,43].

### 2.3. IBI Framework and Metric Definition

In this study, three independent IBI frameworks were developed for macroinvertebrates, zooplankton, and phytoplankton to characterize the integrated responses of different biotic groups to anthropogenic disturbance and trophic conditions. The overall procedure for IBI development followed Zhu et al. (2023) and You et al. (2021) related approaches [28,44].

For the macroinvertebrate IBI, candidate metrics were established based on the complete species (or morphospecies) inventory, supplemented with locally compiled information on sensitivity scores, tolerance values, and functional feeding group (FFG) assignments. Candidate metrics were organized into five ecological attributes: (1) Taxonomic richness, including total taxonomic units, richness of aquatic insects, and richness of EPT taxa; (2) Community composition, including abundance and relative abundance of representative groups (e.g., Oligochaeta, Chironomidae, Mollusca); (3) Diversity characteristics, including the Shannon–Wiener, Simpson, Margalef, and Pielou indices; (4) Pollution tolerance patterns, in which taxa were classified as intolerant, tolerant, or facultative according to tolerance values, and metrics were constructed using group richness/proportions; composite indices such as BMWP and HBI were also included; (5) Functional feeding structure, quantified by the richness and proportion of key FFGs (e.g., shredders, scrapers/grazers, filter feeders, collectors, and predators) to represent benthic food-web functional organization.

For the zooplankton IBI, candidate metrics emphasized species composition, quantitative structure, and trophic-state signals. These included total taxonomic units and richness of major groups (e.g., rotifers, cladocerans, and copepods), community density and biomass (and their allocation among groups), mean individual body mass, abundance and contribution of dominant taxa, diversity indices, and the abundance/proportion of feeding types (e.g., filter feeders and predators). In addition, trophic-indicator attributes (oligotrophic/eutrophic affinity) were compiled from the literature to construct trophic-status metrics such as the E/O index and Fertility of zooplankton.

For the phytoplankton IBI, candidate metrics focused on major algal-group composition and typical phytoplankton-based water-quality indices. Metrics included total taxonomic units; richness and proportions of Bacillariophyta (diatoms), Cyanobacteria, and Chlorophyta; total cell density; cell density and biomass by major algal groups and their relative contributions; diversity indices; and diatom-based indices such as the Diatom Quotient (DQ) based on centric vs. pennate diatom structure, the Palmer algal genus pollution index based on pollution-indicator genera, and the Generic Diatom Index (GDI) based on specific diatom genus assemblages.

In subsequent steps, these candidate metrics were screened according to their discriminatory power between the operational reference and impaired groups and their redundancy, resulting in the final metric subsets used for IBI assessment. Metric retention sought to preserve complementary ecological information across richness, composition, tolerance, and functional or trophic attributes so that the final multimetric framework would not be dominated by any single ecological dimension. When multiple candidate metrics showed comparable screening performance, preference was given to metrics with clearer ecological meaning and more consistent response directions across years and seasons, in order to improve the temporal robustness of the final metric sets. Detailed metric definitions and parameter settings are provided in Table S2a–c.

### 2.4. Acquisition of Biological Attributes for Candidate Metrics

Baseline taxonomic information was obtained from GBIF and NCBI. Their API services were used to construct a detailed taxonomic hierarchy for subsequent data integration and indicator calculation [45,46]. During the development of the three IBI frameworks (for macroinvertebrates, zooplankton, and phytoplankton), additional biological attribute information was required beyond the species checklist and site-specific abundance/biomass data. Functional feeding group (FFG) information for zooplankton and macroinvertebrates was compiled from the European freshwater ecological trait database https://www.freshwaterecology.info (accessed on 7 December 2025 ) [47]. For macroinvertebrates, sensitivity and tolerance values were obtained from the Ministry of Ecology and Environment document Technical Guidelines for Water Eco-Environmental Quality Monitoring and Evaluating of River and Stream (Draft). For zooplankton, oligotrophic/eutrophic indicator attributes were derived from Wang (2007), which documented indicator species for contrasting trophic conditions [48]. In the phytoplankton IBI calculations, one of the metrics was the Palmer algal genus pollution index. Palmer genus-level coefficients were extracted from Palmer (1969), which synthesized records of algae tolerant to high organic pollution from 165 contributors and 269 publications to develop a quantitative assessment tool. Palmer selected the top 20 genera and assigned each a concise pollution index factor ranging from 1 to 5 [49].

### 2.5. IBI Calculation Procedure

The IBI calculation followed four main steps: metric calculation, metric screening, standardization, and graded assessment. First, based on the predefined candidate metric sets for macroinvertebrates, zooplankton, and phytoplankton, raw data (e.g., abundance and biomass for each site across years and seasons) were used to compute metric values for each site, forming a multimetric baseline data matrix.

**Figure 2:**
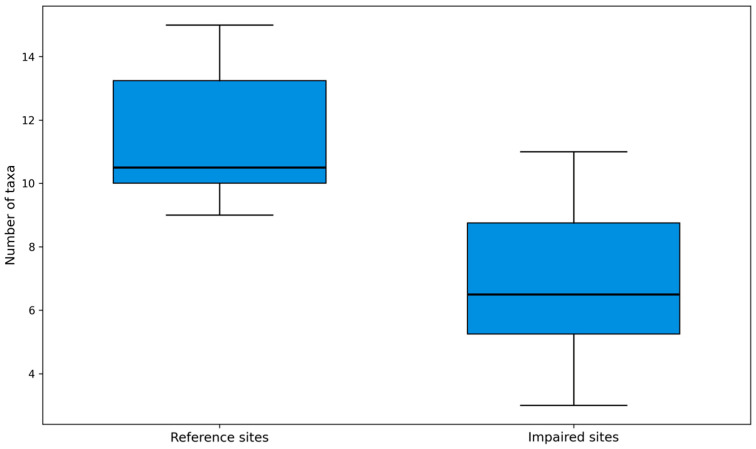
Example boxplot for distinguishing reference and impaired sites (macroinvertebrates, May 2021; metric M1_Number of taxa).

Second, metrics with limited information content were removed, including those with a high proportion of zeros in the operational reference-calibration sites or an excessively high zero frequency in the impaired-calibration sites. For each metric, the 5th, 25th, 50th, 75th, and 95th percentiles were calculated separately for the two calibration groups defined through expert-based habitat and disturbance assessment. Only metrics with adequate discriminatory power were retained—specifically, those for which the median (50th percentile) of one group did not fall within the interquartile range (25th–75th percentile) of the other group, indicating limited overlap and clear separation under the local disturbance gradient. This screening step was intended to identify disturbance-responsive metrics within Dongjiang Lake rather than to constitute external validation of the final IBI framework. To reduce the possibility that final metric selection was driven by a single sampling period, the discriminatory performance of candidate metrics was compared across years and seasons during screening. When alternative metrics showed similar boxplot separation, priority was given to those with more stable response directions and broader ecological interpretability. This procedure served as a pragmatic sensitivity check within the present dataset. A boxplot example is shown in Figure 2.

**Figure 3:**
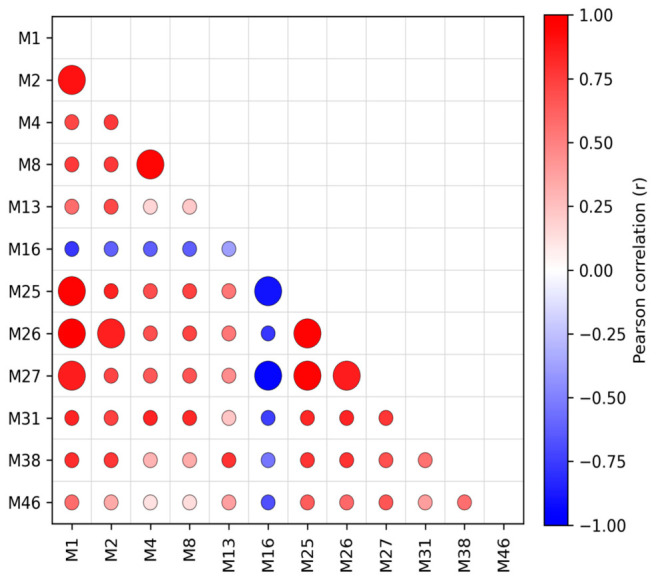
Example output of correlation analysis (macroinvertebrates, May 2021).

Third, redundancy among retained metrics was evaluated by pairwise correlation analysis. When several metrics described similar ecological attributes, one metric was preferentially retained on the basis of ecological interpretability, literature support, and complementarity with other attribute classes; highly correlated metrics (|r| > 0.85) were treated as candidates for removal. When the candidate pool was small, overly aggressive reduction was avoided so that the final index retained sufficient ecological information. Thus, the final metric sets were not selected solely on the basis of one correlation threshold, but also on whether alternative metrics conveyed overlapping information and whether the retained metrics together preserved the main ecological dimensions of the assemblages. An example correlation matrix is shown in Figure 3.

Fourth, each retained metric was classified according to its expected direction along a disturbance gradient, as either decreased (declining with increasing disturbance) or increased (rising with increasing disturbance). Using the 5th and 95th percentiles of the combined reference/impaired distributions as endpoints, each metric was linearly transformed to a 0–100 scale so that metrics with different units and directions became comparable; higher scores consistently indicated better ecological condition. For each biotic group, standardized metric scores were averaged (arithmetic mean) to obtain the site-level macroinvertebrate IBI, zooplankton IBI, and phytoplankton IBI values.

Finally, a unified five-class grading scheme was established using the 75th percentile of reference-site IBI values as the threshold for “excellent”. The range from 0 to this threshold was evenly divided into four classes-poor, fair, moderate, and good-resulting in the following five categories: poor, fair, moderate, good, and excellent. This framework enabled relative classification of ecological condition across sites and sampling periods under the present local calibration. To facilitate reader understanding of the screening and assessment workflow, the main text presents representative examples of a correlation matrix and boxplots for reference vs. impaired sites. Correlation plots and boxplots for each sampling event (May and September, 2021–2023) are provided in the Supplementary Materials (Files S7 and S8). NMDS ordination and metric–IBI correlations were used subsequently as supporting analyses to facilitate ecological interpretation and internal consistency checks, not as formal independent validation of index performance.

To formally evaluate the sensitivity of the IBI results to alternative metric selection, we conducted a within-dataset stability analysis for each biotic group, year, and sampling month. For each subset, the final metric set used in the main analysis was treated as the main metric set, and two alternative metric sets (Alt A and Alt B) were constructed by replacing selected metrics with eligible candidate metrics that had passed the preceding screening procedure. Replacement metrics were required to have the same response direction as the metric being replaced, were preferentially selected from the same ecological attribute category, and were chosen to have the most similar discrimination effect between reference and impaired sites. When no unused eligible substitute was available, the corresponding alternative set retained the main metric and this was recorded explicitly. For each main and alternative metric set, IBI scores were recalculated using the same scoring procedure as in the main analysis, and site classes were reassigned using the same reference-site-based classification rule. Stability was quantified by comparing each alternative set with the main set using Spearman correlation of IBI scores, mean absolute difference (MAD), exact class agreement, same-or-adjacent class agreement, and quadratic weighted kappa. This analysis was designed to assess within-dataset robustness to reasonable alternative metric substitutions rather than external validation.

### 2.6. Data Analysis

All data analyses and figure production were performed in Python 3.11, primarily using the open-source libraries NumPy v2.4.3, pandas v3.0.1, and Matplotlib v3.10.8 for numerical computation, data processing, and visualization [50,51,52]. The computation of IBI candidate metrics, percentile statistics for reference and impaired sites, 0–100 linear standardization, determination of IBI class thresholds, and visualization procedures (including boxplots and 3D pie charts) were all implemented within this environment. Two additional analyses were conducted to support interpretation of the locally calibrated IBI framework. Differences in community structure were examined using non-metric multidimensional scaling (NMDS) ordination based on species abundance matrices [53]. Bray–Curtis dissimilarities were first calculated among sampling sites, and two-dimensional NMDS coordinates were then obtained to visualize community-composition patterns across years and seasons; NMDS stress values were reported to evaluate ordination fit. In addition, permutational multivariate analysis of variance (PERMANOVA) based on Bray–Curtis dissimilarities was used to test whether community composition differed among years and seasons. Because the same sites were sampled repeatedly across survey periods, temporal permutations were restricted within site, and 999 permutations were used. Tests of multivariate homogeneity of dispersion (betadisper) were also performed as a supplementary check on whether significant PERMANOVA results could be influenced by unequal within-group dispersion. Associations between overall IBI patterns and representative metrics were examined using Spearman’s rank correlation analysis [54]. For each of the three biotic groups (macroinvertebrates, zooplankton, and phytoplankton), Spearman correlation coefficients and significance levels were calculated between seven representative metrics and the corresponding IBI classifications to assess whether the multimetric outcomes were directionally consistent with commonly used ecological descriptors. Results were summarized as correlation-matrix heatmaps to facilitate comparisons of association strength across metrics and biotic groups. These analyses were used only as internal consistency checks and ecological interpretation tools, not as independent validation of index performance, because both the IBIs and the representative metrics were derived from the same biological datasets and partially overlapping ecological information.

## 3. Results

### 3.1. Community Structure Characteristics

Across the six surveys conducted from 2021 to 2023, a total of 327 taxonomic units (species or morphospecies) were recorded in Dongjiang Lake across the three major biotic groups-macroinvertebrates, zooplankton, and phytoplankton. These taxa belonged to 7 phyla, 12 classes, and 53 families, indicating a relatively high level of biodiversity in the study area. Specifically, macroinvertebrates comprised 94 taxa from 4 phyla, 6 classes, and 41 families, and were dominated by Arthropoda (73 taxa, ~78% of macroinvertebrate richness). Insecta was the most species-rich class (70 taxa) and showed clear dominance in both richness and abundance (accounting for ~72% of total macroinvertebrate abundance). Zooplankton included 77 taxa, belonging mainly to Protozoa, Arthropoda, and Rotifera, with 29 rotifer taxa and 36 cladoceran + copepod taxa, which together formed the core of the zooplankton assemblage. Phytoplankton comprised 156 taxa across seven algal phyla; diatoms (55 taxa, 35%) and green algae (51 taxa, 33%) were co-dominant in richness, while cyanobacteria accounted for 24 taxa (~15%). Overall, phytoplankton contributed the largest share of total richness, followed by macroinvertebrates, whereas zooplankton had lower richness but played important roles in terms of abundance and biomass. The raw data for the three groups over the three-year period are provided in Supplementary Table S3.

In terms of temporal dynamics, macroinvertebrate richness was relatively stable over the three years. Annual total richness fluctuated narrowly between 57 and 59 taxa, and mean site-level richness was approximately 8–9 taxa, with only minor interannual differences. Richness was slightly higher in 2022 and decreased slightly in 2023, but the magnitude of change was limited. At the seasonal scale, macroinvertebrate richness in May and September was similar, with September typically slightly higher. Most taxa were shared between the two seasons within each year, and only a small number of taxa were season-specific, suggesting a relatively stable “core species pool”. Thus, the macroinvertebrate assemblage appeared to respond primarily through adjustments in dominance and abundance structure rather than through large-scale species turnover, and overall diversity showed limited fluctuation, reflecting longer-term and cumulative environmental conditions.

Zooplankton showed more pronounced interannual variation. Annual richness increased from 36 taxa in 2021 to 41 taxa in 2022 and 53 taxa in 2023, and mean site-level richness increased from approximately 7 taxa to 8–9 taxa, with a further rise in 2023. This pattern indicates an overall increasing trend in zooplankton diversity during the study period. Seasonally, differences in richness between May and September were small within each year, and species overlap between the two seasons was high, suggesting that assemblage composition was largely shaped by the same species pool and that seasonal differences were mainly expressed as changes in abundance magnitude and the replacement of a few dominant taxa. In contrast, the proportion of shared species among different years was notably lower, with a consistent number of newly recorded taxa each year, implying that zooplankton were sensitive to interannual environmental variability and exhibited relatively active compositional updates at the species level.

Phytoplankton richness was consistently higher than that of the other two groups and displayed a gradual increasing trend. Annual richness increased from 73 taxa in 2021 to more than 90 taxa in 2022 and 2023, while mean site-level richness increased from approximately 23 taxa to ~27 taxa. Seasonally, richness in May and September differed only slightly, and overlap between the two seasons was extremely high; in some years, species composition was nearly identical between seasons, indicating strong within-year stability. Notably, however, the number of taxa shared among different years accounted for only a subset of the total, and both 2022 and 2023 included substantial numbers of newly recorded taxa. This suggests that, although the overall algal-group structure remained relatively stable, year-to-year supplementation and replacement at the species level were frequent.

In addition to richness and compositional patterns, several representative indicators of pollution stress and eutrophication showed clear interannual variation across the three biological groups. For macroinvertebrates, the annual mean BMWP index declined from 42.75 in 2021 and 41.88 in 2022 to 30.58 in 2023, whereas the HBI index increased from 5.43 and 5.70 to 6.50 over the same period. At the same time, the mean proportion of intolerant taxa decreased from 35.9% and 35.0% in 2021–2022 to 15.8% in 2023, while tolerant taxa increased from 19.8% to 27.7%, indicating elevated pollution stress in 2023. For zooplankton, trophic-state-related indicators also varied markedly: the E/O index increased from 0.28 in 2021 to 1.09 in 2022 and remained relatively high in 2023 (0.97), while the fertility of zooplankton index rose from 0.41 in 2021 to 1.13 in 2022 and remained above the 2021 level in 2023 (0.62). Filter-feeders abundance further increased to 38.93 in 2023, compared with 11.55 in 2021 and 12.78 in 2022. For phytoplankton, the Palmer algal genus pollution index remained relatively high throughout the study period (annual mean 14.28–17.33), and the proportion of Cyanophyta cell density increased from 27.3% in 2021 and 28.4% in 2022 to 36.9% in 2023, indicating a stronger eutrophication-related signal in that year.

Taken together, the three biotic groups indicate that aquatic communities in Dongjiang Lake were characterized by high biodiversity, a stable core species pool, and varying degrees of interannual compositional updating. Macroinvertebrates were comparatively stable in richness but showed clearer deterioration in pollution-tolerance-related indicators in 2023, whereas zooplankton and phytoplankton were more sensitive at the interannual scale and displayed clearer trophic or eutrophication-related variation. This community and indicator background provides an essential basis for interpreting subsequent spatiotemporal variations in IBI results.

### 3.2. NMDS Analysis of Community Structure

**Figure 4:**
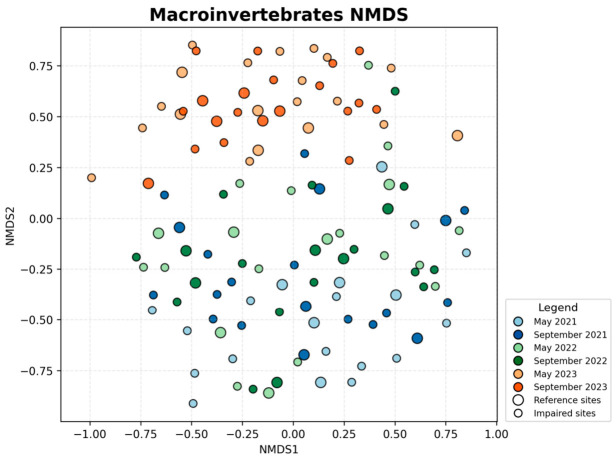
NMDS ordination of macroinvertebrate community structure based on Bray–Curtis dissimilarities (stress = 0.349).

The macroinvertebrate NMDS ordination (Figure 4) showed a relatively dispersed distribution of sampling sites, and the separation by year or season was less apparent than that observed for plankton. The two-dimensional ordination had a stress value of 0.349. Samples from different years and seasons overlapped extensively in ordination space, although a slight upward shift in the 2023 samples along the second axis could still be observed, whereas 2021 and 2022 samples were more frequently distributed in the central to lower portion of the plot. PERMANOVA indicated that macroinvertebrate community composition differed significantly among years (pseudo-F = 5.84, R^2^ = 0.091, *p* = 0.001), whereas the seasonal effect was not significant (pseudo-F = 1.30, R^2^ = 0.010, *p* = 0.070). This pattern is consistent with the overall stability of macroinvertebrate richness and the high seasonal overlap described in Section 3.1, indicating that macroinvertebrate assemblage composition and overall structure remained relatively robust during the study period. Accordingly, the NMDS configuration mainly reflects modest interannual structuring rather than a strong seasonal gradient. Reference and impaired sites almost completely overlapped in the macroinvertebrate NMDS space, suggesting that differences among macroinvertebrate IBI classes are more likely driven by the combined effects of multiple dimensions (e.g., proportions of sensitive vs. tolerant taxa, diversity, and functional feeding structure), rather than by species-composition differences captured by NMDS alone.

**Figure 5:**
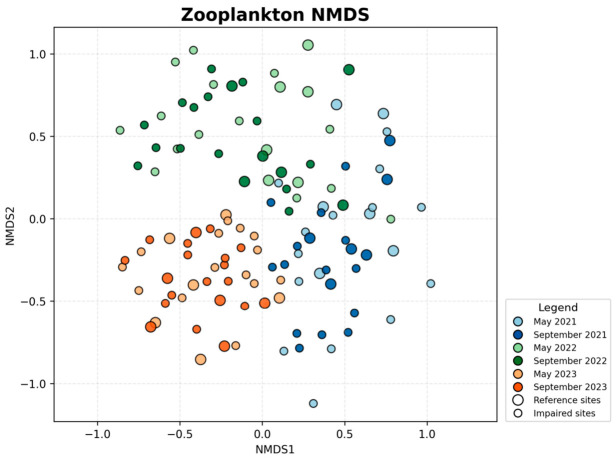
NMDS ordination of zooplankton community structure based on Bray–Curtis dissimilarities (stress = 0.317).

The zooplankton NMDS ordination (Figure 5) showed more evident visual interannual structuring. In the ordination plot, samples from 2022 were mainly clustered in the upper region, 2023 samples were concentrated in the lower region, and 2021 samples were distributed predominantly on the right side, forming three visually differentiated clouds corresponding to the three years. The two-dimensional ordination had a stress value of 0.317. PERMANOVA confirmed significant compositional differences among years (pseudo-F = 9.06, R^2^ = 0.133, *p* = 0.001) and between seasons (pseudo-F = 2.46, R^2^ = 0.018, *p* = 0.001). This pattern agrees with the results in Section 3.1 showing increasing zooplankton richness and mean site-level richness over time and a relatively low proportion of shared species among years, suggesting that zooplankton communities underwent pronounced interannual structural adjustment during the study period. Within each year, May and September samples were positioned relatively close to each other, indicating that seasonal differences were weaker than interannual differences. Sites with different IBI backgrounds also showed substantial overlap in the zooplankton NMDS space, implying that the zooplankton IBI reflects multidimensional community attributes beyond the species-composition contrasts represented by NMDS.

**Figure 6:**
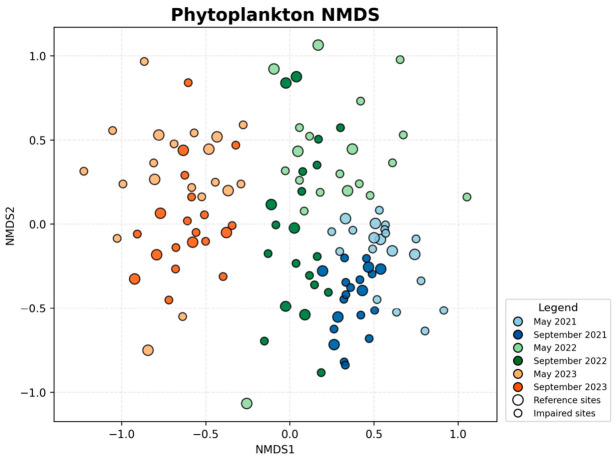
NMDS ordination of phytoplankton community structure based on Bray–Curtis dissimilarities (stress = 0.258).

The phytoplankton NMDS ordination (Figure 6) suggested a clear interannual compositional gradient. Along the first axis, 2021 samples were mainly located on the right, 2022 samples occupied intermediate positions, and 2023 samples shifted overall to the left, forming an approximate temporal sequence from right to left. The two-dimensional ordination had a stress value of 0.258. PERMANOVA showed that phytoplankton community composition differed significantly among years (pseudo-F = 11.61, R^2^ = 0.160, *p* = 0.001) and between seasons (pseudo-F = 5.86, R^2^ = 0.040, *p* = 0.001). This finding is consistent with the previously observed gradual increase in phytoplankton richness and the presence of interannual compositional updating, indicating a progressive adjustment in overall phytoplankton community structure over the three years. Within each year, May and September samples still overlapped substantially in ordination space, suggesting that seasonal differences were present but smaller than the year effect. Reference and impaired sites showed partial overlap in the phytoplankton ordination, indicating that despite differences in environmental context, the two categories were not completely separated in community structure. This pattern also helps explain the spatial overlap of the good-to-moderate phytoplankton IBI classifications reported later.

Overall, the NMDS ordinations and PERMANOVA results indicate that phytoplankton and zooplankton exhibited pronounced interannual shifts in community structure, and that both groups also showed significant seasonal differences. In contrast, macroinvertebrate communities were characterized by significant interannual differentiation but weak seasonal structuring. In all three groups, year explained more variation than season, indicating that interannual turnover was the dominant temporal signal in community composition during the study period. Additional dispersion tests suggested that some significant PERMANOVA results may also reflect unequal within-group dispersion; therefore, the multivariate patterns reported here are interpreted conservatively as evidence of overall community differentiation rather than strictly centroid separation. Importantly, these NMDS patterns should be interpreted as community-composition background against which the IBI results can be understood, rather than as direct equivalents of IBI scores. For zooplankton and phytoplankton, the stronger interannual compositional shifts observed in ordination space were broadly consistent with the tendency toward lower IBI classes in 2023, suggesting that the disturbance events in that year altered both species composition and trophic or water-quality related ecological attributes. By contrast, the weaker temporal separation of macroinvertebrate assemblages in NMDS does not imply low sensitivity of the B-IBI, because the macroinvertebrate index also incorporates tolerance-related and functional-feeding information that can shift even when overall taxonomic overlap remains relatively high in ordination space.

### 3.3. IBI Result

Among the upstream, lake-area, and downstream sites that were assigned to the impaired category under the local reference-impaired calibration, IBI assessments during 2021–2023 showed pronounced temporal dynamics and clear differences among biotic groups. Overall, impaired sites were most frequently classified as moderate or good, with smaller proportions classified as poor or fair and occasional excellent ratings. Under the present locally calibrated framework, the highest relative condition classifications were observed in 2022, when IBIs across the three spatial subregions and the three biotic groups were mostly within the good-to-excellent classes. In 2021, assessments were dominated by moderate-to-good classes, but localized fair and poor outcomes were already present. In 2023, the proportion of poor-to-fair classifications increased again across multiple subregions and biotic groups, indicating a relative shift toward lower IBI classes within the study system in that year. Seasonally, differences between May and September were also evident: in some years, September (after the high-flow season) showed shifts toward lower IBI classes for macroinvertebrates and zooplankton, whereas phytoplankton in 2023 exhibited a widespread shift toward lower classes in May followed by a partial shift back toward higher classes in September, reflecting a rapid response to hydrological and trophic fluctuations.

Across the three biotic groups, the macroinvertebrate IBI showed the largest interannual amplitude and was the group most likely to yield poor–fair ratings at impaired sites. In 2021, impaired sites in the upstream and downstream subregions recorded relatively high frequencies of fair and poor macroinvertebrate IBI classes, while the lake area was mostly moderate, consistent with a sensitive benthic response in disturbed river–reservoir transition and embayment environments. In 2022, macroinvertebrate IBI values increased markedly across all three subregions: upstream impaired sites were mainly good, downstream sites were largely good–excellent, and the lake area was dominated by good–moderate, suggesting substantial improvement in benthic habitat conditions throughout the basin. In 2023, macroinvertebrate IBI patterns diverged again. The proportion of poor–fair outcomes increased at upstream and downstream impaired sites; notably, the downstream subregion shifted from being “predominantly excellent” in 2022 to “predominantly moderate, with mixed fair and poor” in 2023. In contrast, the lake-area impaired sites remained relatively stable at an intermediate moderate–good level, indicating a buffering effect and higher resilience. Taken together, the macroinvertebrate IBI at impaired sites followed a trajectory of lower status in 2021, overall improvement in 2022, and renewed divergence in 2023, and it consistently revealed a spatial pattern in which upstream and downstream reaches were more sensitive to disturbance variation than the lake area.

In comparison, the zooplankton and phytoplankton IBIs generally yielded higher assessments at impaired sites than the macroinvertebrate IBI, but both groups also exhibited clear degradation signals in 2023. For zooplankton, during 2021–2022, May assessments across upstream, lake-area, and downstream subregions were mostly good–excellent, and September assessments generally remained good or above, with only occasional poor records in the lake area in some years. In particular, 2022 showed near-universal good–excellent zooplankton IBI classes across the three subregions, indicating favorable ecological conditions. However, in 2023, the downstream impaired sites showed a notable decline, with a marked increase in fair–poor outcomes; the lake area also shifted from excellent–good toward good–moderate with some fair, whereas the upstream subregion remained largely excellent–good, with only a few poor–fair records in specific seasons. This resulted in a clear longitudinal contrast, with the upstream remaining relatively better and downstream degradation being more pronounced.

For phytoplankton, assessments during 2021–2022 across the three subregions were dominated by excellent–good, and most sampling periods were comparable to, or approached, reference-site conditions. In May 2023, however, phytoplankton IBI values across all three subregions showed a widespread short-term decline toward fair–poor, particularly at upstream and downstream impaired sites, suggesting that trophic status and/or organic pollution levels may have increased substantially during that period. By September, phytoplankton IBI values partially recovered: upstream and lake-area subregions returned to predominantly excellent–good, while the downstream subregion improved to some extent but still retained a non-negligible proportion of fair–poor classifications.

**Figure 7:**
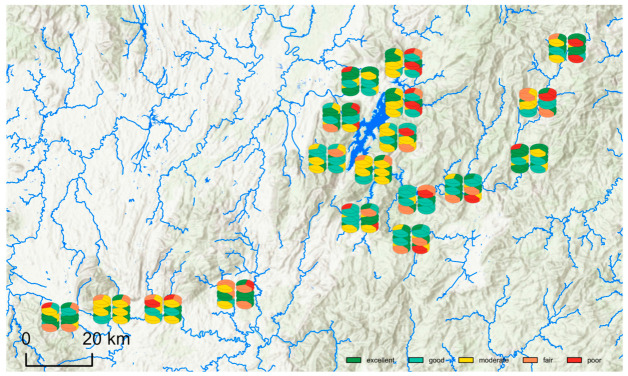
IBI results across sampling sites and periods. For each site, the left pie chart represents May, and the right pie chart represents September; within each pair, the bottom layer corresponds to 2021, the middle layer to 2022, and the top layer to 2023. Within each pie chart, the lower sector denotes phytoplankton, the upper-left sector denotes macroinvertebrates, and the upper-right sector denotes zooplankton.

Collectively, these results indicate that some sites categorized as impaired according to background disturbance still attained good-to-excellent classes in certain years and biotic groups, suggesting that the three IBIs captured relative spatiotemporal variation within Dongjiang Lake rather than a rigid binary separation between all reference and all impaired assemblages. Formal within-dataset sensitivity analysis further showed that the IBI results were highly stable under reasonable alternative metric substitutions. Across all biotic groups, overall Spearman correlations between the main and alternative IBI scores were 0.984 for Alt A and 0.977 for Alt B, the mean absolute score differences were 2.00 and 2.16 points, exact agreement in five-level classification was 90.9% and 91.3%, and same-or-adjacent class agreement reached 100.0% for both alternative sets. These results indicate that the overall site-classification patterns were not materially altered by reasonable substitutions among candidate metrics with similar screening performance (Tables S5 and S6). At the same time, the three-group comparison highlights distinct response characteristics: macroinvertebrates were more sensitive to long-term and cumulative disturbance, whereas zooplankton and phytoplankton responded more rapidly to nutrient conditions and short-term water-quality fluctuations. Clear differences were also evident among upstream, lake-area, and downstream subregions in both the magnitude of response and the apparent recovery capacity of the three IBIs. The IBI assessment results are summarized in Figure 7, the corresponding assessment categories are listed in Table S4, and the stability-analysis results are summarized in Tables S5 and S6.

## 4. Discussion

### 4.1. Interpretation of IBI-Based Relative Ecological Condition Patterns in Dongjiang Lake

Based on the integrated assessment using the three IBI frameworks for macroinvertebrates, zooplankton, and phytoplankton, impaired sites in Dongjiang Lake were generally classified within the moderate-to-good range during 2021–2023. Across the three years, IBI classes at most sites were concentrated within the moderate–good range, with smaller proportions classified as poor–fair and occasional excellent outcomes. This pattern indicates that impaired sites were subject to measurable human disturbance but had not broadly transitioned into a severely degraded state and still exhibited recovery potential in certain years and for certain biotic groups. These results should be interpreted as relative IBI-based condition categories within Dongjiang Lake rather than as independently validated absolute measures of ecosystem health.

Temporally, the IBI results followed an overall trajectory of relatively lower status in 2021, broad improvement in 2022, and renewed decline in 2023. The lower classifications observed in 2023 may have been associated with two concurrent disturbance processes in the study area: dredging activities and heavy rainfall events during that year. Dredging can resuspend bottom sediments, disturb benthic habitats, and promote the release of nutrients and organic matter from previously deposited materials into the water column, while heavy rainfall can increase catchment runoff, external nutrient and particulate inputs, and short-term hydrological instability. Together, these processes may help explain why relative ecological condition appeared lower in 2023 than in 2022. Overall, the temporal pattern suggests fluctuation rather than a unidirectional improvement or persistent deterioration during the study period.

Spatially, the IBIs revealed clear differences along the river–reservoir continuum, with distinct patterns across the upstream, lake-area, and downstream subregions. Impaired sites in the upstream and downstream sections showed larger fluctuations in IBI class and were more likely to shift into fair–poor categories in 2021 and 2023, indicating stronger sensitivity to disturbances such as catchment inflows, point-source discharges, and reservoir regulation. This spatial pattern is also consistent with the likely effects of the dredging activities and heavy rainfall that occurred in 2023. Heavy rainfall would be expected to enhance runoff-driven inputs of suspended particles and nutrients into upstream reaches, whereas downstream sections may experience the combined influence of materials transported through the reservoir system, regulated outflow, and additional local disturbance. In contrast, impaired sites in the lake area were more consistently classified as moderate–good, with comparatively smaller spatial variability, suggesting a buffering effect against short-term nutrient inputs and water-quality fluctuations. Because the lake area has a larger water volume and longer residence time, it may partially smooth short-term hydrological and water-quality perturbations, whereas upstream and downstream reaches are more directly exposed to external loading and disturbance propagation. These findings imply that sensitive upstream reaches and downstream confluence sections should be prioritized in ecological management, while the lake area currently provides a degree of ecological buffering.

The three biotic-group IBIs were complementary in terms of both temporal sensitivity and dominant pressure types. The macroinvertebrate B-IBI exhibited the largest interannual variation and was most likely to yield fair–poor classifications at impaired sites. Clear degradation signals were already evident in upstream and downstream sections in 2021, followed by broad recovery to good–excellent in 2022 and renewed declines in 2023—most notably in the downstream section, which shifted from being “predominantly excellent” to “predominantly moderate or below”. This pattern is consistent with macroinvertebrate life-history characteristics (longer life cycles, limited mobility, and high sensitivity to persistent habitat disturbance), but it may also reflect the direct habitat disturbance associated with dredging in 2023, because benthic habitats are particularly vulnerable to sediment removal, substrate instability, and resuspension of fine materials. In contrast, the zooplankton Z-IBI and phytoplankton P-IBI generally remained at good–excellent levels during 2021–2022, reflecting overall favorable water quality and trophic conditions in those years. However, both indices deteriorated markedly in 2023: Z-IBI declined at downstream and some lake-area sites, whereas P-IBI showed synchronous degradation toward fair–poor across all three subregions in May 2023, followed by partial recovery in September. This behavior is consistent with the rapid responsiveness of plankton-based IBIs to short-term environmental fluctuations, including nutrient pulses, increased suspended matter, reduced water clarity, and hydrological instability associated with heavy rainfall and sediment disturbance. Thus, the contrasting responses among the three biotic groups likely reflect differences in the temporal scale at which dredging- and rainfall-related disturbances were translated into biological change.

Taken together, multitaxon IBIs provide a tiered and informative overview of relative ecological condition patterns in Dongjiang Lake. The macroinvertebrate-based index primarily captures long-term pressure on benthic substrates and habitat quality and is effective for identifying persistently stressed reaches and embayments, whereas zooplankton- and phytoplankton-based indices are more sensitive to short-term variability in nutrients and organic pollution and can therefore support early warning for eutrophication and water-quality deterioration. By jointly applying the three IBIs, this study distinguishes which biotic groups were most sensitive to specific pressure types and which subregions were most prone to degradation, thereby providing targeted scientific evidence for zoned management, pollutant-load reduction, and ecological restoration planning in the Dongjiang Lake Basin. In this context, the NMDS results help clarify why the IBI patterns differed among biotic groups. For zooplankton and phytoplankton, the clearer interannual separation in ordination space was broadly consistent with the lower IBI classes observed in 2023, indicating that dredging and heavy rainfall in that year likely affected both community composition and trophic- or water-quality-related ecological attributes. For macroinvertebrates, however, the weaker temporal separation in NMDS suggests that overall species composition remained relatively overlapping across survey periods, whereas the B-IBI was still able to register deterioration because it captures shifts in tolerance structure, functional feeding composition, and habitat-related stress that are not always expressed as strong ordination separation. Thus, NMDS and IBI should be viewed as complementary rather than redundant descriptors of ecological change in the present study.

### 4.2. Correlations Between IBI and Other Metrics

To examine whether the three multimetric indices showed ecologically interpretable directional consistency with commonly used descriptors, we selected seven representative metrics for each biotic group and performed correlation analyses. In addition to these internal consistency checks, selected established or conventional indices were considered as limited comparative benchmarks where appropriate, particularly BMWP and HBI for macroinvertebrates and Palmer/GDI/DQ-related descriptors for phytoplankton. These analyses were intended only as internal consistency checks and ecological interpretation tools, not as independent validation tests, because the indices and the representative metrics were derived from related biological information within the same dataset.

**Figure 8:**
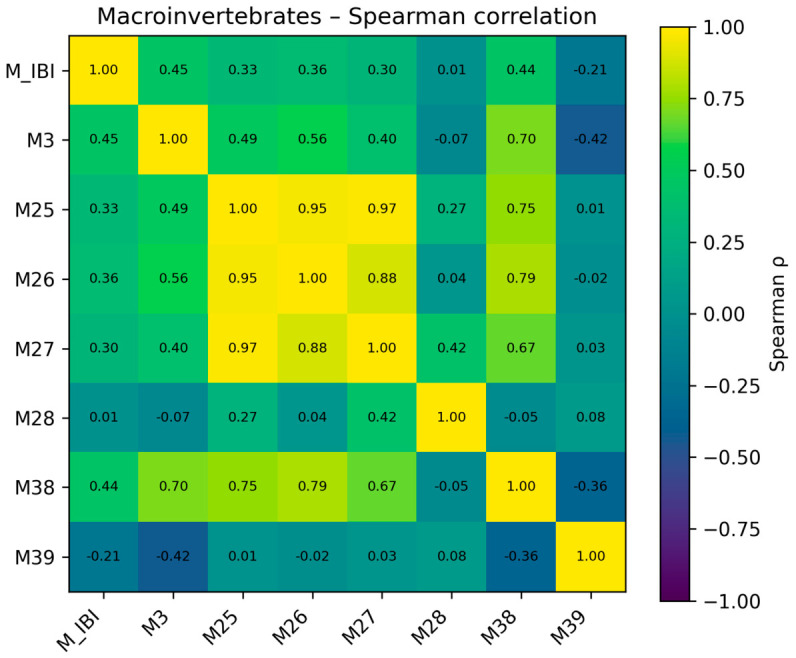
Spearman correlation results for selected macroinvertebrate metrics.

For macroinvertebrates, the B-IBI showed overall weak to moderate associations with single metrics. Spearman correlations between B-IBI and EPT richness (M3) as well as the BMWP index (M38) were both approximately 0.44. Correlations with the Shannon–Wiener index (M25), Margalef index (M26), and Simpson index (M27) were around 0.30–0.36, all positive, indicating that the macroinvertebrate IBI partly captured variation in taxonomic richness, the presence of sensitive taxa, and composite tolerance patterns. In contrast, the relationship between B-IBI and Pielou evenness (M28) was close to zero, and the correlation with the HBI index (M39) was weakly negative (ρ ≈ −0.21). This suggests that, within the impaired-site dataset considered here, fluctuations in evenness and variation in a single pollution index did not translate into a simple linear response in the IBI score. The positive directional relationship with BMWP provides limited comparative context against an established macroinvertebrate benchmark, suggesting that the locally calibrated B-IBI was broadly aligned with a conventional assessment tool while still incorporating additional multimetric information tailored to the present reservoir system. Overall, B-IBI was directionally consistent with conventional diversity and tolerance indices but was not redundant with any single metric. The Spearman correlation results for macroinvertebrates are shown in Figure 8.

**Figure 9:**
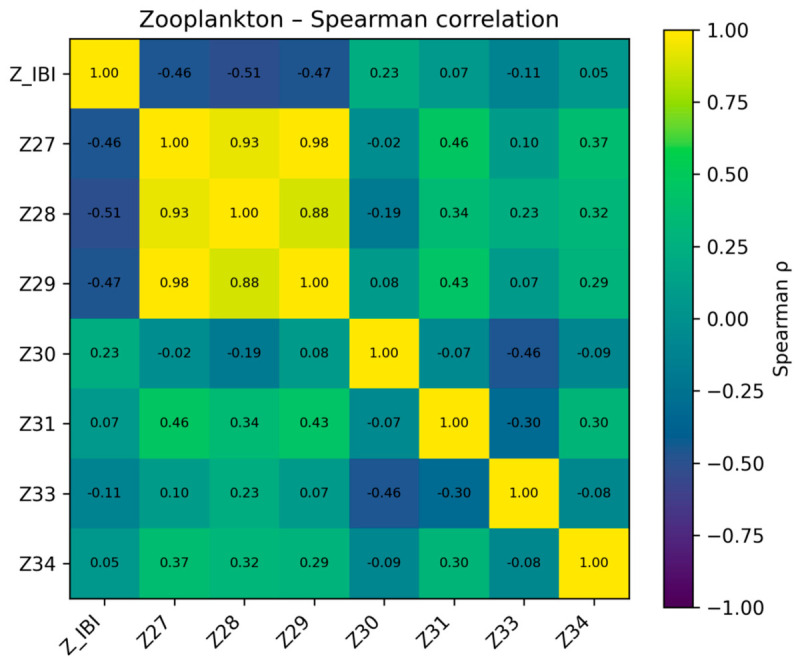
Spearman correlation results for selected zooplankton metrics.

For zooplankton, a distinct pattern emerged. The Z-IBI showed moderate negative correlations with the three diversity indices (Z27, Z28, Z29), with Spearman coefficients ranging from approximately −0.46 to −0.51, whereas correlations with Pielou evenness (Z30), predator abundance (Z31), the B/T index (Z33), and the E/O index (Z34) were generally weak (mostly |ρ| ≤ 0.30). In other words, within the impaired-site samples, sites with higher zooplankton diversity sometimes corresponded to lower Z-IBI scores. This pattern likely reflects situations in which eutrophication and the dominance of small, pollution-tolerant taxa produce an apparent increase in “diversity” without an improvement in biological integrity. Accordingly, Z-IBI appears to penalize assemblages dominated by tolerant taxa and characterized by reduced proportions of sensitive groups, rather than simply tracking diversity as an indicator of integrity. The Spearman correlation results for zooplankton are shown in Figure 9.

**Figure 10:**
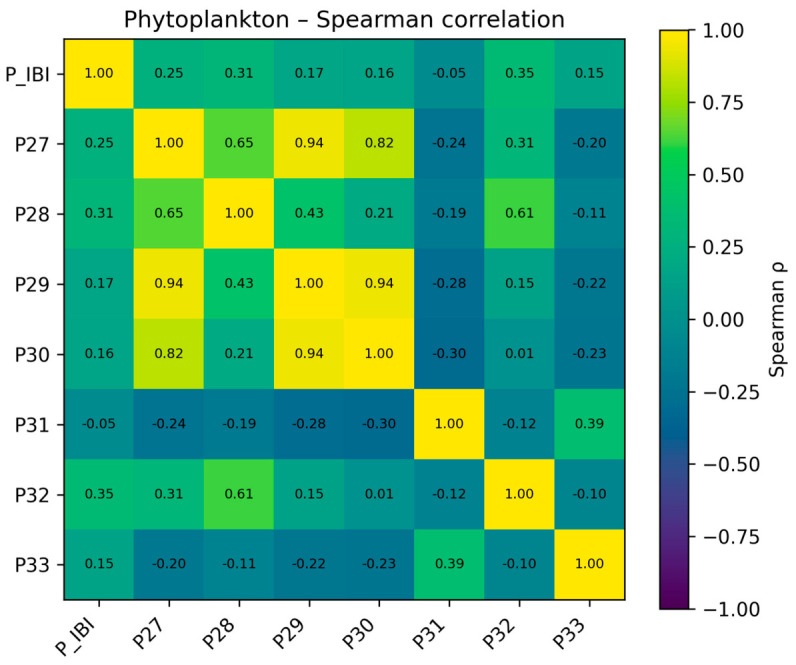
Spearman correlation results for selected phytoplankton metrics.

For phytoplankton, correlations between the P-IBI and diversity or water-quality indicator indices were also generally weak. Spearman coefficients between P-IBI and the Shannon–Wiener index (P27), Margalef index (P28), and the Palmer algal genus pollution index (P32) were approximately 0.25, 0.31, and 0.35, respectively, representing weak to moderate positive relationships. Correlations with the Simpson index (P29), Pielou evenness (P30), and the Generic Diatom Index (P33) were typically around 0.15–0.17, while the association with the Diatom Quotient (P31) was slightly negative (ρ ≈ −0.05). These results indicate that P-IBI was broadly consistent in direction with changes in algal diversity and organic pollution indicators, but the strength of association was limited, suggesting that no single phytoplankton index fully represents the integrated characterization of community structure and trophic conditions captured by P-IBI. The Spearman correlation results for phytoplankton are shown in Figure 10.

Taken together, the results across the three biotic groups indicate that IBIs were generally weakly to moderately correlated with conventional diversity indices and commonly used water-quality indicator indices. This pattern is consistent with the expectation that a multimetric index integrates complementary ecological dimensions and should not be reducible to any single component metric. These correlations should therefore be interpreted as evidence of internal consistency only, not as external validation of predictive performance. Likewise, the temporal robustness discussed in this study was evaluated only within the 2021–2023 monitoring dataset, although the added within-dataset sensitivity analysis showed that reasonable alternative metric substitutions did not materially alter IBI scores or classification patterns. Broader external validation against independent environmental gradients and longer time-series data would further strengthen the transferability of the framework.

Ecologically, the absence of very strong correlations does not indicate that the IBIs lack interpretability; rather, it highlights their integrative role within the present local assessment framework. For macroinvertebrates and phytoplankton, the IBIs were directionally consistent with diversity and classical pollution indices, whereas for zooplankton the IBI captured degradation scenarios in which diversity increased despite dominance by tolerant taxa. Within the current manuscript, the IBIs are therefore best interpreted as locally calibrated multimetric tools for relative ecosystem-health classification in Dongjiang Lake.

## 5. Conclusions

This study demonstrates that a locally calibrated multitaxon IBI framework can be applied to a deep reservoir-type lake to capture relative ecological-condition patterns across the upstream–lake–downstream continuum of Dongjiang Lake. By integrating macroinvertebrates, zooplankton, and phytoplankton within one assessment framework, the study extends IBI application beyond single-group, primarily river-oriented approaches and shows the value of multitaxon integration for reservoir-system assessment.

The main finding of this study is that the three biotic groups provided complementary ecological signals rather than redundant information. Macroinvertebrates were more effective in reflecting habitat-related and cumulative disturbance, whereas zooplankton and phytoplankton responded more rapidly to short-term changes in nutrients, hydrology, and water quality. This complementarity indicates that ecological condition in deep reservoir-type lakes cannot be fully characterized by any single biological group alone and that multitaxon integration improves the interpretability of biomonitoring results.

Under the present local calibration, the framework also revealed clear temporal and spatial heterogeneity in Dongjiang Lake, with stronger fluctuations in upstream and downstream sections than in the lake area and with lower relative condition classes observed in 2023. These patterns highlight the practical value of the framework for identifying spatially sensitive subregions and distinguishing between pressure types that operate on different time scales. Therefore, the study provides not only a case application for Dongjiang Lake, but also a methodological reference for integrated relative ecological-condition assessment in similar deep reservoir-type lake systems. Although the added within-dataset stability analysis showed high robustness to reasonable alternative metric substitutions, future work should still strengthen long-term robustness testing and external validation against independent ecological benchmarks.

## Data Availability

The original contributions presented in this study are included in the article/Supplementary Materials. Further inquiries can be directed to the corresponding author.

## Supplementary Materials

The following supporting information can be downloaded at: https://www.mdpi.com/article/10.3390/biology15100765/s1, Table S1: Coordinates and basic information for the 20 sampling sites in Dongjiang Lake; Table S2: Full candidate metric lists and serial-number details for the macroinvertebrate, zooplankton, and phytoplankton IBI frameworks; Table S3: Raw community data for macroinvertebrates, zooplankton, and phytoplankton across the six surveys conducted during 2021–2023; Table S4: IBI classification results for each site, sampling period, and biotic group; Table S5: Main and alternative metric sets used in the within-dataset sensitivity/stability analysis for each biotic group × year × month subset; Table S6: Stability statistics comparing the main metric sets with Alt A and Alt B, including Spearman rho, mean absolute difference, exact class agreement, same-or-adjacent class agreement, and weighted kappa; File S7: Boxplots showing the discriminatory performance of candidate metrics between the operational reference and impaired groups for each sampling event; File S8: Correlation matrices used for redundancy screening of candidate metrics for each sampling event.

## References

[1] Karr J.R. (1981). Assessment of Biotic Integrity Using Fish Communities. Fisheries.

[2] Ruaro R., Gubiani E.A. (2013). A scientometric assessment of 30 years of the Index of Biotic Integrity in aquatic ecosystems: Applications and main flaws. Ecol. Indic..

[3] Karr J.R. (1991). Biological Integrity: A Long-Neglected Aspect of Water Resource Management. Ecol. Appl..

[4] Karr J.R. (2010). Defining and measuring river health. Freshw. Biol..

[5] Wu N., Cai Q., Fohrer N. (2012). Development and evaluation of a diatom-based index of biotic integrity (D-IBI) for rivers impacted by run-of-river dams. Ecol. Indic..

[6] Hughes R.M., Howlin S., Kaufmann P.R. (2004). A Biointegrity Index (IBI) for Coldwater Streams of Western Oregon and Washington. Trans. Am. Fish. Soc..

[7] Chin A.T.M., Tozer D.C., Walton N.G., Fraser G.S. (2015). Comparing disturbance gradients and bird-based indices of biotic integrity for ranking the ecological integrity of Great Lakes coastal wetlands. Ecol. Indic. Integr. Monit. Assess. Manag..

[8] Lunde K.B., Resh V.H. (2012). Development and validation of a macroinvertebrate index of biotic integrity (IBI) for assessing urban impacts to Northern California freshwater wetlands. Environ. Monit. Assess..

[9] Kane D.D., Gordon S.I., Munawar M., Charlton M.N., Culver D.A. (2009). The Planktonic Index of Biotic Integrity (P-IBI): An approach for assessing lake ecosystem health. Ecol. Indic..

[10] Stoddard J.L., Larsen D.P., Hawkins C.P., Johnson R.K., Norris R.H. (2006). Setting expectations for the ecological condition of streams: The concept of reference condition. Ecol. Appl..

[11] Bedoya D., Novotny V., Manolakos E.S. (2009). Instream and offstream environmental conditions and stream biotic integrity: Importance of scale and site similarities for learning and prediction. Ecol. Model..

[12] Kerans B.L., Karr J.R. (1994). A Benthic Index of Biotic Integrity (B-IBI) for Rivers of the Tennessee Valley. Ecol. Appl..

[13] Weisberg S.B., Ranasinghe J.A., Dauer D.M., Schaffner L.C., Frithsen D.J.B. (1997). An estuarine benthic index of biotic integrity (B-IBI) for Chesapeake Bay. Estuaries.

[14] Jun Y.C., Won D.H., Lee S.H., Kong D.S., Hwang S.J. (2012). A Multimetric Benthic Macroinvertebrate Index for the Assessment of Stream Biotic Integrity in Korea. Int. J. Environ. Res. Public Health.

[15] Chainho P., Chaves M.L., Costa J.L., Costa M.J., Dauer D.M. (2008). Use of multimetric indices to classify estuaries with different hydromorphological characteristics and different levels of human pressure. Mar. Pollut. Bull..

[16] Ode P.R., Rehn A.C., May J.T. (2005). A Quantitative Tool for Assessing the Integrity of Southern Coastal California Streams. Environ. Manag..

[17] Zhang Q., Yang T., Wan X., Wang Y., Wang W. (2021). Community characteristics of benthic macroinvertebrates and identification of environmental driving factors in rivers in semi-arid areas—A case study of Wei River Basin, China. Ecol. Indic..

[18] Lougheed V.L., Chow-Fraser P. (2002). Development and Use of a Zooplankton Index of Wetland Quality in the Laurentian Great Lakes Basin. Ecol. Appl..

[19] Carpenter K.E., Johnson J.M., Buchanan C. (2006). An index of biotic integrity based on the summer polyhaline zooplankton community of the Chesapeake Bay. Mar. Environ. Res..

[20] Mutethya E., Yongo E., Zhang P., Guo Z., Changqing Y. (2024). Ecological health assessment using zooplankton index of biotic integrity (Z-IBI) in urban rivers in Hainan Island, China. Environ. Monit. Assess..

[21] Wu N., Schmalz B., Fohrer N. (2012). Development and testing of a phytoplankton index of biotic integrity (P-IBI) for a German lowland river. Ecol. Indic..

[22] Gao W., Xiong F., Lu Y., Qu X., Xin W., Chen Y. (2023). Development of a phytoplankton-based index of biotic integrity for ecological health assessment in the Yangtze River. Ecol. Process..

[23] Zhu H., Hu X.-D., Wu P.-P., Chen W.-M., Wu S.-S., Li Z.-Q., Zhu L., Xi Y.-L., Huang R. (2021). Development and Testing of the Phytoplankton Biological Integrity Index (P-IBI) in Dry and Wet Seasons for Lake Gehu. Ecol. Indic..

[24] Padisák J., Borics G., Grigorszky I., Soróczki-Pintér É. (2006). Use of Phytoplankton Assemblages for Monitoring Ecological Status of Lakes within the Water Framework Directive: The Assemblage Index. Hydrobiologia.

[25] Wu Z., Wang F., Wang X., Li K., Zhang L. (2023). Water quality assessment using phytoplankton functional groups in the middle-lower Changjiang River, China. Limnologica.

[26] Wu Z., Kong M., Cai Y., Wang X., Li K. (2019). Index of biotic integrity based on phytoplankton and water quality index: Do they have a similar pattern on water quality assessment? A study of rivers in Lake Taihu Basin, China. Sci. Total Environ..

[27] Hu X., Hu M., Zhu Y., Wang G., Xue B., Shrestha S. (2022). Phytoplankton community variation and ecological health assessment for impounded lakes along the eastern route of China’s South-to-North Water Diversion Project. J. Environ. Manag..

[28] You Q., Yang W., Jiana M., Hu Q. (2021). A comparison of metric scoring and health status classification methods to evaluate benthic macroinvertebrate-based index of biotic integrity performance in Poyang Lake wetland. Sci. Total Environ..

[29] Yang Z., Zhu D., Zhu Q., Hu L., Chen X. (2020). Development of new fish-based indices of biotic integrity for estimating the effects of cascade reservoirs on fish assemblages in the upper Yangtze River, China. Ecol. Indic..

[30] Tan X., Ma P., Bunn S.E., Zhang Q. (2015). Development of a benthic diatom index of biotic integrity (BD-IBI) for ecosystem health assessment of human dominant subtropical rivers, China. J. Environ. Manag..

[31] Wang W., Cai W. (2016). Index of Biotic Integrity and Its Application to Aquatic Ecological Health Assessment. J. Ecol. Rural. Environ..

[32] China National Environmental Monitoring Centre (2021). Technical Specifications for Aquatic Ecological Monitoring—Freshwater Benthic Macroinvertebrates.

[33] Wang Y.Y. (2020). Atlas of Common Aquatic Organisms in Chinese River Basins.

[34] Morse J.C., Yang L., Tian L. (1994). Aquatic Insects of China Useful for Monitoring Water Quality.

[35] Wang J.C., Wang X.H. (2011). Chironomid Larvae of Northern China.

[36] China National Environmental Monitoring Centre (2022). Technical Specifications for Freshwater Zooplankton Monitoring.

[37] Wang J.J. (1952). Freshwater Rotifers of China.

[38] Jiang X.Z., Du N.S. (1979). Fauna Sinica, Arthropoda Crustacea: Freshwater Cladocera.

[39] Shen J.R., Dai A.Y., Zhang C.Z., Li Z.Y., Song D.X., Song Y.Z., Chen G.X. (1979). Fauna Sinica, Arthropoda Crustacea: Freshwater Copepoda.

[40] China National Environmental Monitoring Centre (2022). Technical Specifications for Aquatic Ecological Monitoring—Freshwater Phytoplankton.

[41] Hu H.J., Wei Y.X. (2006). The Freshwater Algae of China: Systematics, Taxonomy and Ecology.

[42] Editorial Committee of Flora Algarum Sinicarum Aquae Dulcis (1998–2016). Flora Algarum Sinicarum Aquae Dulcis.

[43] Zhang Z.S., Huang X.F. (1991). Methods for the Study of Freshwater Plankton.

[44] Zhu H., Zhang Y.Z., Peng Y.C., Shi B.C., Liu T., Dong H.B., Wang Y., Ren Y.C., Xi Y.L. (2023). Assessing the ecological health of the Qingyi River Basin using multi-community indices of biotic integrity. Ecol. Indic..

[45] GBIF Secretariat (2023). GBIF Backbone Taxonomy. Checklist Dataset.

[46] Schoch C.L., Stacy C., Mikhail D., Hotton C.L., Sivakumar K., Rogneda K., Detlef L., Richard M., Kathleen O.N., Barbara R. (2020). NCBI Taxonomy: A comprehensive update on curation, resources and tools. Database.

[47] Schmidt-Kloiber A., Hering D. (2015). www.freshwaterecology.info—An online tool that unifies, standardises and codifies more than 20,000 European freshwater organisms and their ecological preferences. Ecol. Indic..

[48] Wang F.J. (2007). Evaluation of Water Quality and the Type of Nourishment in the Eastern Half of Lake Chaohu by Means of Plankton. Master’s Thesis.

[49] Palmer C.M. (1969). A Composite Rating of Algae Tolerating Organic Pollution. J. Phycol..

[50] Python Software Foundation Python Language Reference, Version 3.11; Python Software Foundation: Wilmington, DE, USA, 2023. https://docs.python.org/3/reference/.

[51] Harris C.R., Millman K.J., Walt S.J., Gommers R., Virtanen P., Cournapeau D., Wieser E., Taylor J., Berg S., Smith N.J. (2020). Array programming with NumPy. Nature.

[52] Hunter J.D. (2007). Matplotlib: A 2D Graphics Environment. Comput. Sci. Eng..

[53] Clarke K.R. (1993). Non-parametric multivariate analyses of changes in community structure. Austral Ecol..

[54] Spearman C. (2010). The proof and measurement of association between two things. Int. J. Epidemiol..

