# Supplementary figures and images for: Development of Multitaxon Indices of Biotic Integrity for Aquatic Ecosystem Health Assessment in Dongjiang Lake
Source: Biology (Basel). 2026 May 11;15(10):765. doi: 10.3390/biology15100765 (PMC13203498; doi:10.3390/biology15100765)

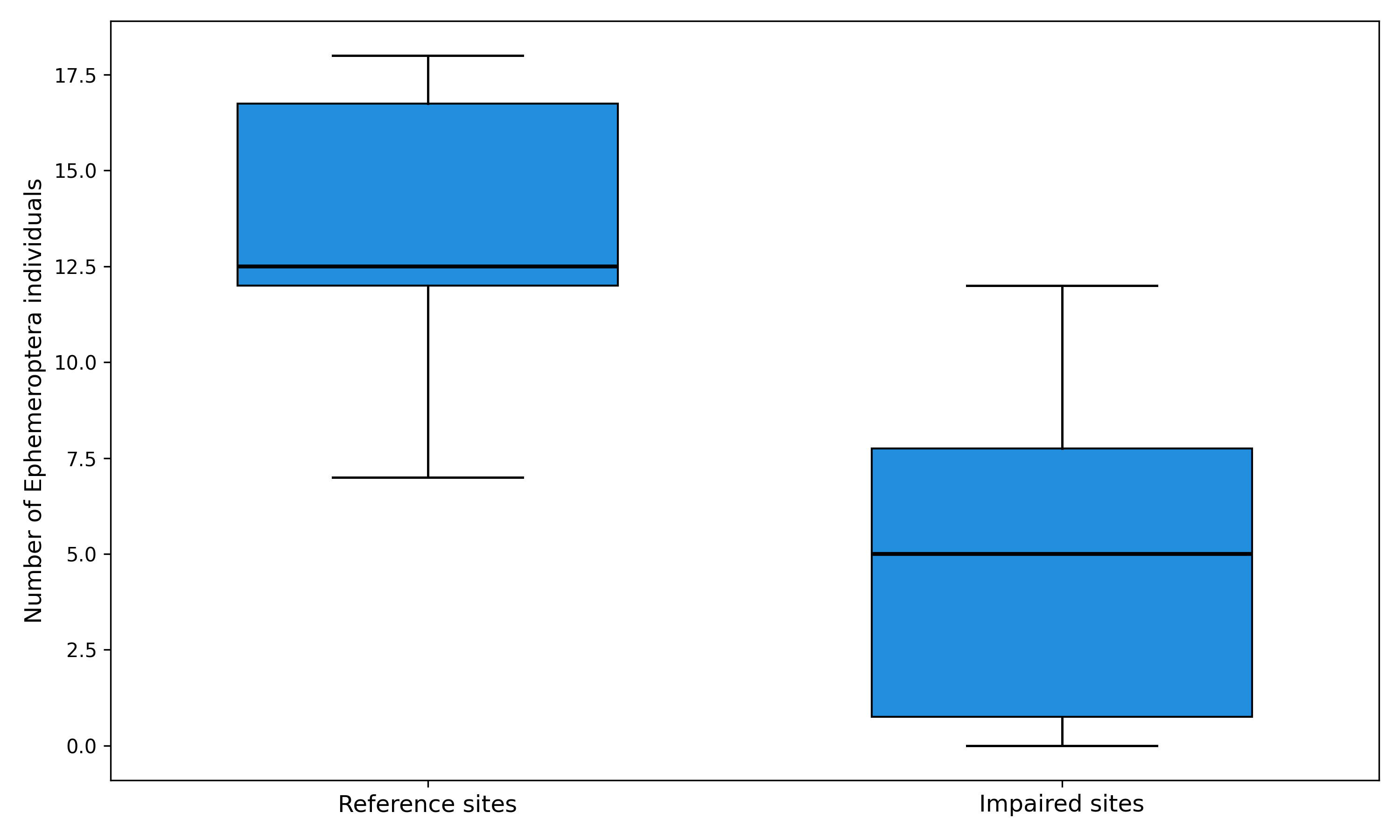

Supplement: Supplementary file 1 [file biology-15-00765-s001.zip › Supplementary/File S7/macroinvertebrates_2021_05/M13_Number of Ephemeroptera individuals.png]

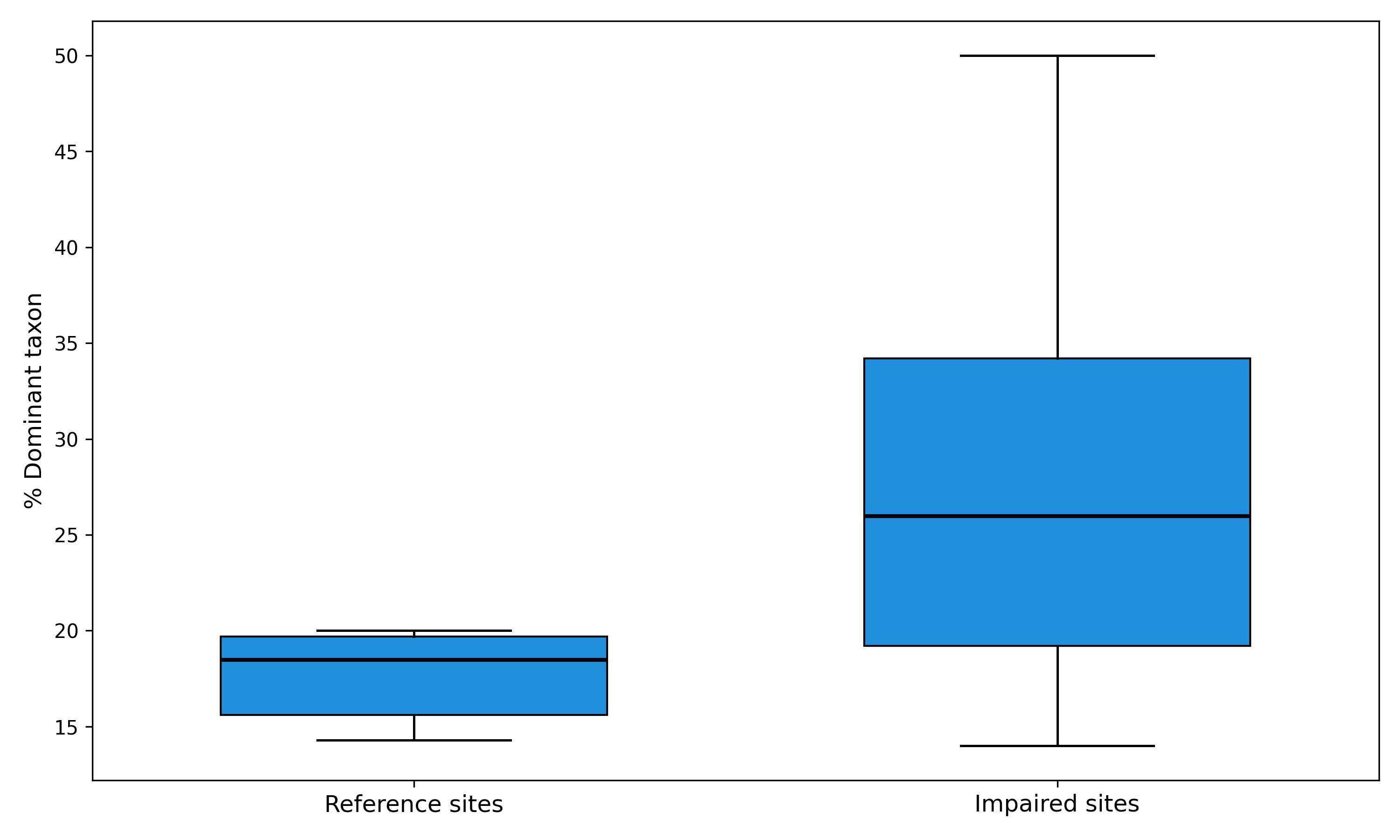

Supplement: Supplementary file 1 [file biology-15-00765-s001.zip › Supplementary/File S7/macroinvertebrates_2021_05/M16_% Dominant taxon.png]

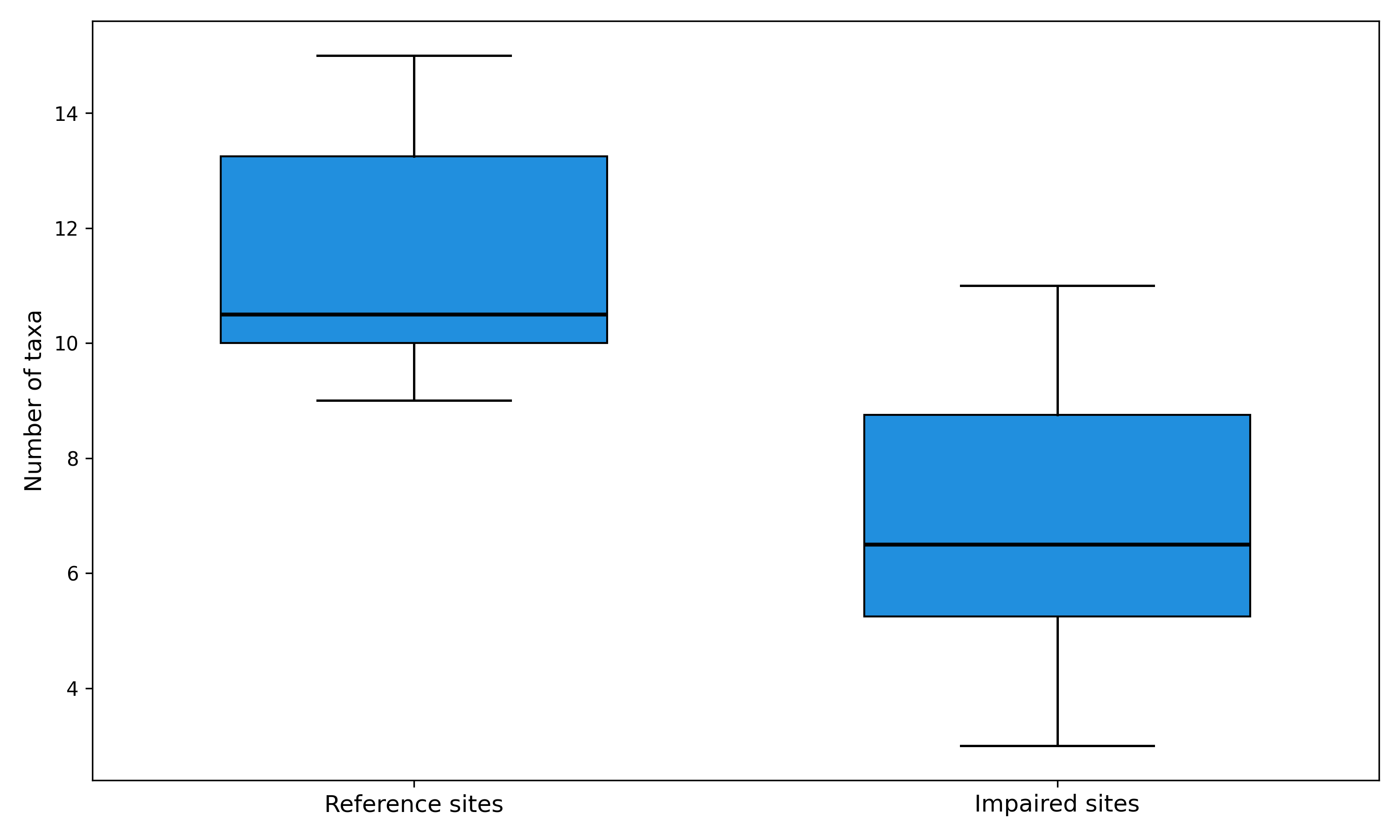

Supplement: Supplementary file 1 [file biology-15-00765-s001.zip › Supplementary/File S7/macroinvertebrates_2021_05/M1_Number of taxa.png]

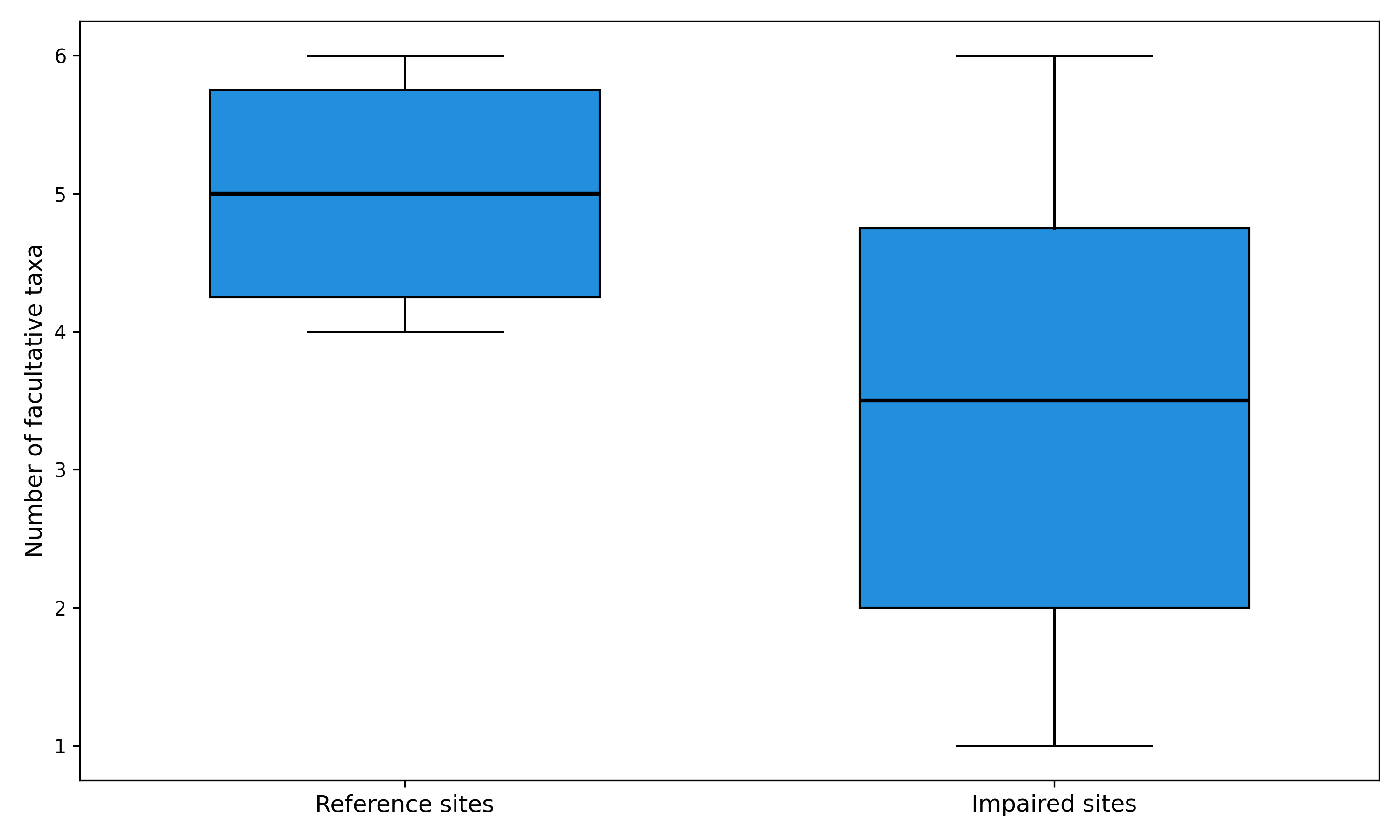

Supplement: Supplementary file 1 [file biology-15-00765-s001.zip › Supplementary/File S7/macroinvertebrates_2021_05/M31_Number of facultative taxa.png]

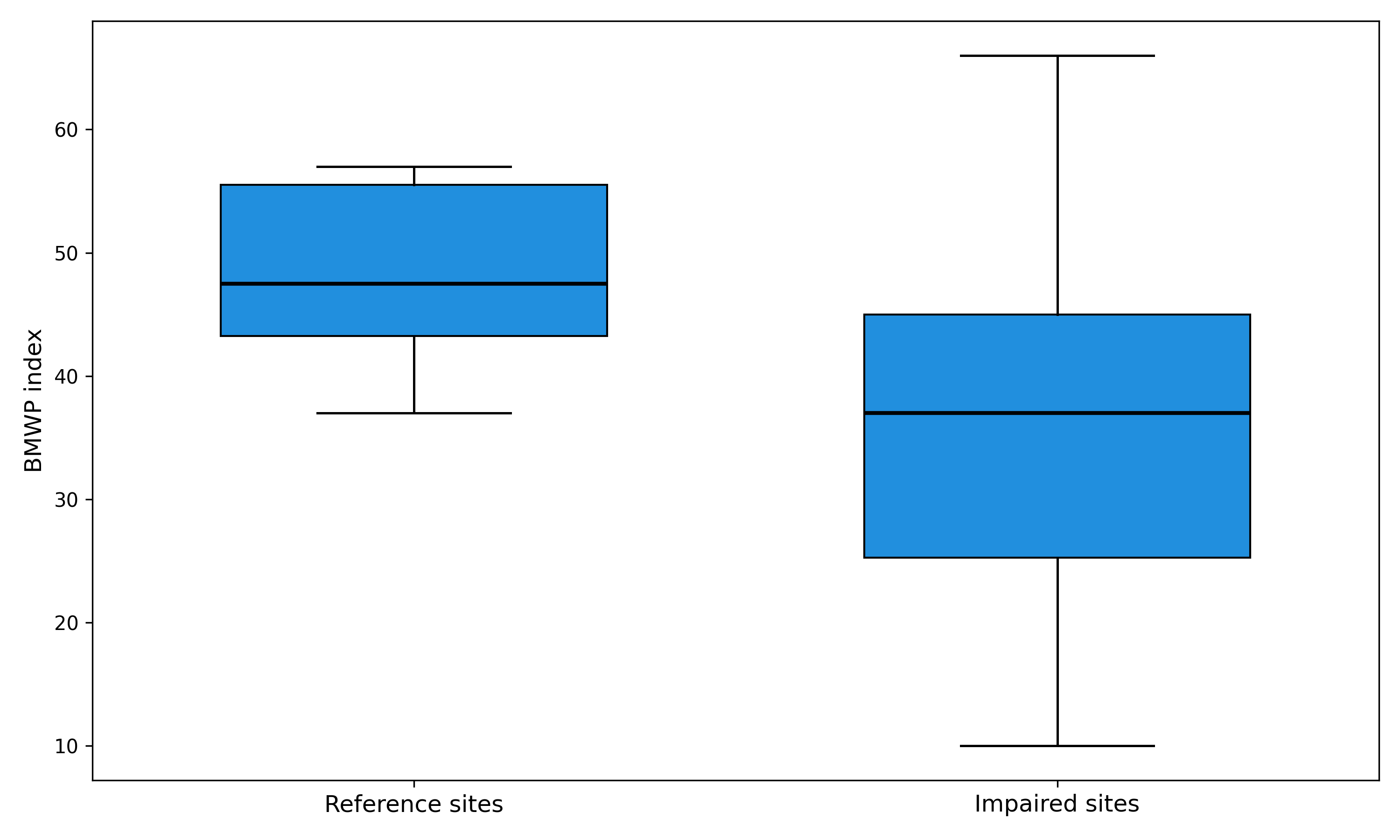

Supplement: Supplementary file 1 [file biology-15-00765-s001.zip › Supplementary/File S7/macroinvertebrates_2021_05/M38_BMWP index.png]

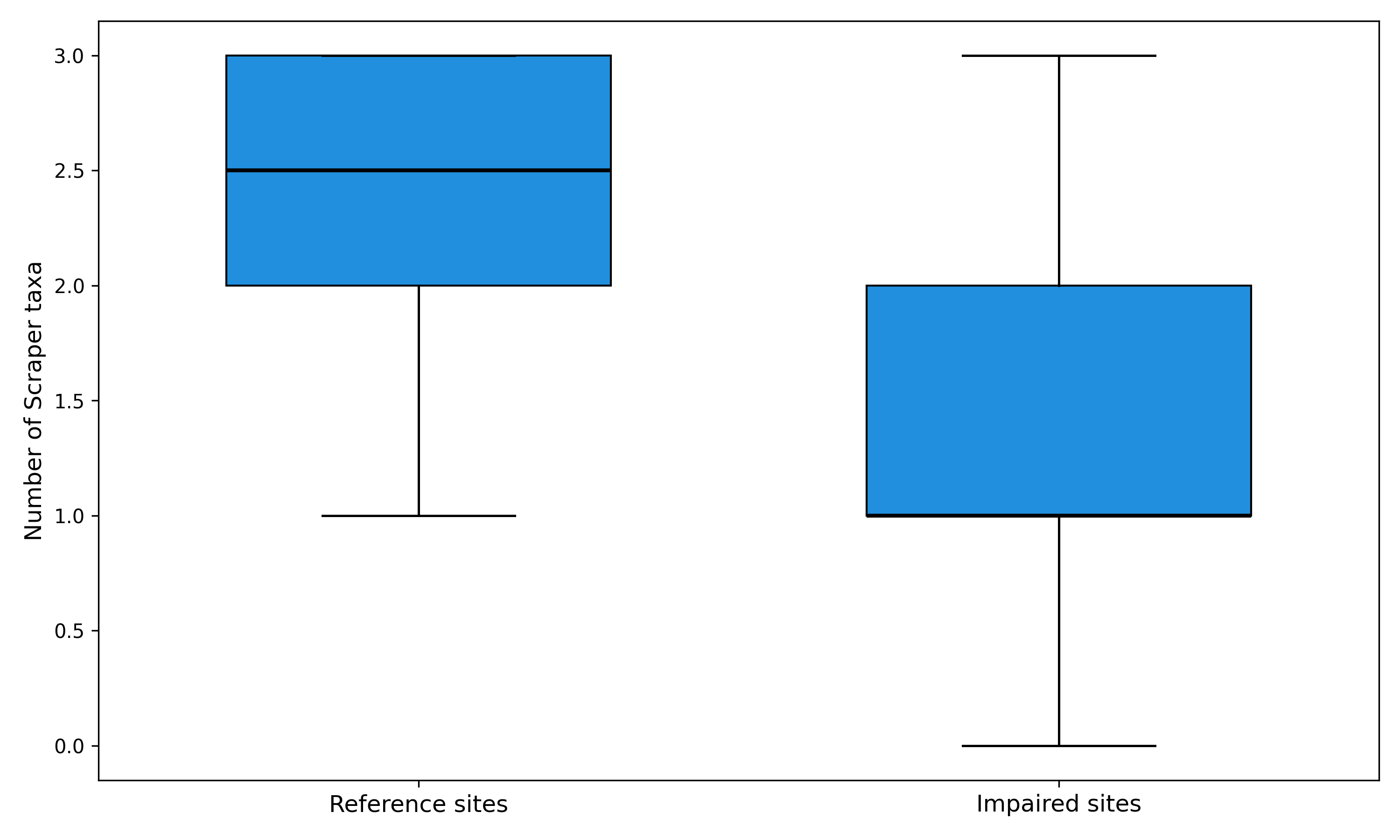

Supplement: Supplementary file 1 [file biology-15-00765-s001.zip › Supplementary/File S7/macroinvertebrates_2021_05/M46_Number of Scraper taxa.png]

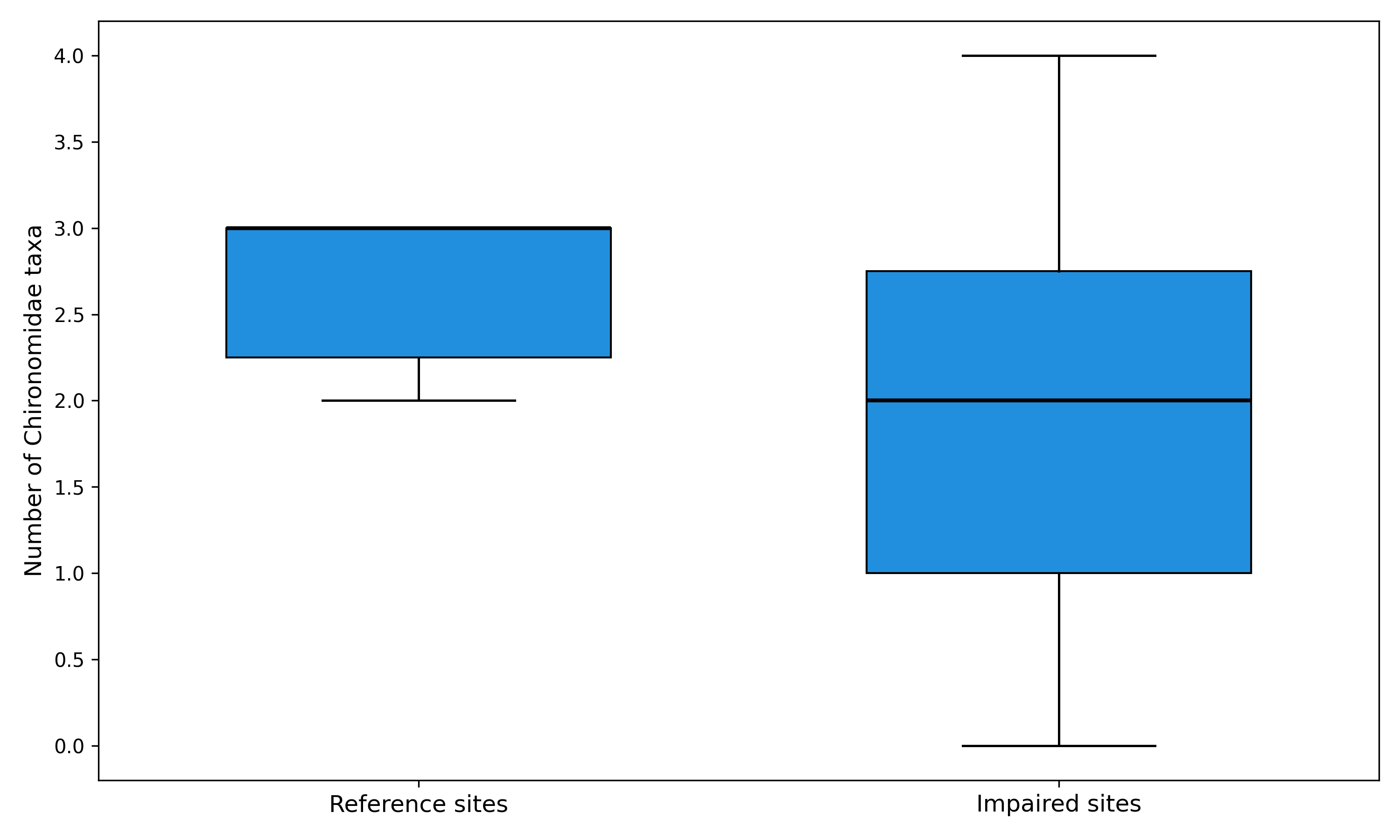

Supplement: Supplementary file 1 [file biology-15-00765-s001.zip › Supplementary/File S7/macroinvertebrates_2021_05/M4_Number of Chironomidae taxa.png]

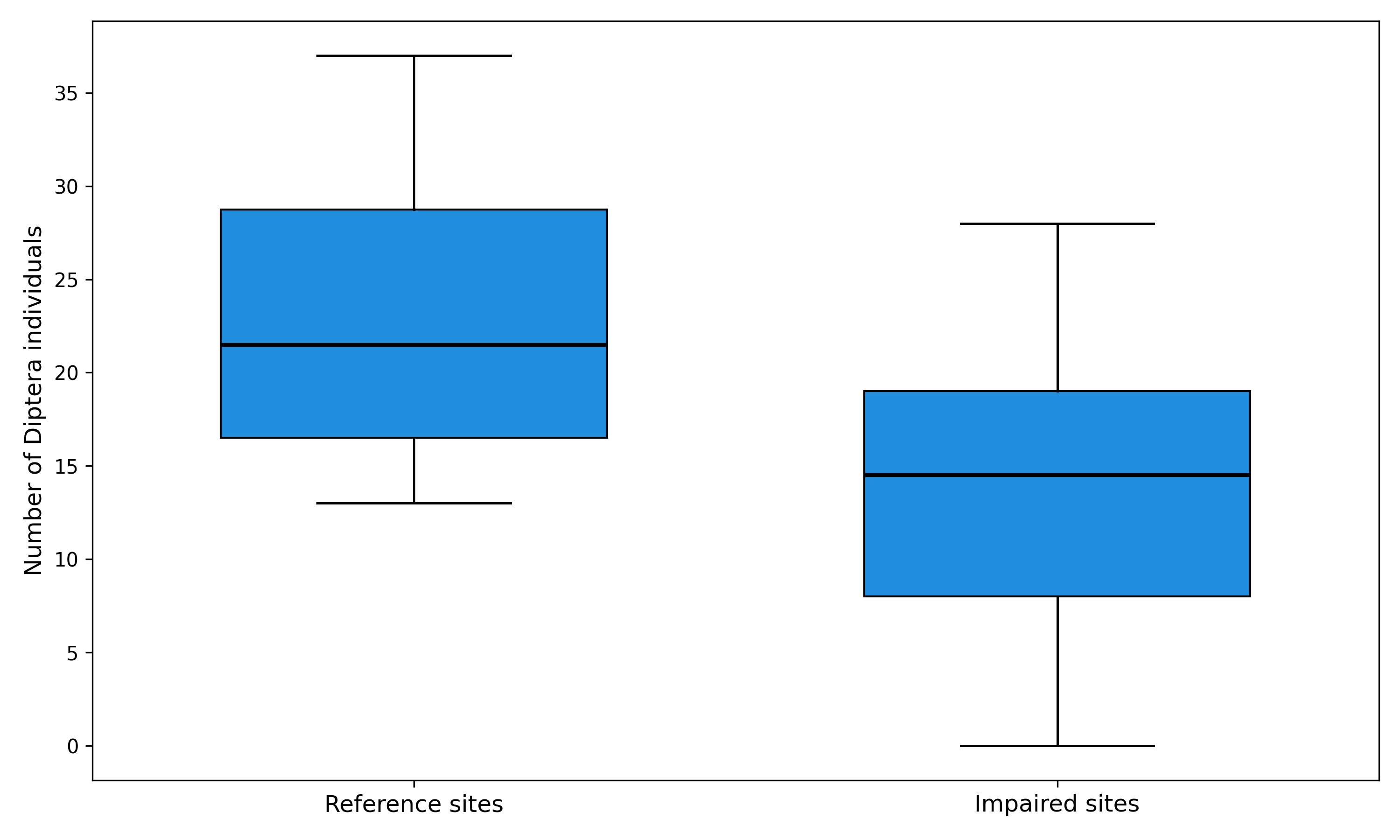

Supplement: Supplementary file 1 [file biology-15-00765-s001.zip › Supplementary/File S7/macroinvertebrates_2021_09/M15_Number of Diptera individuals.png]

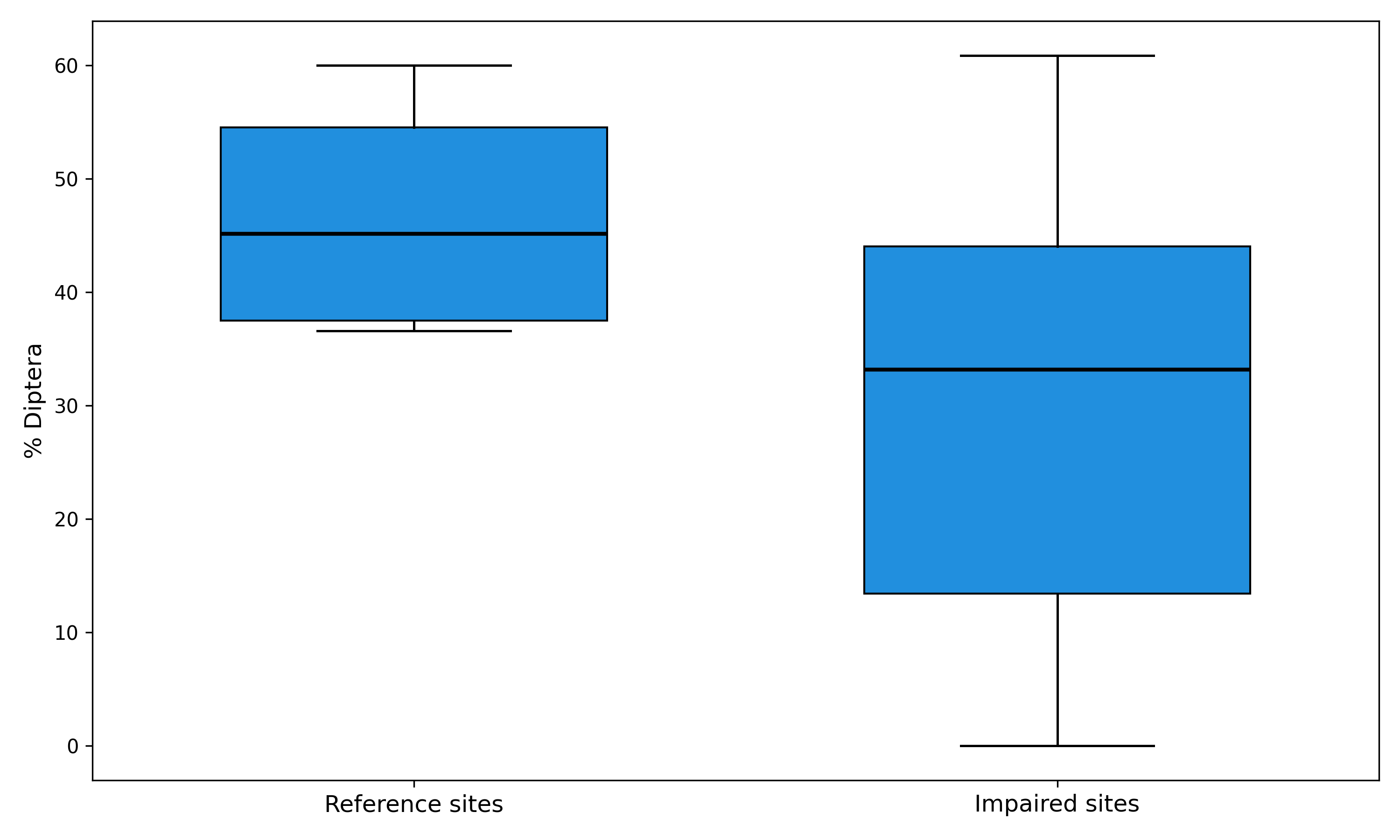

Supplement: Supplementary file 1 [file biology-15-00765-s001.zip › Supplementary/File S7/macroinvertebrates_2021_09/M19_% Diptera.png]

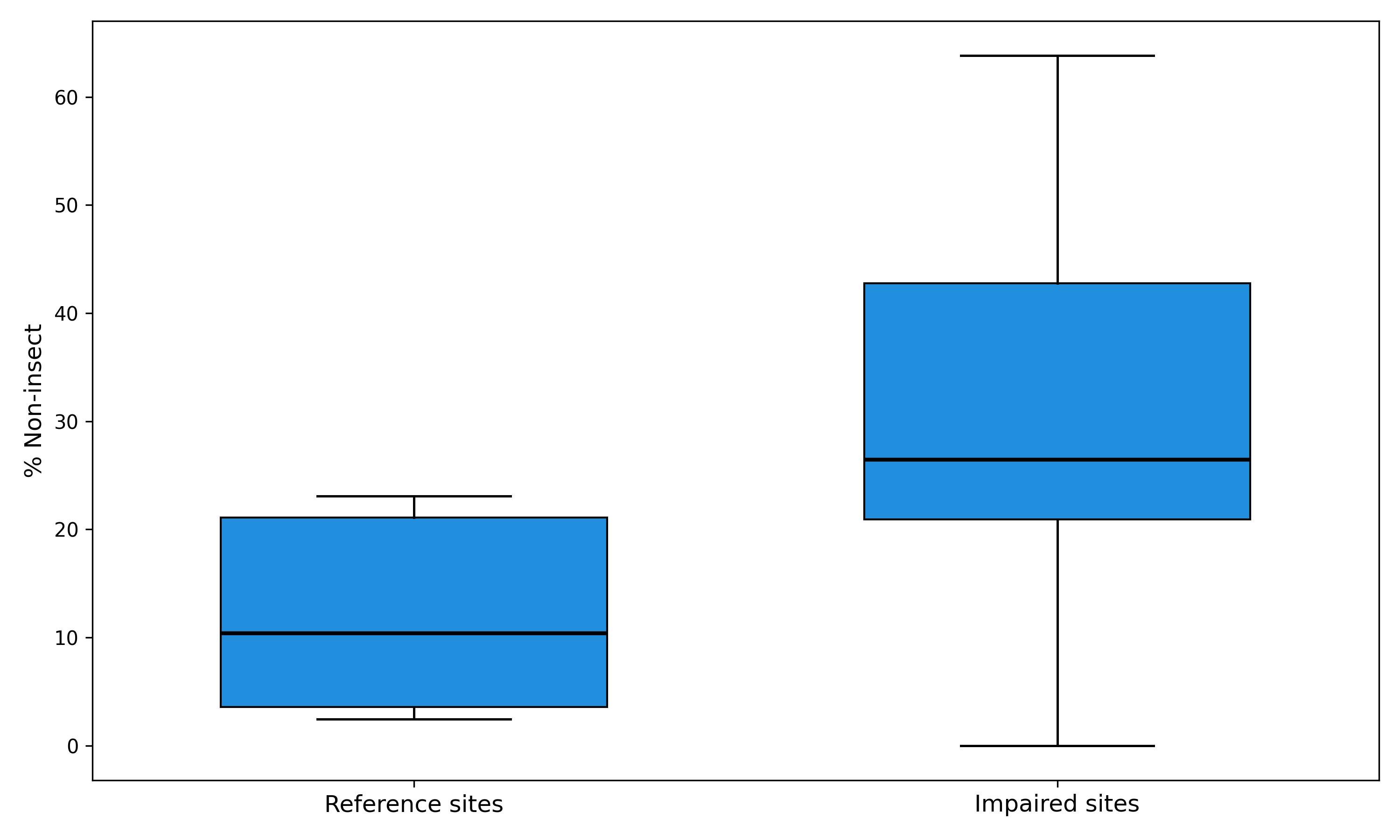

Supplement: Supplementary file 1 [file biology-15-00765-s001.zip › Supplementary/File S7/macroinvertebrates_2021_09/M23_% Non-insect.png]

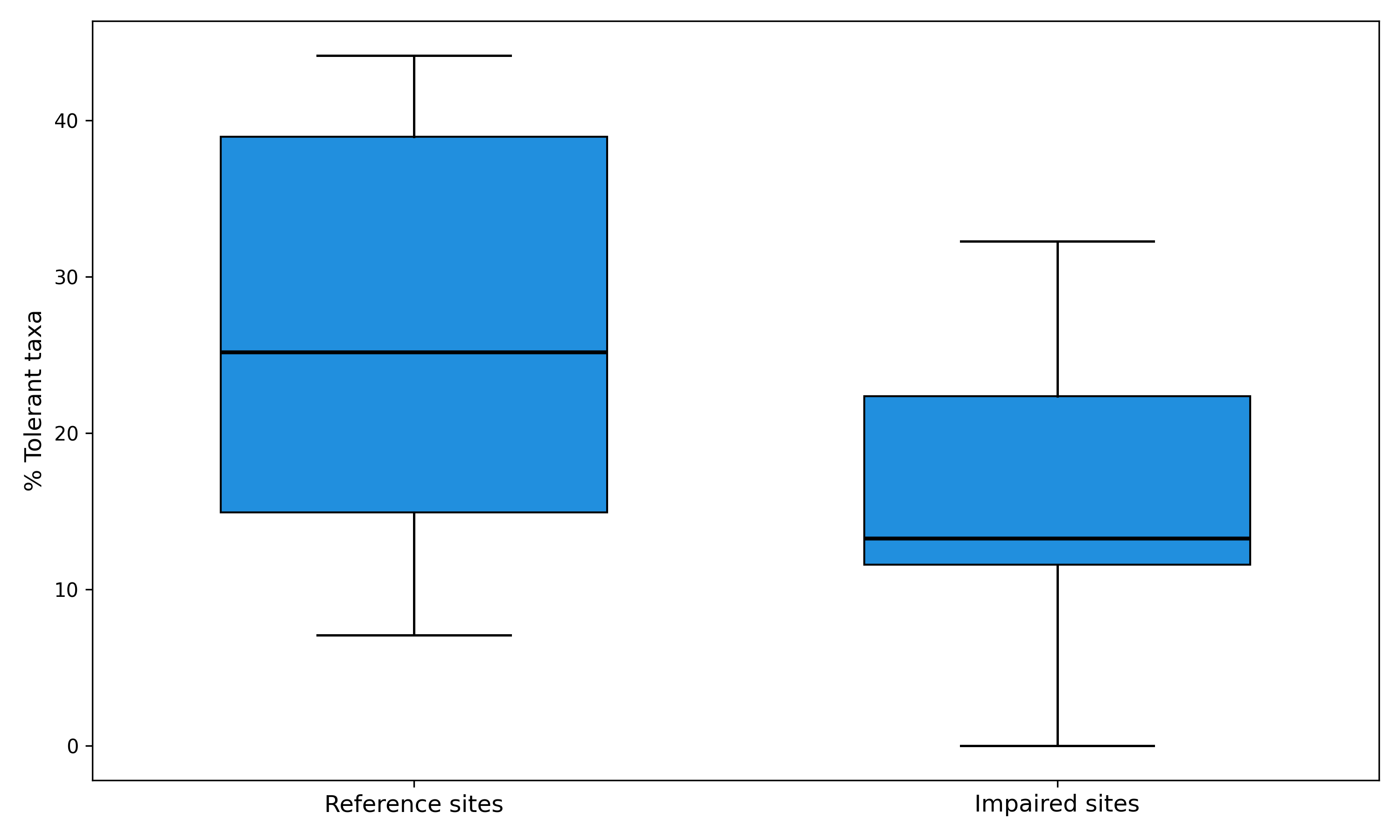

Supplement: Supplementary file 1 [file biology-15-00765-s001.zip › Supplementary/File S7/macroinvertebrates_2021_09/M36_% Tolerant taxa.png]

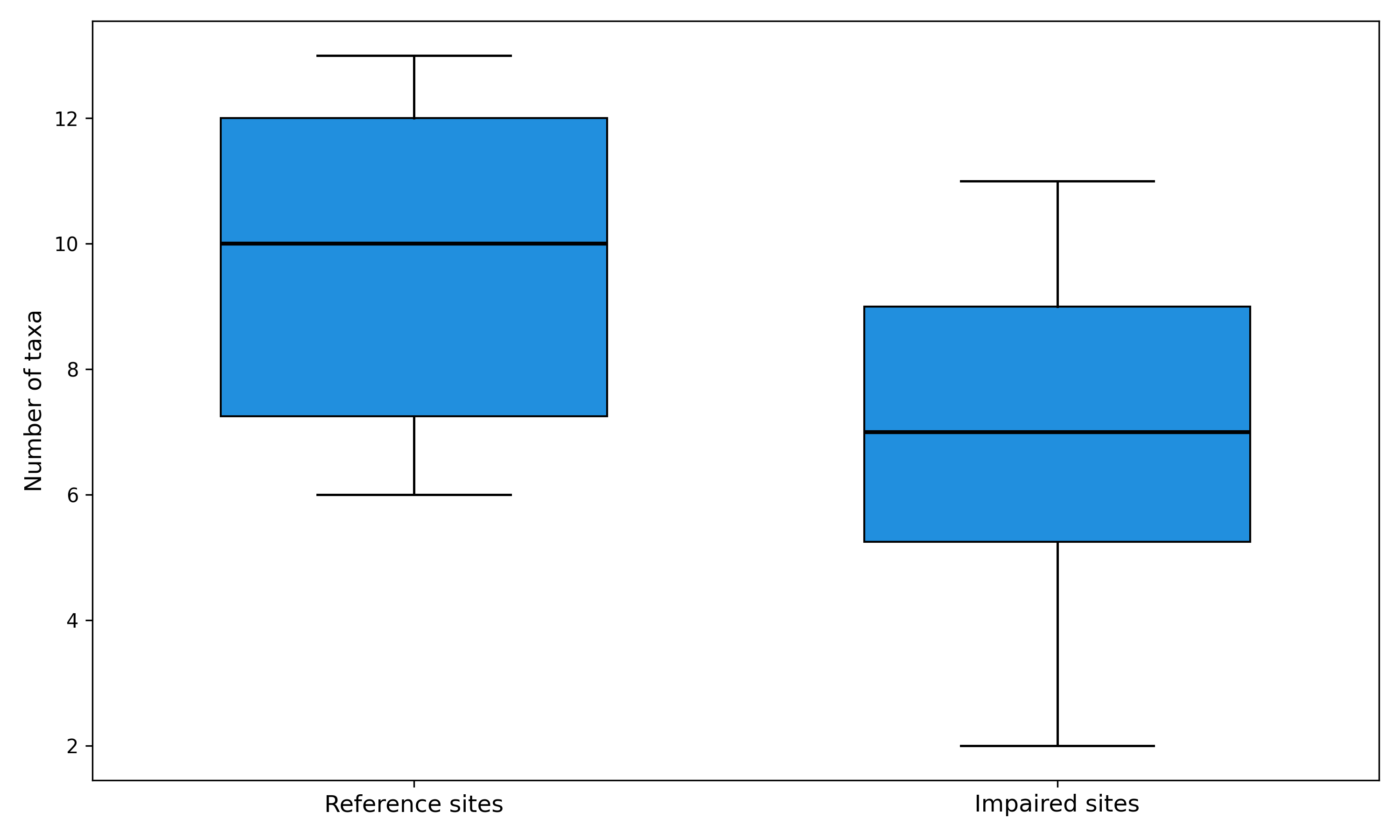

Supplement: Supplementary file 1 [file biology-15-00765-s001.zip › Supplementary/File S7/macroinvertebrates_2022_05/M1_Number of taxa.png]

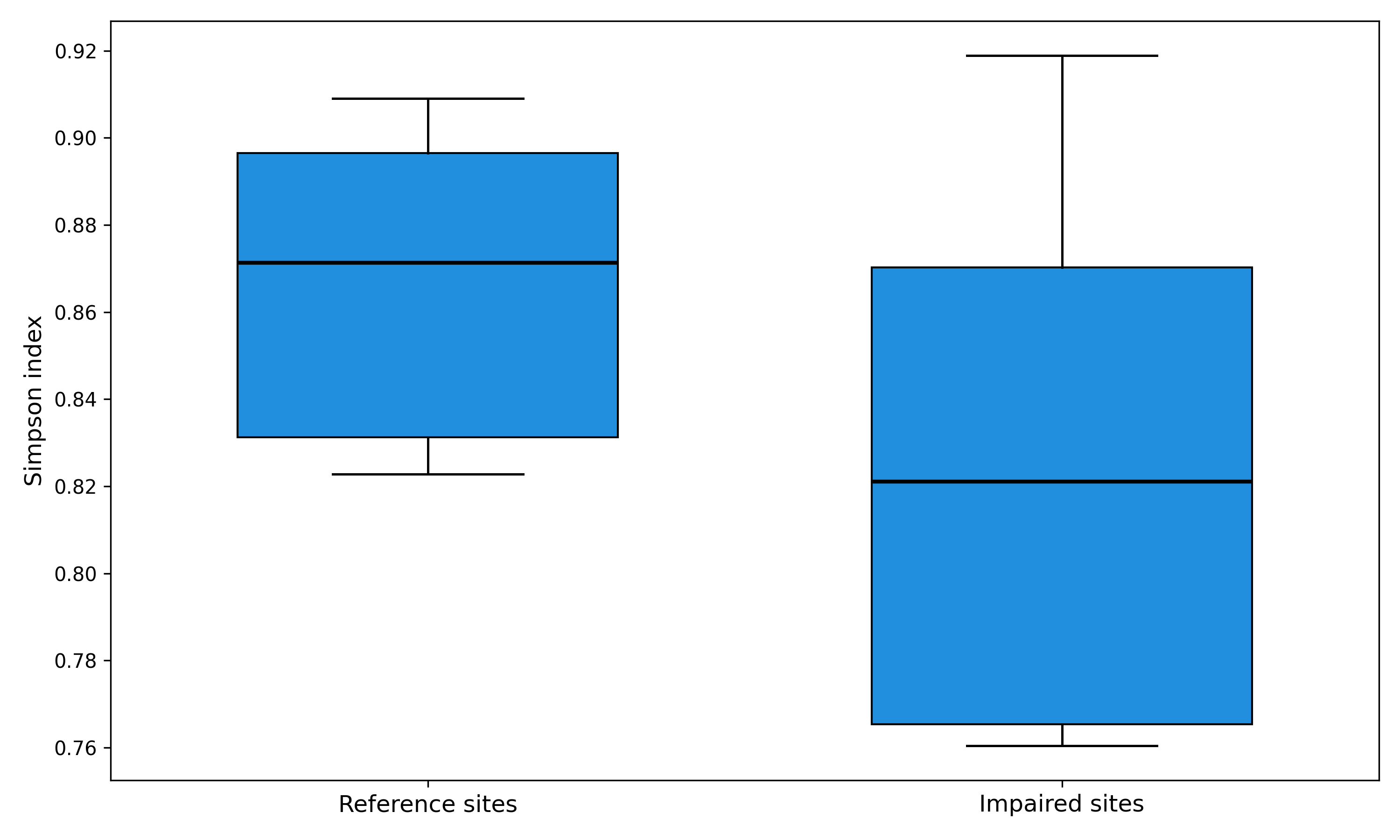

Supplement: Supplementary file 1 [file biology-15-00765-s001.zip › Supplementary/File S7/macroinvertebrates_2022_05/M27_Simpson index.png]

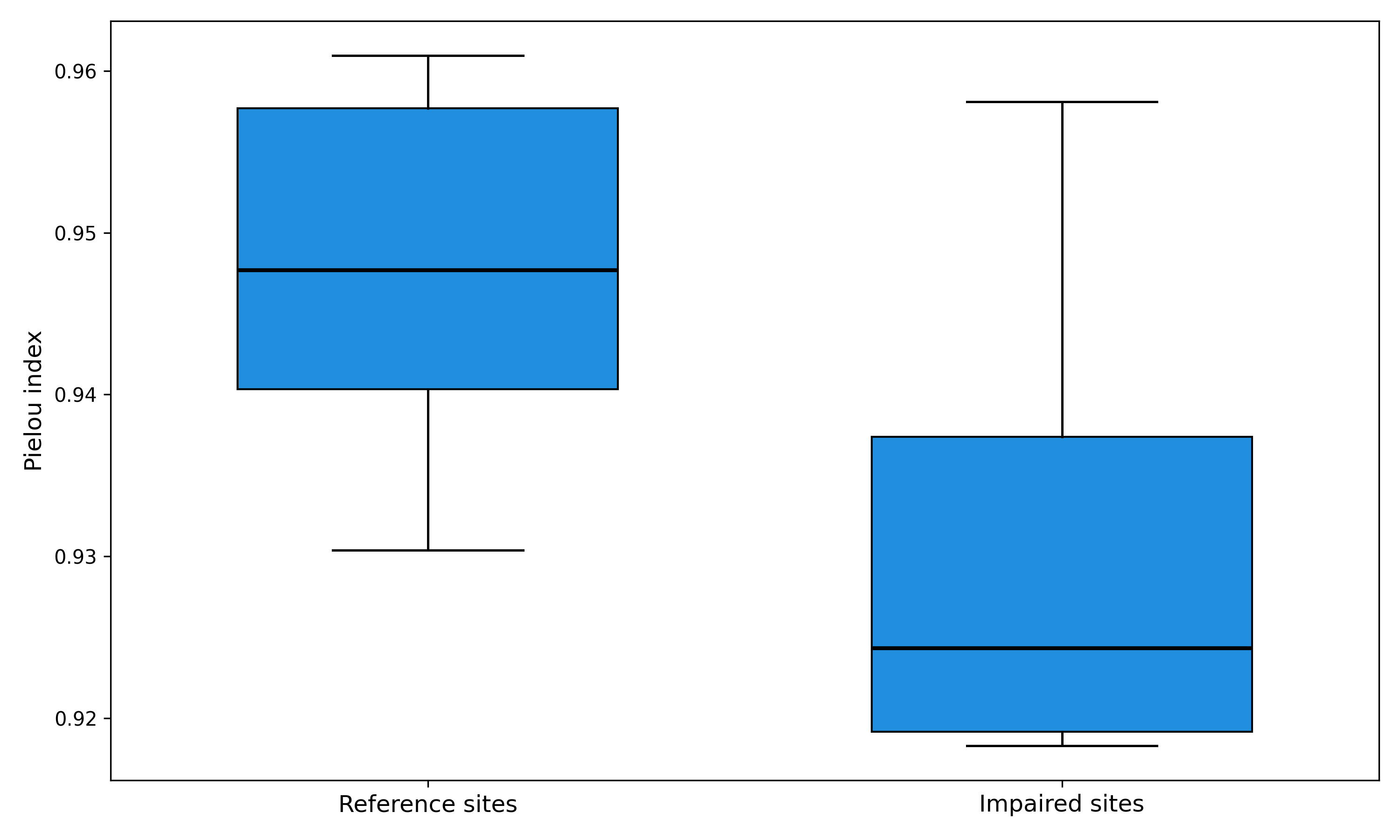

Supplement: Supplementary file 1 [file biology-15-00765-s001.zip › Supplementary/File S7/macroinvertebrates_2022_05/M28_Pielou index.png]

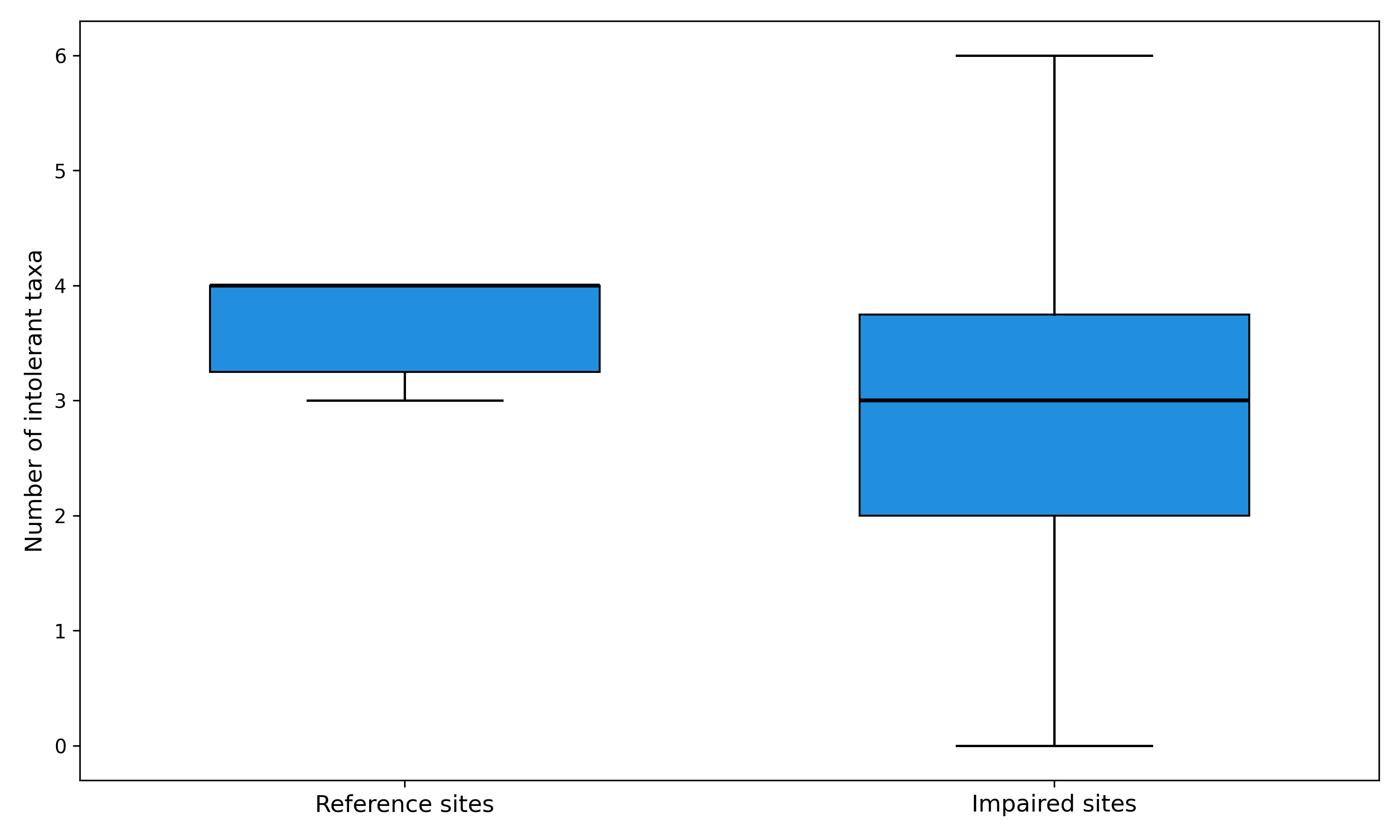

Supplement: Supplementary file 1 [file biology-15-00765-s001.zip › Supplementary/File S7/macroinvertebrates_2022_05/M29_Number of intolerant taxa.png]

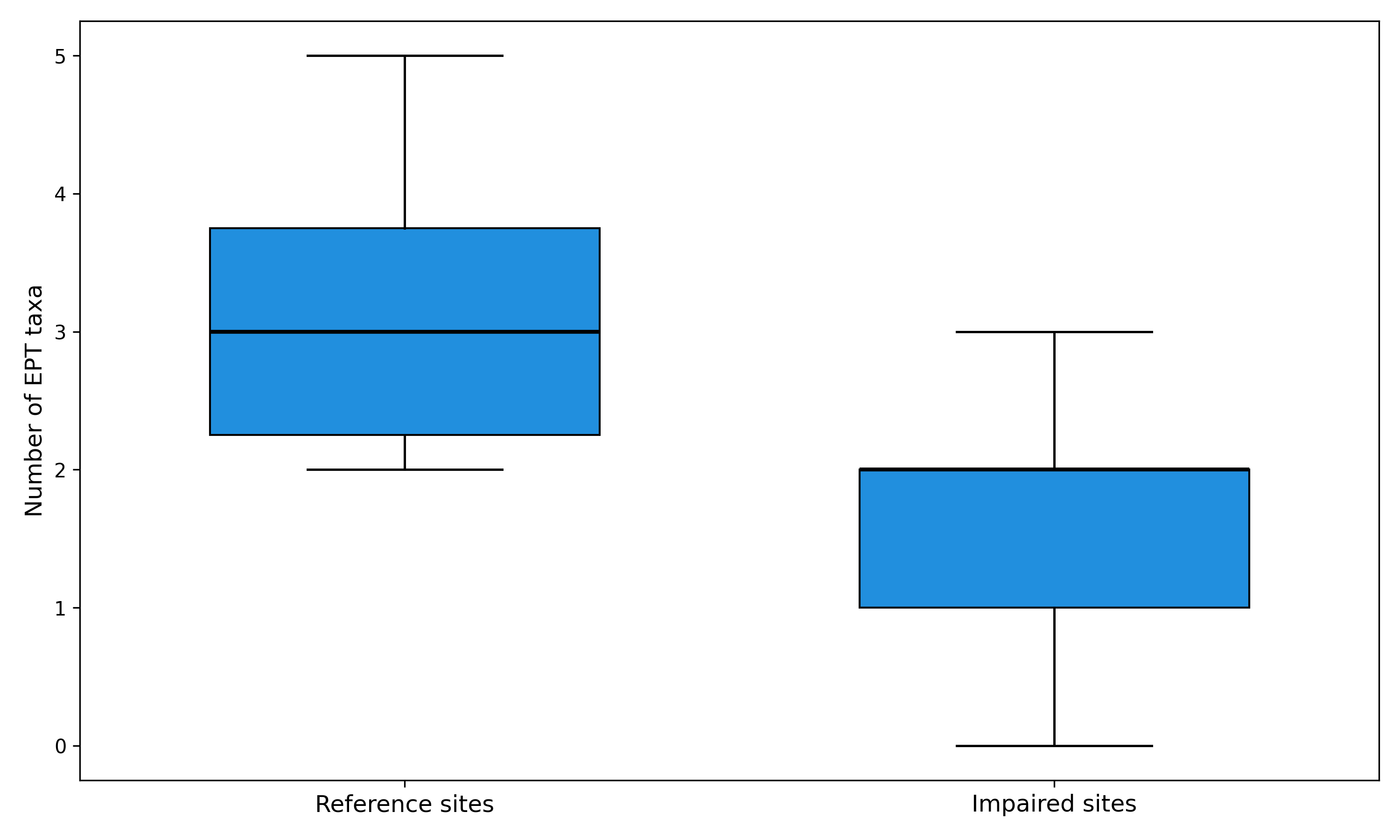

Supplement: Supplementary file 1 [file biology-15-00765-s001.zip › Supplementary/File S7/macroinvertebrates_2022_05/M3_Number of EPT taxa.png]

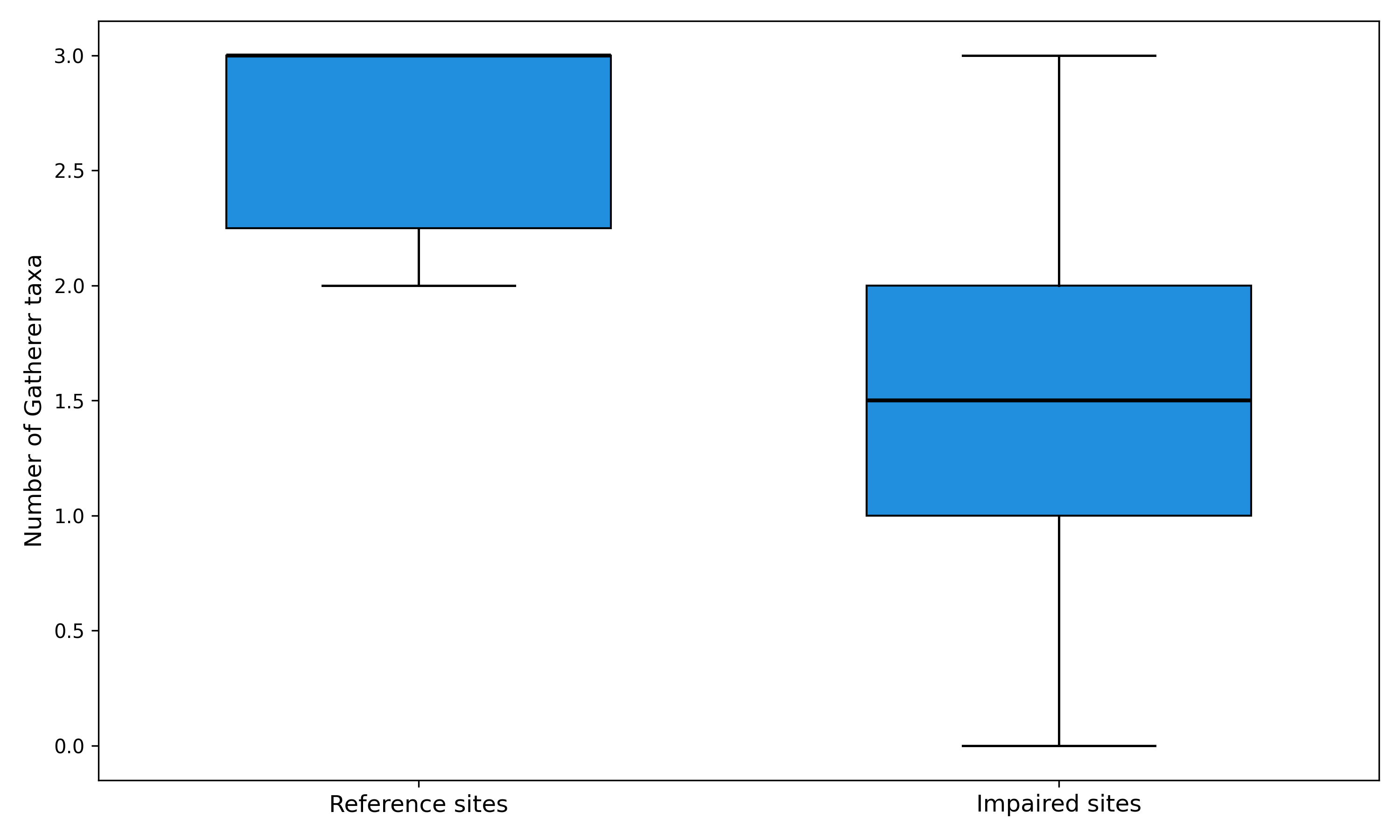

Supplement: Supplementary file 1 [file biology-15-00765-s001.zip › Supplementary/File S7/macroinvertebrates_2022_05/M48_Number of Gatherer taxa.png]

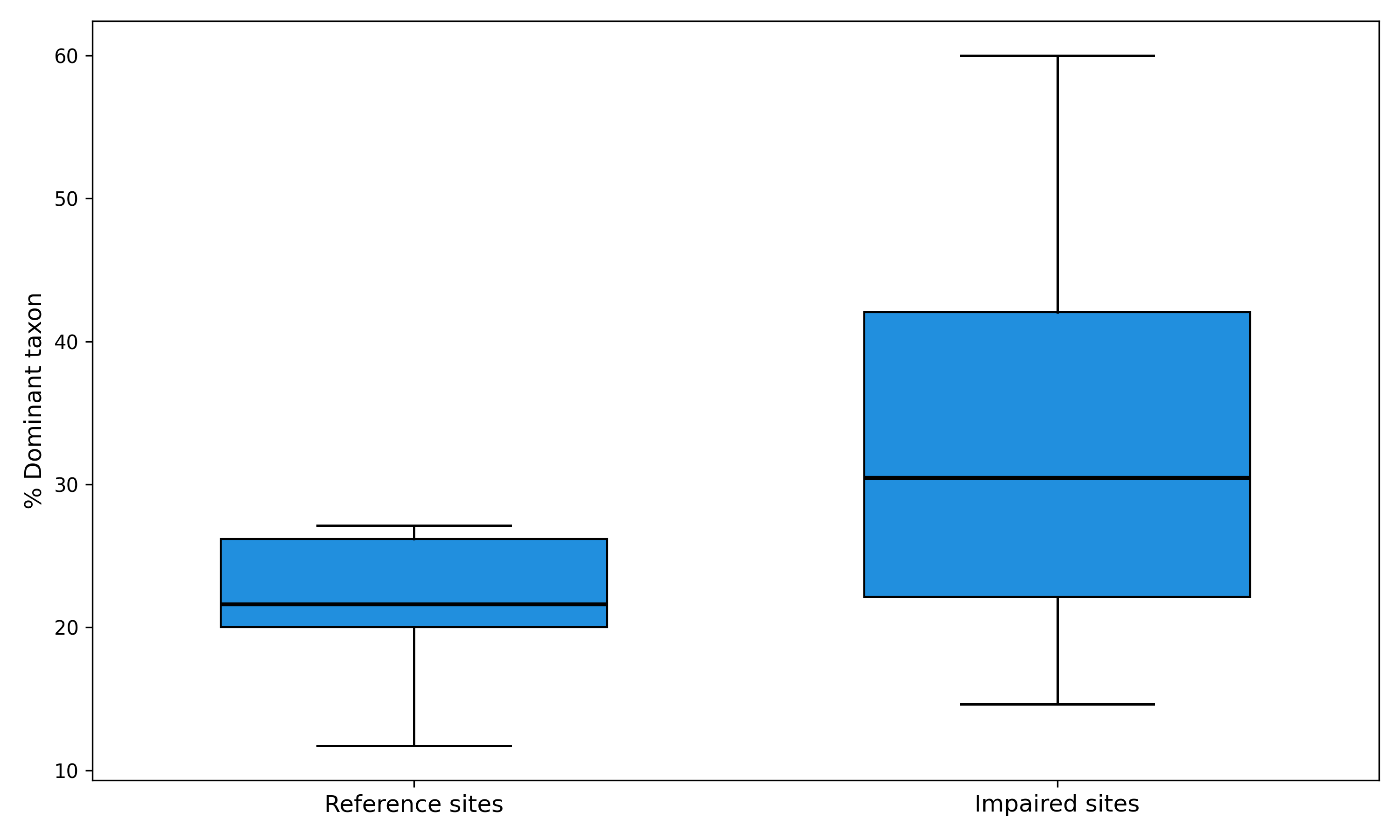

Supplement: Supplementary file 1 [file biology-15-00765-s001.zip › Supplementary/File S7/macroinvertebrates_2022_09/M16_% Dominant taxon.png]

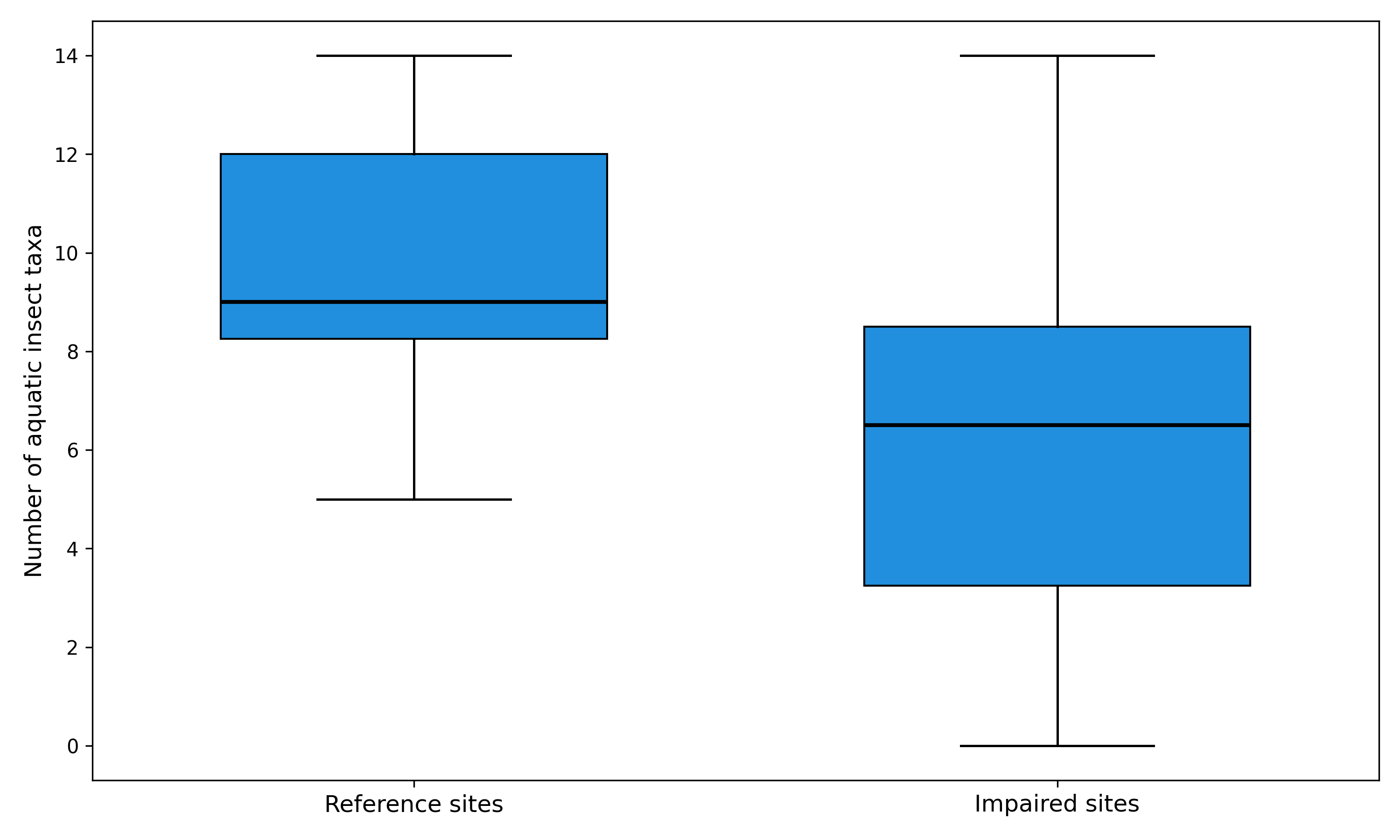

Supplement: Supplementary file 1 [file biology-15-00765-s001.zip › Supplementary/File S7/macroinvertebrates_2022_09/M2_Number of aquatic insect taxa.png]

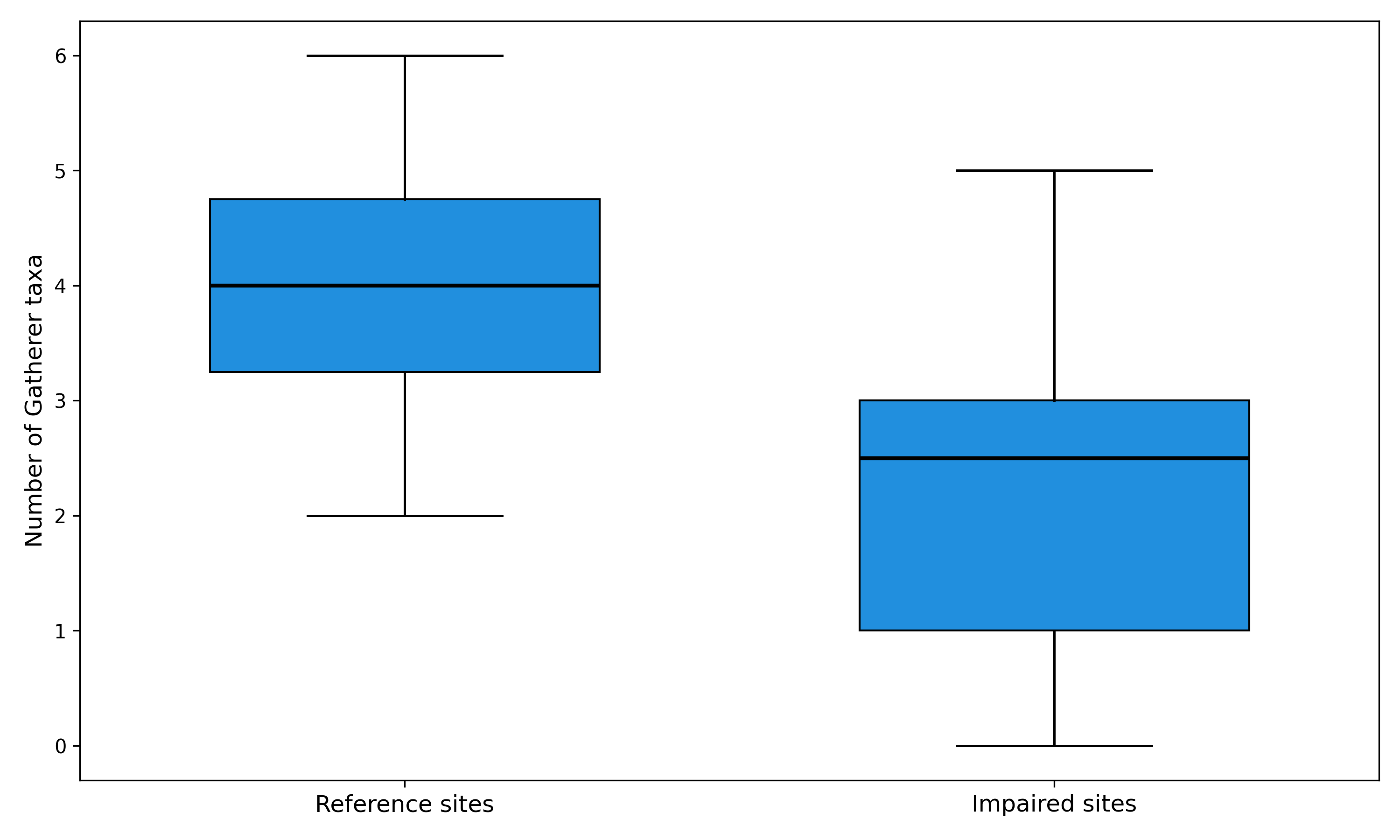

Supplement: Supplementary file 1 [file biology-15-00765-s001.zip › Supplementary/File S7/macroinvertebrates_2022_09/M48_Number of Gatherer taxa.png]

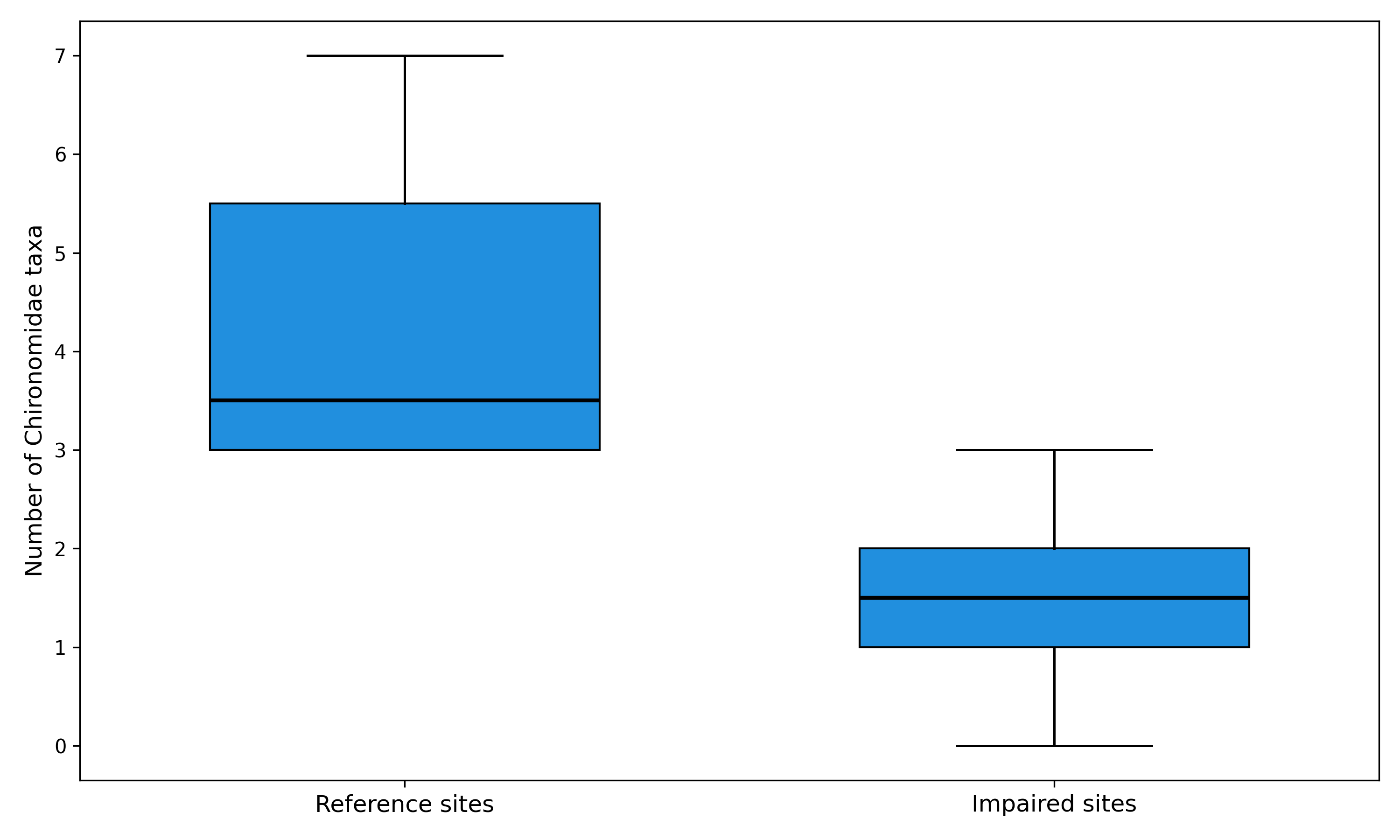

Supplement: Supplementary file 1 [file biology-15-00765-s001.zip › Supplementary/File S7/macroinvertebrates_2022_09/M4_Number of Chironomidae taxa.png]

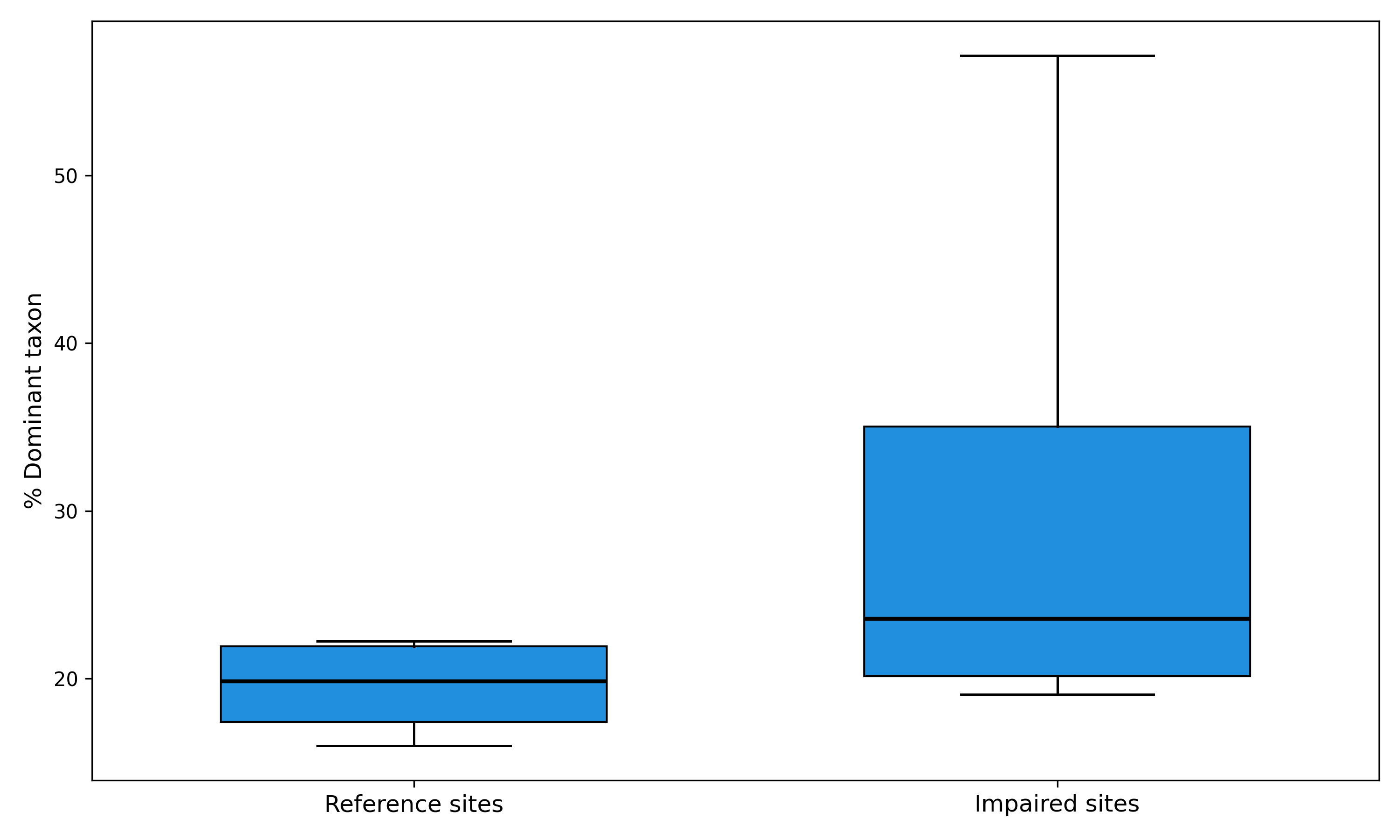

Supplement: Supplementary file 1 [file biology-15-00765-s001.zip › Supplementary/File S7/macroinvertebrates_2023_05/M16_% Dominant taxon.png]

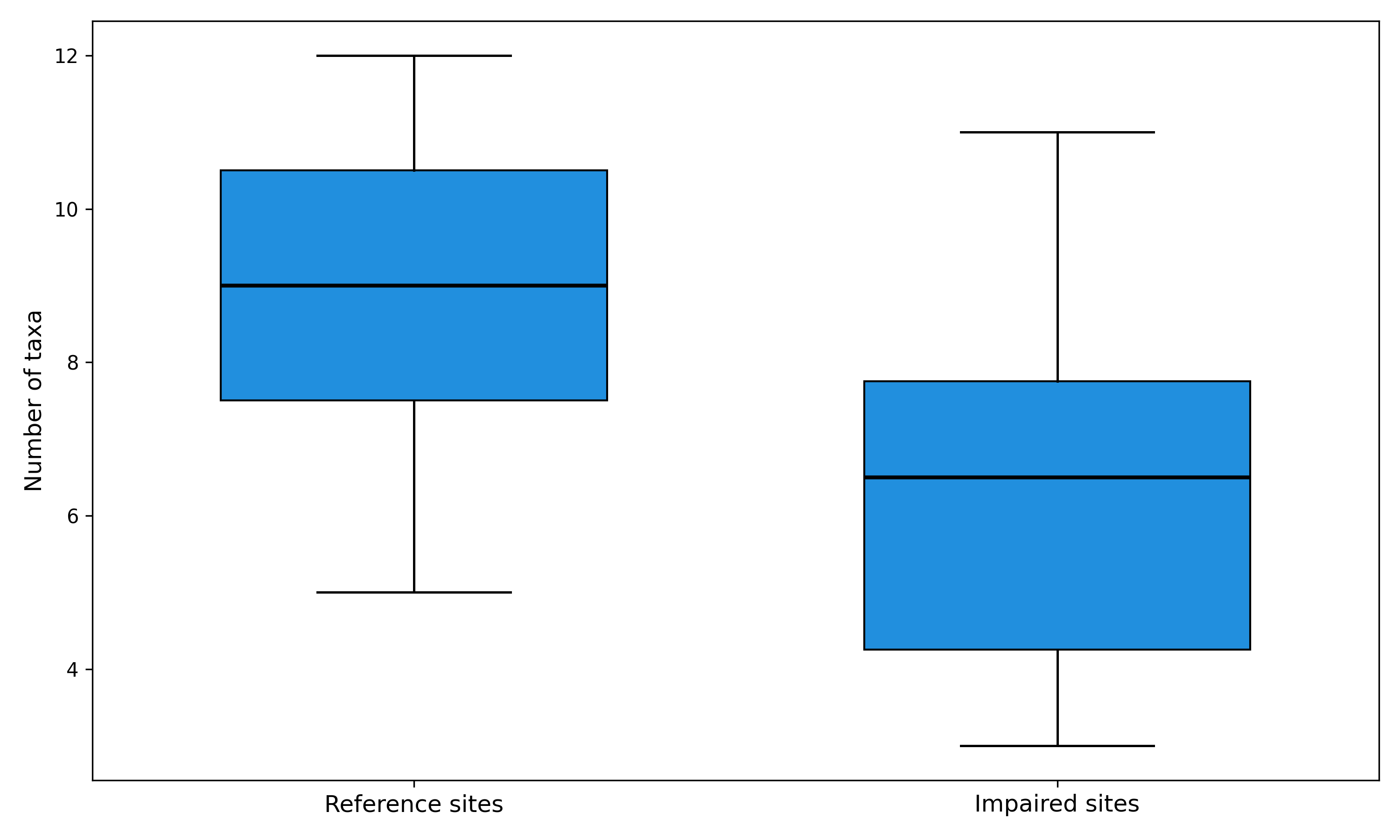

Supplement: Supplementary file 1 [file biology-15-00765-s001.zip › Supplementary/File S7/macroinvertebrates_2023_05/M1_Number of taxa.png]

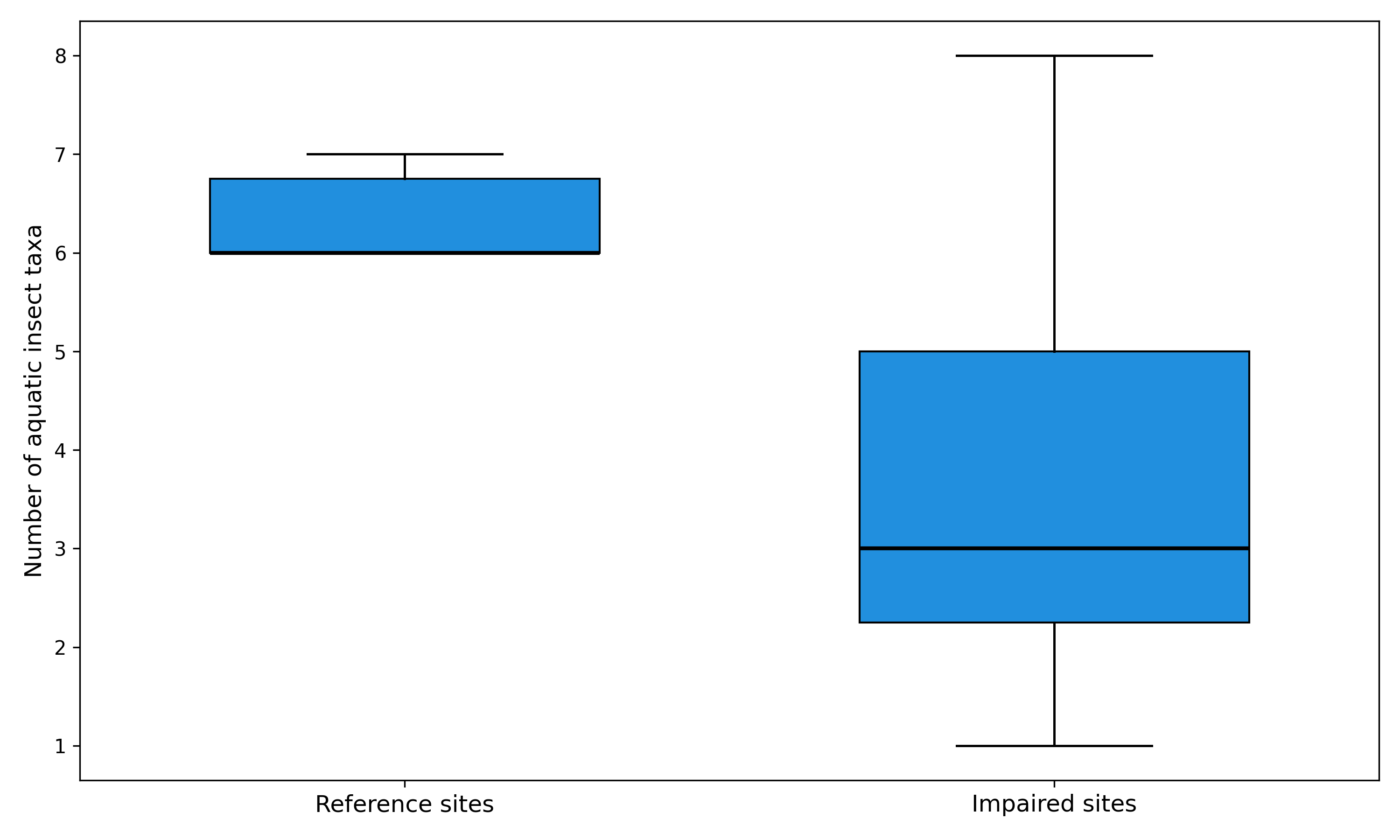

Supplement: Supplementary file 1 [file biology-15-00765-s001.zip › Supplementary/File S7/macroinvertebrates_2023_05/M2_Number of aquatic insect taxa.png]

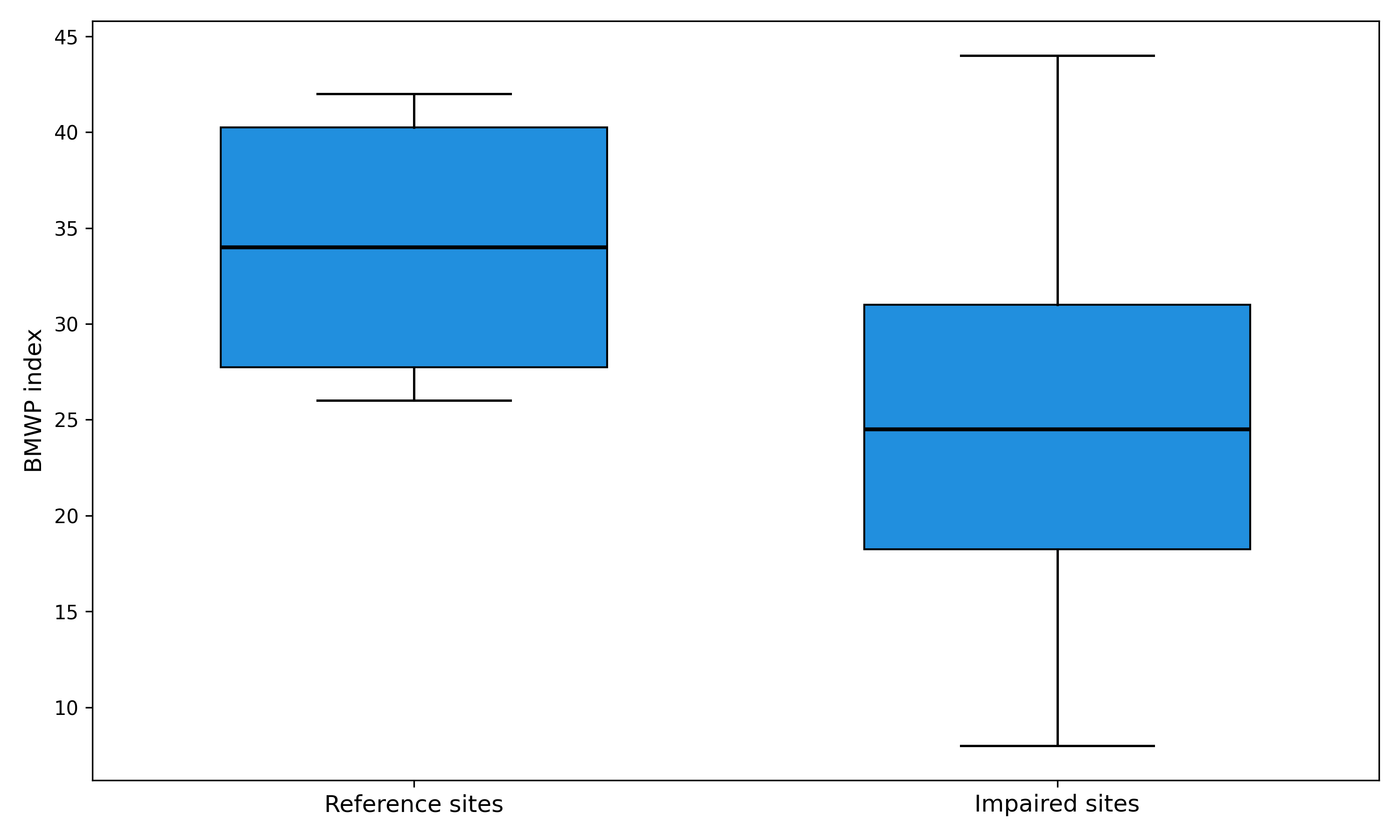

Supplement: Supplementary file 1 [file biology-15-00765-s001.zip › Supplementary/File S7/macroinvertebrates_2023_05/M38_BMWP index.png]

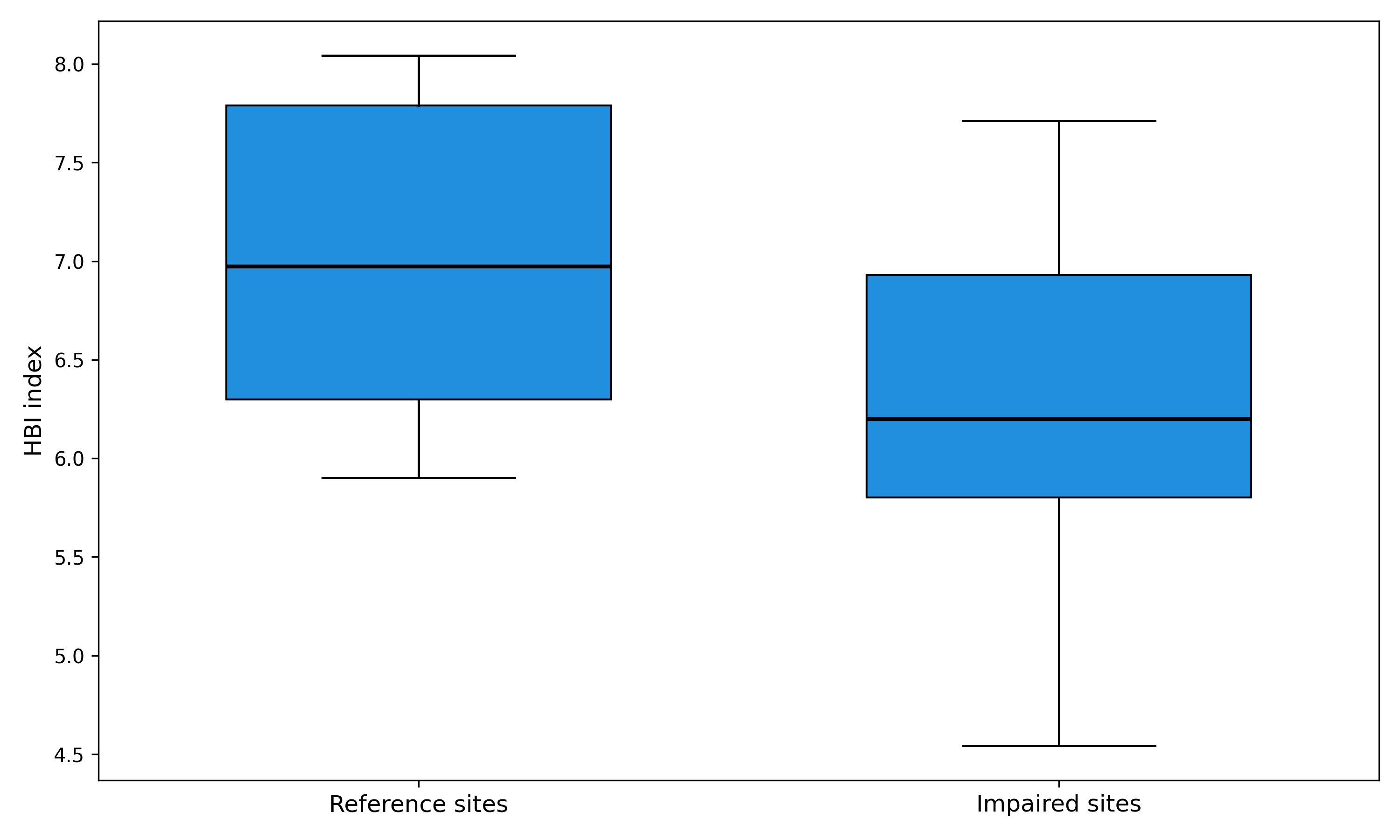

Supplement: Supplementary file 1 [file biology-15-00765-s001.zip › Supplementary/File S7/macroinvertebrates_2023_05/M39_HBI index.png]

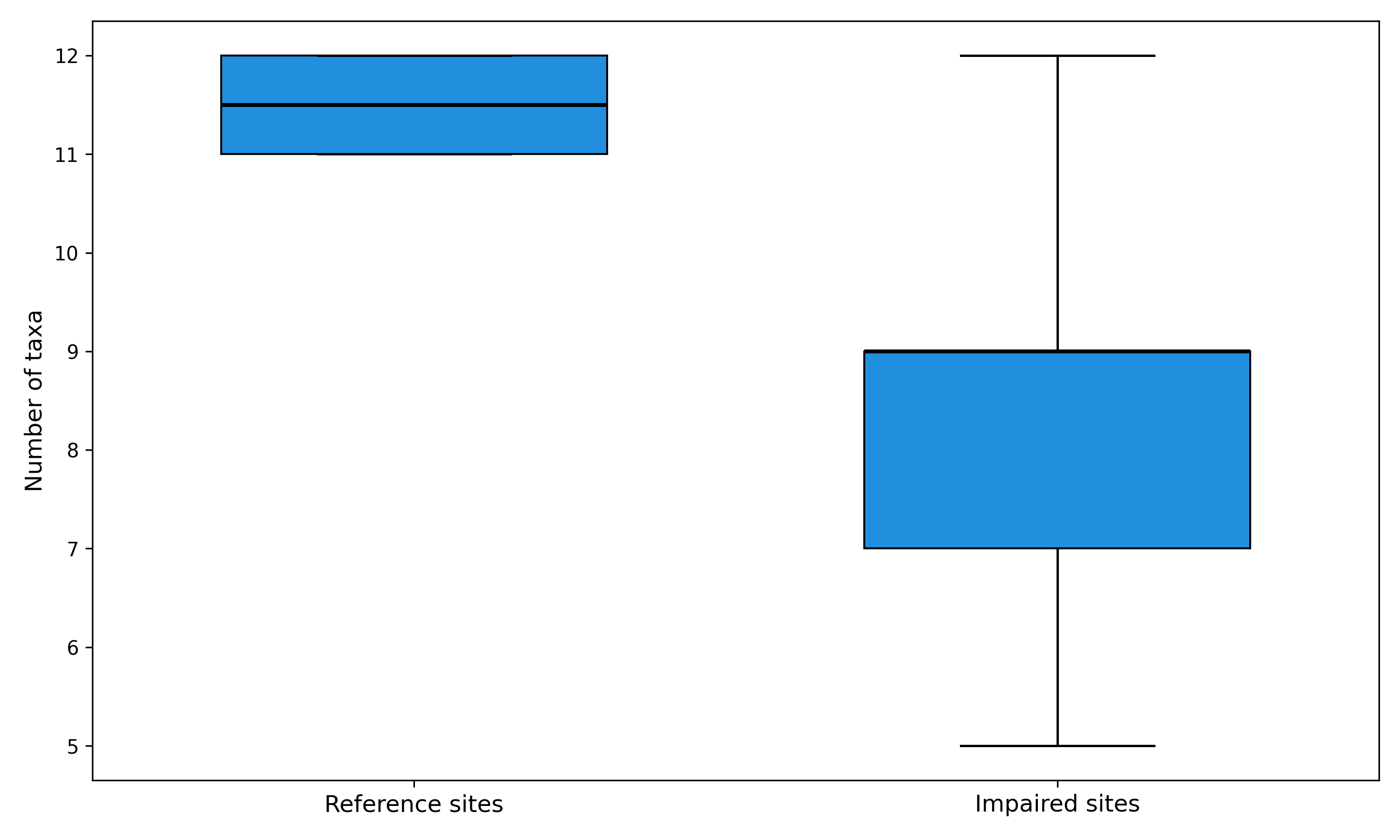

Supplement: Supplementary file 1 [file biology-15-00765-s001.zip › Supplementary/File S7/macroinvertebrates_2023_09/M1_Number of taxa.png]

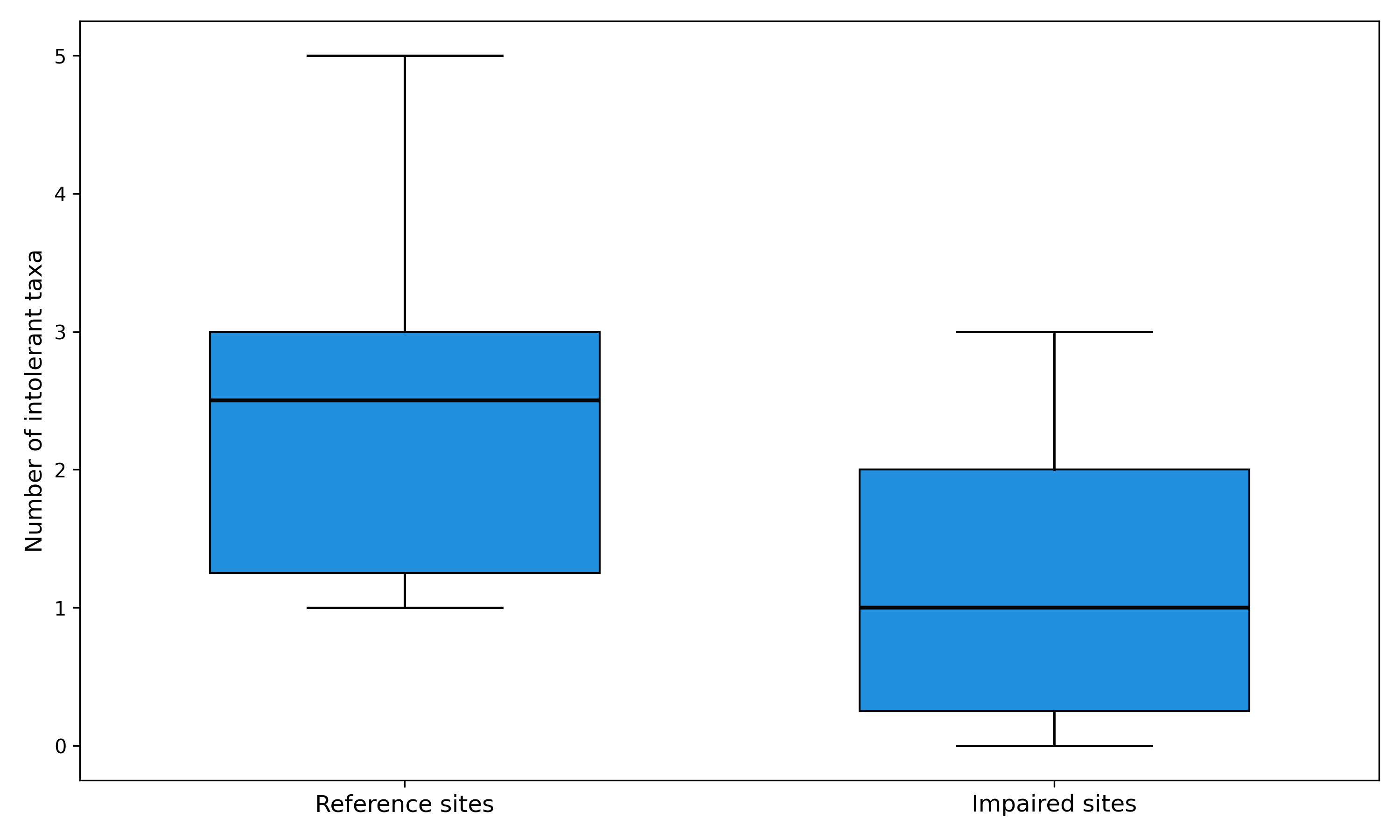

Supplement: Supplementary file 1 [file biology-15-00765-s001.zip › Supplementary/File S7/macroinvertebrates_2023_09/M29_Number of intolerant taxa.png]

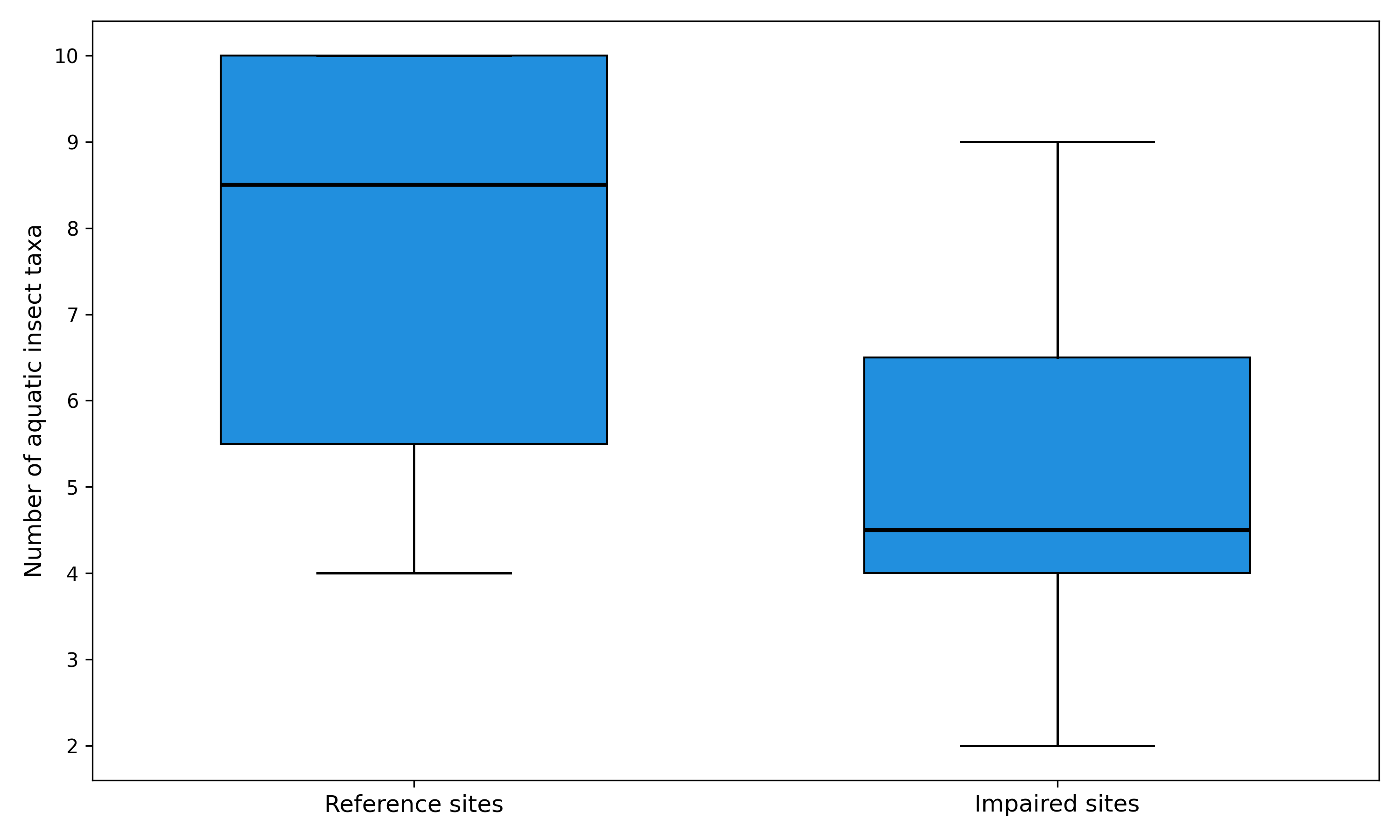

Supplement: Supplementary file 1 [file biology-15-00765-s001.zip › Supplementary/File S7/macroinvertebrates_2023_09/M2_Number of aquatic insect taxa.png]

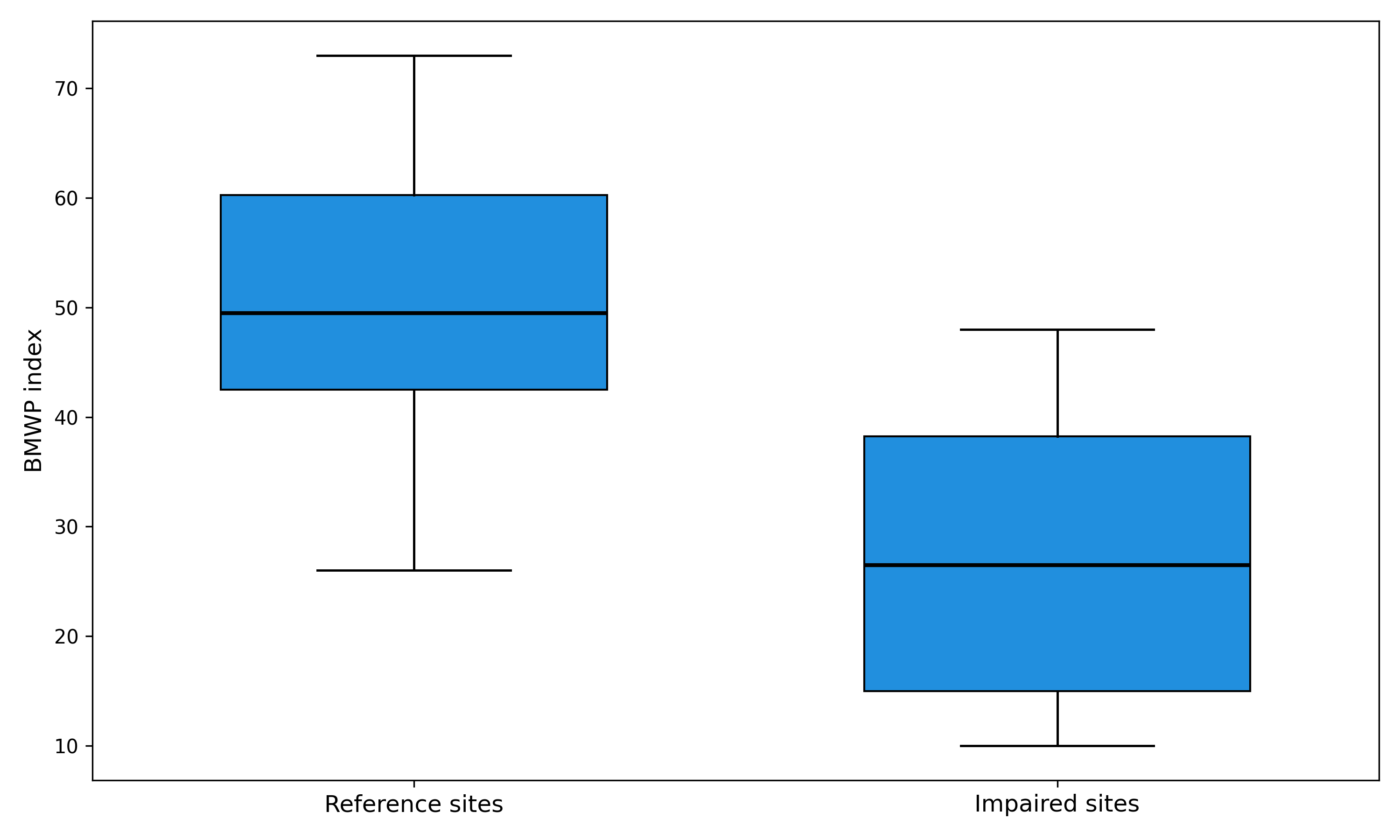

Supplement: Supplementary file 1 [file biology-15-00765-s001.zip › Supplementary/File S7/macroinvertebrates_2023_09/M38_BMWP index.png]

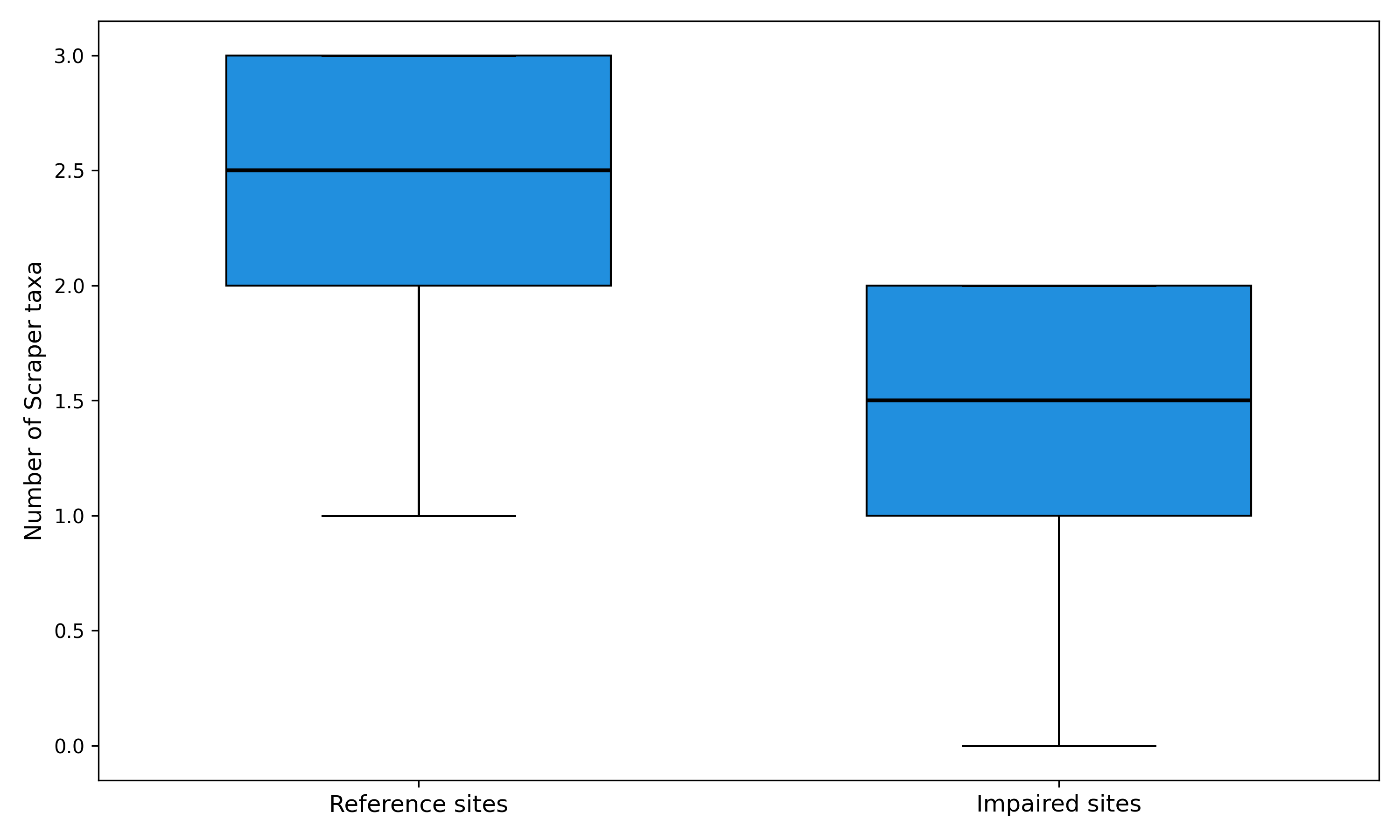

Supplement: Supplementary file 1 [file biology-15-00765-s001.zip › Supplementary/File S7/macroinvertebrates_2023_09/M46_Number of Scraper taxa.png]

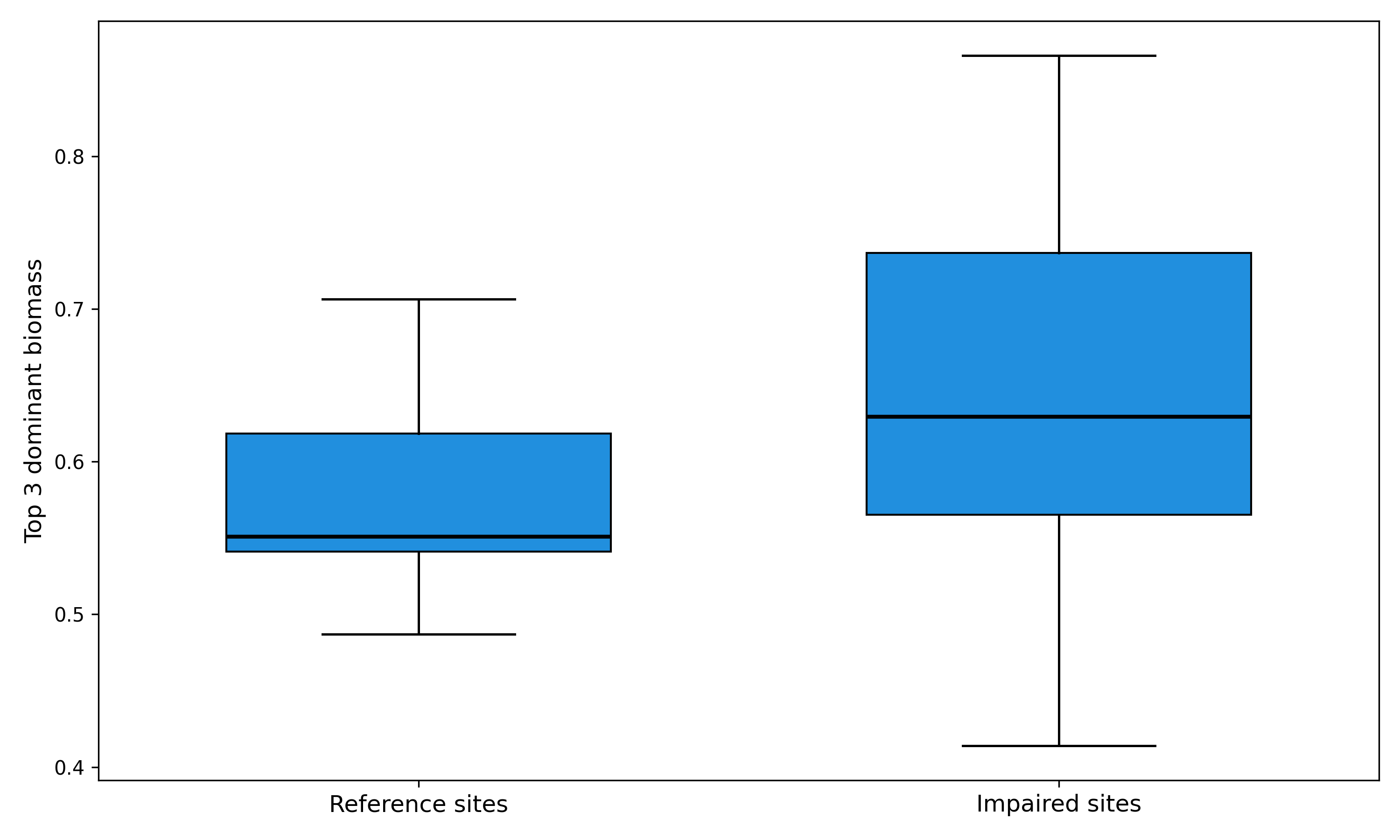

Supplement: Supplementary file 1 [file biology-15-00765-s001.zip › Supplementary/File S7/phytoplankton_2021_05/P14_Top 3 dominant biomass.png]

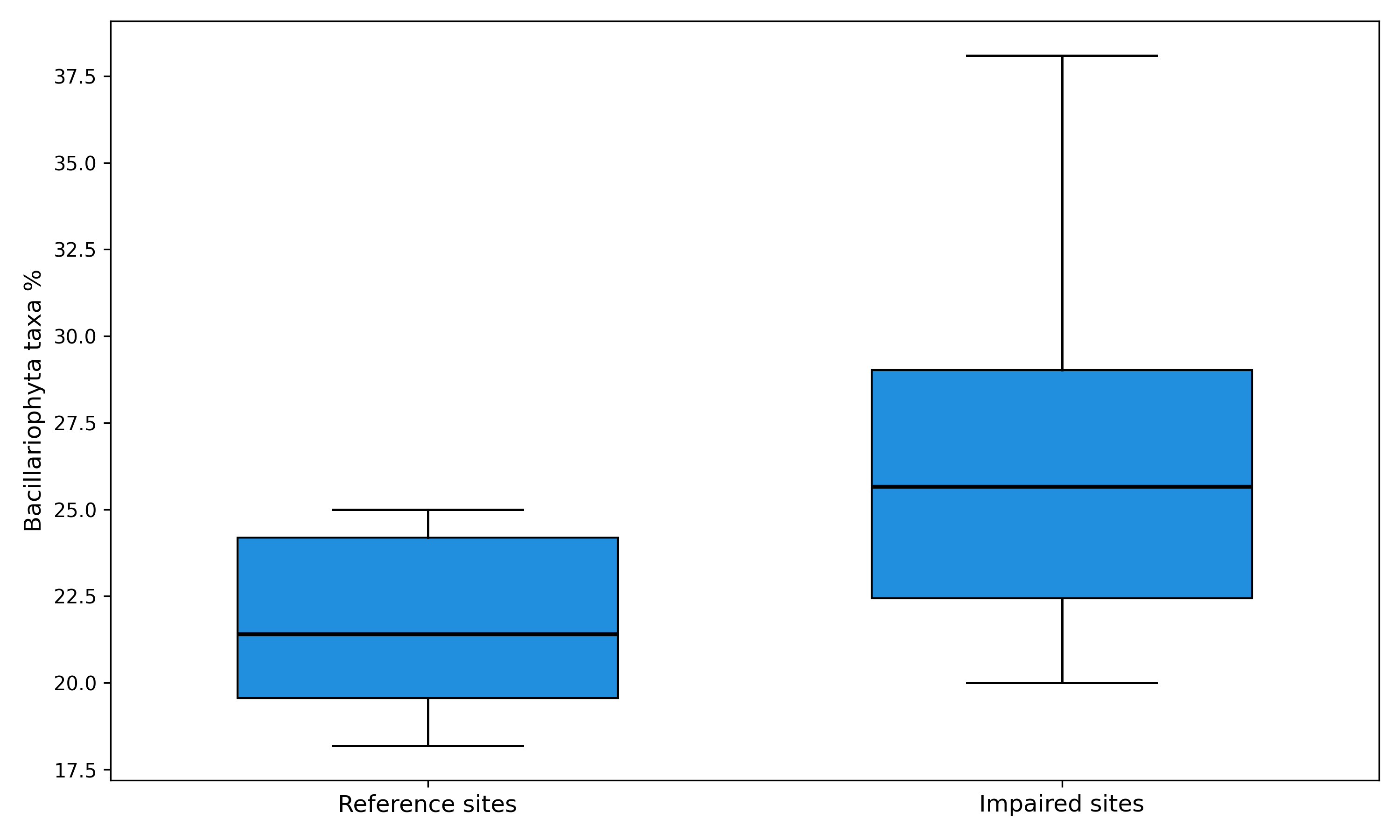

Supplement: Supplementary file 1 [file biology-15-00765-s001.zip › Supplementary/File S7/phytoplankton_2021_05/P15_Bacillariophyta taxa %.png]

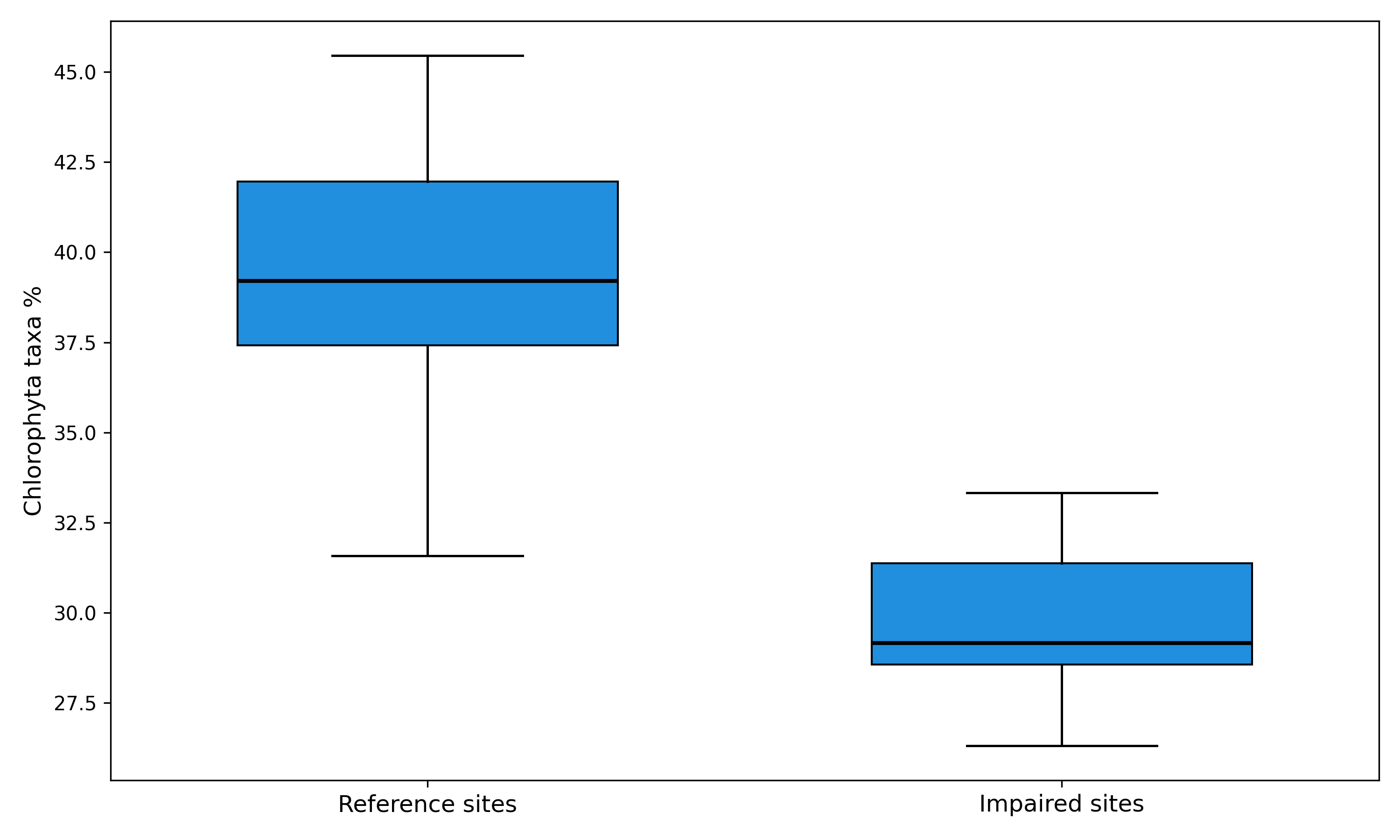

Supplement: Supplementary file 1 [file biology-15-00765-s001.zip › Supplementary/File S7/phytoplankton_2021_05/P17_Chlorophyta taxa %.png]

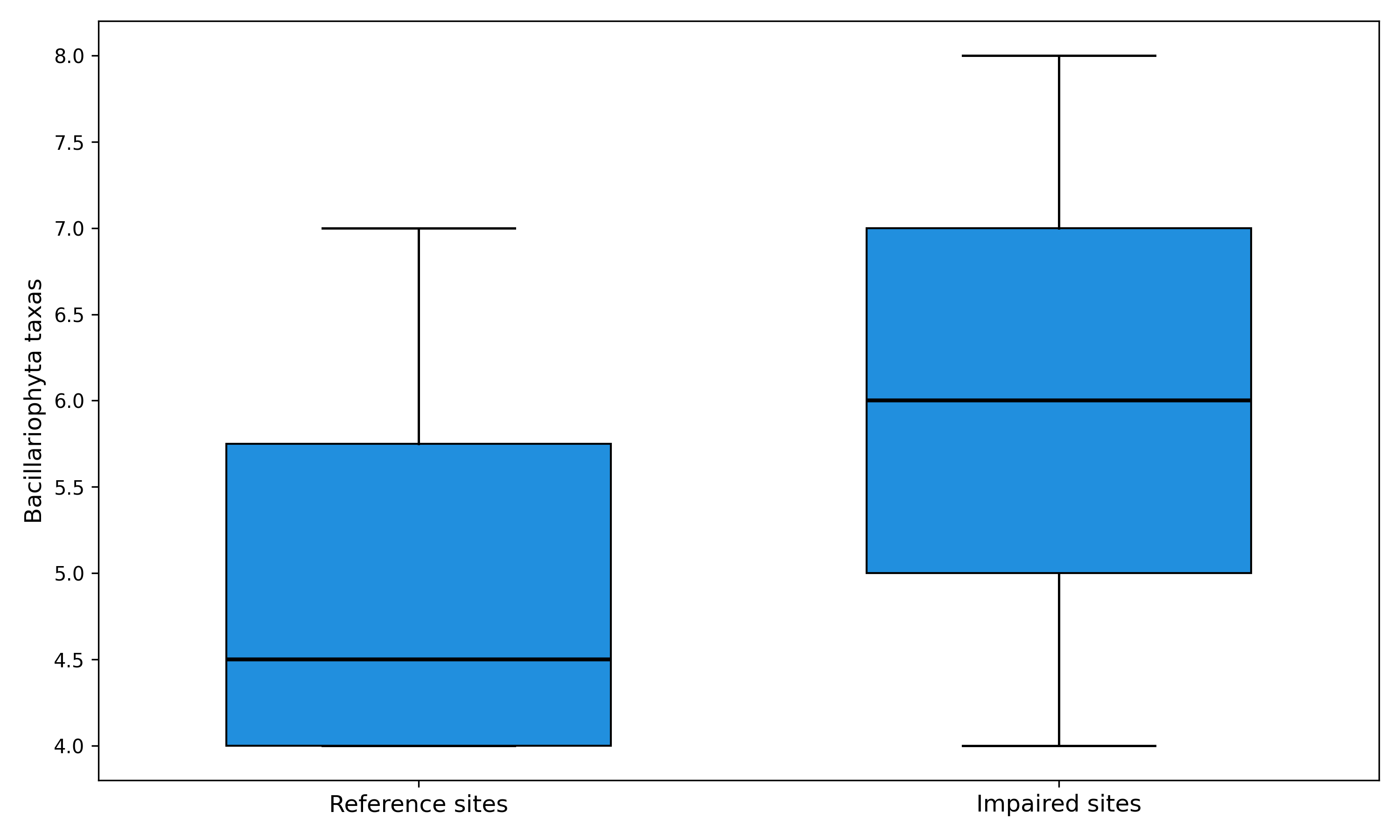

Supplement: Supplementary file 1 [file biology-15-00765-s001.zip › Supplementary/File S7/phytoplankton_2021_05/P2_Bacillariophyta taxas.png]

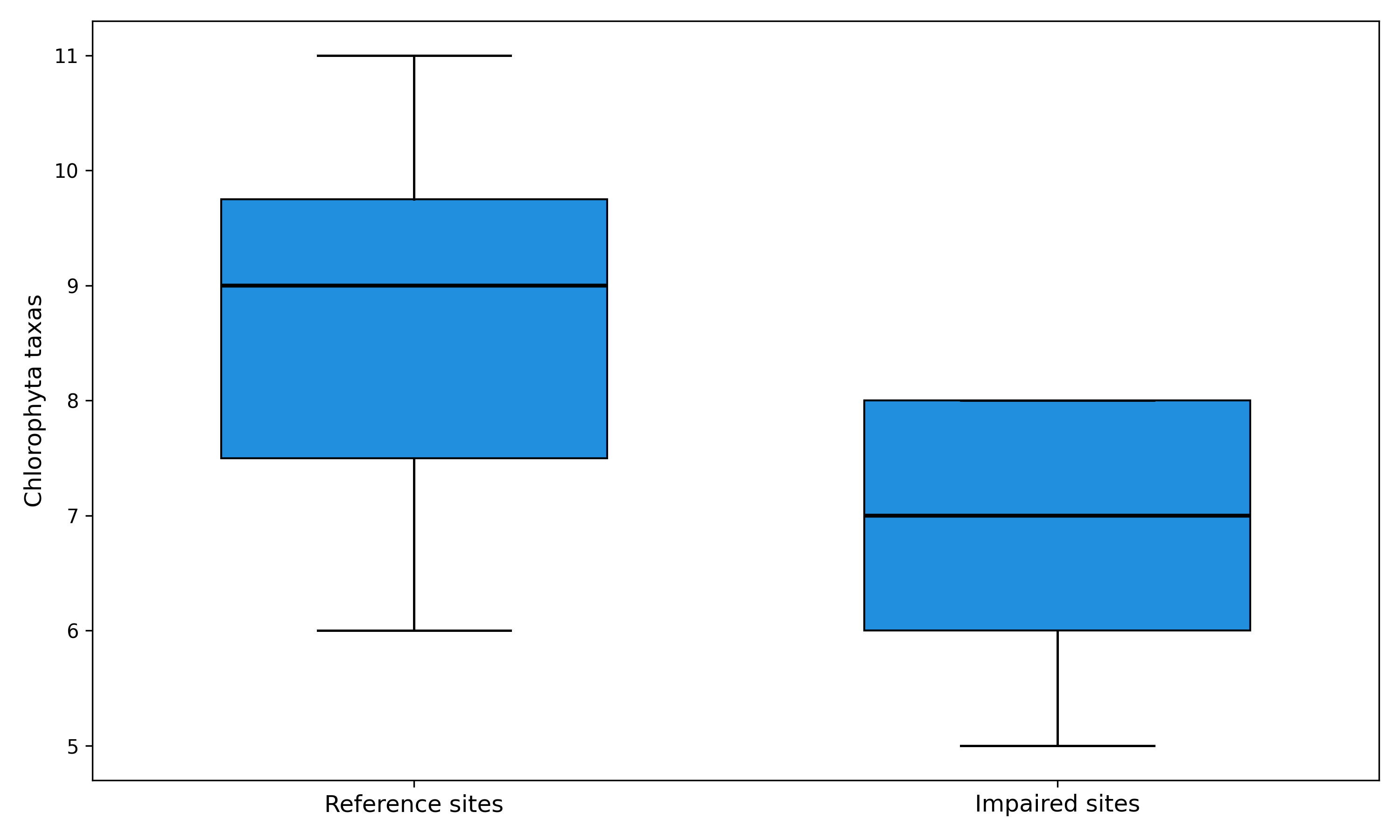

Supplement: Supplementary file 1 [file biology-15-00765-s001.zip › Supplementary/File S7/phytoplankton_2021_05/P4_Chlorophyta taxas.png]

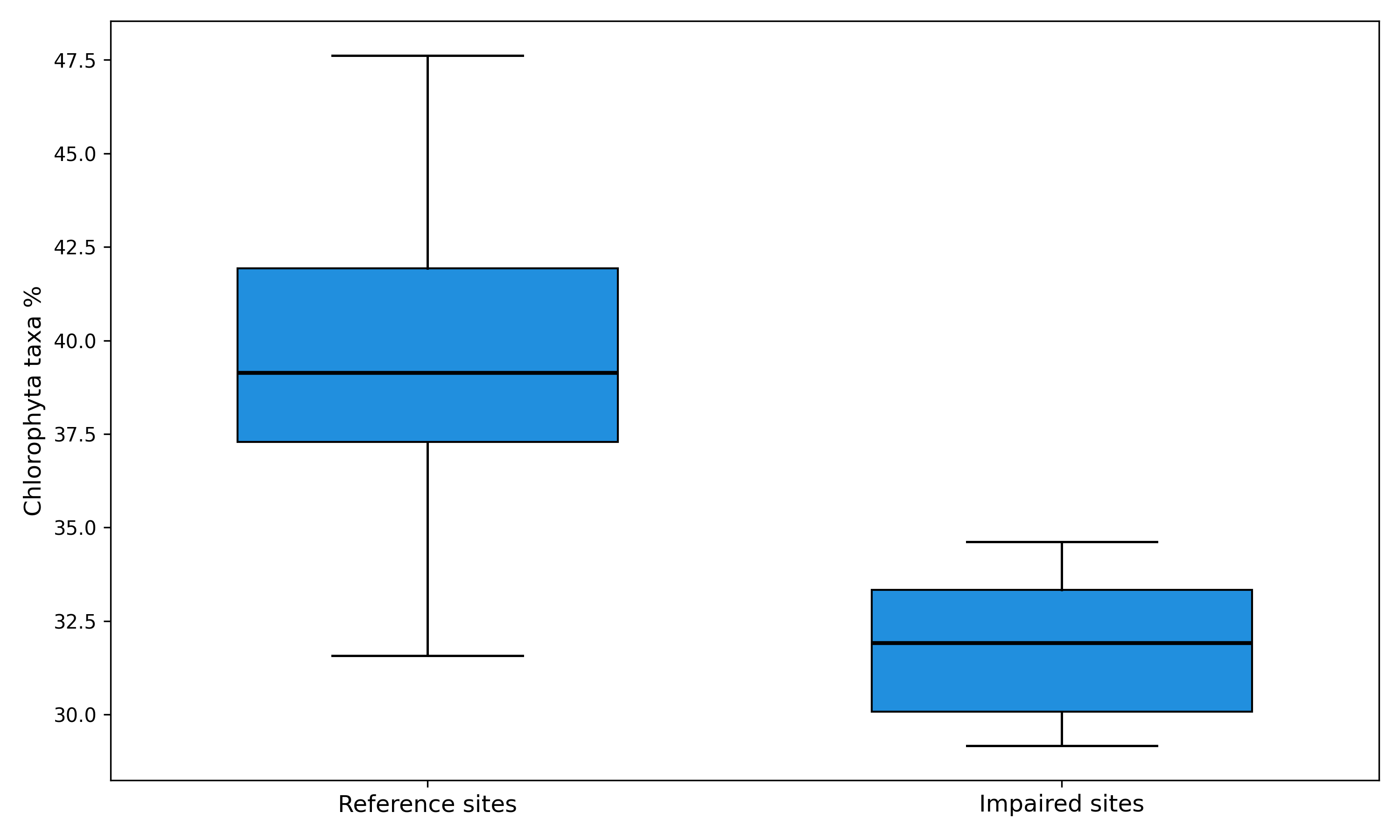

Supplement: Supplementary file 1 [file biology-15-00765-s001.zip › Supplementary/File S7/phytoplankton_2021_09/P17_Chlorophyta taxa %.png]

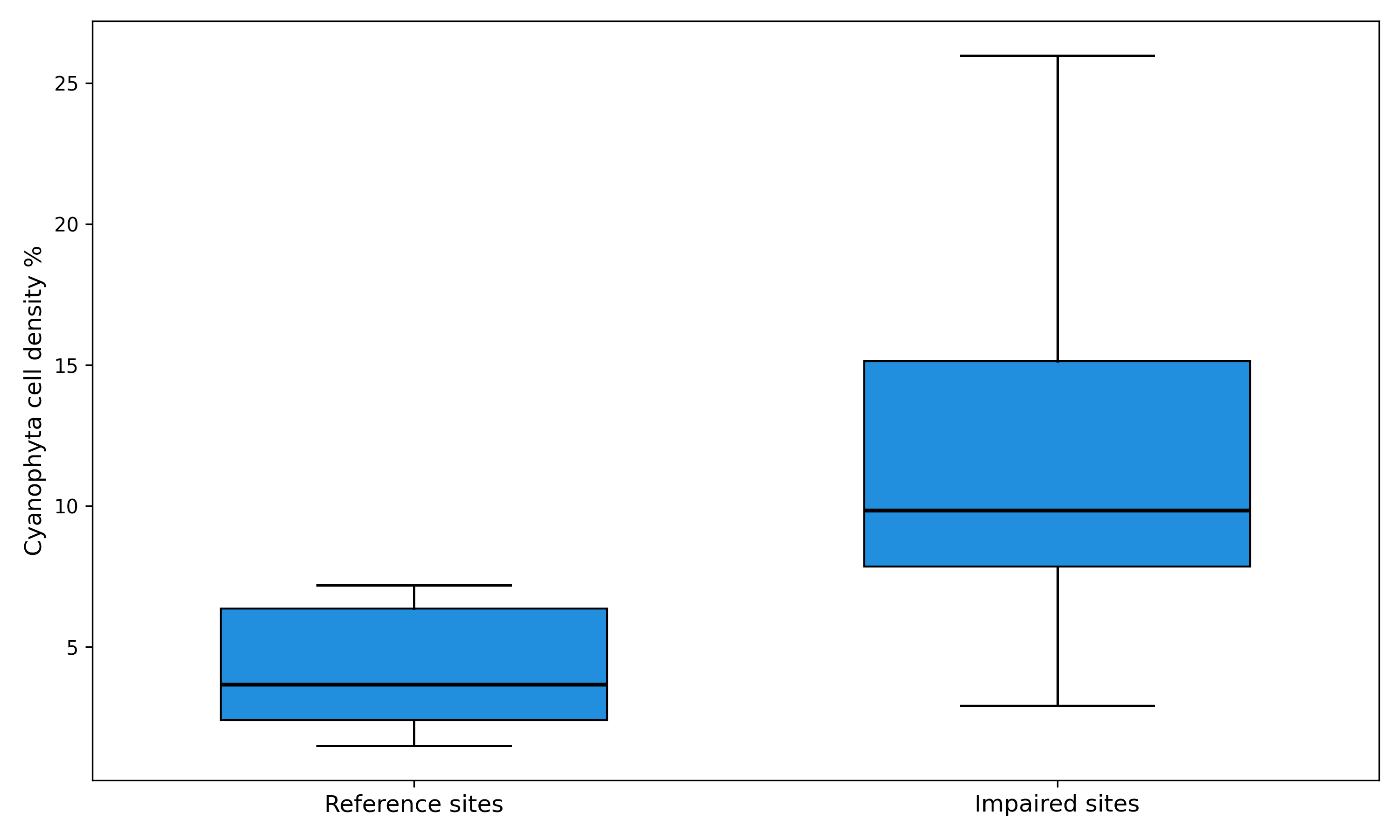

Supplement: Supplementary file 1 [file biology-15-00765-s001.zip › Supplementary/File S7/phytoplankton_2021_09/P19_Cyanophyta cell density %.png]

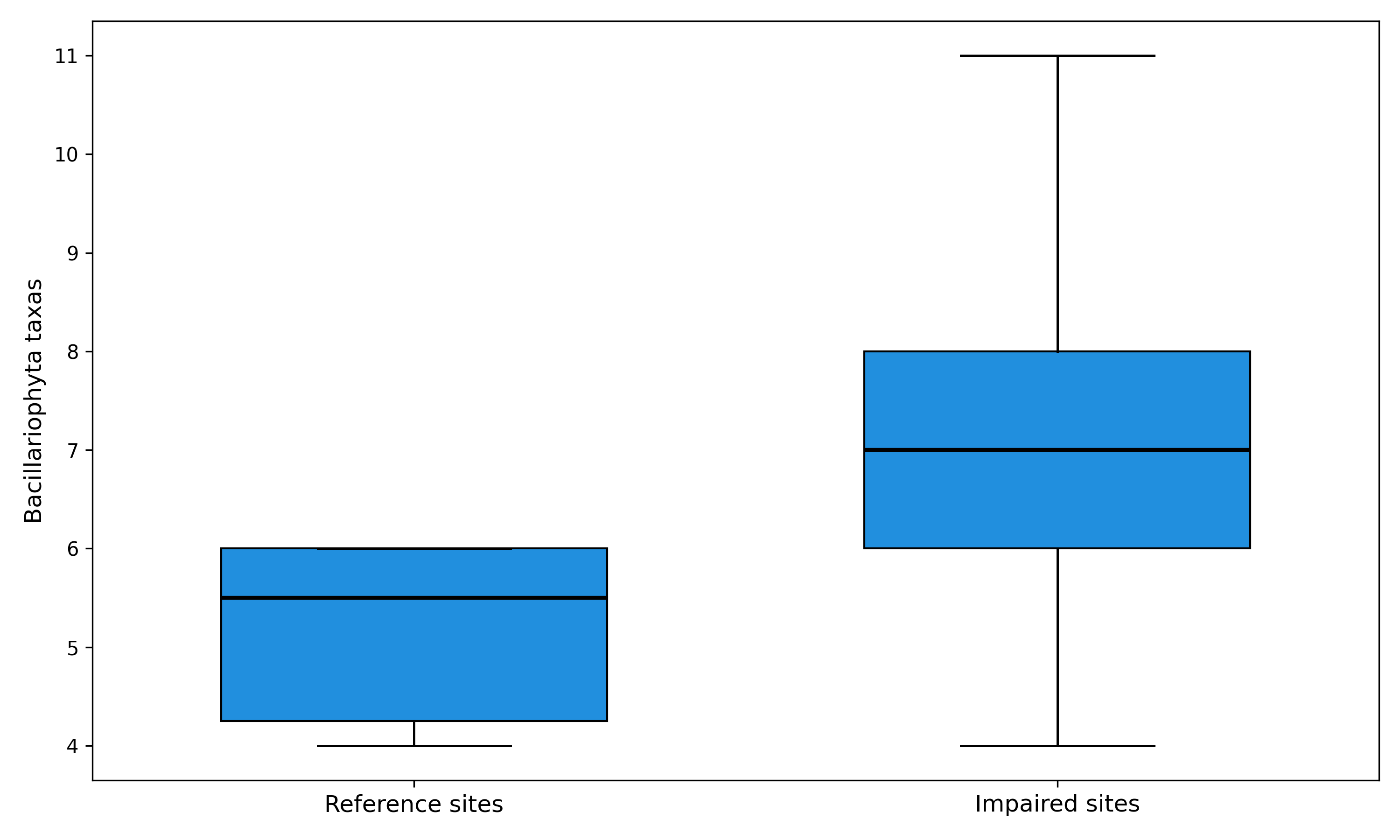

Supplement: Supplementary file 1 [file biology-15-00765-s001.zip › Supplementary/File S7/phytoplankton_2021_09/P2_Bacillariophyta taxas.png]

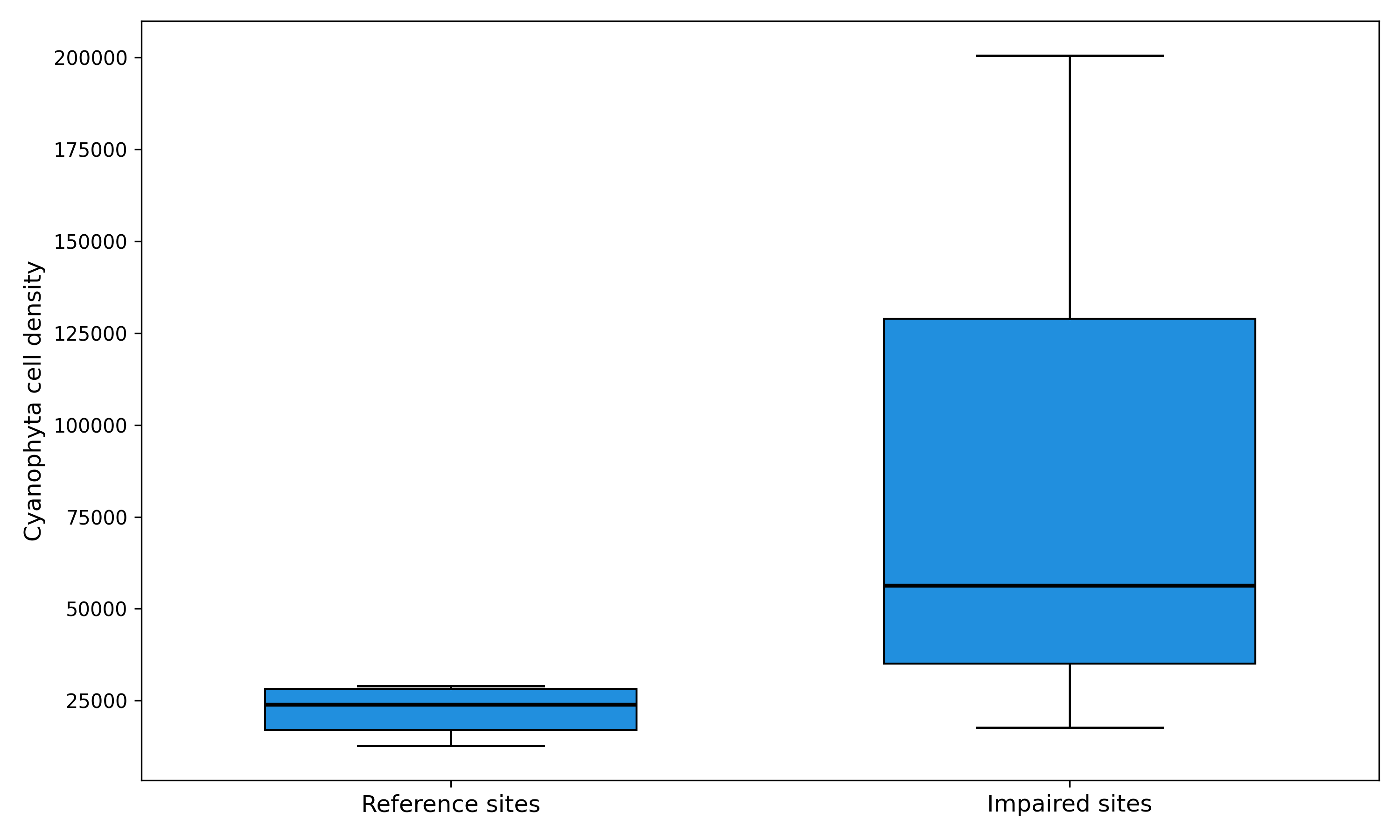

Supplement: Supplementary file 1 [file biology-15-00765-s001.zip › Supplementary/File S7/phytoplankton_2021_09/P7_Cyanophyta cell density.png]

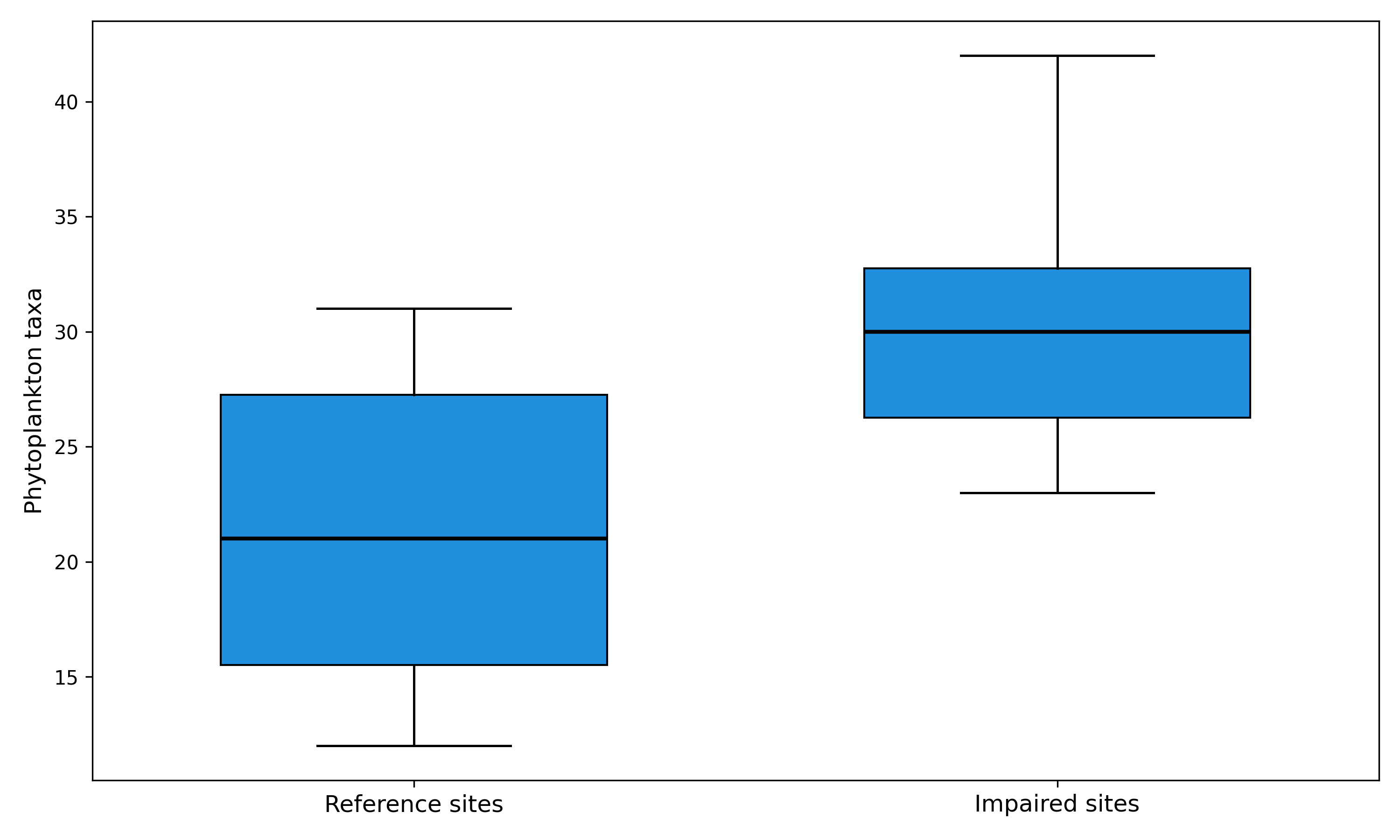

Supplement: Supplementary file 1 [file biology-15-00765-s001.zip › Supplementary/File S7/phytoplankton_2022_05/P1_Phytoplankton taxa.png]

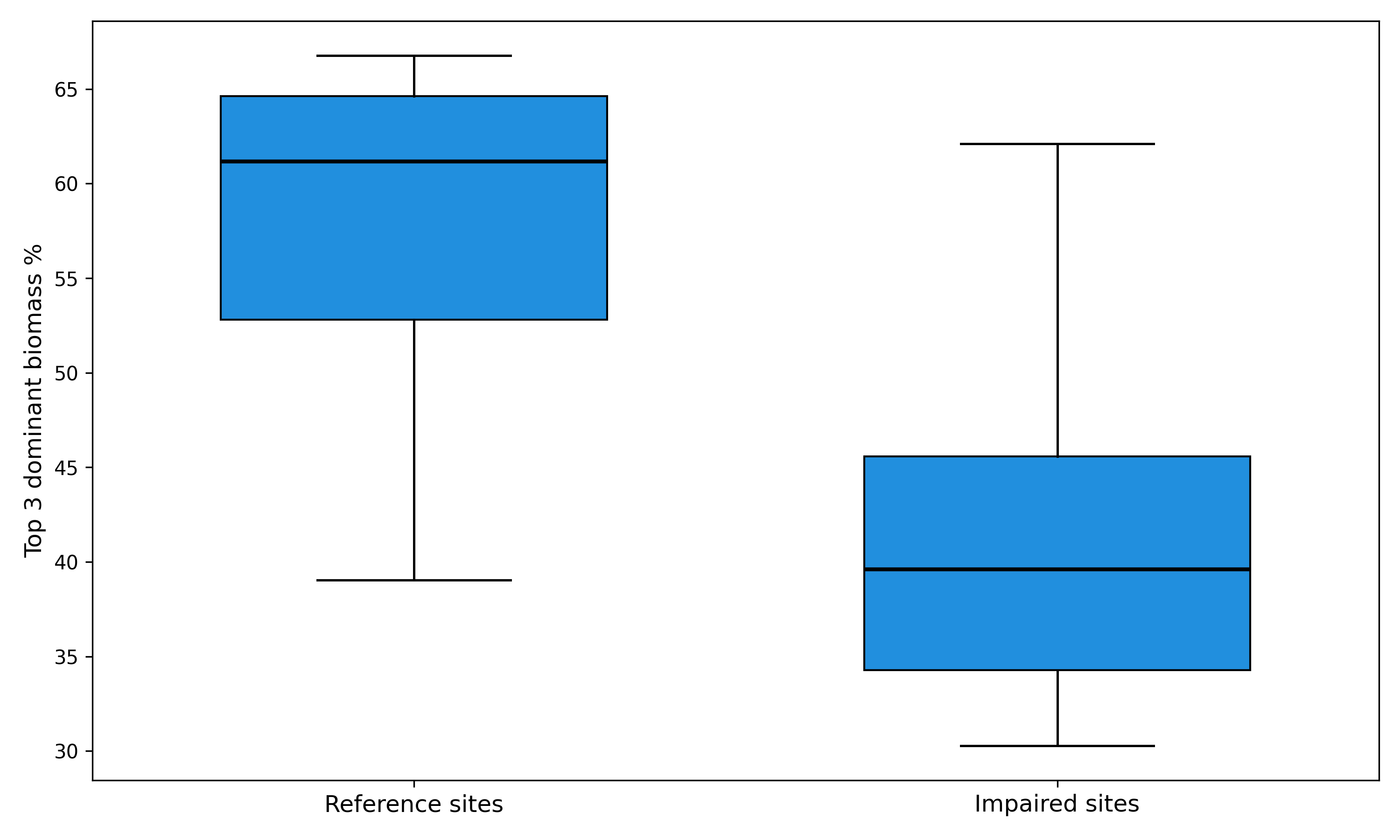

Supplement: Supplementary file 1 [file biology-15-00765-s001.zip › Supplementary/File S7/phytoplankton_2022_05/P26_Top 3 dominant biomass %.png]

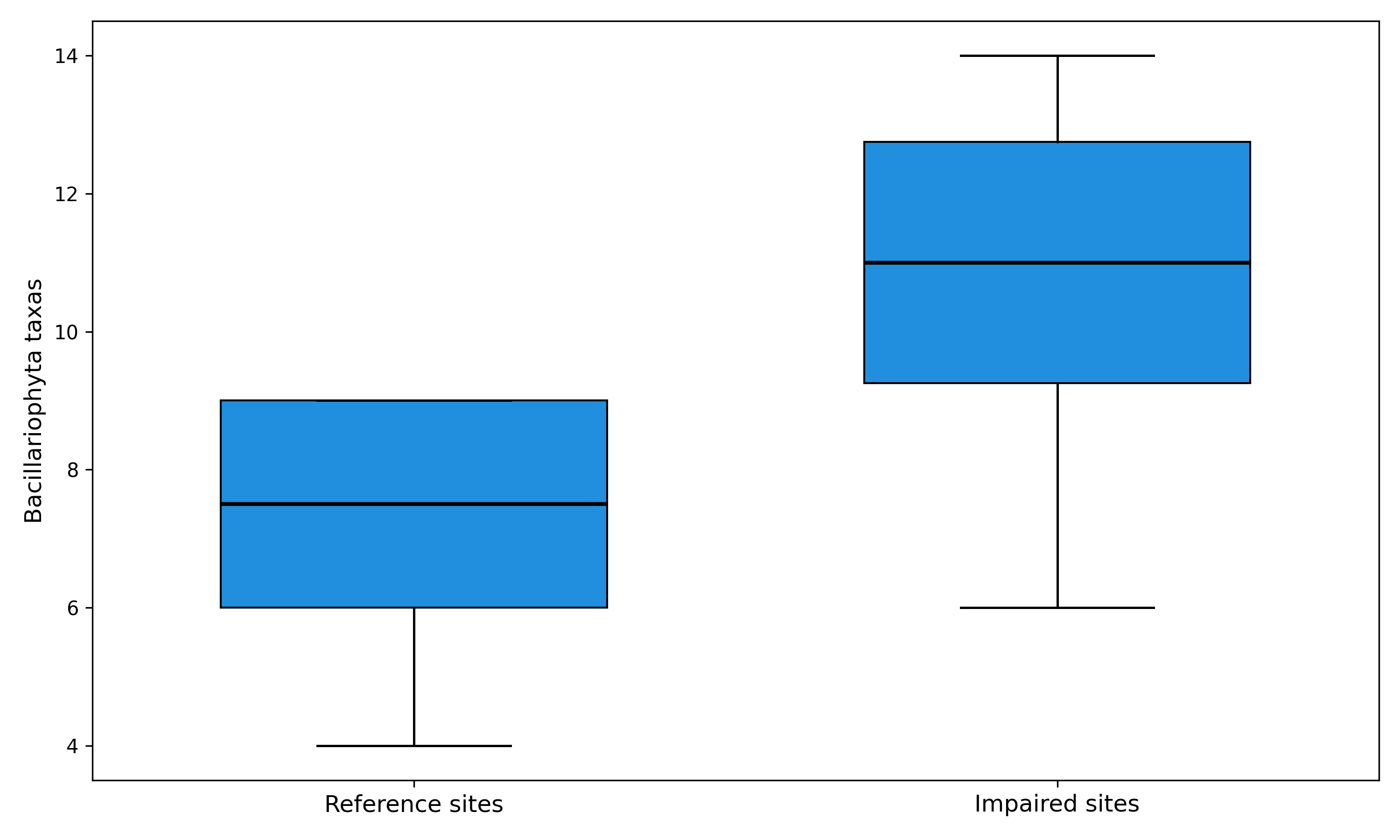

Supplement: Supplementary file 1 [file biology-15-00765-s001.zip › Supplementary/File S7/phytoplankton_2022_05/P2_Bacillariophyta taxas.png]

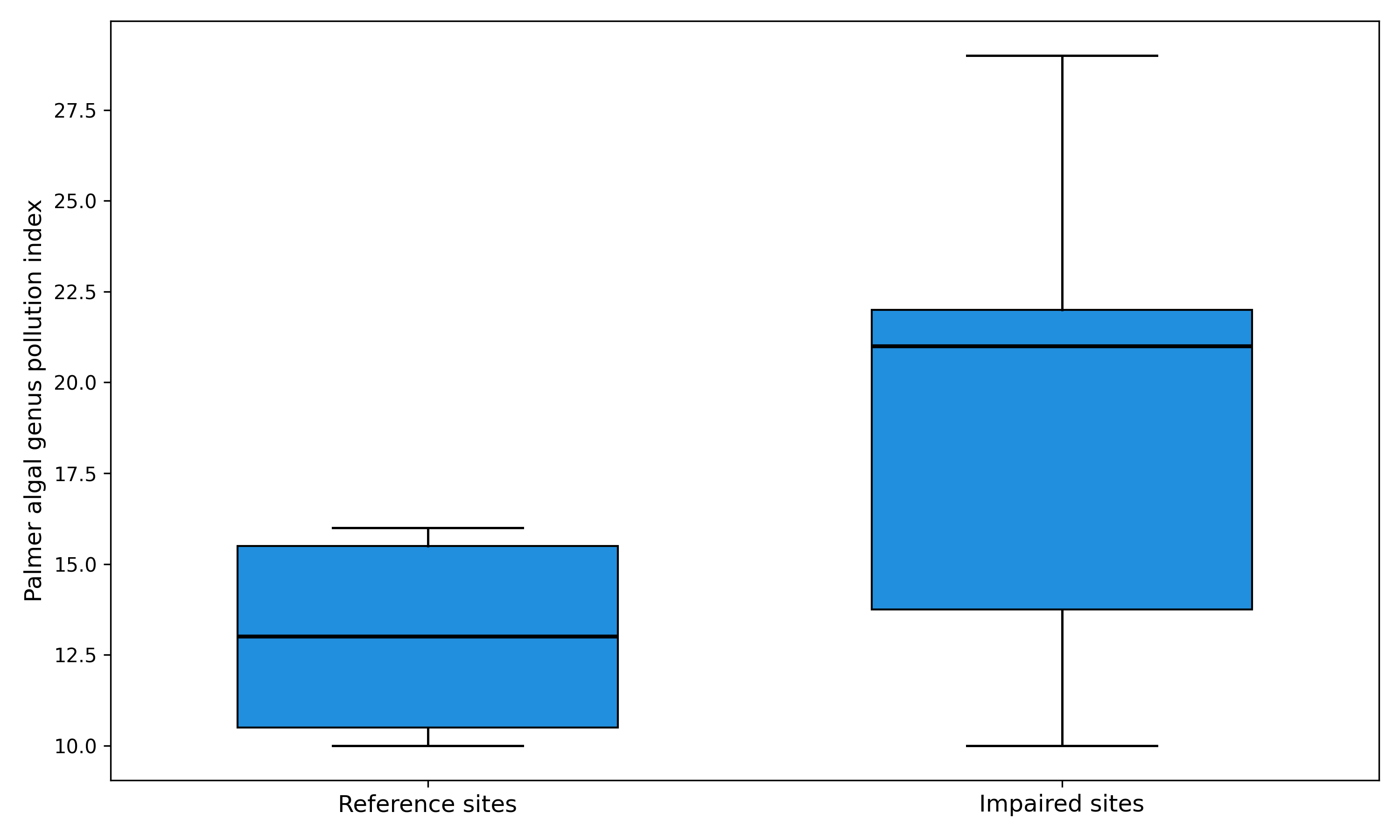

Supplement: Supplementary file 1 [file biology-15-00765-s001.zip › Supplementary/File S7/phytoplankton_2022_05/P32_Palmer algal genus pollution index.png]

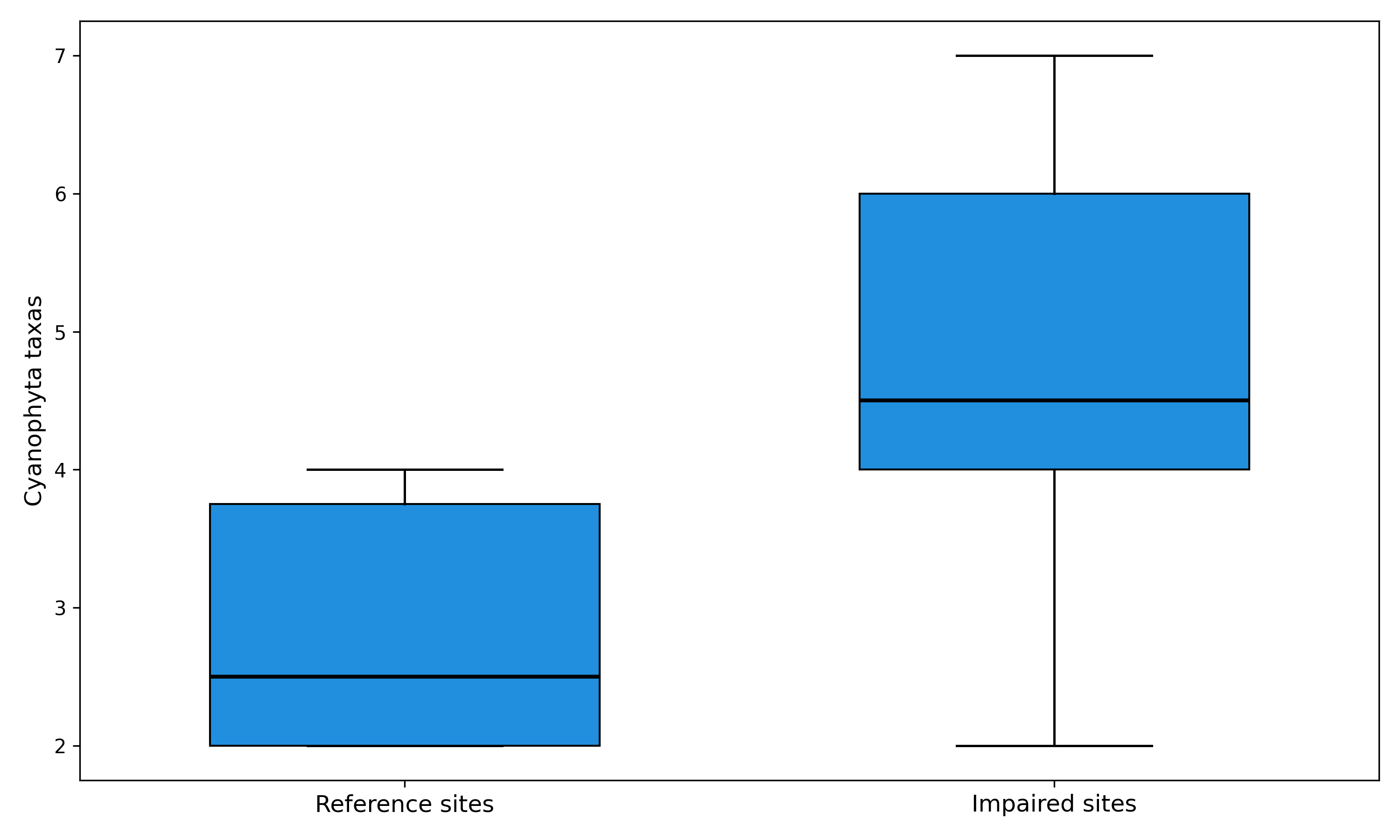

Supplement: Supplementary file 1 [file biology-15-00765-s001.zip › Supplementary/File S7/phytoplankton_2022_05/P3_Cyanophyta taxas.png]

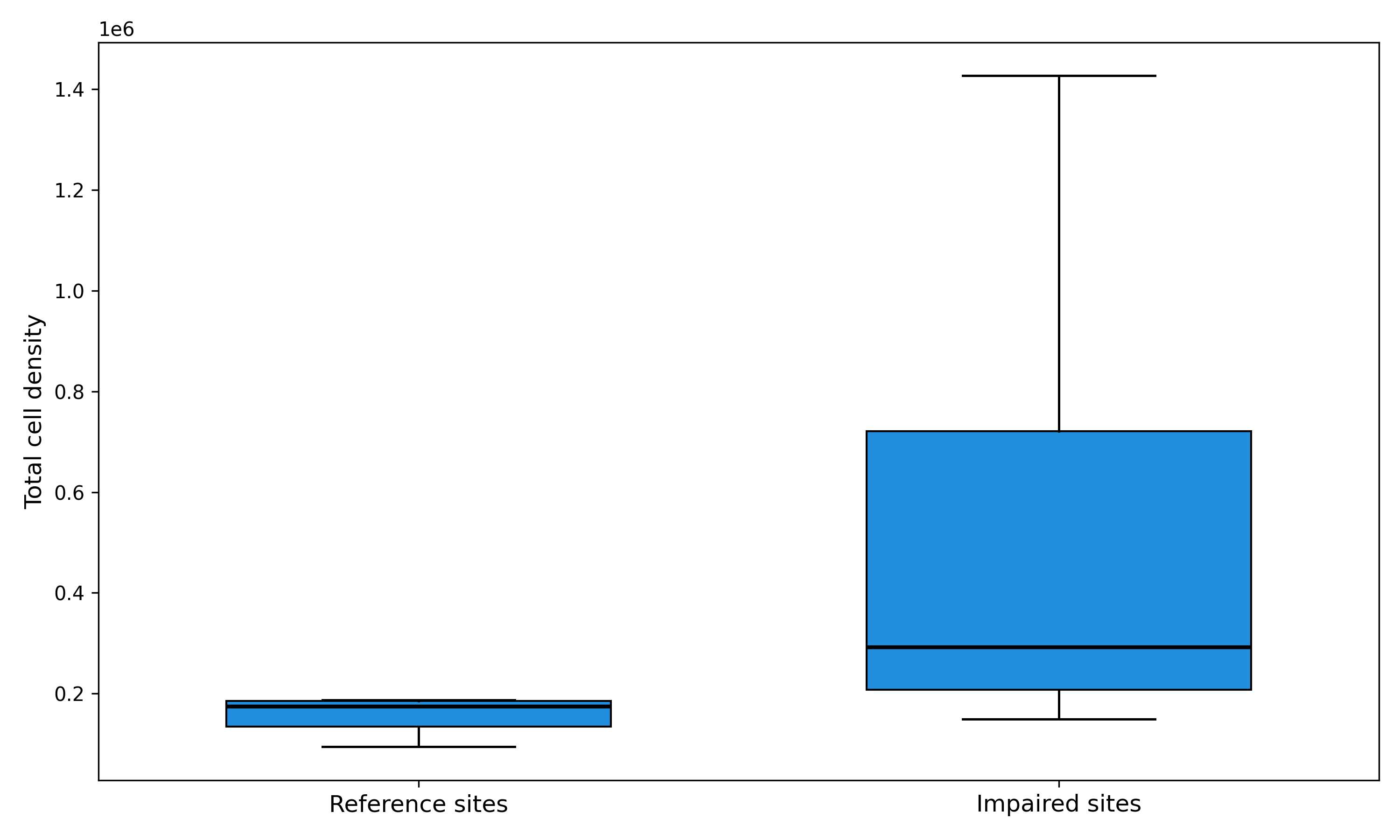

Supplement: Supplementary file 1 [file biology-15-00765-s001.zip › Supplementary/File S7/phytoplankton_2022_05/P5_Total cell density.png]

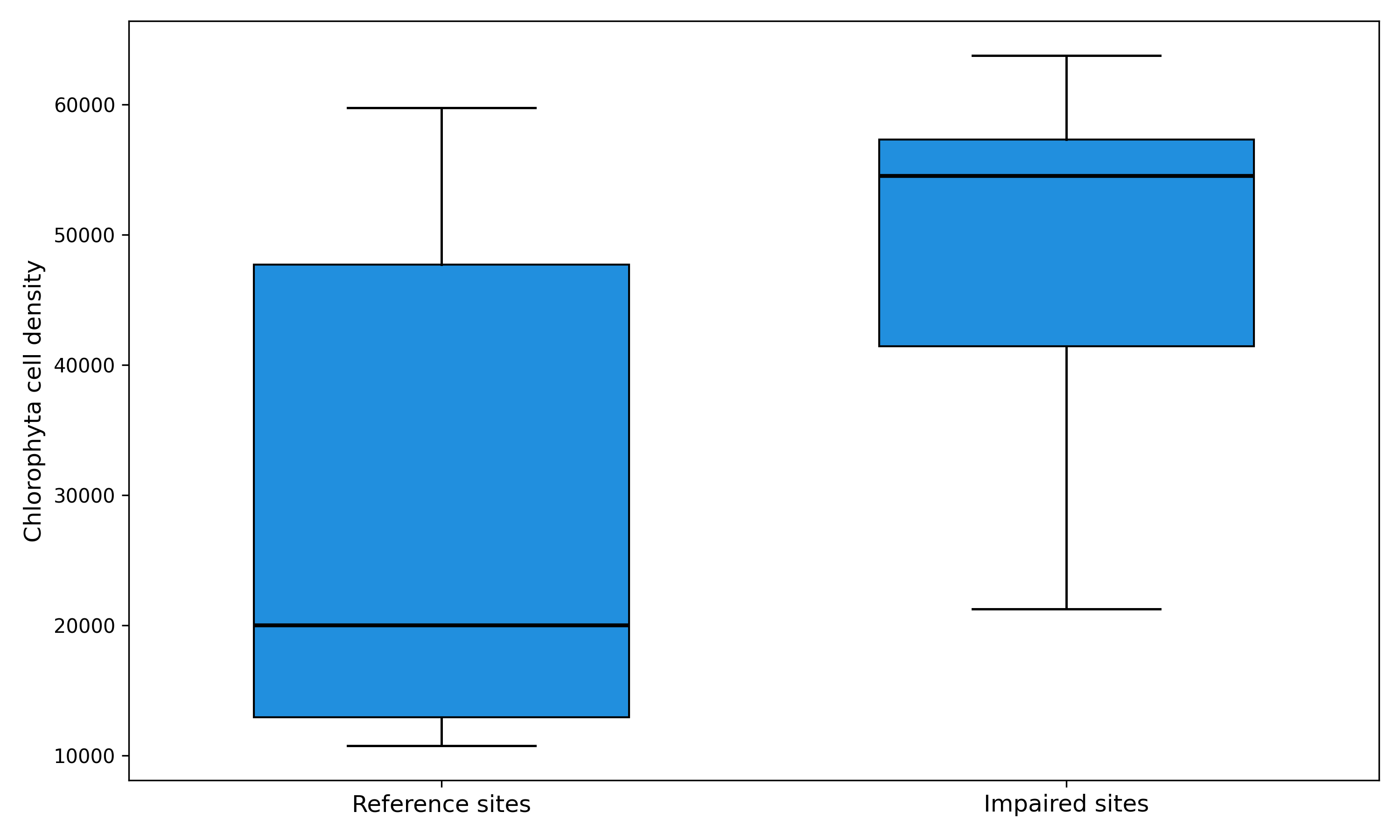

Supplement: Supplementary file 1 [file biology-15-00765-s001.zip › Supplementary/File S7/phytoplankton_2022_05/P8_Chlorophyta cell density.png]

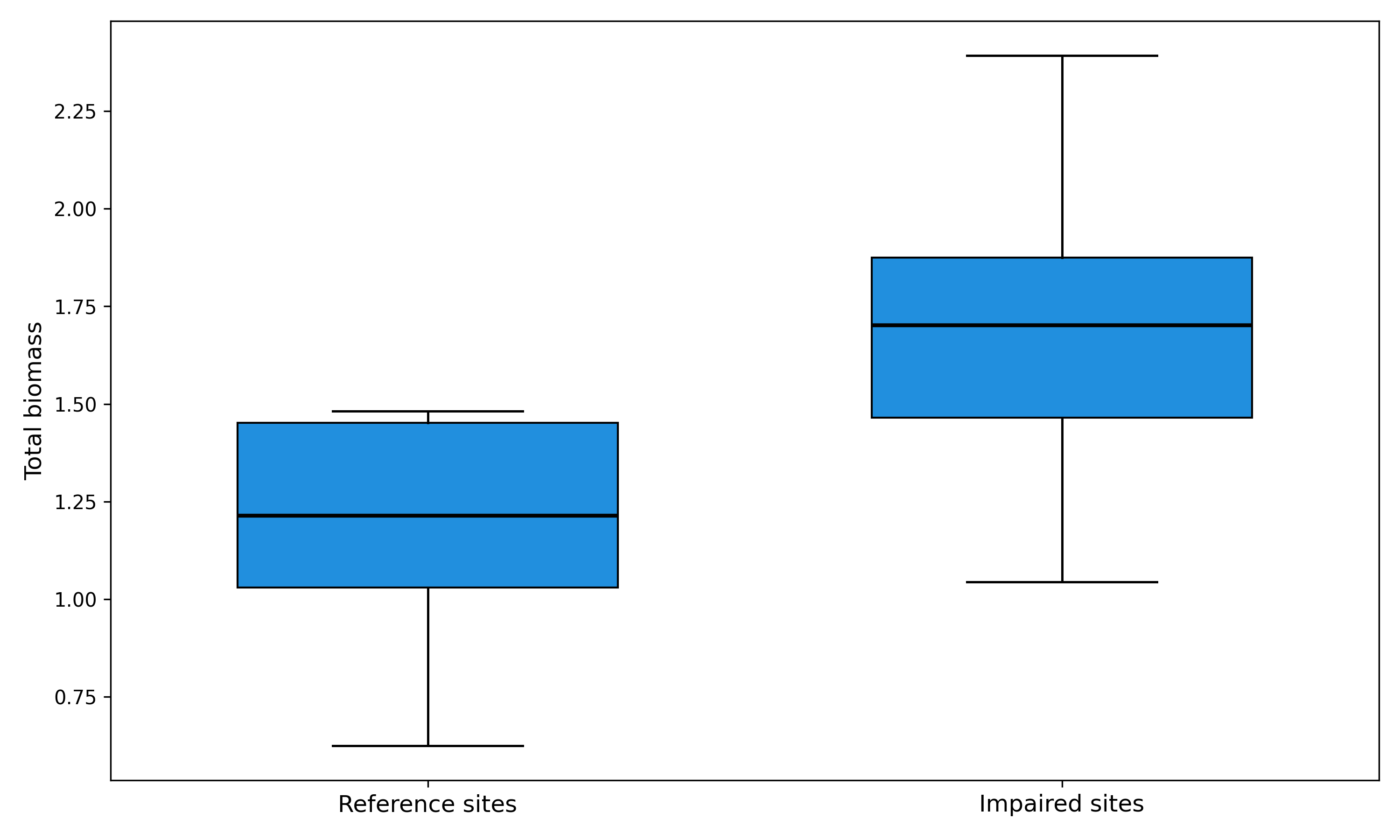

Supplement: Supplementary file 1 [file biology-15-00765-s001.zip › Supplementary/File S7/phytoplankton_2022_05/P9_Total biomass.png]

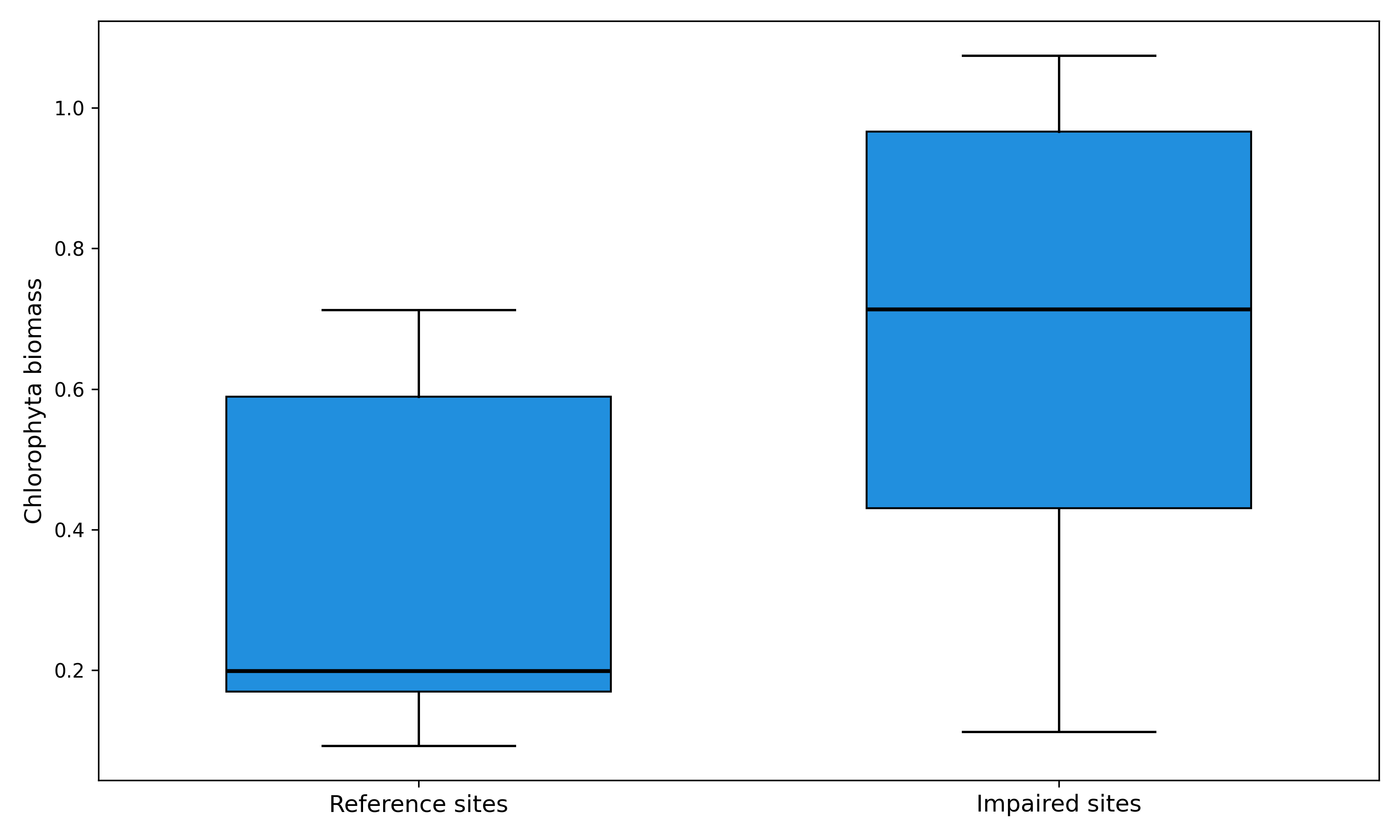

Supplement: Supplementary file 1 [file biology-15-00765-s001.zip › Supplementary/File S7/phytoplankton_2022_09/P12_Chlorophyta biomass.png]

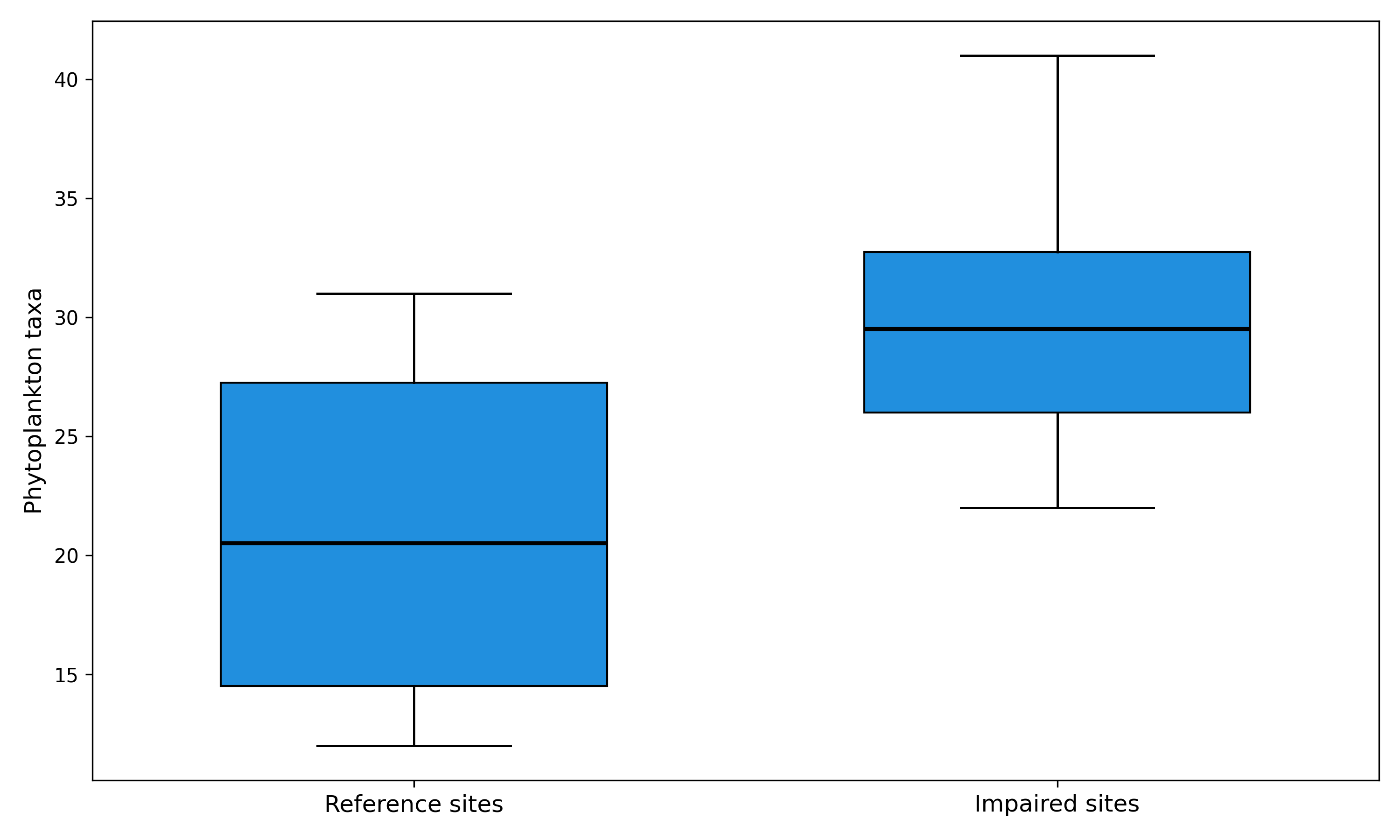

Supplement: Supplementary file 1 [file biology-15-00765-s001.zip › Supplementary/File S7/phytoplankton_2022_09/P1_Phytoplankton taxa.png]

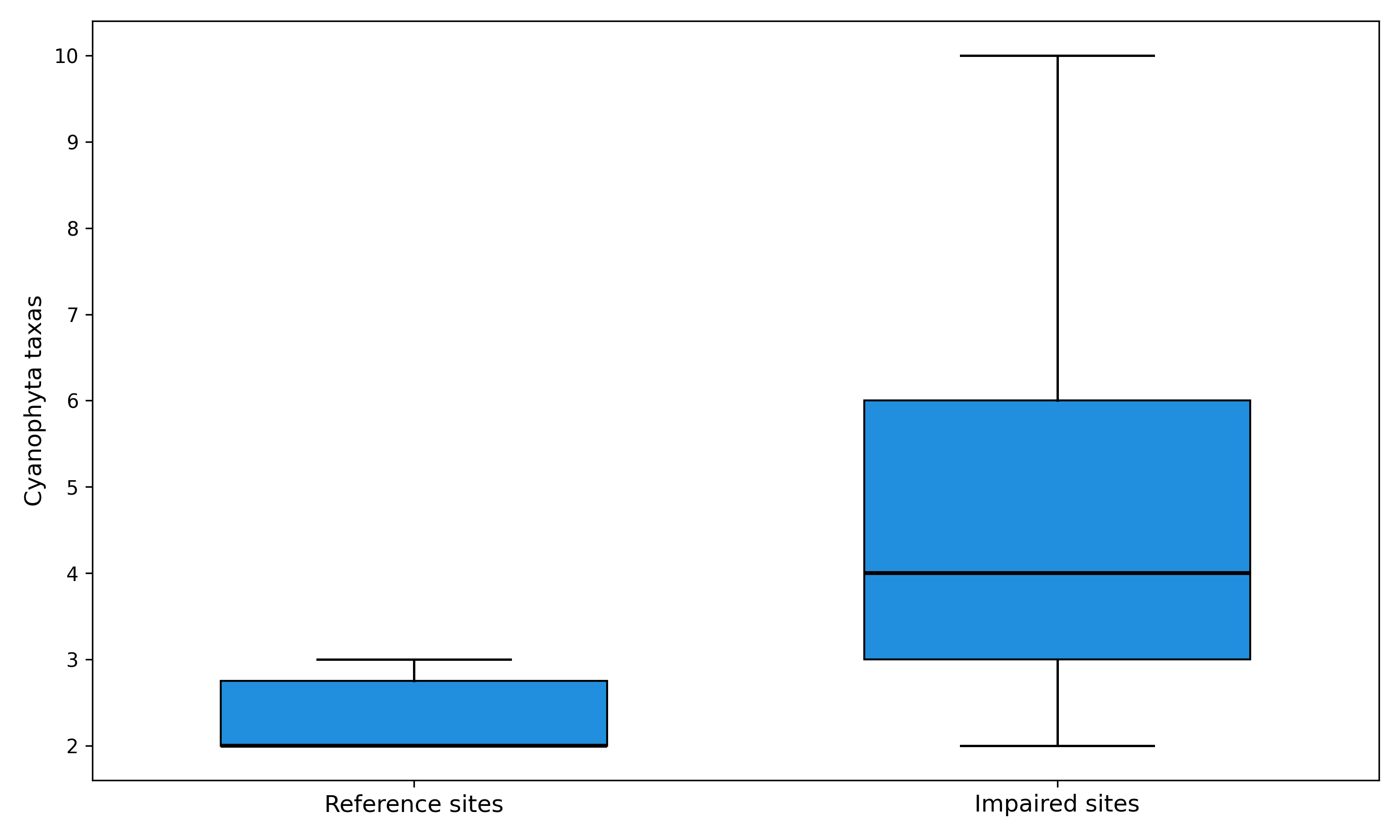

Supplement: Supplementary file 1 [file biology-15-00765-s001.zip › Supplementary/File S7/phytoplankton_2022_09/P3_Cyanophyta taxas.png]

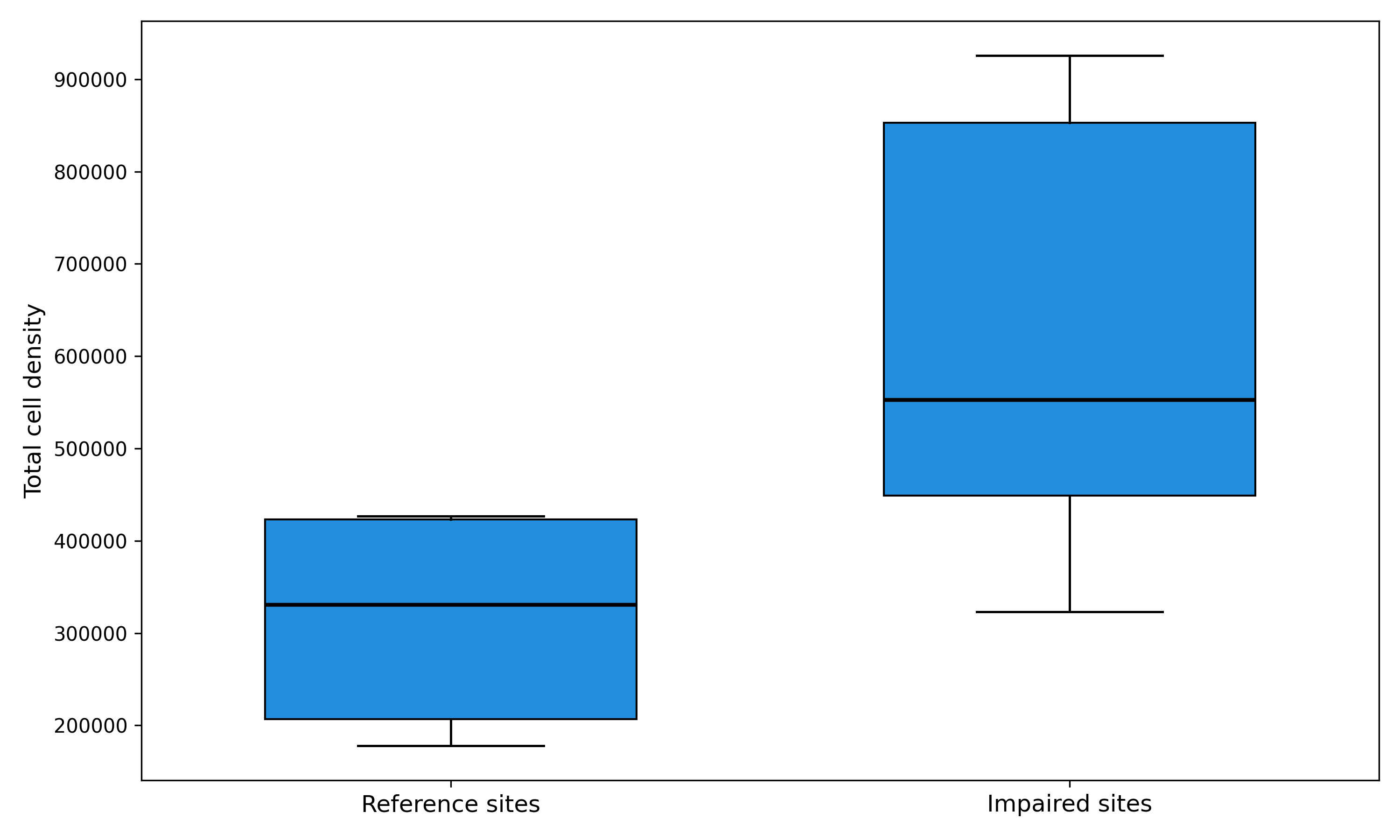

Supplement: Supplementary file 1 [file biology-15-00765-s001.zip › Supplementary/File S7/phytoplankton_2022_09/P5_Total cell density.png]

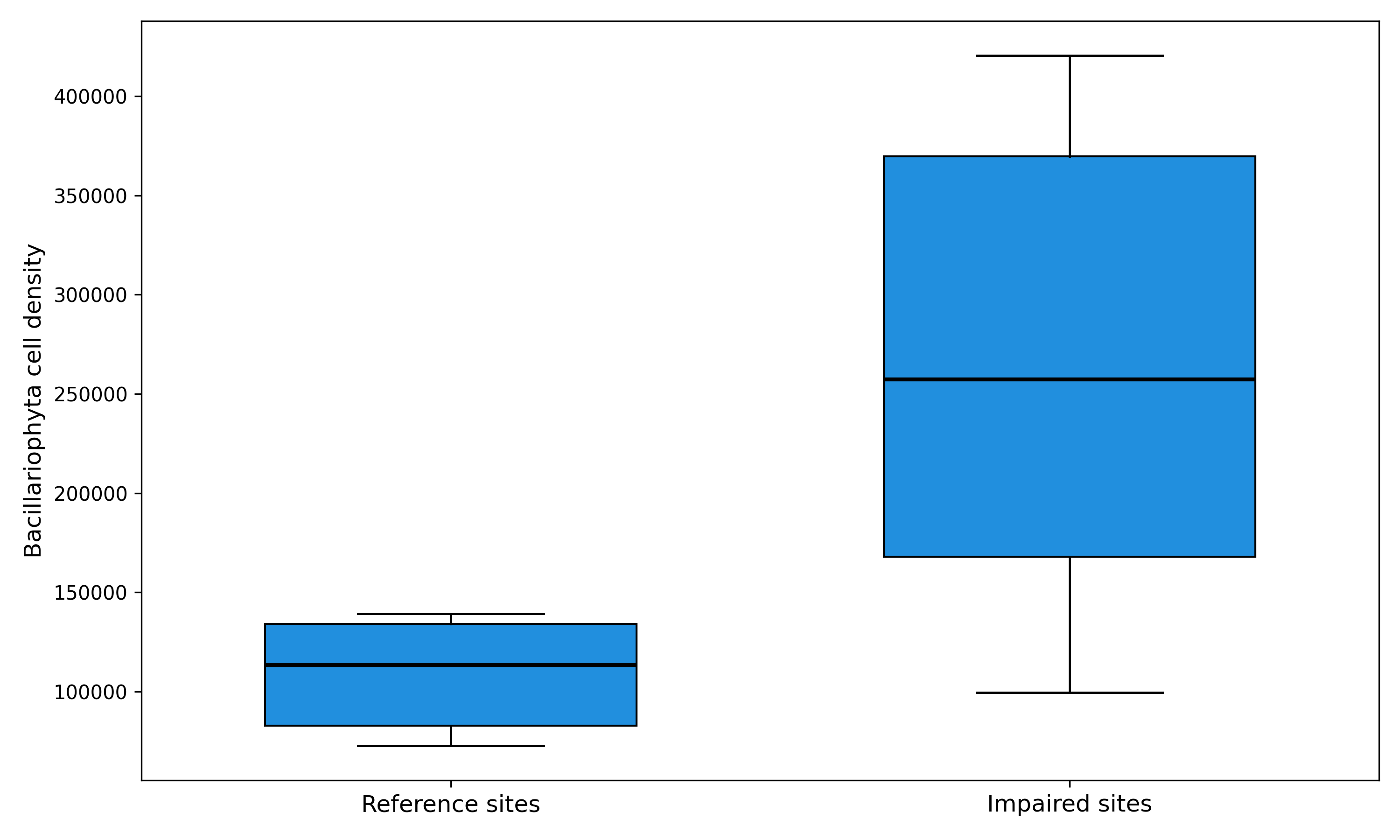

Supplement: Supplementary file 1 [file biology-15-00765-s001.zip › Supplementary/File S7/phytoplankton_2022_09/P6_Bacillariophyta cell density.png]

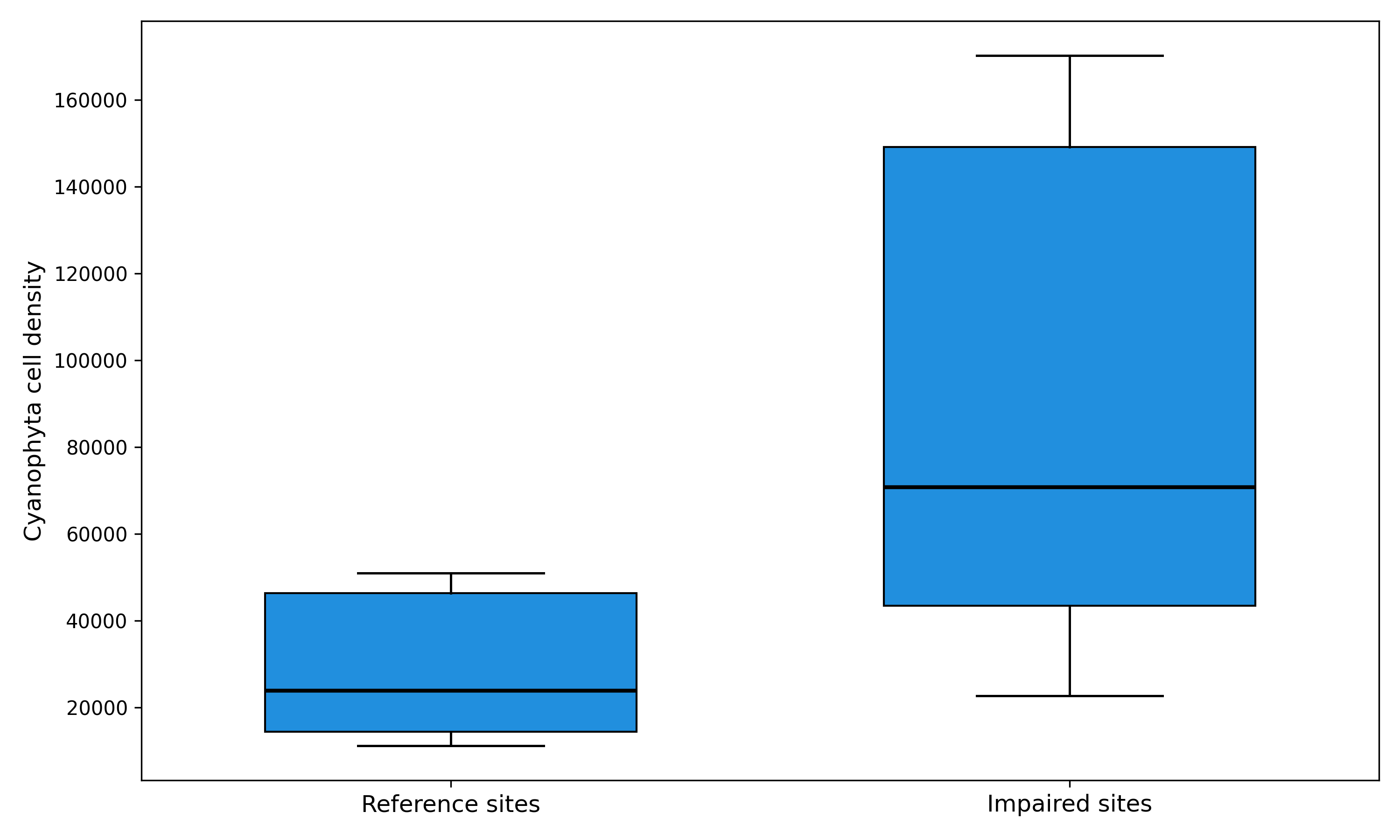

Supplement: Supplementary file 1 [file biology-15-00765-s001.zip › Supplementary/File S7/phytoplankton_2022_09/P7_Cyanophyta cell density.png]

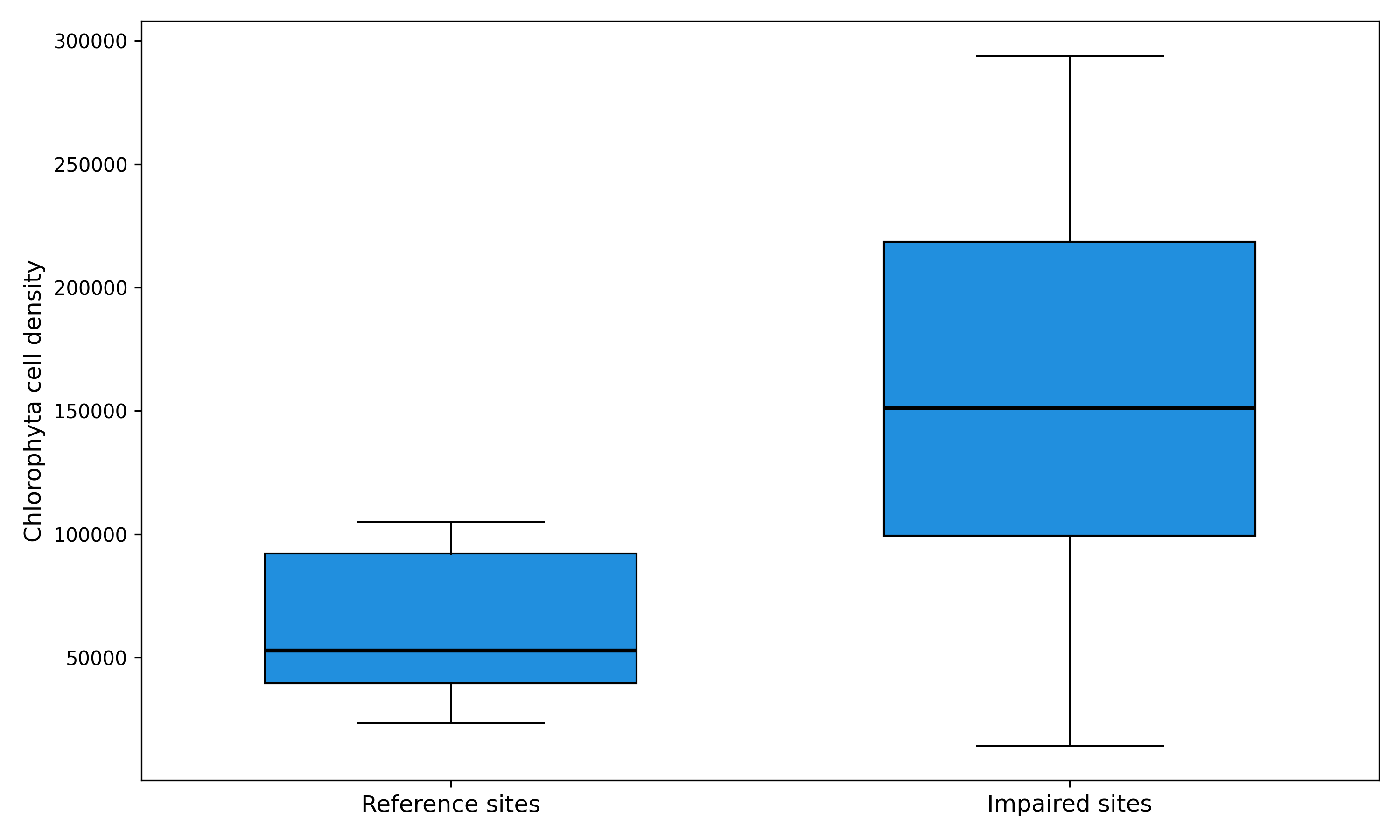

Supplement: Supplementary file 1 [file biology-15-00765-s001.zip › Supplementary/File S7/phytoplankton_2022_09/P8_Chlorophyta cell density.png]

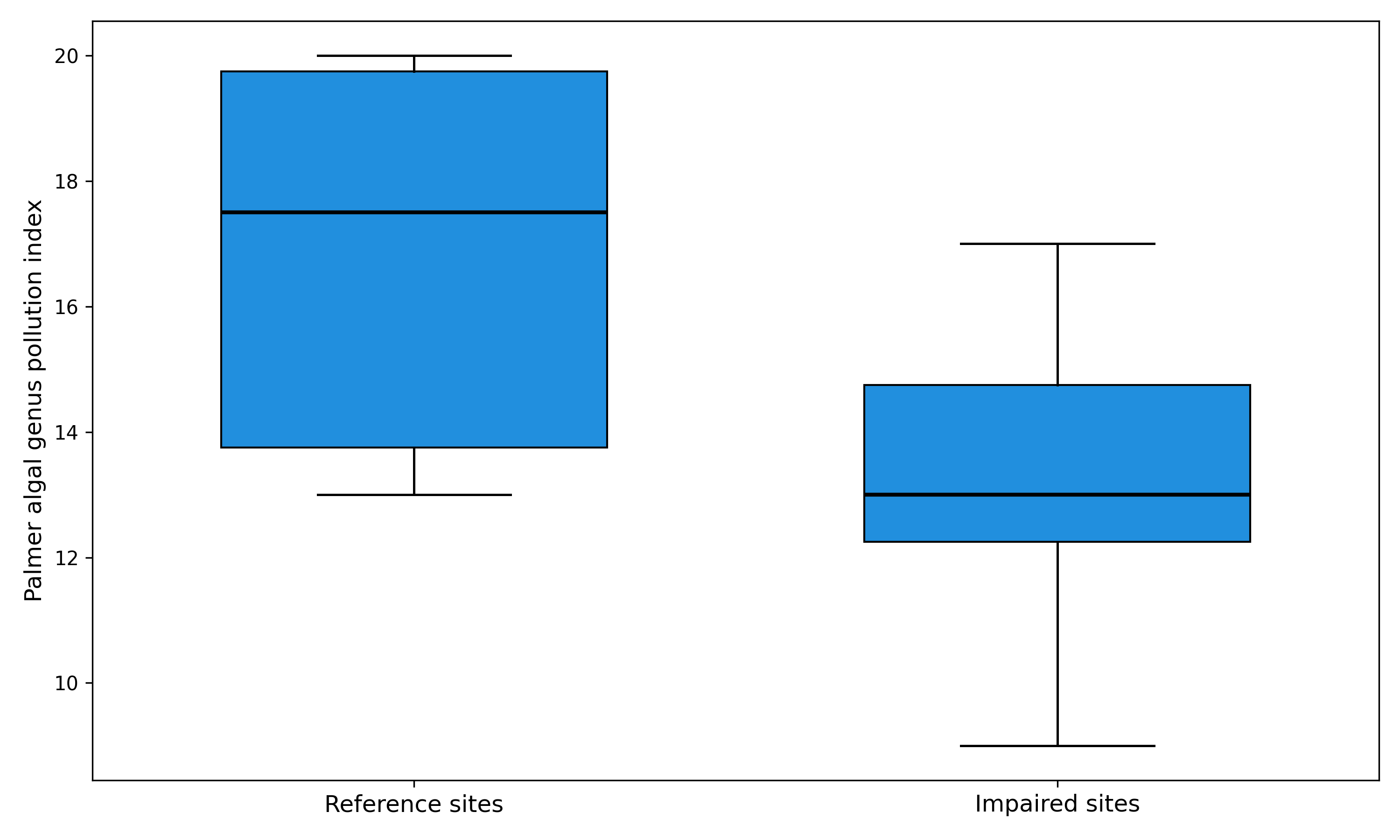

Supplement: Supplementary file 1 [file biology-15-00765-s001.zip › Supplementary/File S7/phytoplankton_2023_05/P32_Palmer algal genus pollution index.png]

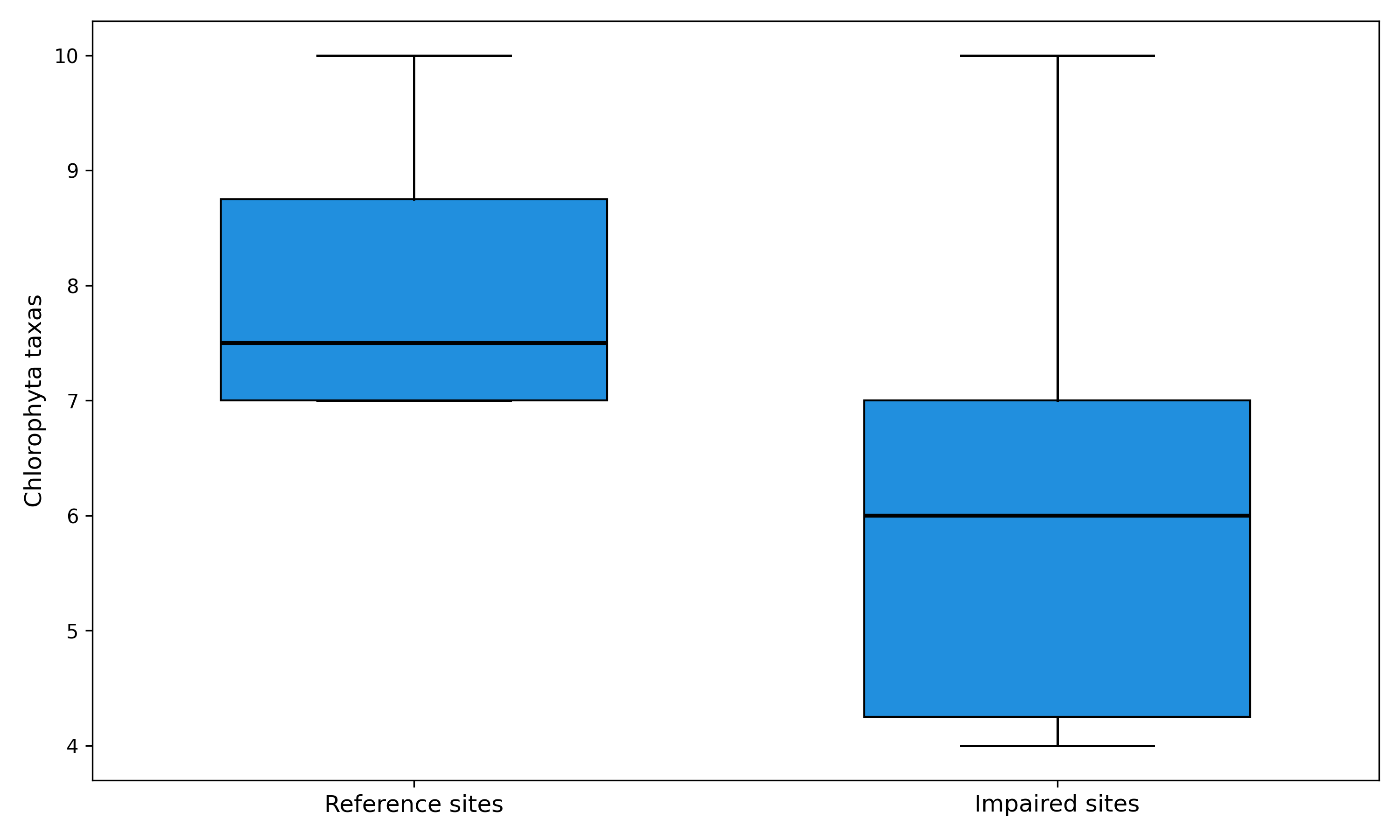

Supplement: Supplementary file 1 [file biology-15-00765-s001.zip › Supplementary/File S7/phytoplankton_2023_05/P4_Chlorophyta taxas.png]

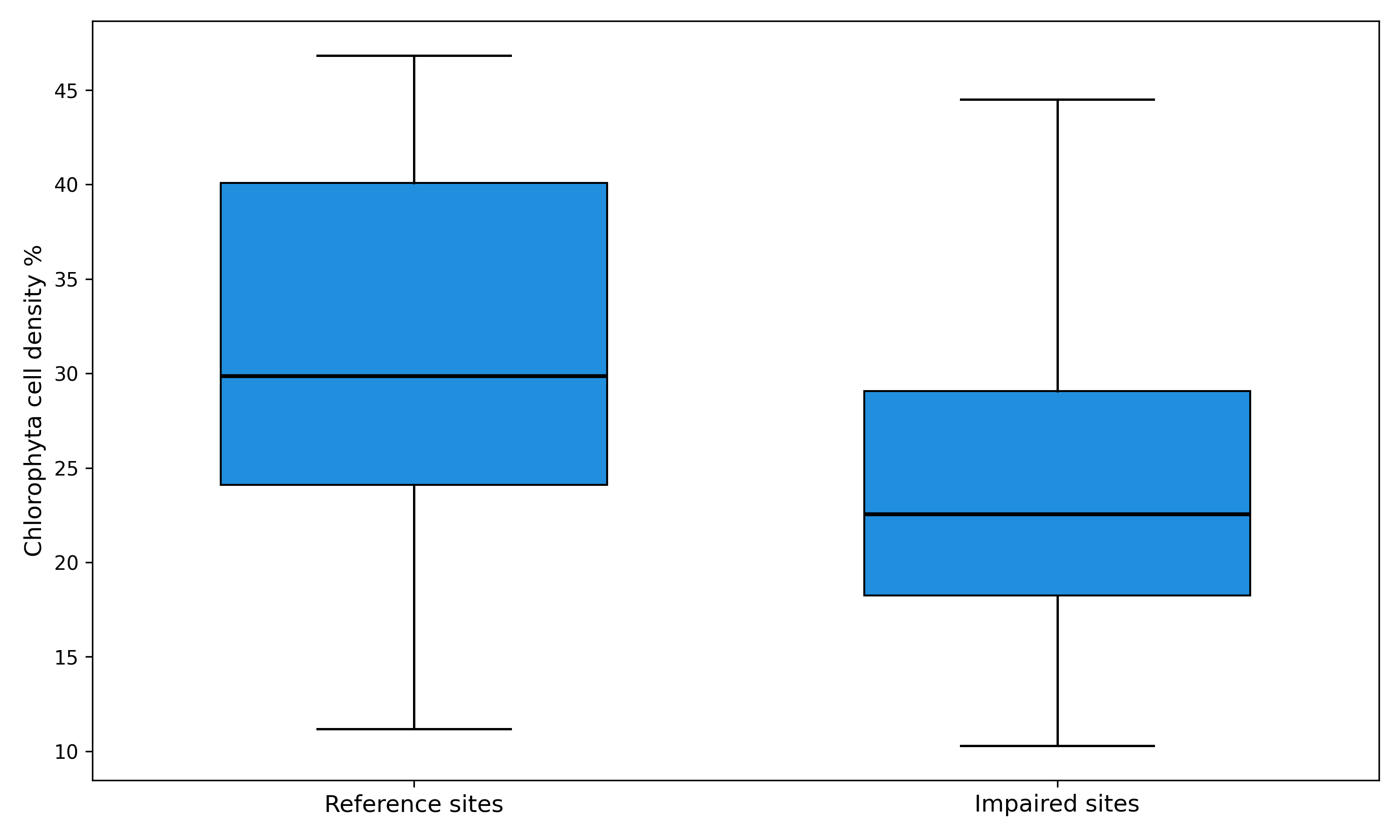

Supplement: Supplementary file 1 [file biology-15-00765-s001.zip › Supplementary/File S7/phytoplankton_2023_09/P20_Chlorophyta cell density %.png]

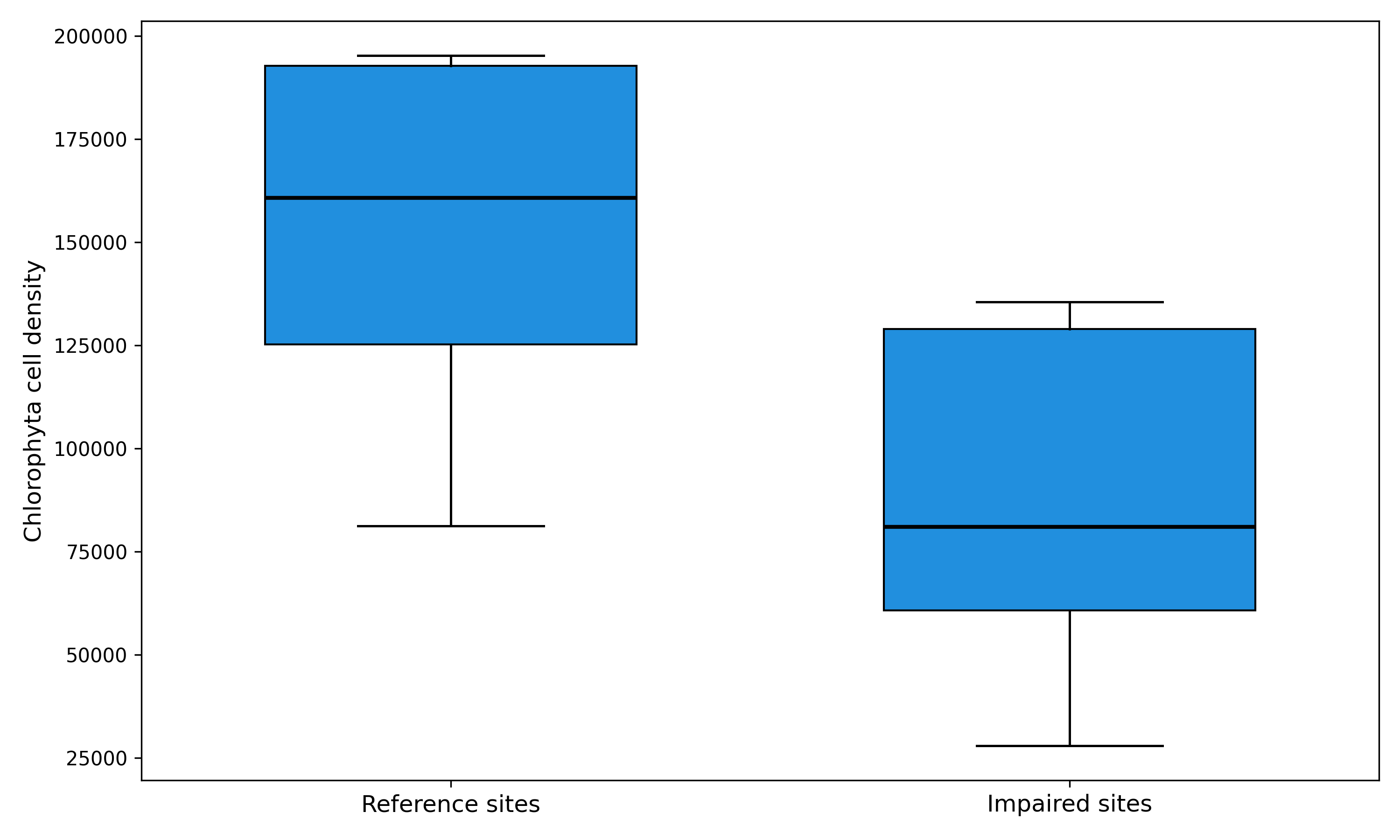

Supplement: Supplementary file 1 [file biology-15-00765-s001.zip › Supplementary/File S7/phytoplankton_2023_09/P8_Chlorophyta cell density.png]

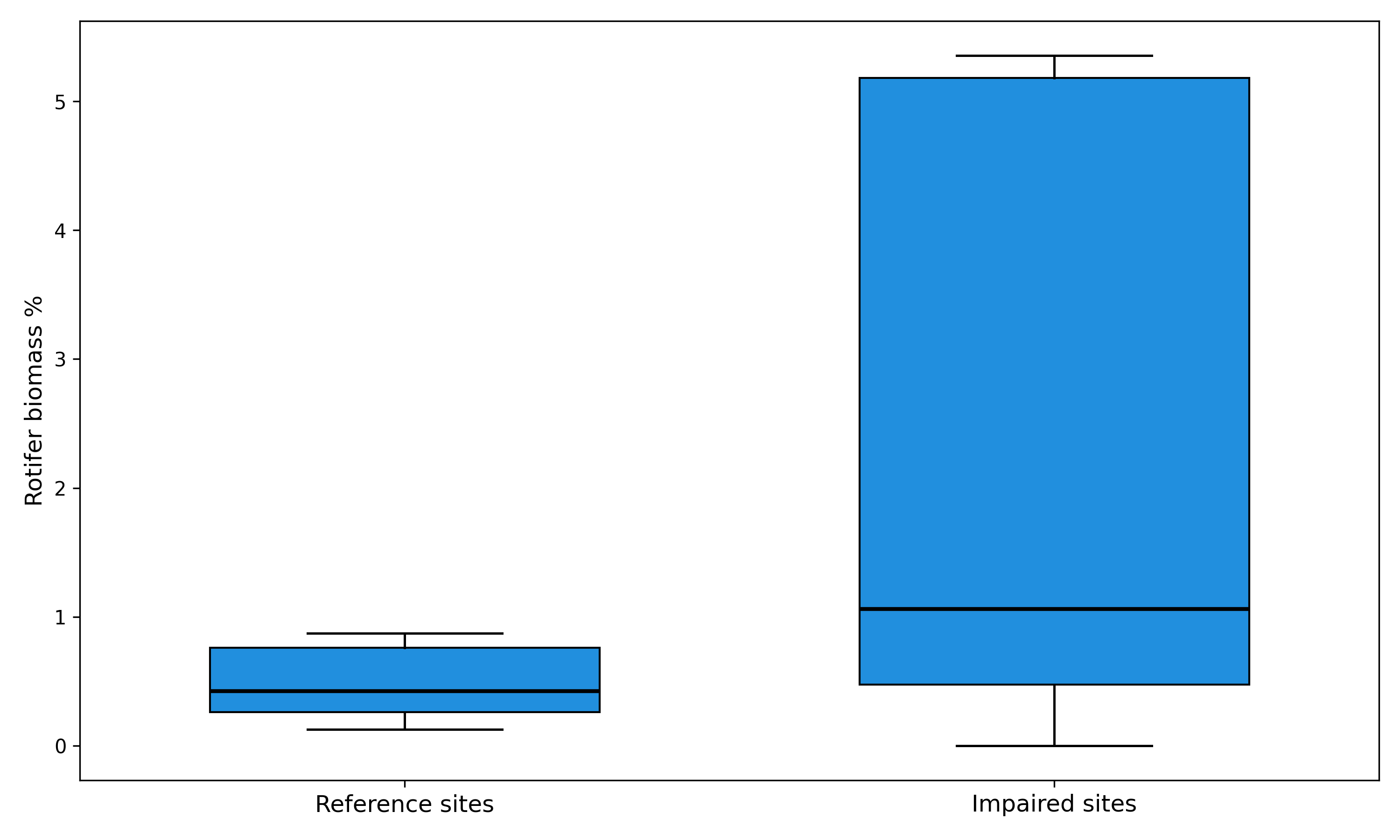

Supplement: Supplementary file 1 [file biology-15-00765-s001.zip › Supplementary/File S7/zooplankton_2021_05/Z22_Rotifer biomass %.png]

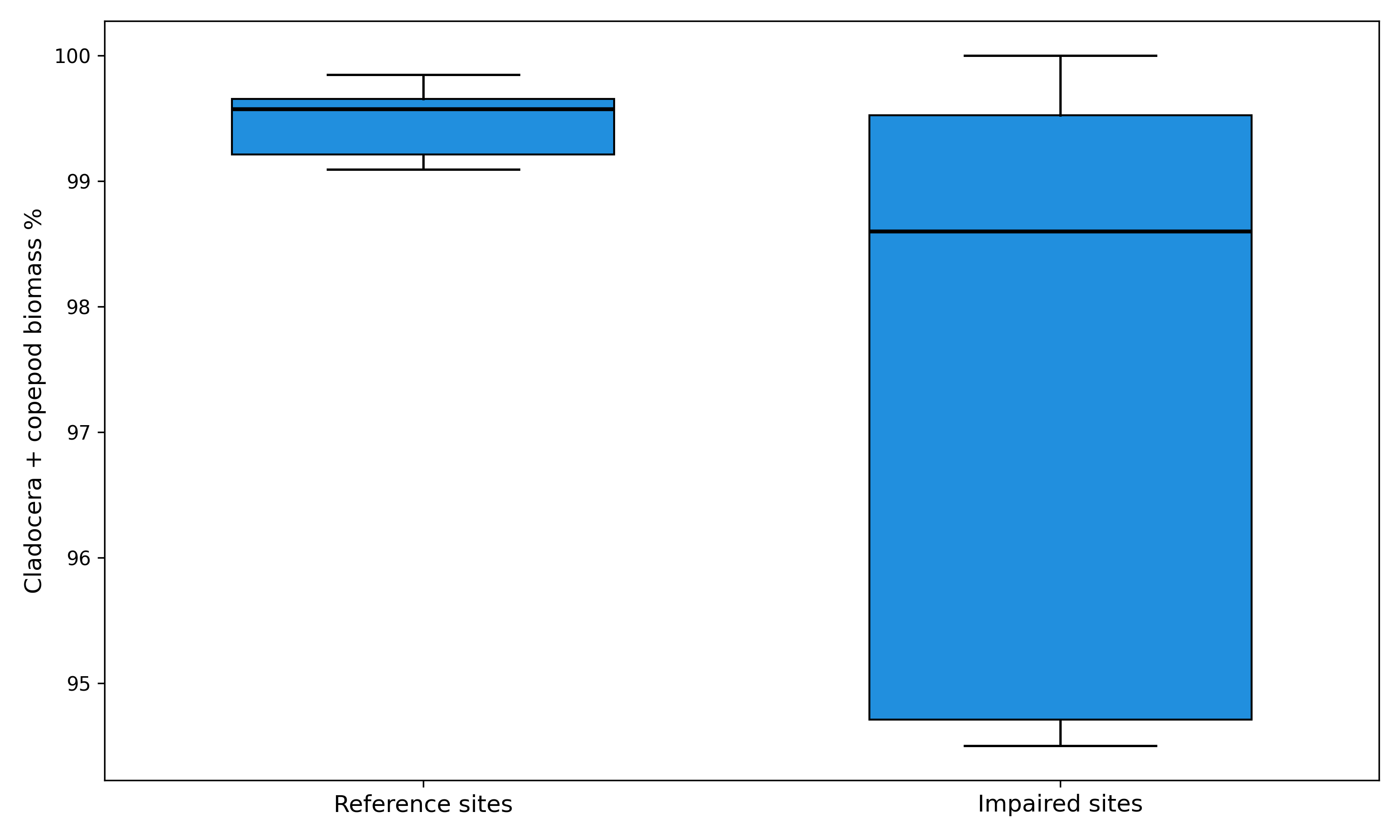

Supplement: Supplementary file 1 [file biology-15-00765-s001.zip › Supplementary/File S7/zooplankton_2021_05/Z25_Cladocera + copepod biomass %.png]

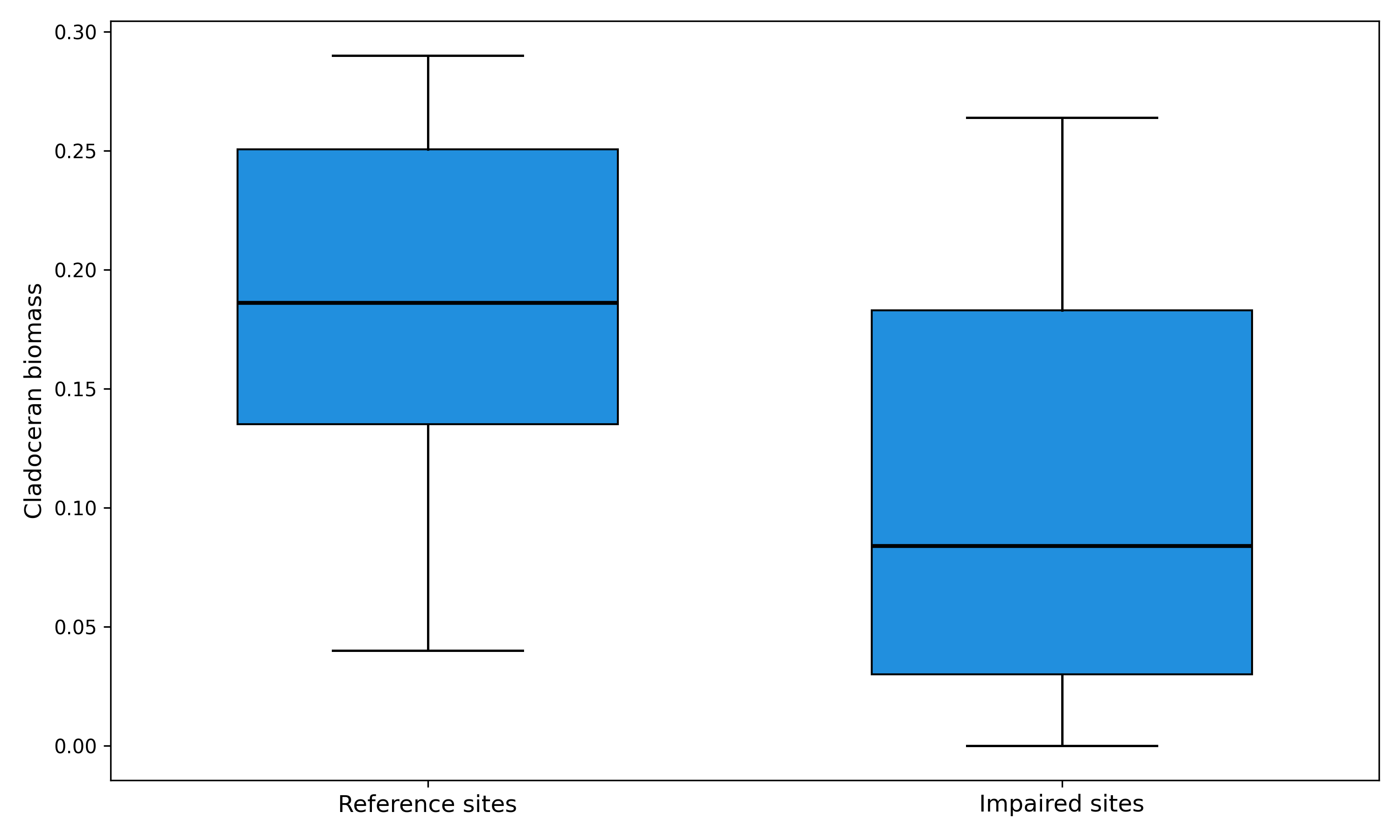

Supplement: Supplementary file 1 [file biology-15-00765-s001.zip › Supplementary/File S7/zooplankton_2021_09/Z14_Cladoceran biomass.png]

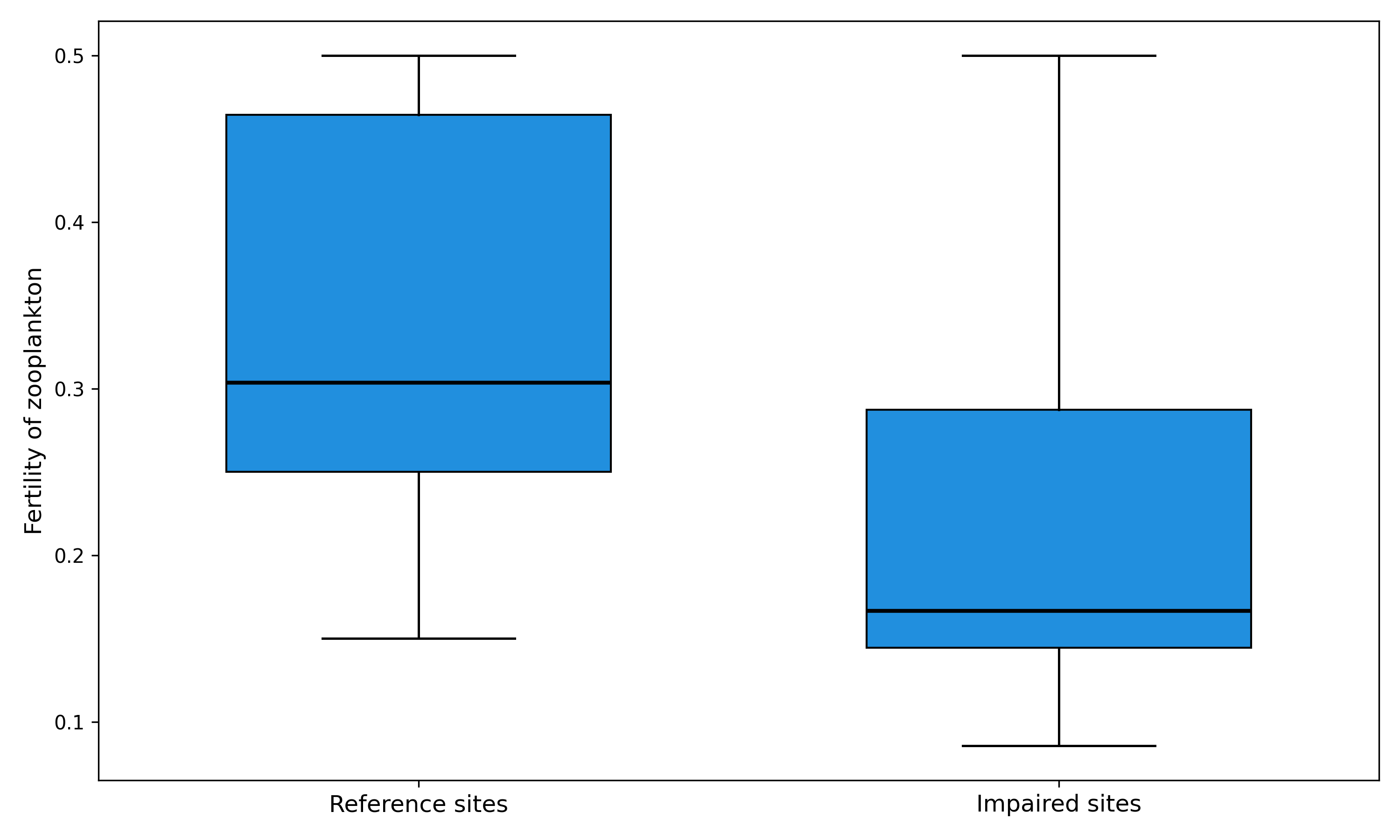

Supplement: Supplementary file 1 [file biology-15-00765-s001.zip › Supplementary/File S7/zooplankton_2021_09/Z35_Fertility of zooplankton.png]

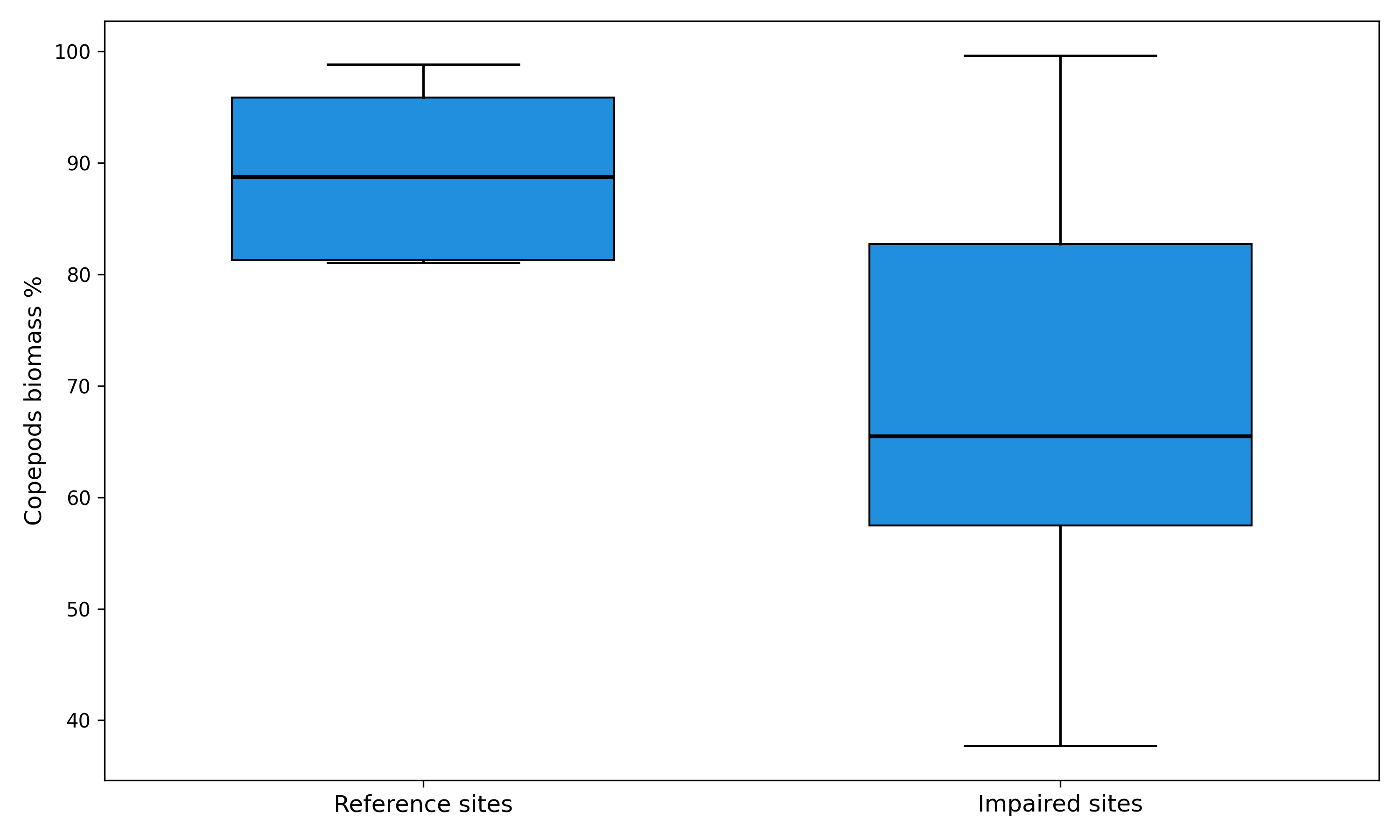

Supplement: Supplementary file 1 [file biology-15-00765-s001.zip › Supplementary/File S7/zooplankton_2022_05/Z24_Copepods biomass %.png]

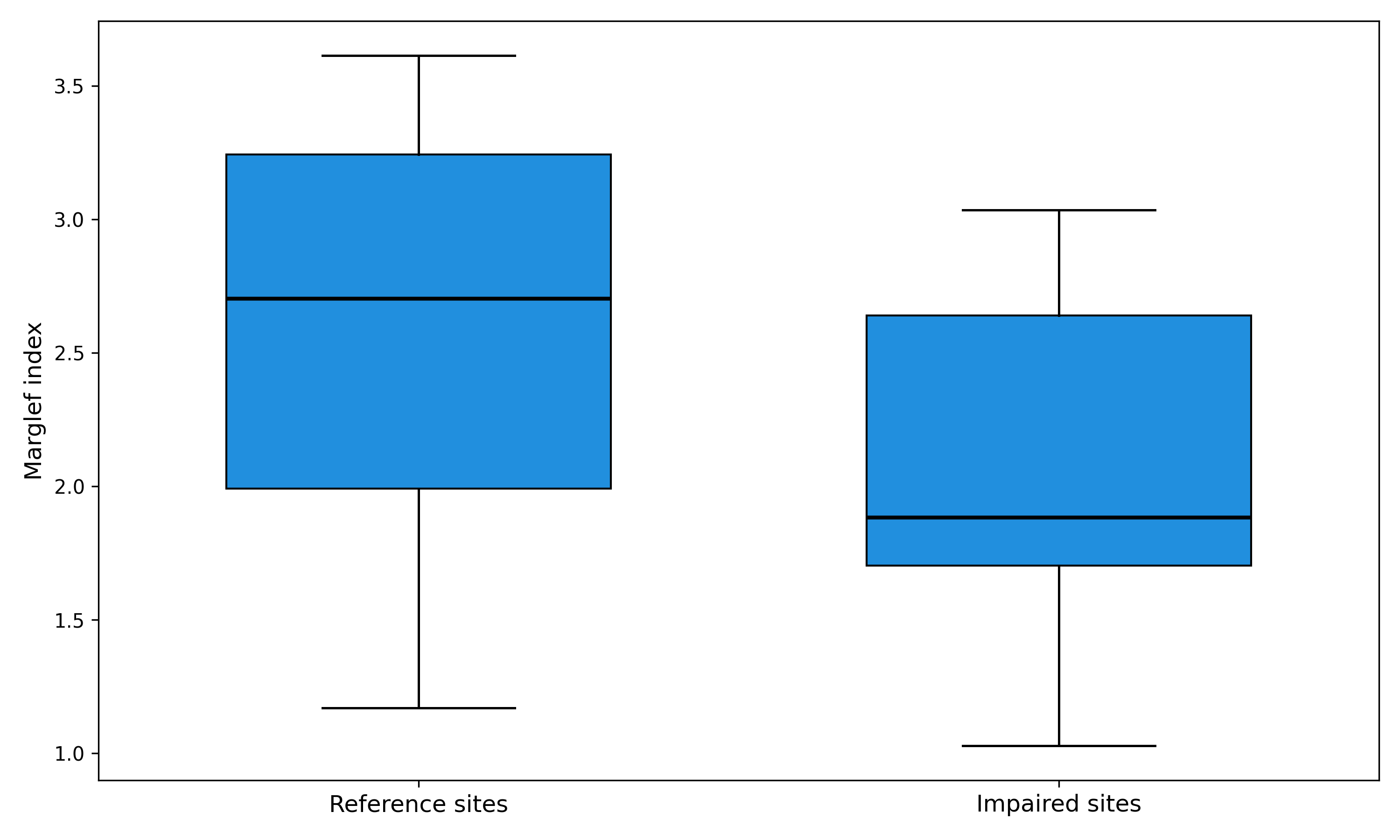

Supplement: Supplementary file 1 [file biology-15-00765-s001.zip › Supplementary/File S7/zooplankton_2022_05/Z28_Marglef index.png]

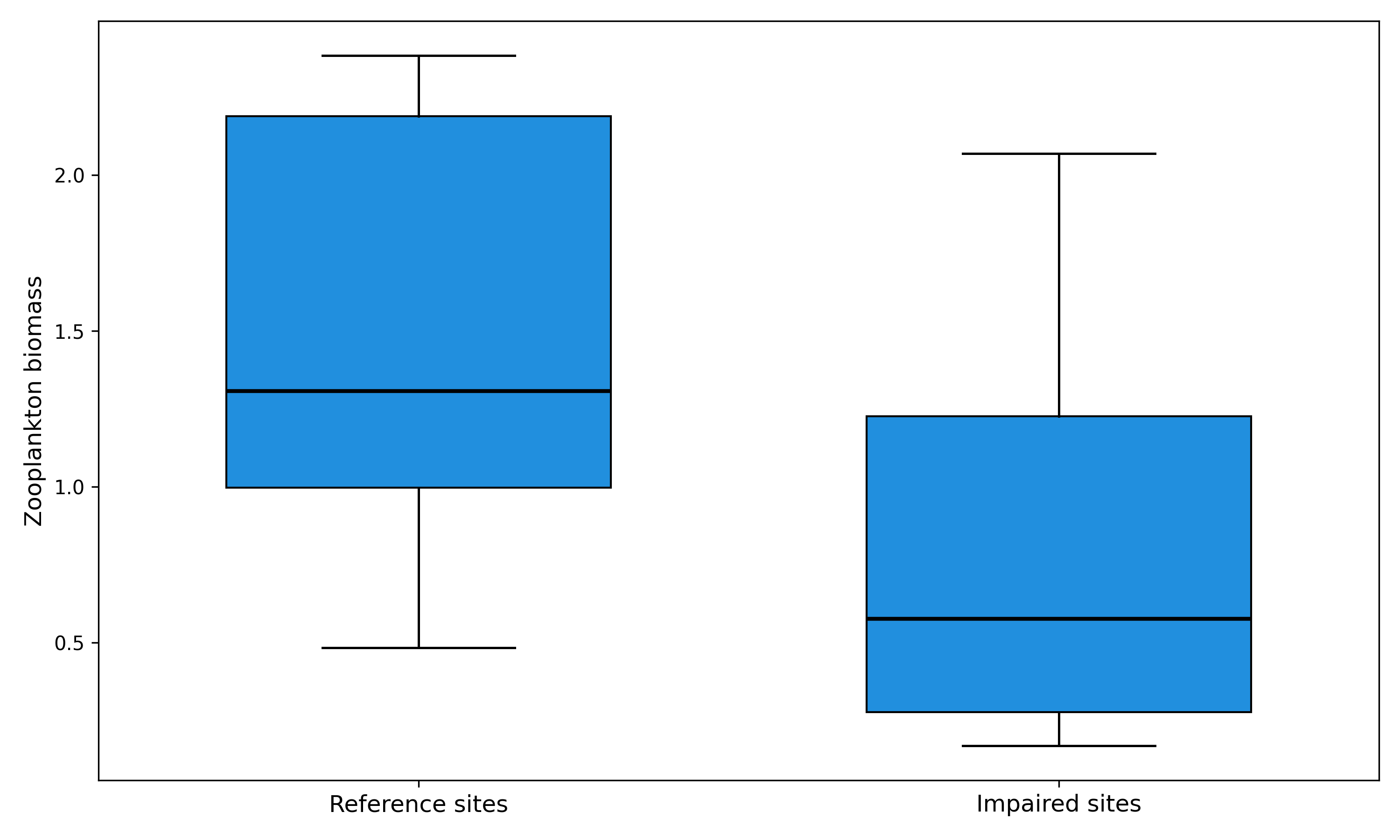

Supplement: Supplementary file 1 [file biology-15-00765-s001.zip › Supplementary/File S7/zooplankton_2022_09/Z12_Zooplankton biomass.png]

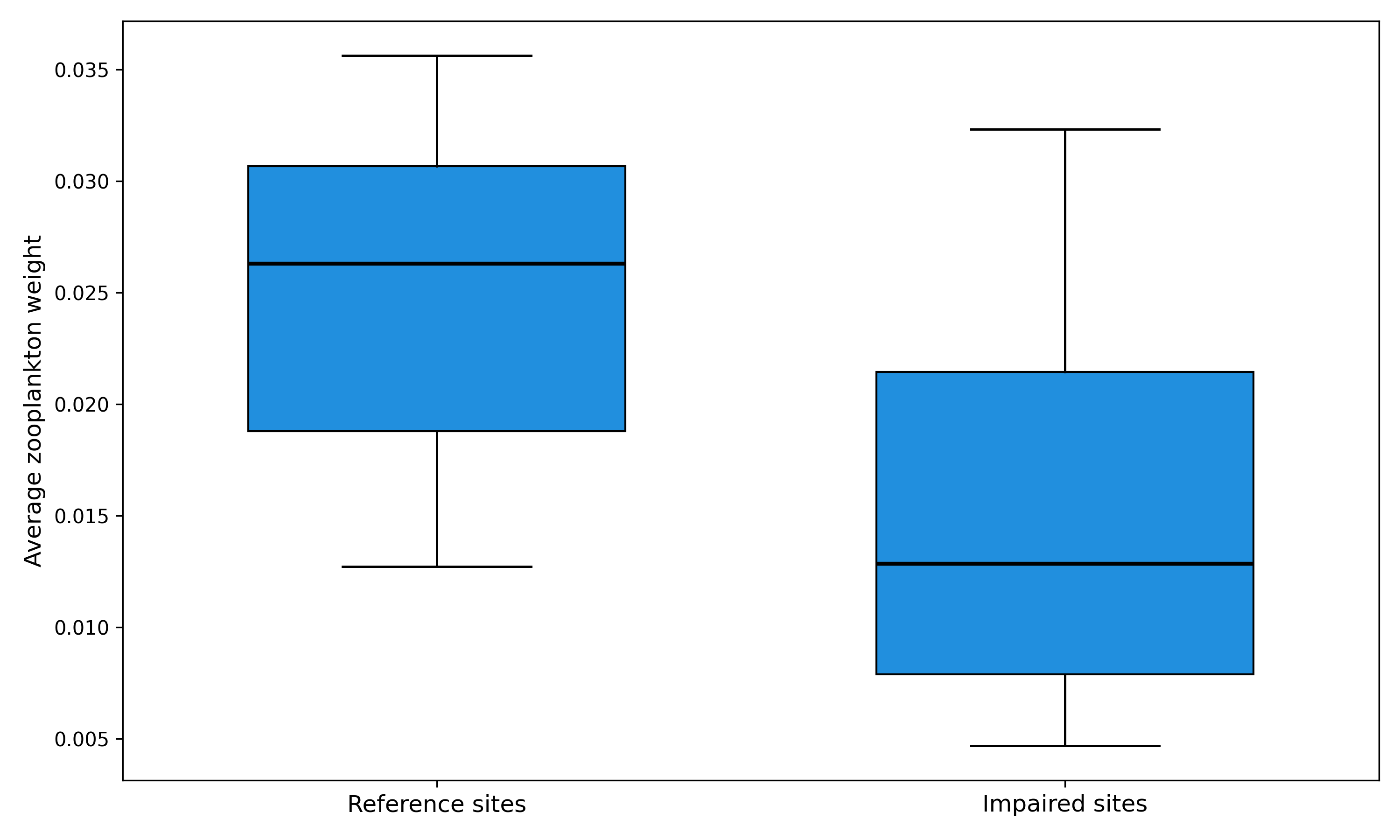

Supplement: Supplementary file 1 [file biology-15-00765-s001.zip › Supplementary/File S7/zooplankton_2022_09/Z16_Average zooplankton weight.png]

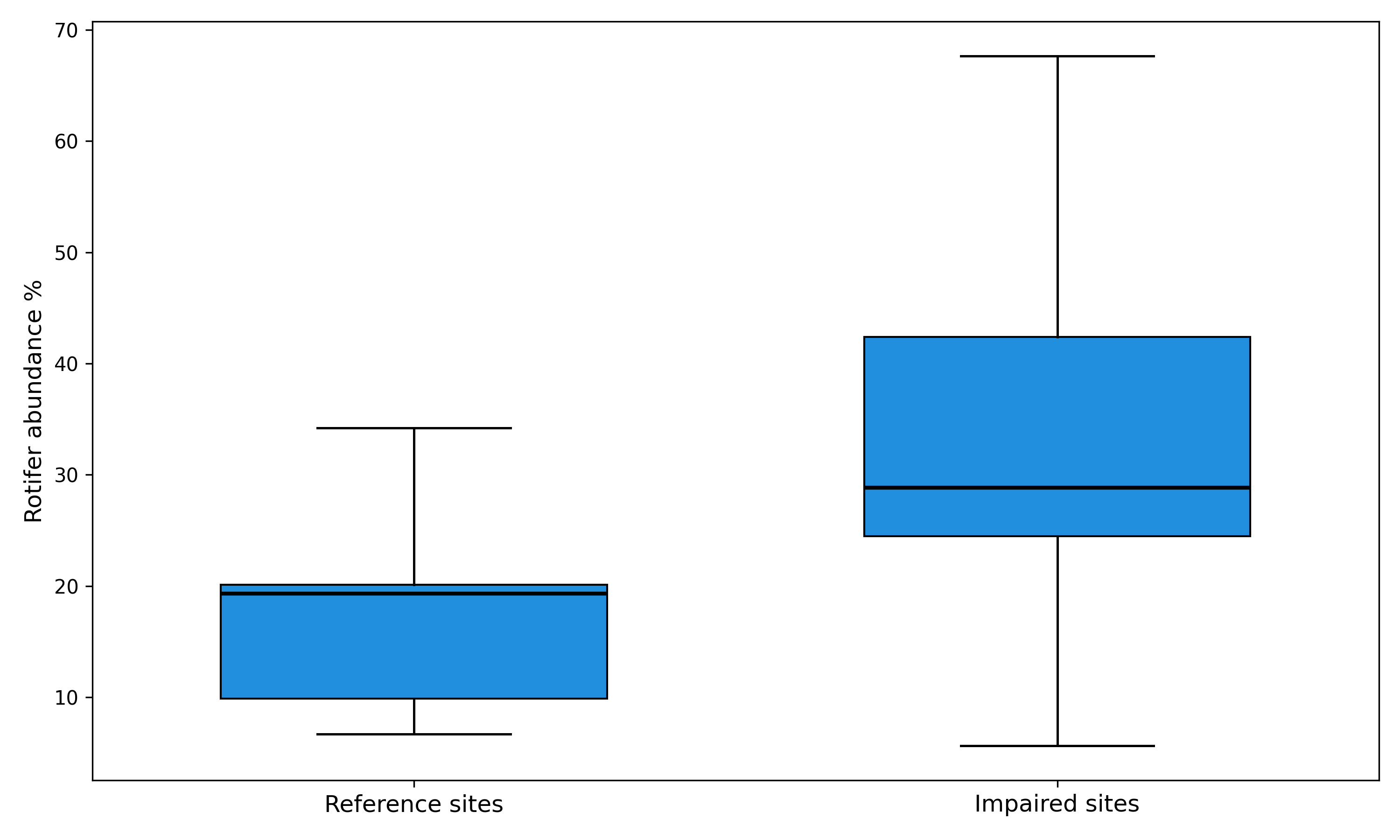

Supplement: Supplementary file 1 [file biology-15-00765-s001.zip › Supplementary/File S7/zooplankton_2022_09/Z18_Rotifer abundance %.png]

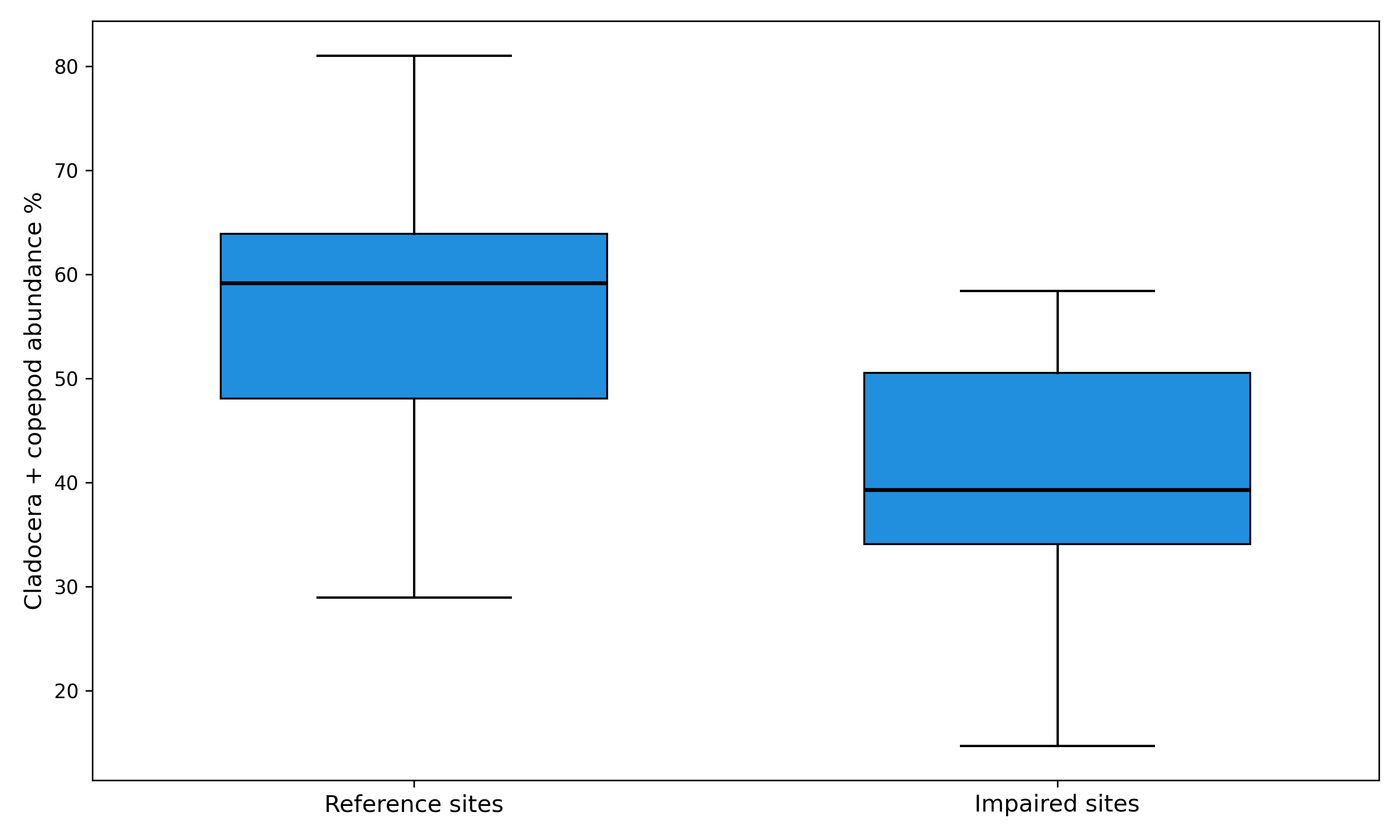

Supplement: Supplementary file 1 [file biology-15-00765-s001.zip › Supplementary/File S7/zooplankton_2022_09/Z21_Cladocera + copepod abundance %.png]

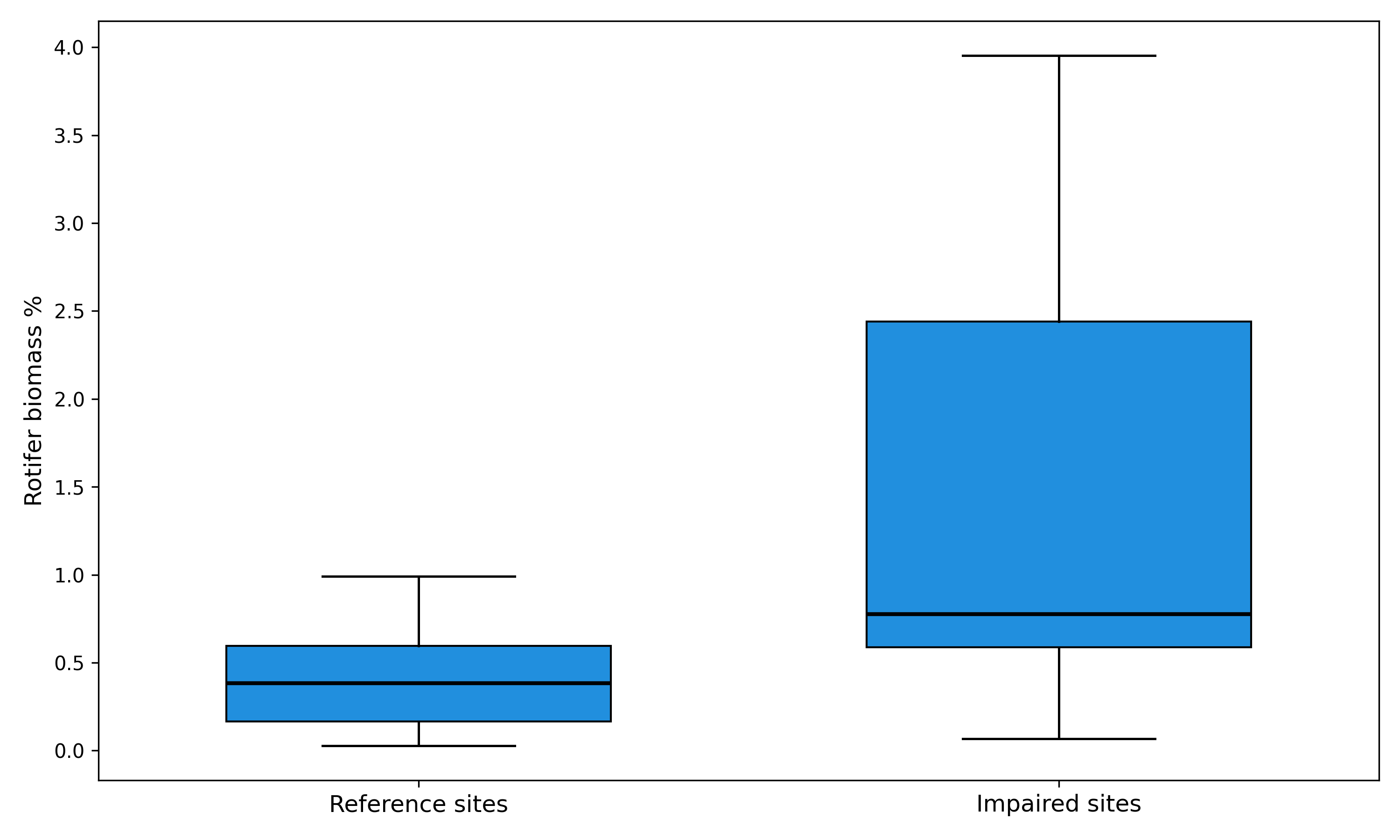

Supplement: Supplementary file 1 [file biology-15-00765-s001.zip › Supplementary/File S7/zooplankton_2022_09/Z22_Rotifer biomass %.png]

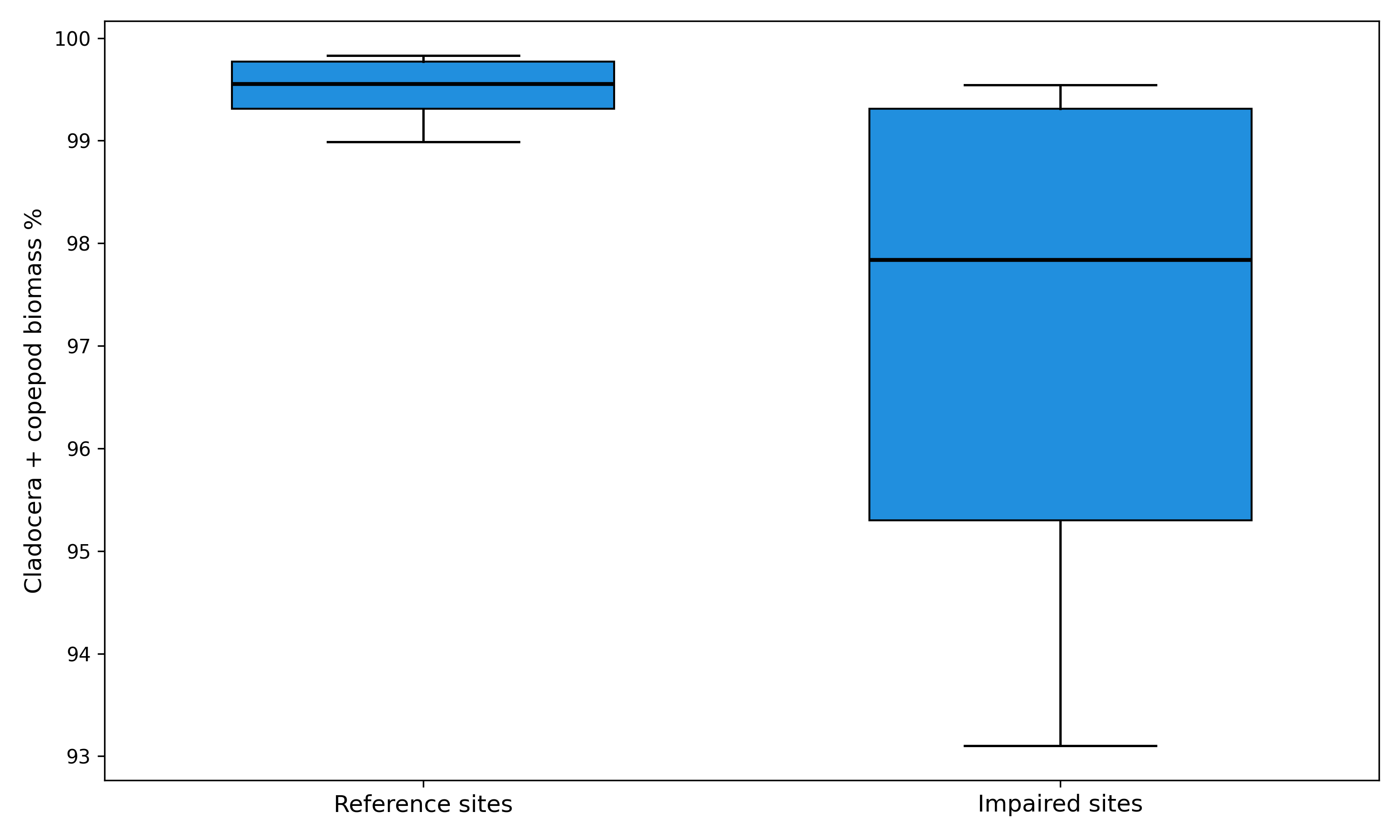

Supplement: Supplementary file 1 [file biology-15-00765-s001.zip › Supplementary/File S7/zooplankton_2022_09/Z25_Cladocera + copepod biomass %.png]

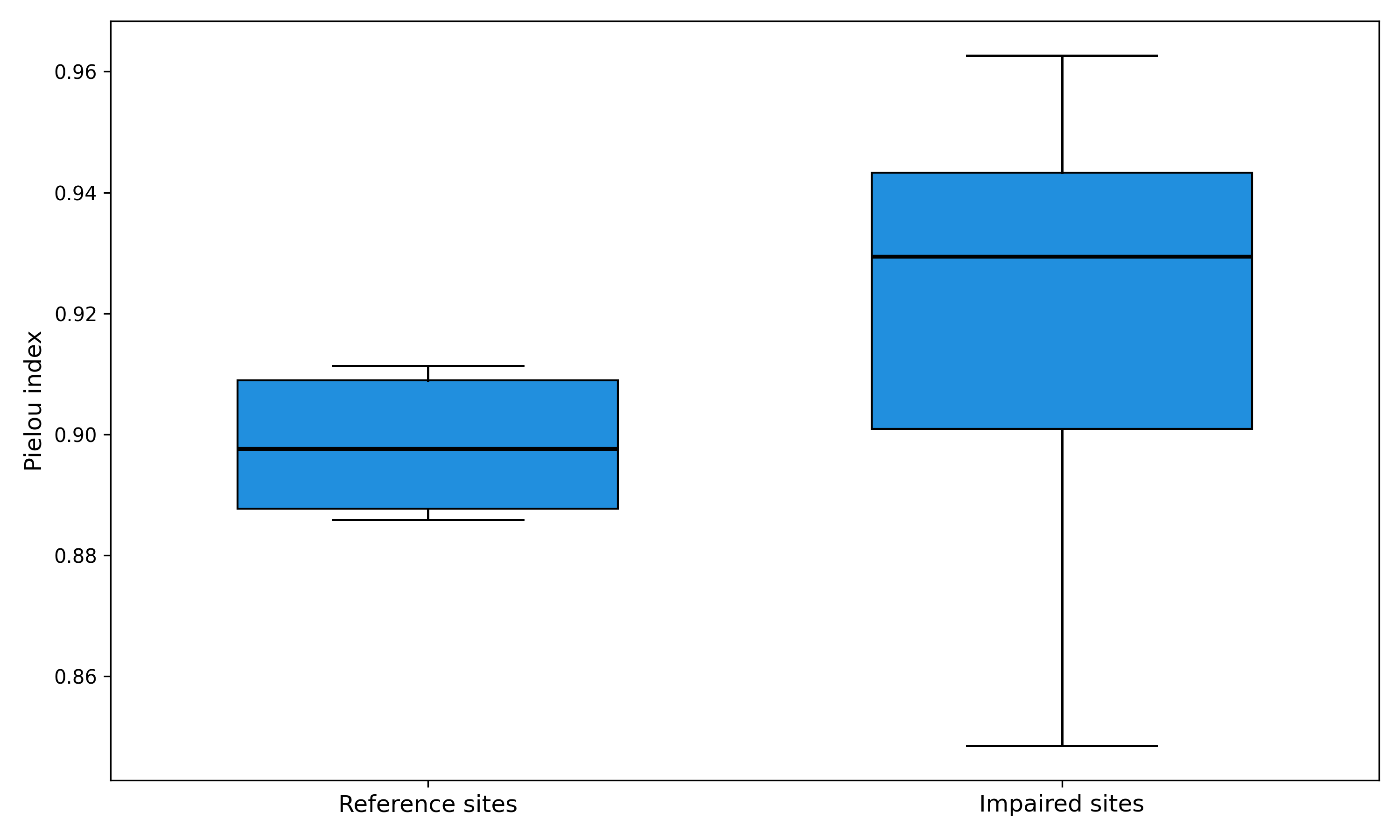

Supplement: Supplementary file 1 [file biology-15-00765-s001.zip › Supplementary/File S7/zooplankton_2022_09/Z30_Pielou index.png]

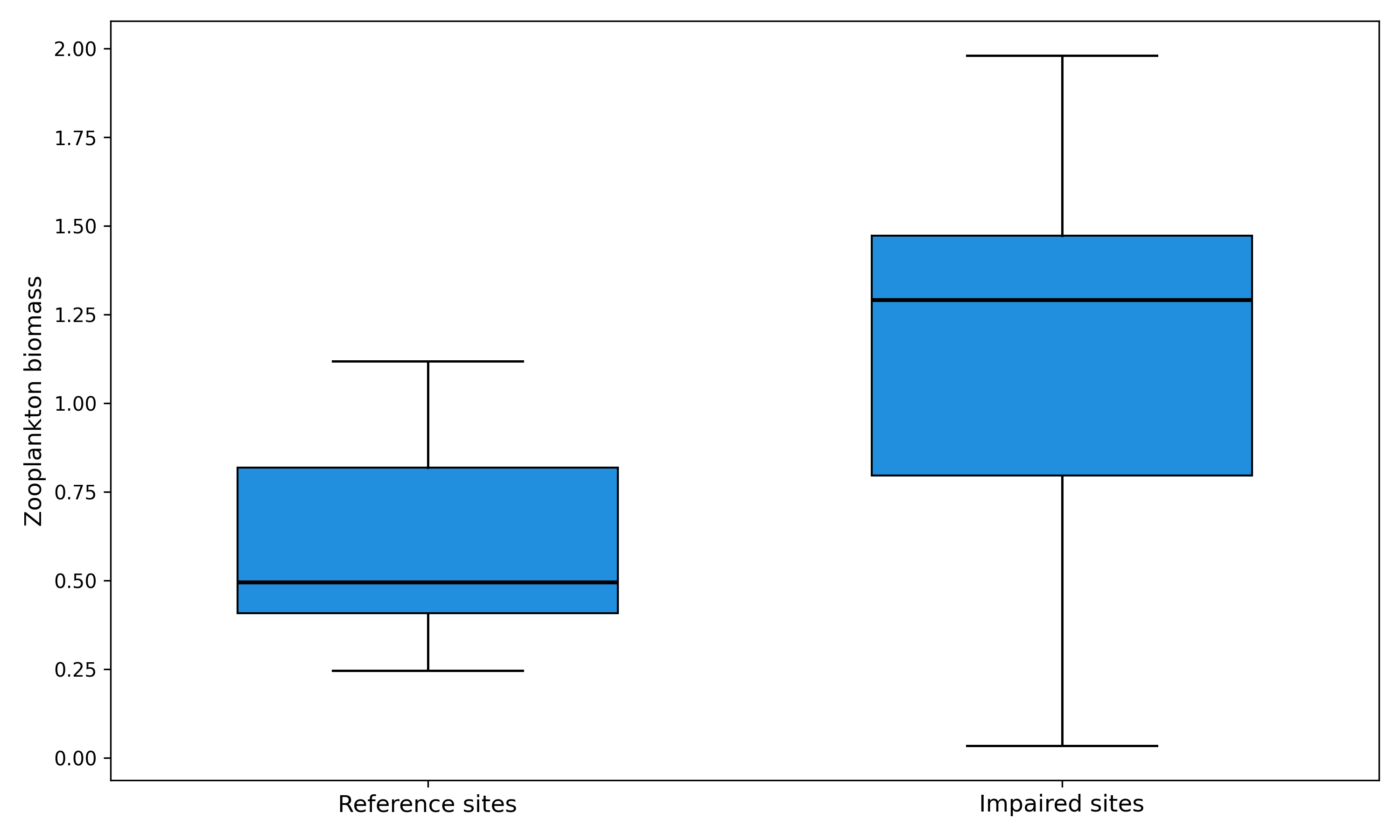

Supplement: Supplementary file 1 [file biology-15-00765-s001.zip › Supplementary/File S7/zooplankton_2023_05/Z12_Zooplankton biomass.png]

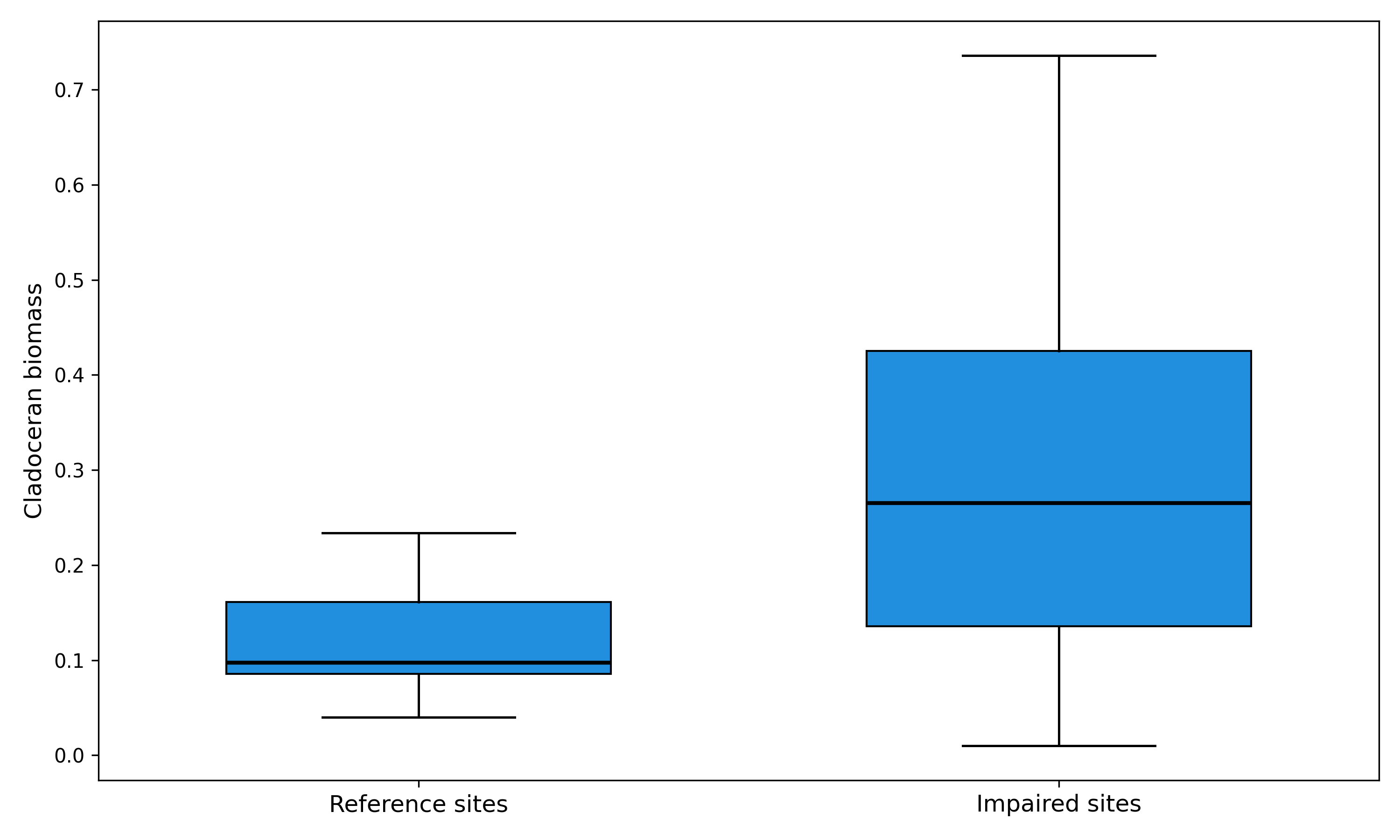

Supplement: Supplementary file 1 [file biology-15-00765-s001.zip › Supplementary/File S7/zooplankton_2023_05/Z14_Cladoceran biomass.png]

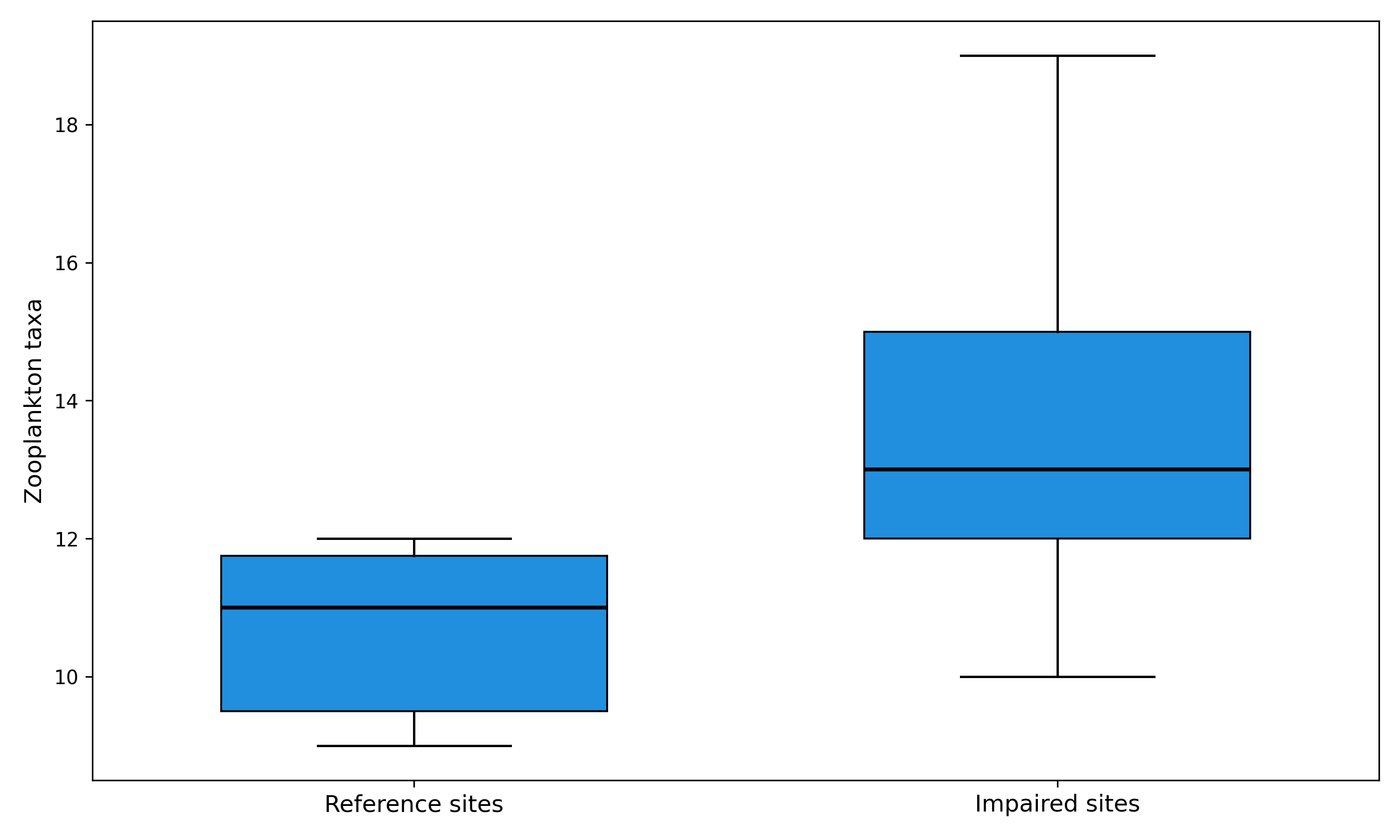

Supplement: Supplementary file 1 [file biology-15-00765-s001.zip › Supplementary/File S7/zooplankton_2023_05/Z1_Zooplankton taxa.png]

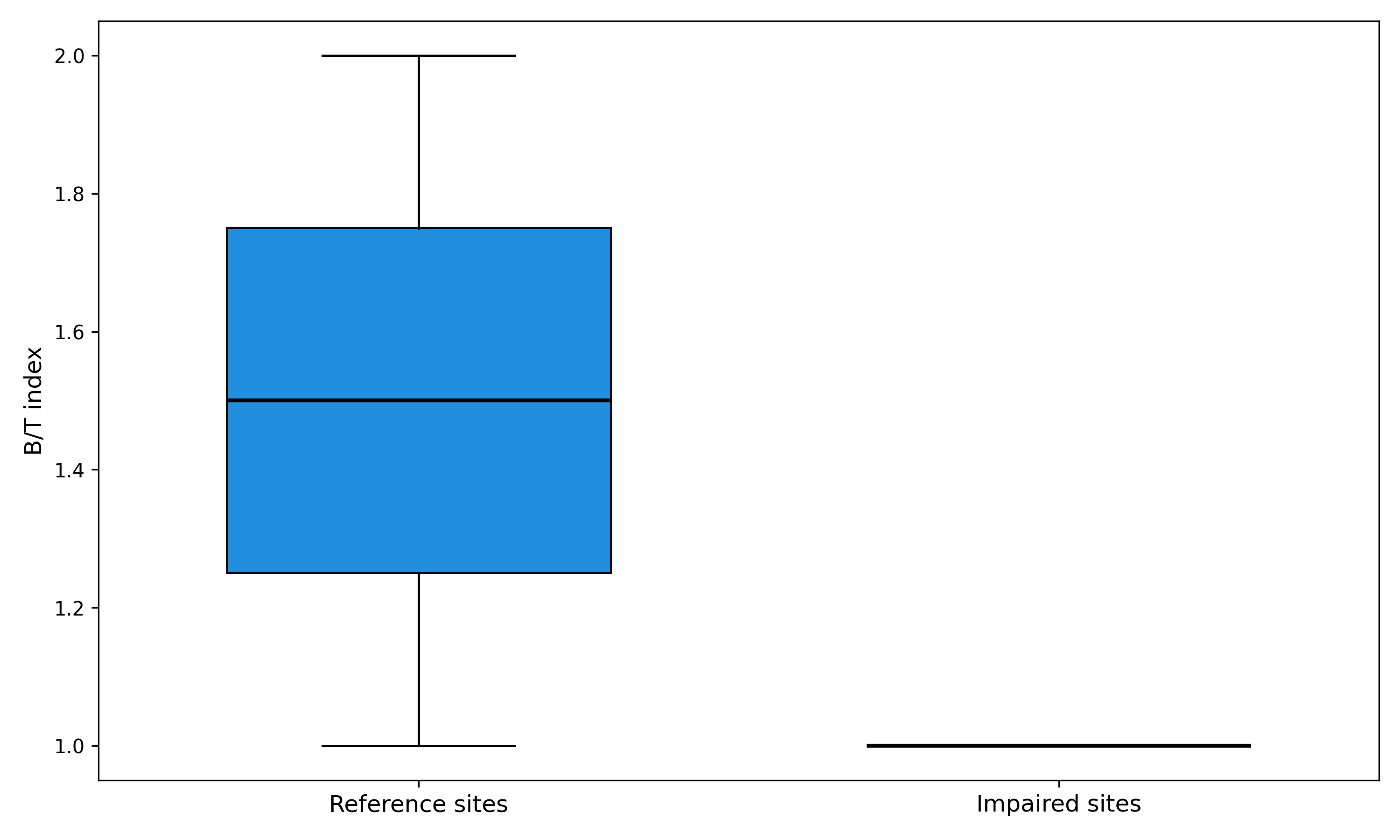

Supplement: Supplementary file 1 [file biology-15-00765-s001.zip › Supplementary/File S7/zooplankton_2023_05/Z33_B_T index.png]

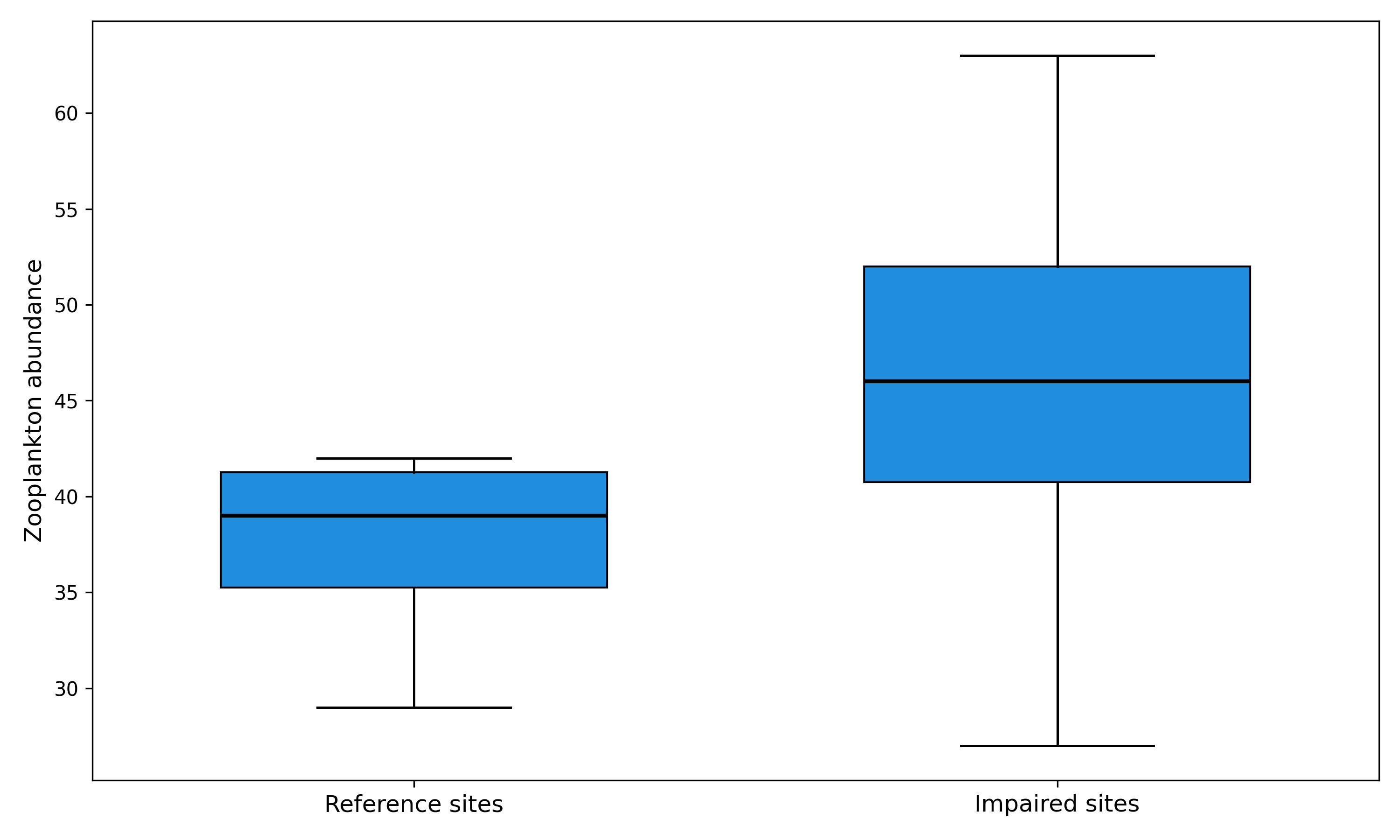

Supplement: Supplementary file 1 [file biology-15-00765-s001.zip › Supplementary/File S7/zooplankton_2023_05/Z7_Zooplankton abundance.png]

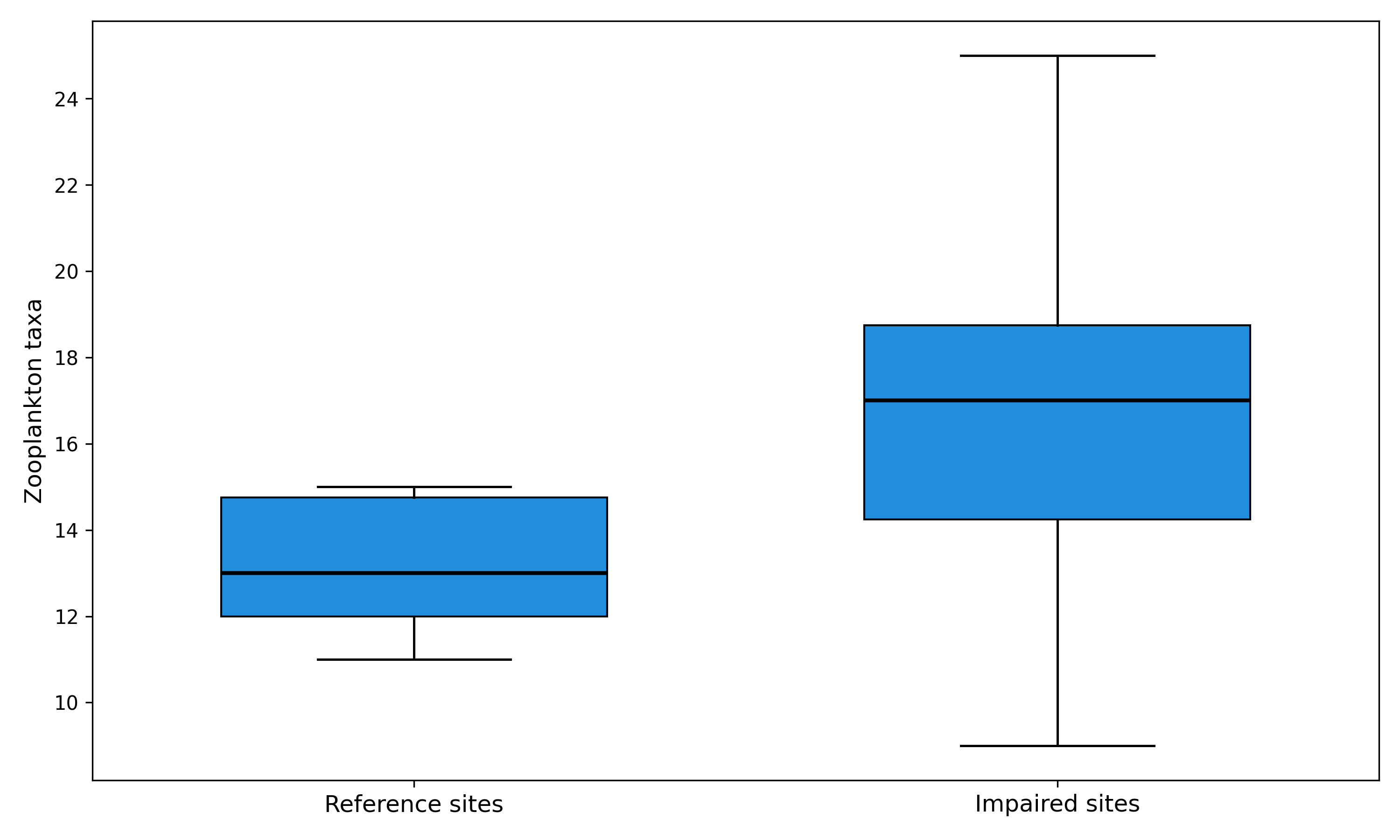

Supplement: Supplementary file 1 [file biology-15-00765-s001.zip › Supplementary/File S7/zooplankton_2023_09/Z1_Zooplankton taxa.png]

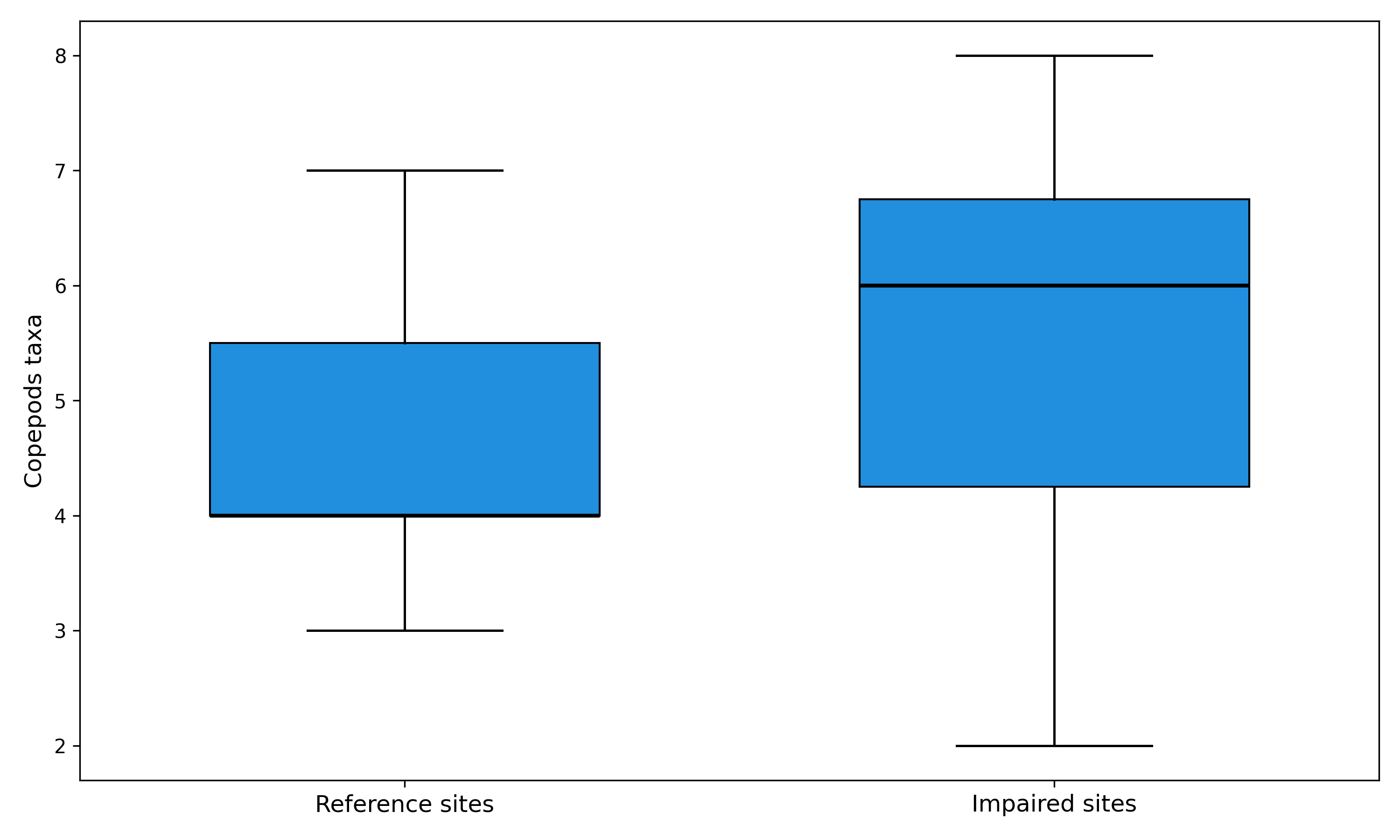

Supplement: Supplementary file 1 [file biology-15-00765-s001.zip › Supplementary/File S7/zooplankton_2023_09/Z4_Copepods taxa.png]

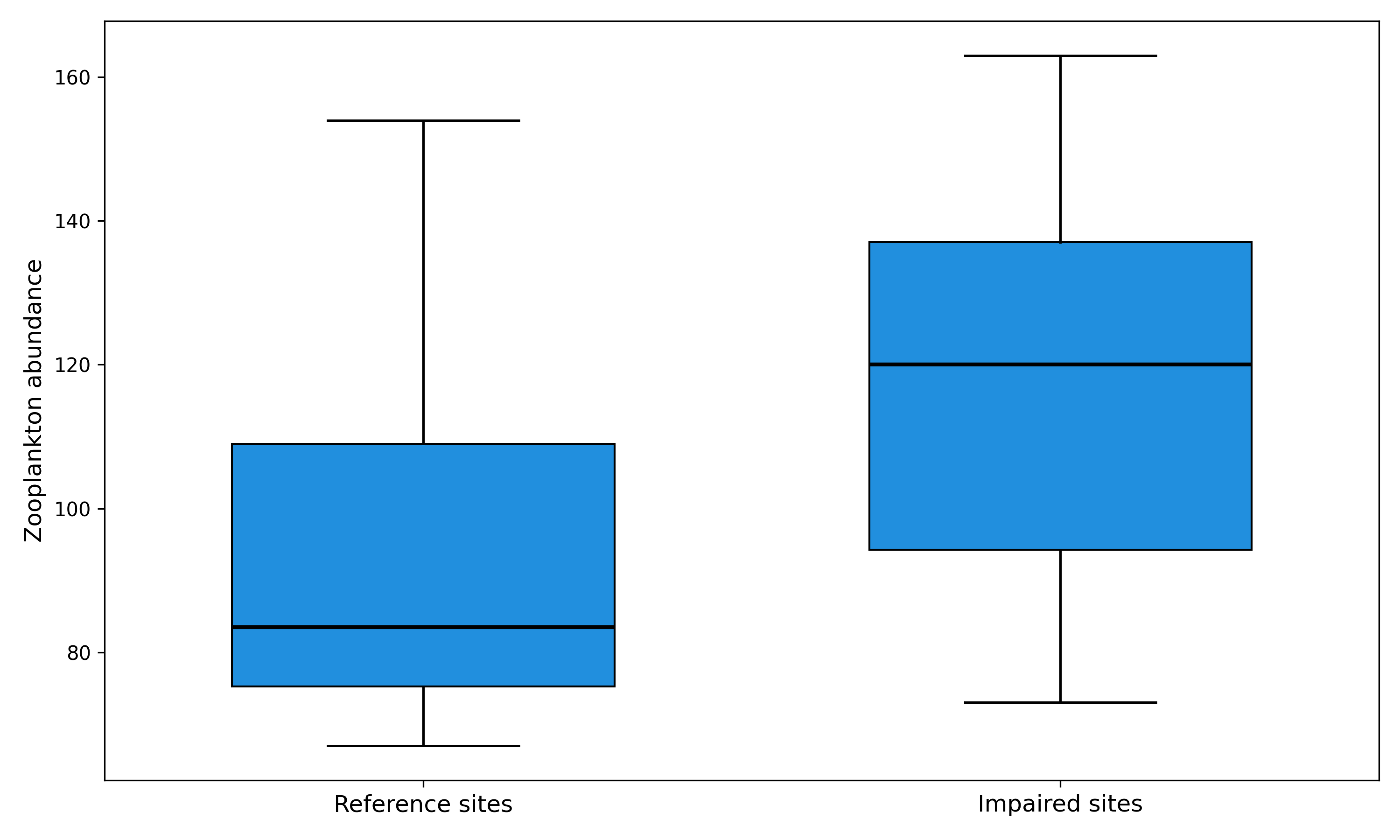

Supplement: Supplementary file 1 [file biology-15-00765-s001.zip › Supplementary/File S7/zooplankton_2023_09/Z7_Zooplankton abundance.png]

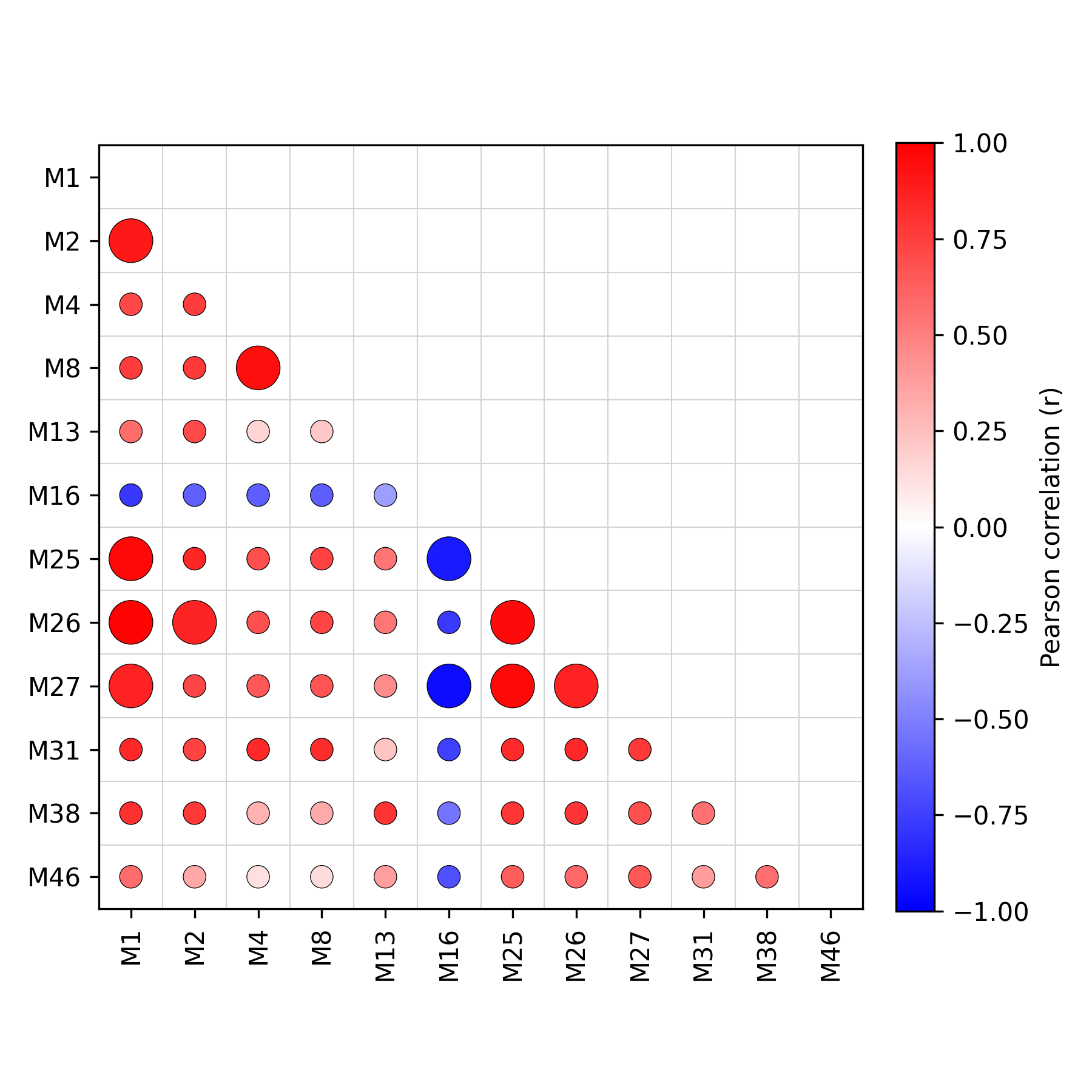

Supplement: Supplementary file 1 [file biology-15-00765-s001.zip › Supplementary/File S8/macroinvertebrates_2021_05.png]

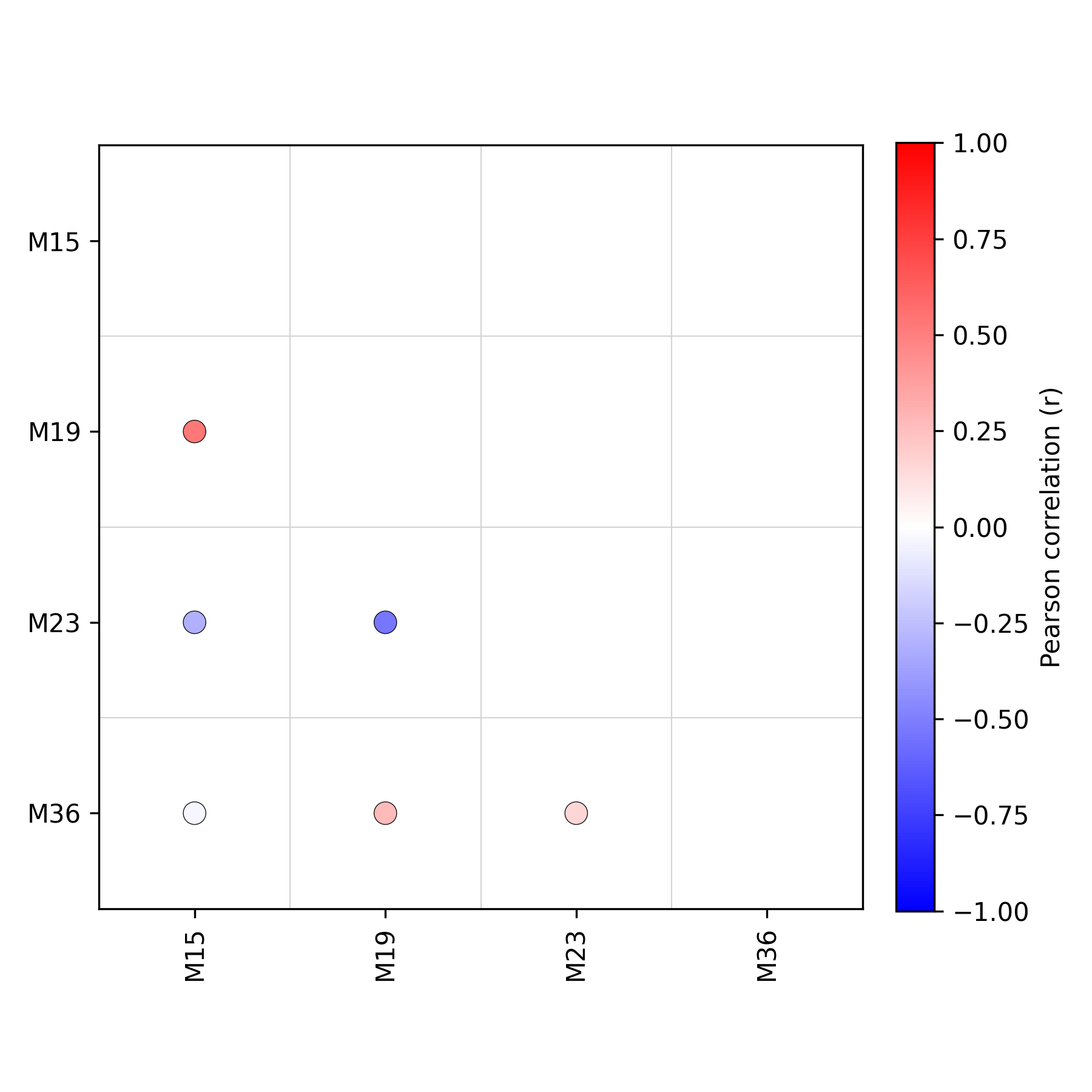

Supplement: Supplementary file 1 [file biology-15-00765-s001.zip › Supplementary/File S8/macroinvertebrates_2021_09.png]

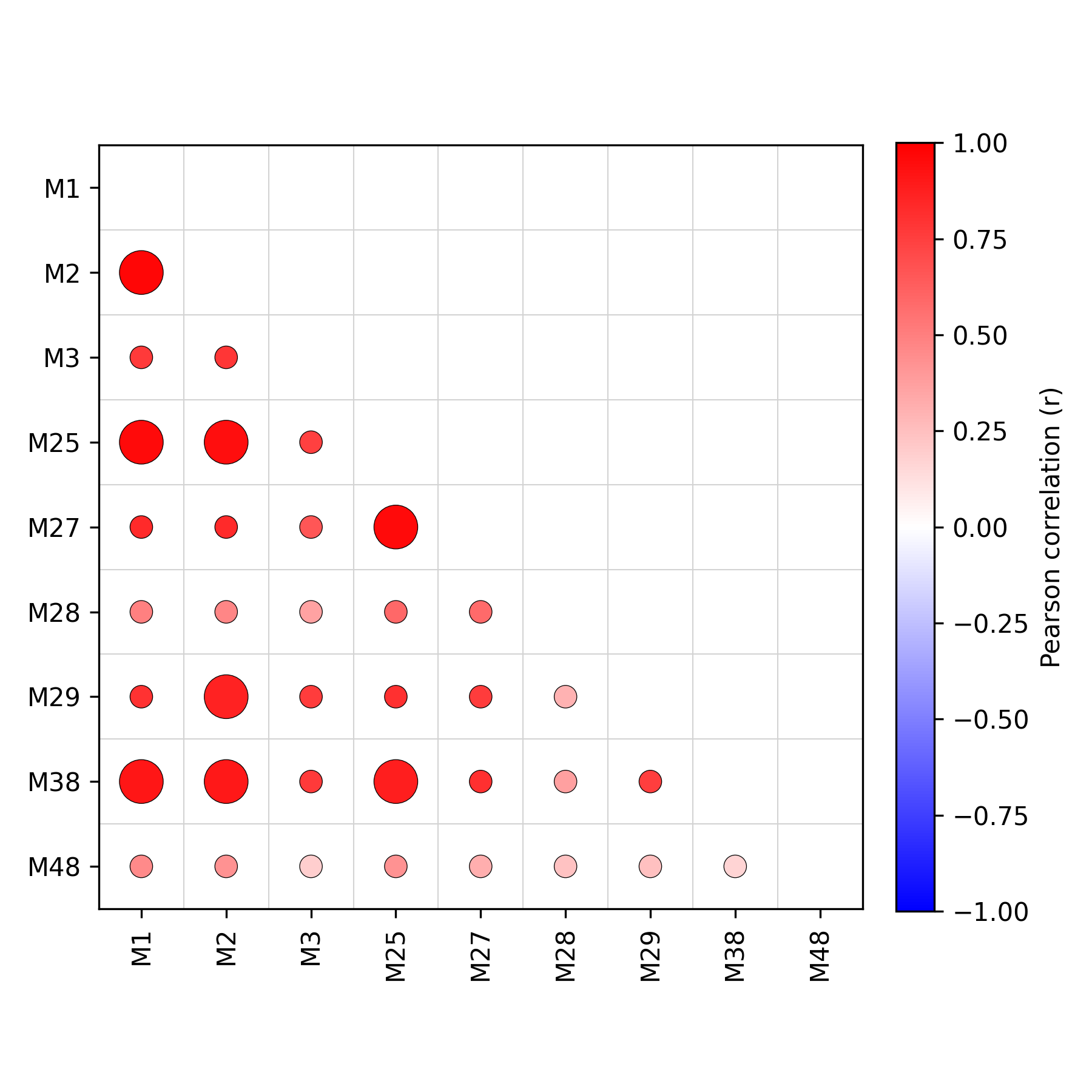

Supplement: Supplementary file 1 [file biology-15-00765-s001.zip › Supplementary/File S8/macroinvertebrates_2022_05.png]

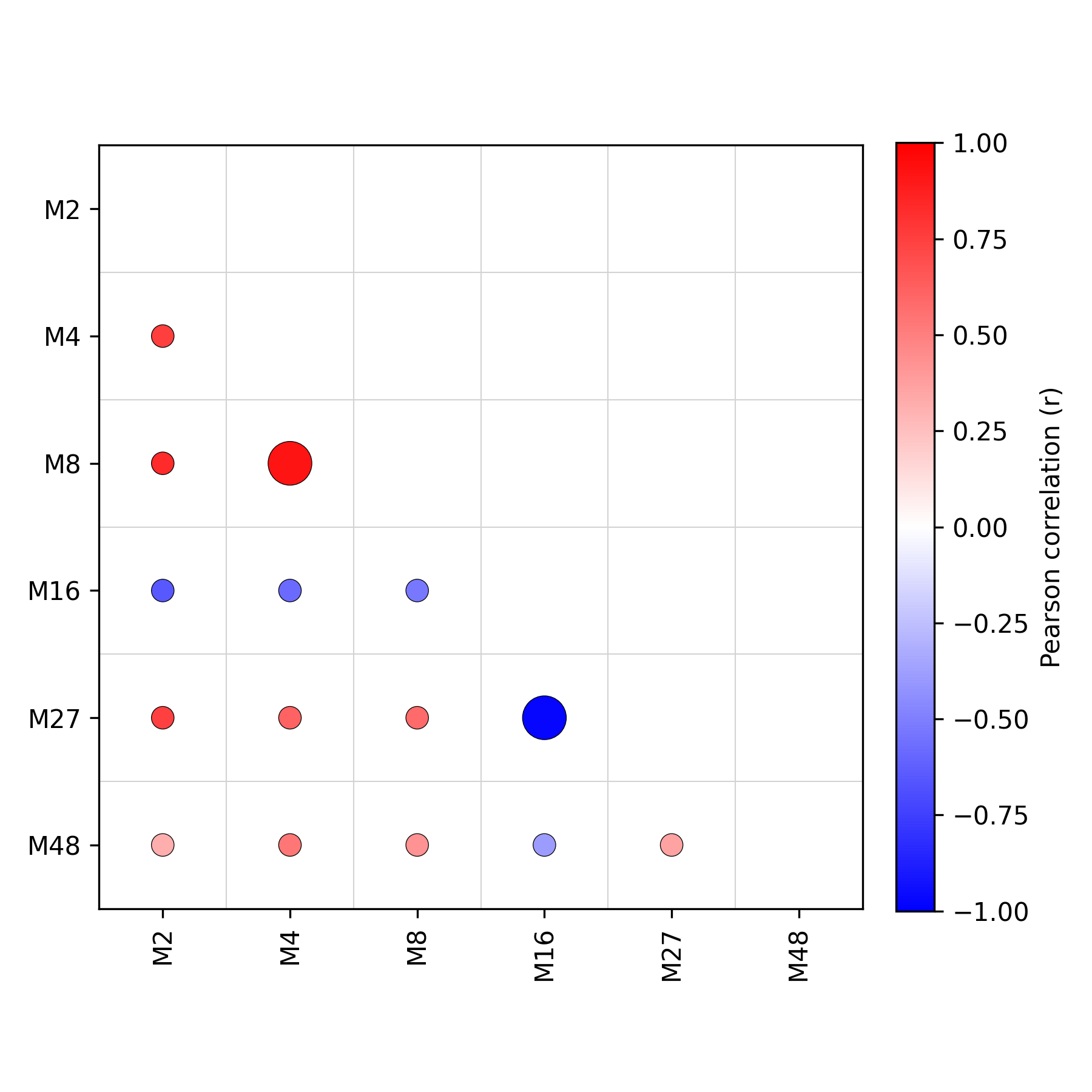

Supplement: Supplementary file 1 [file biology-15-00765-s001.zip › Supplementary/File S8/macroinvertebrates_2022_09.png]

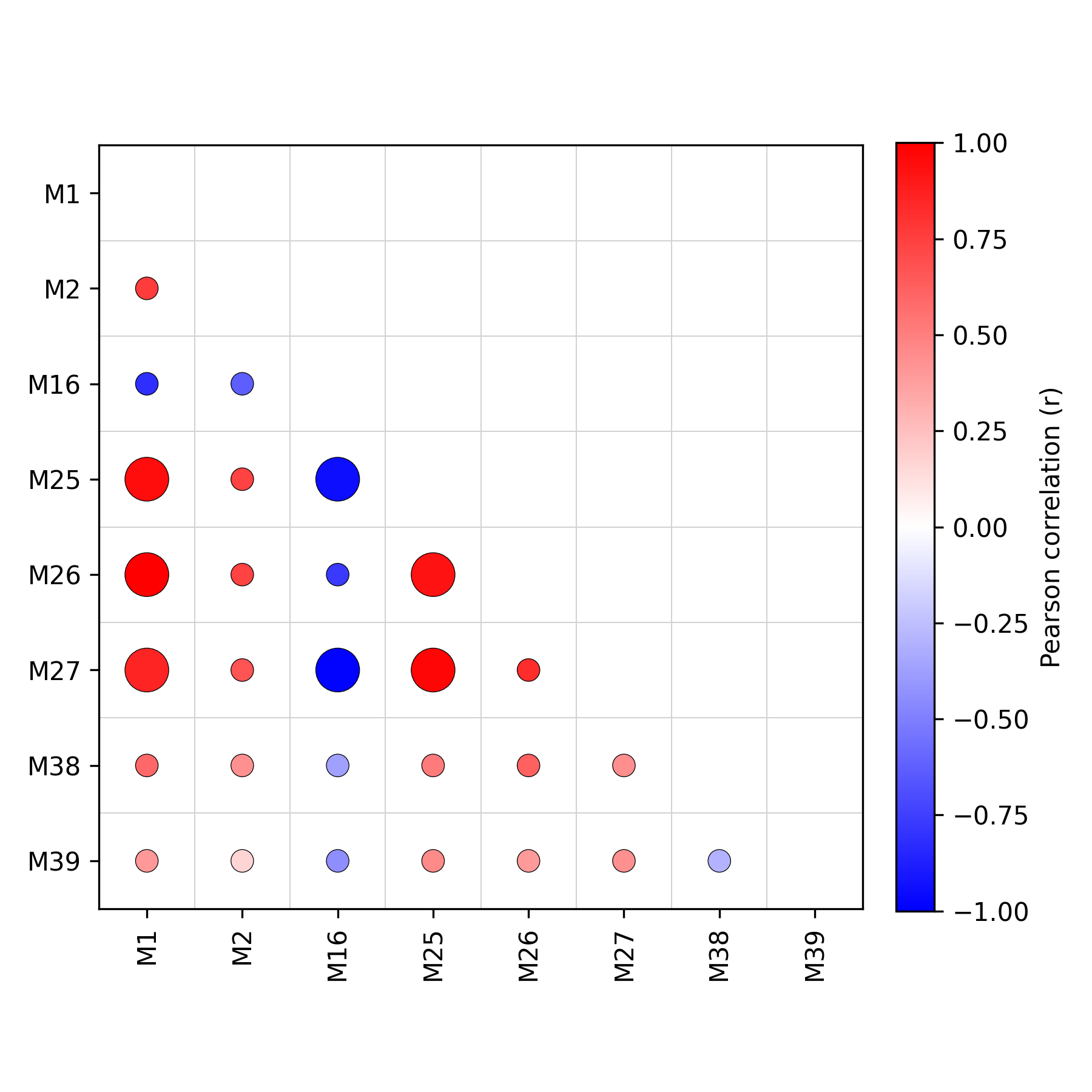

Supplement: Supplementary file 1 [file biology-15-00765-s001.zip › Supplementary/File S8/macroinvertebrates_2023_05.png]

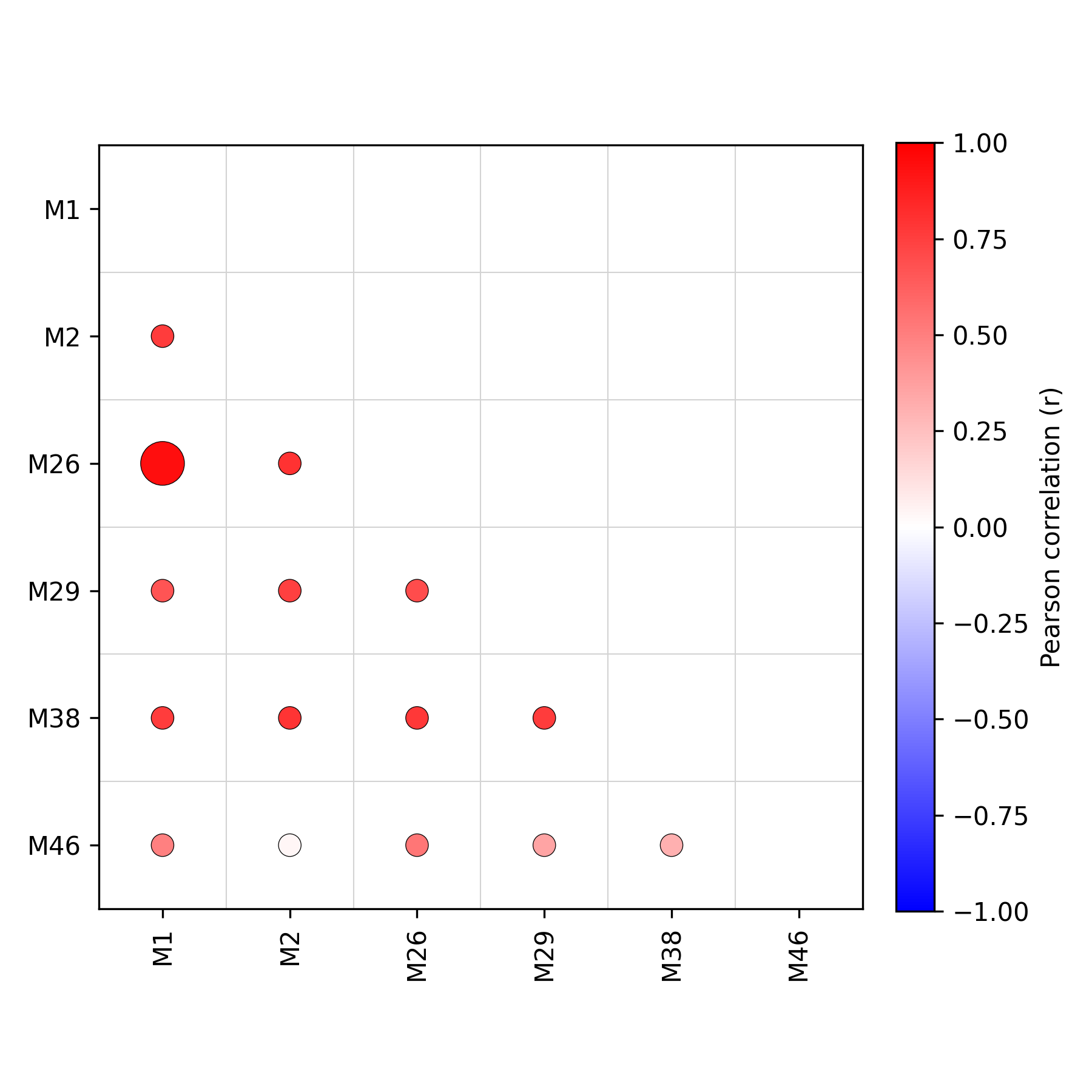

Supplement: Supplementary file 1 [file biology-15-00765-s001.zip › Supplementary/File S8/macroinvertebrates_2023_09.png]

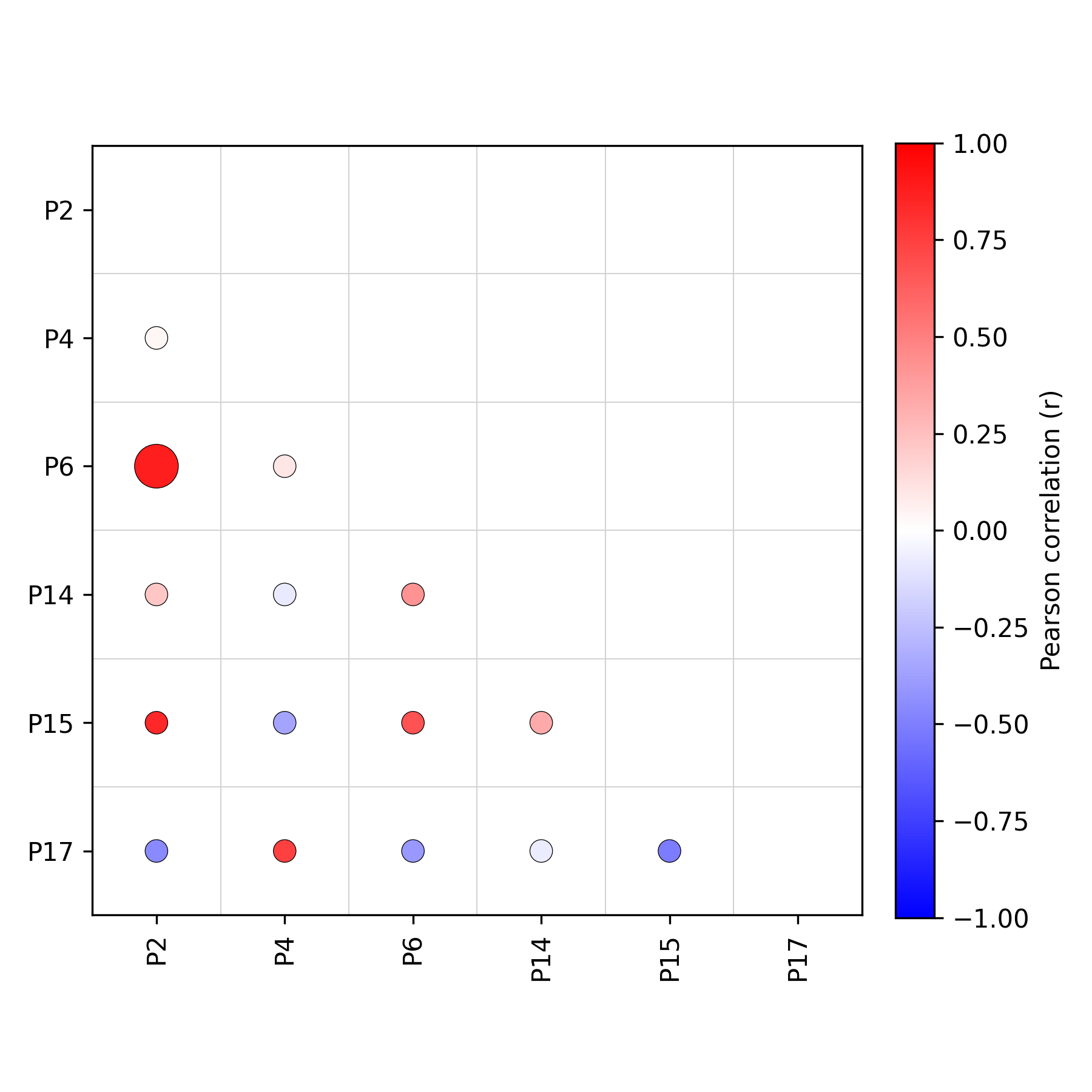

Supplement: Supplementary file 1 [file biology-15-00765-s001.zip › Supplementary/File S8/phytoplankton_2021_05.png]

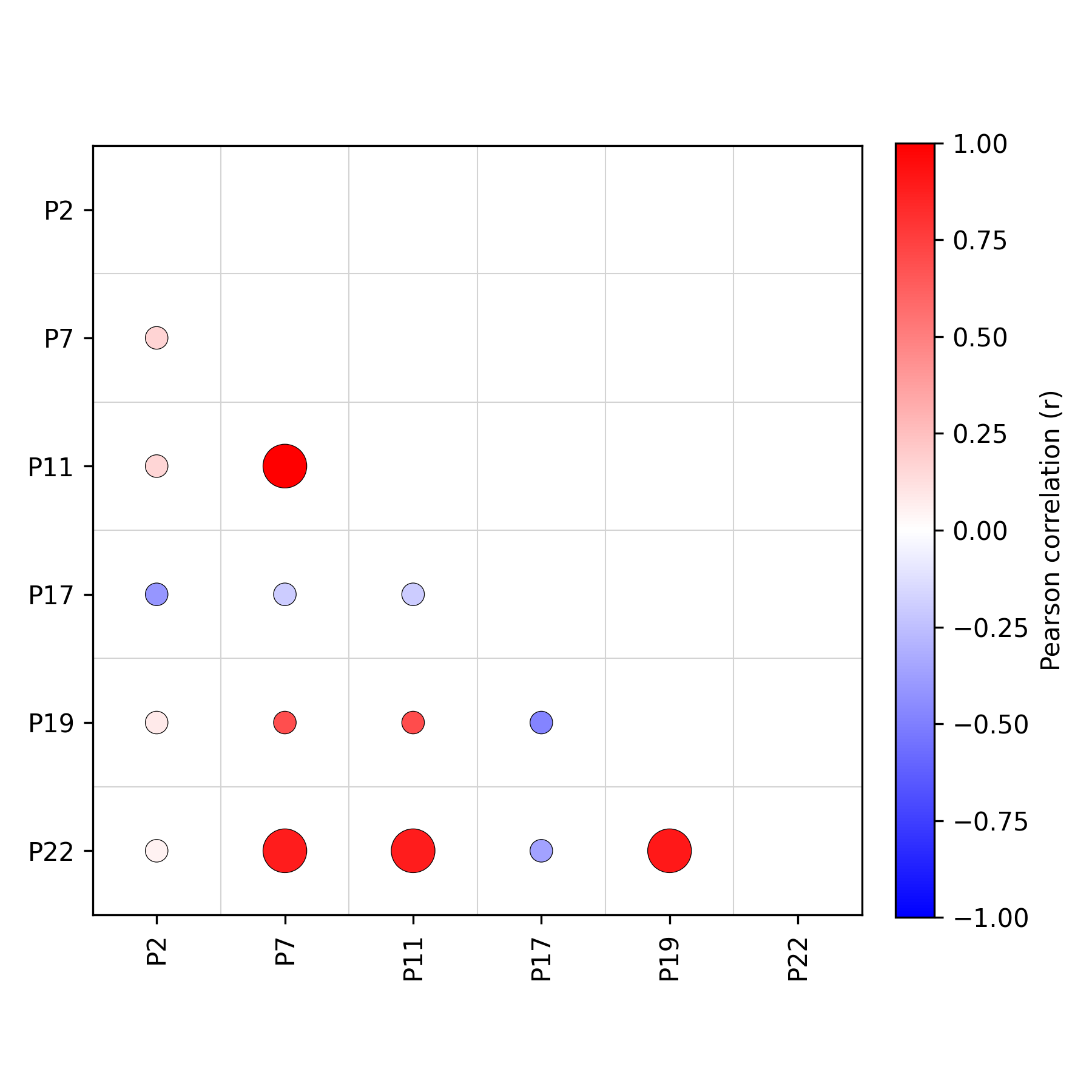

Supplement: Supplementary file 1 [file biology-15-00765-s001.zip › Supplementary/File S8/phytoplankton_2021_09.png]

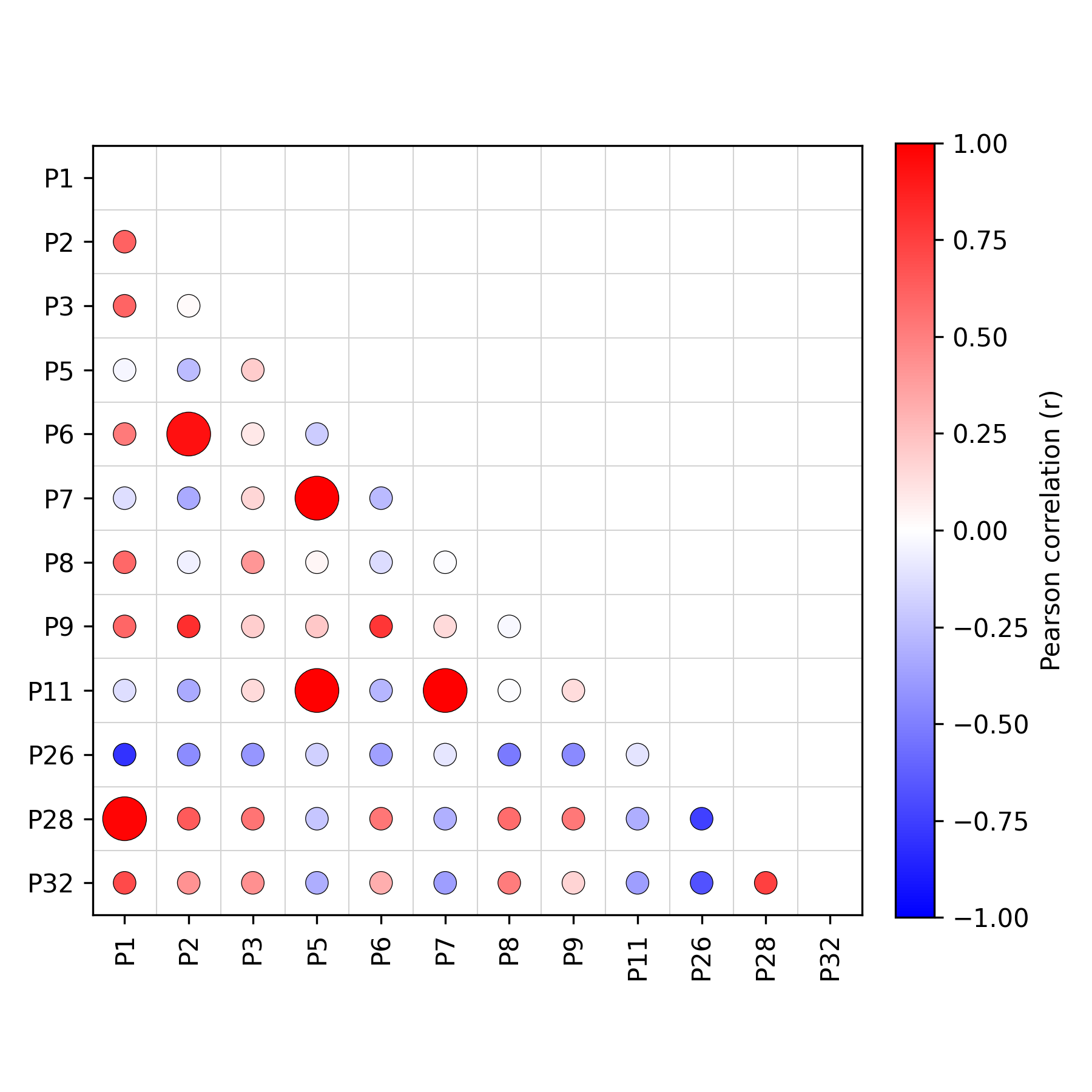

Supplement: Supplementary file 1 [file biology-15-00765-s001.zip › Supplementary/File S8/phytoplankton_2022_05.png]

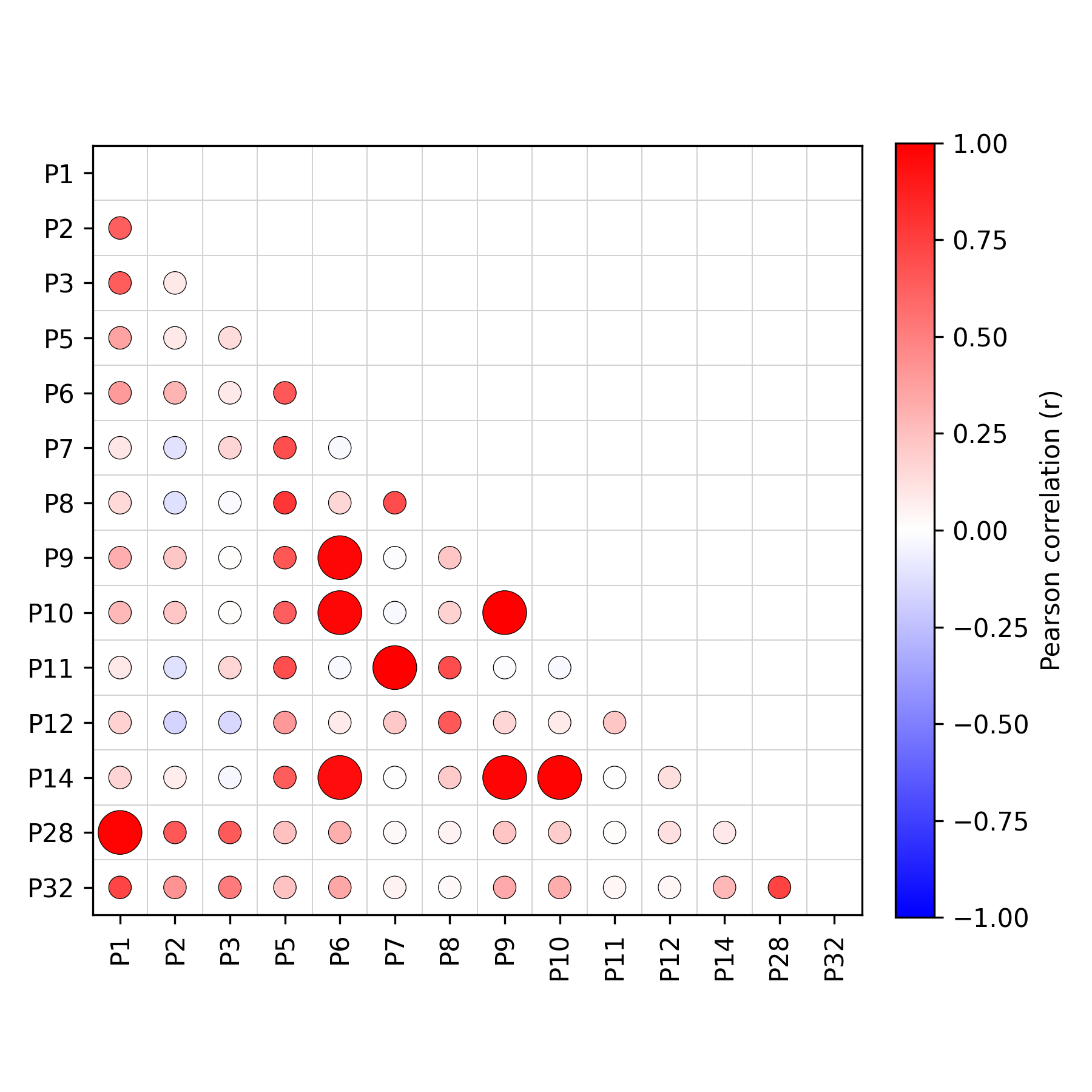

Supplement: Supplementary file 1 [file biology-15-00765-s001.zip › Supplementary/File S8/phytoplankton_2022_09.png]

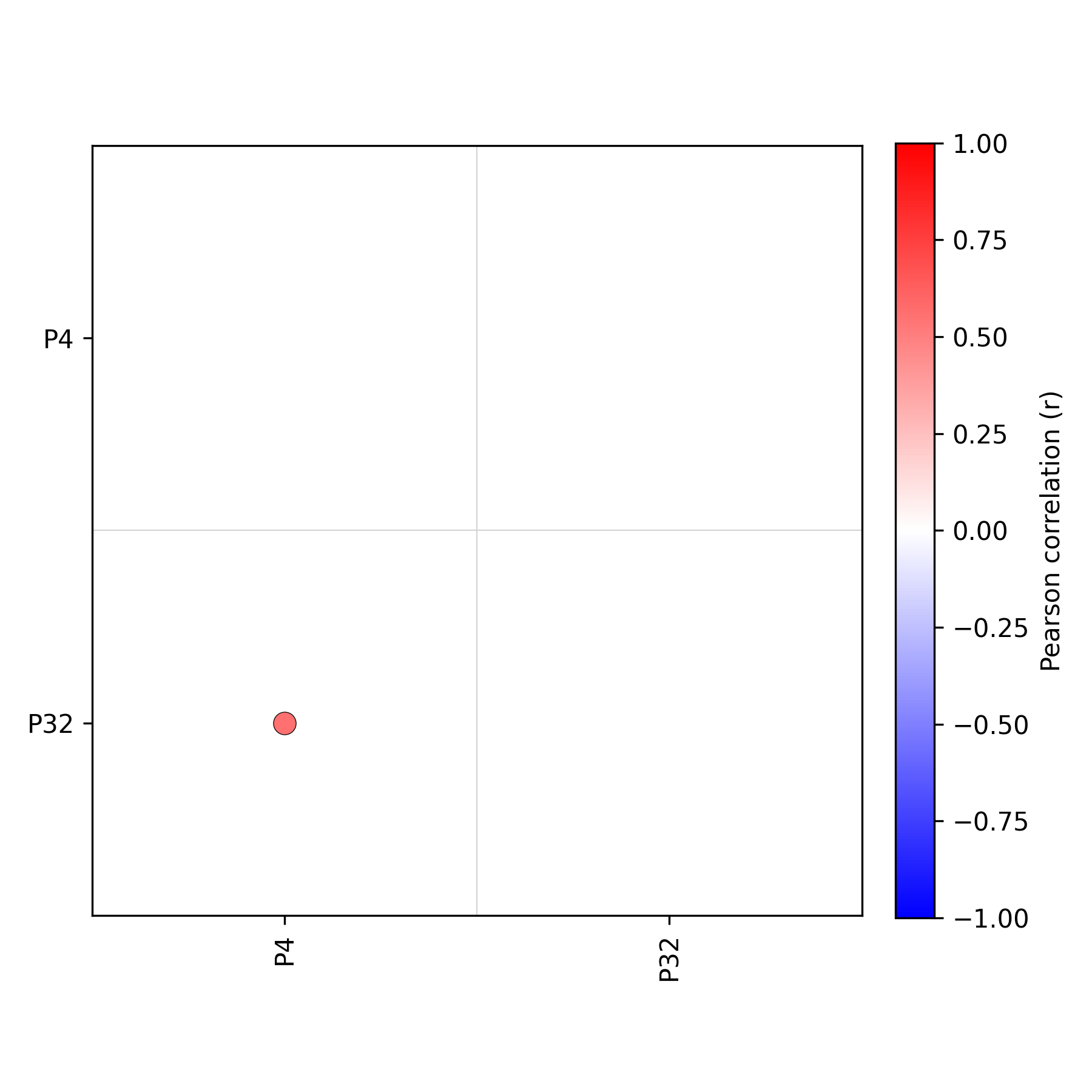

Supplement: Supplementary file 1 [file biology-15-00765-s001.zip › Supplementary/File S8/phytoplankton_2023_05.png]

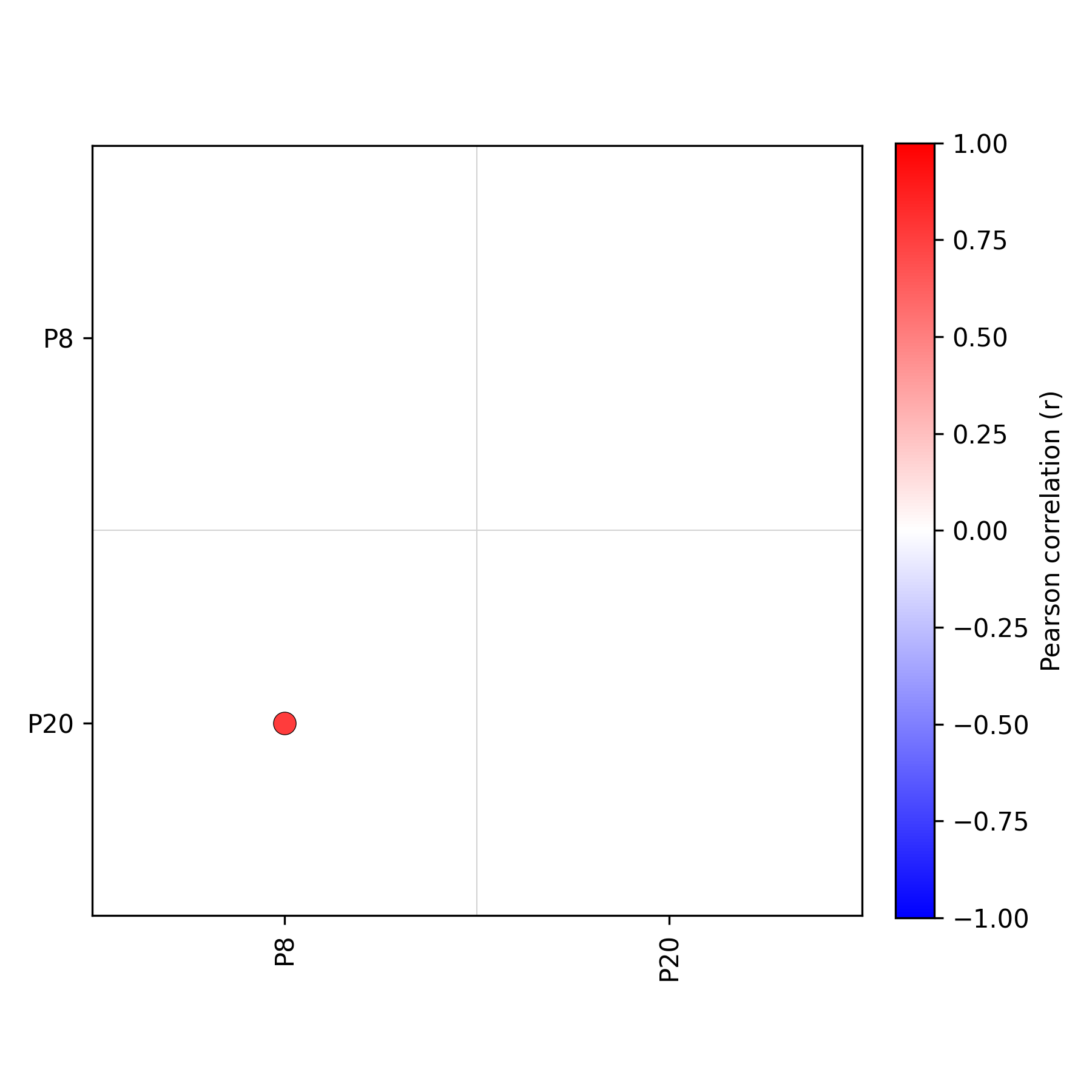

Supplement: Supplementary file 1 [file biology-15-00765-s001.zip › Supplementary/File S8/phytoplankton_2023_09.png]

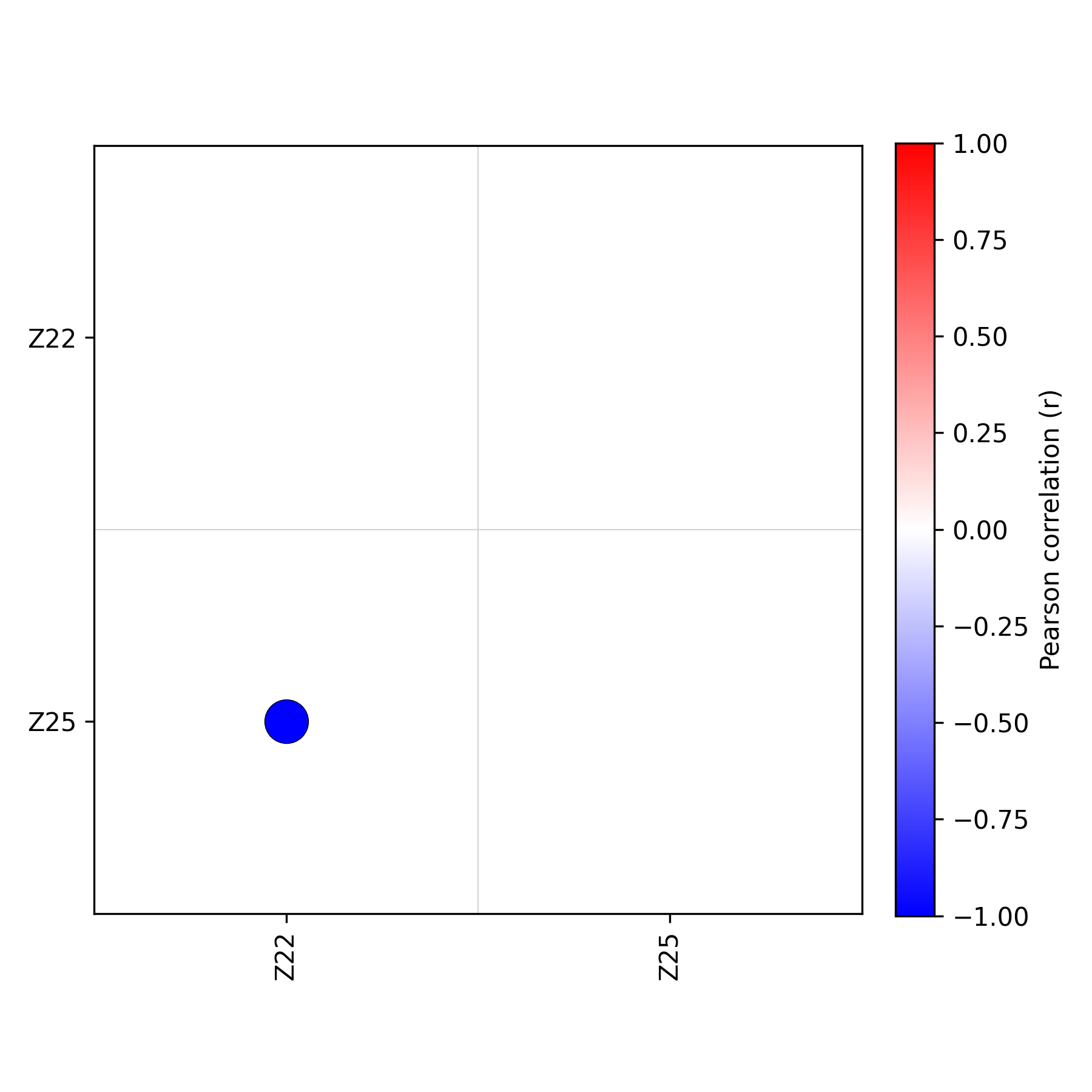

Supplement: Supplementary file 1 [file biology-15-00765-s001.zip › Supplementary/File S8/zooplankton_2021_05.png]

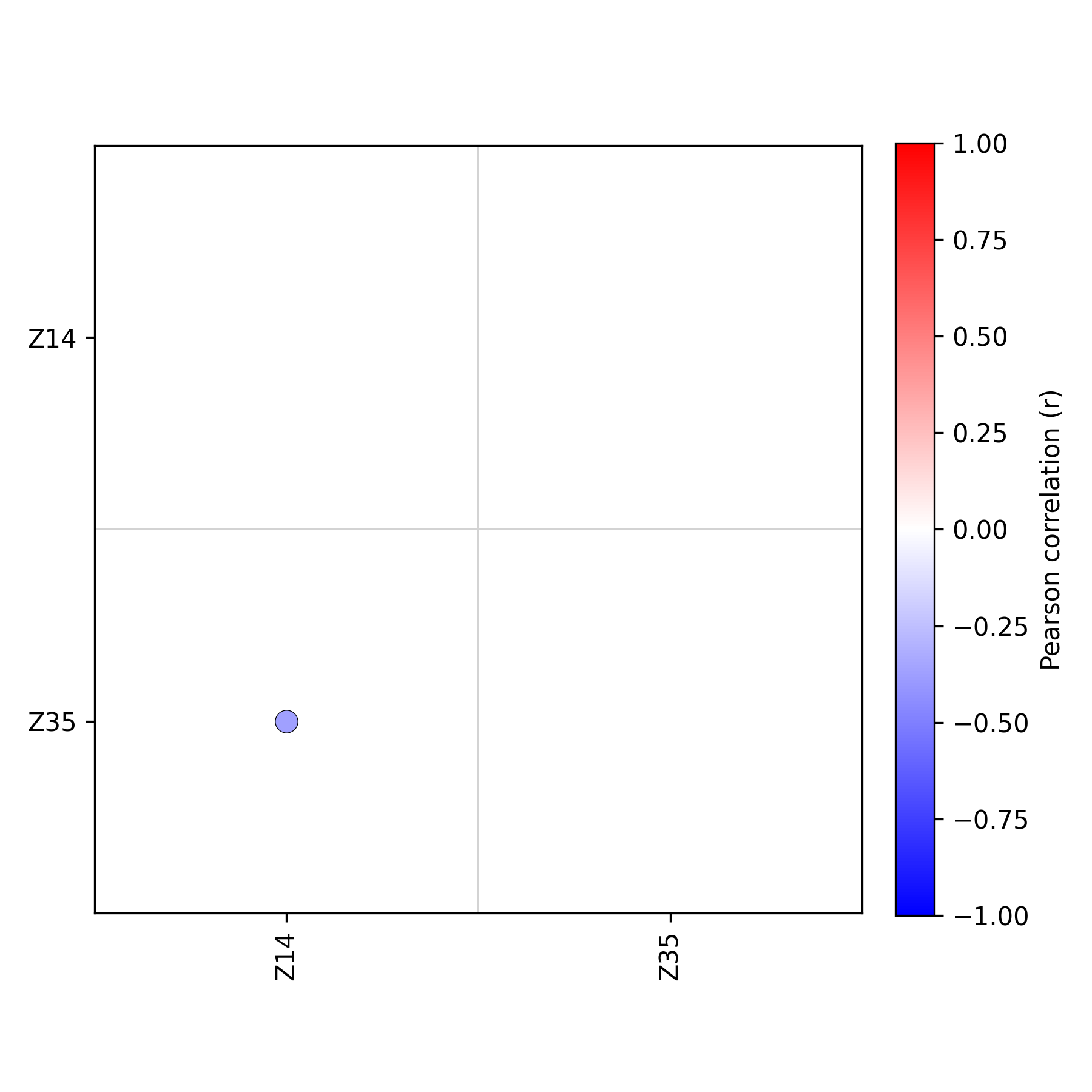

Supplement: Supplementary file 1 [file biology-15-00765-s001.zip › Supplementary/File S8/zooplankton_2021_09.png]

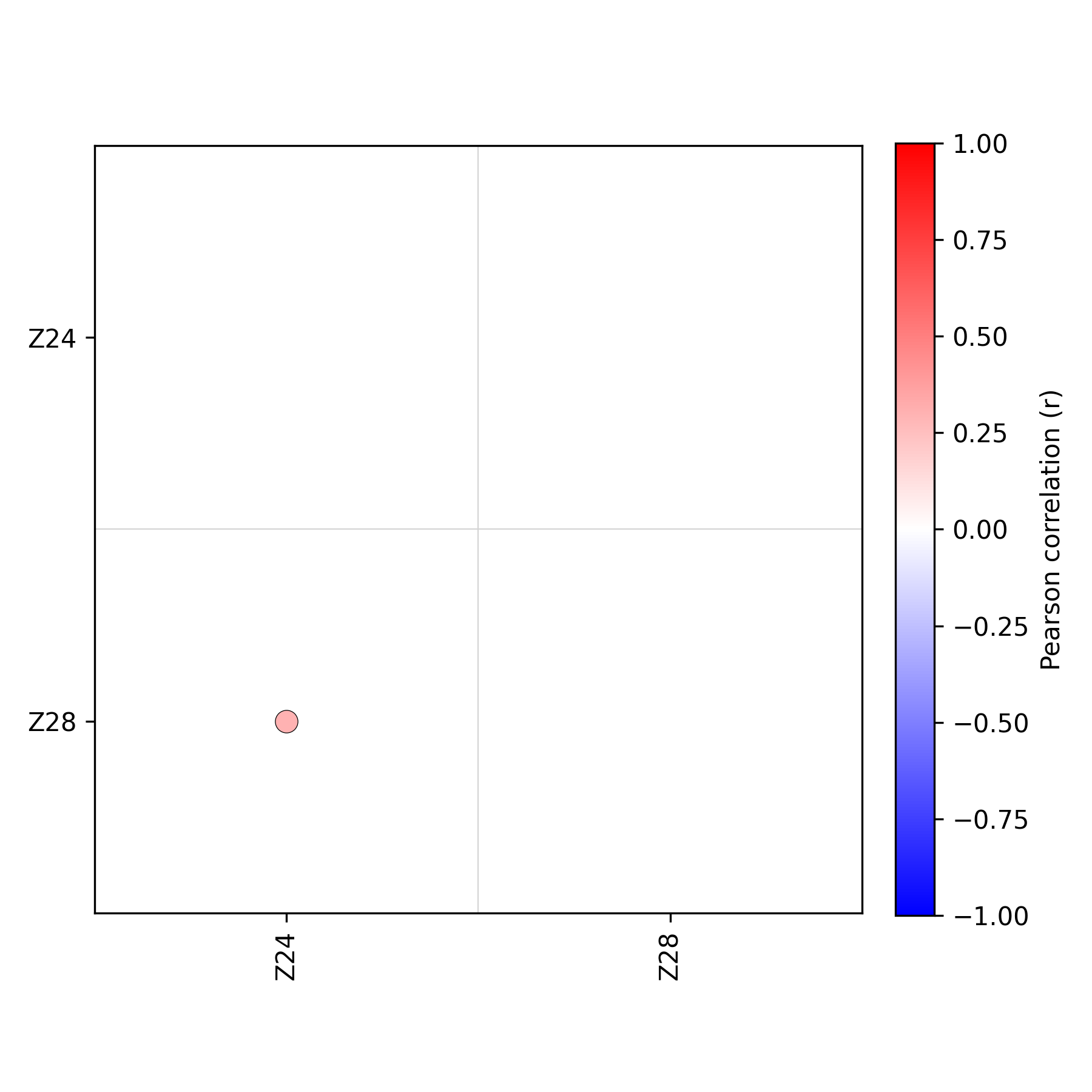

Supplement: Supplementary file 1 [file biology-15-00765-s001.zip › Supplementary/File S8/zooplankton_2022_05.png]

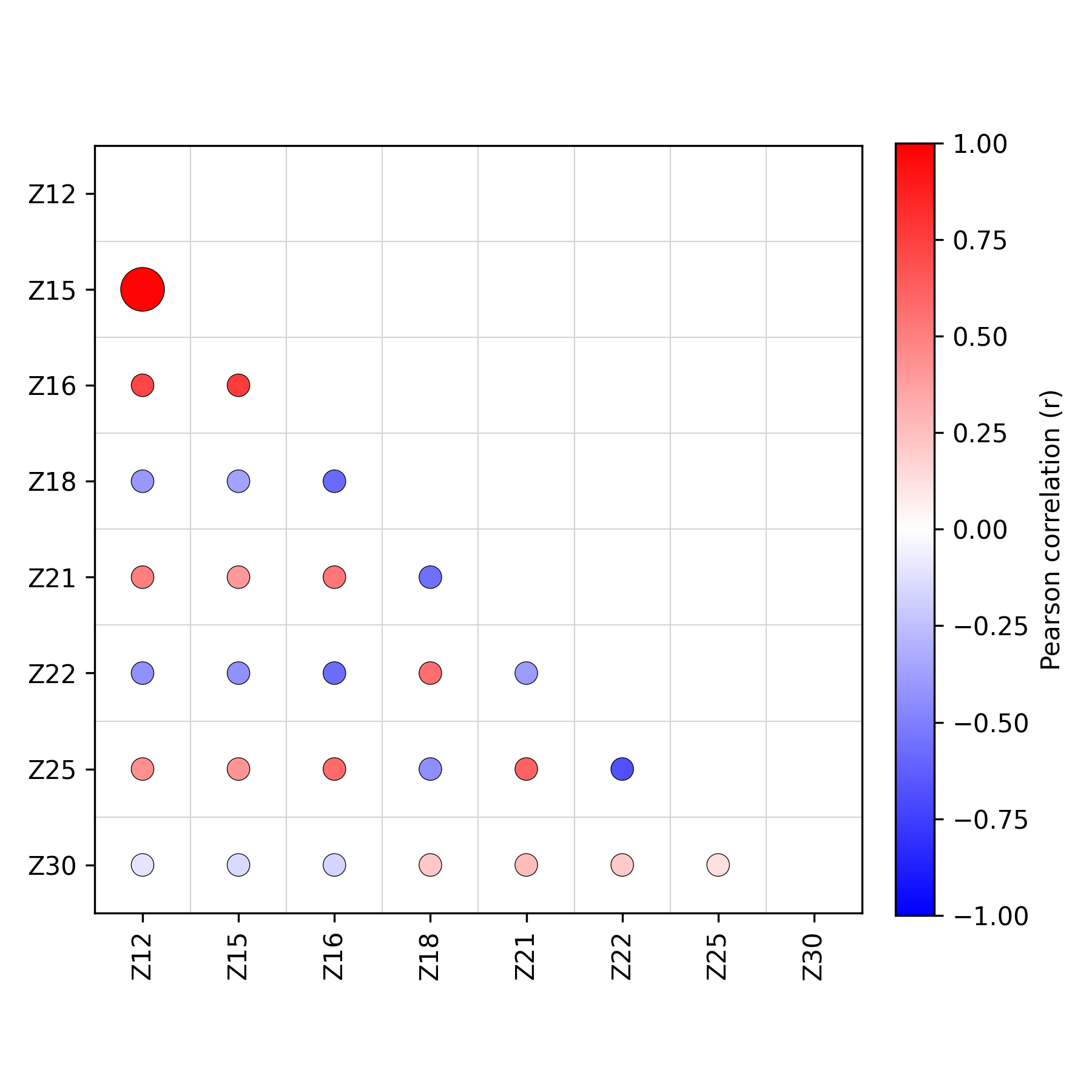

Supplement: Supplementary file 1 [file biology-15-00765-s001.zip › Supplementary/File S8/zooplankton_2022_09.png]

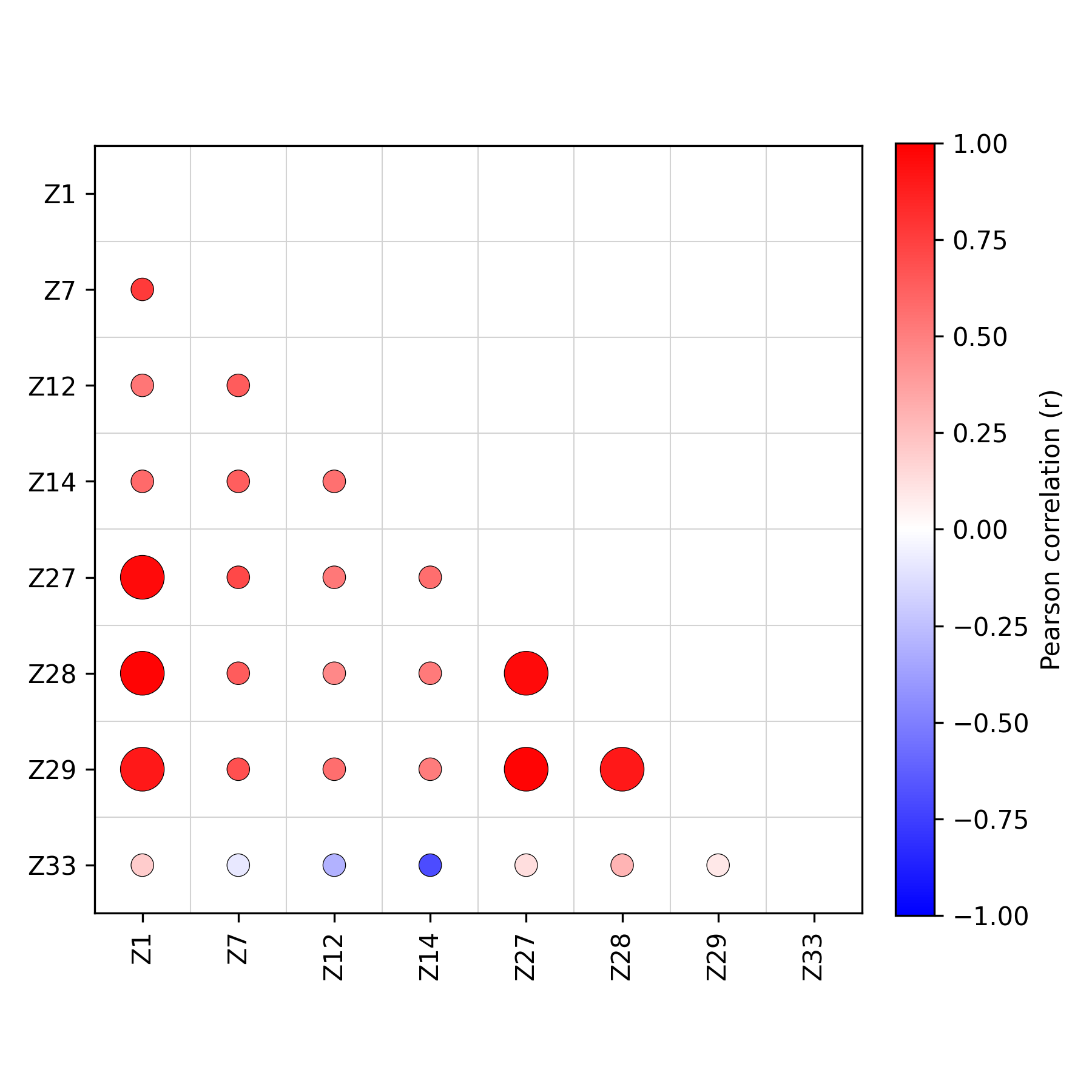

Supplement: Supplementary file 1 [file biology-15-00765-s001.zip › Supplementary/File S8/zooplankton_2023_05.png]

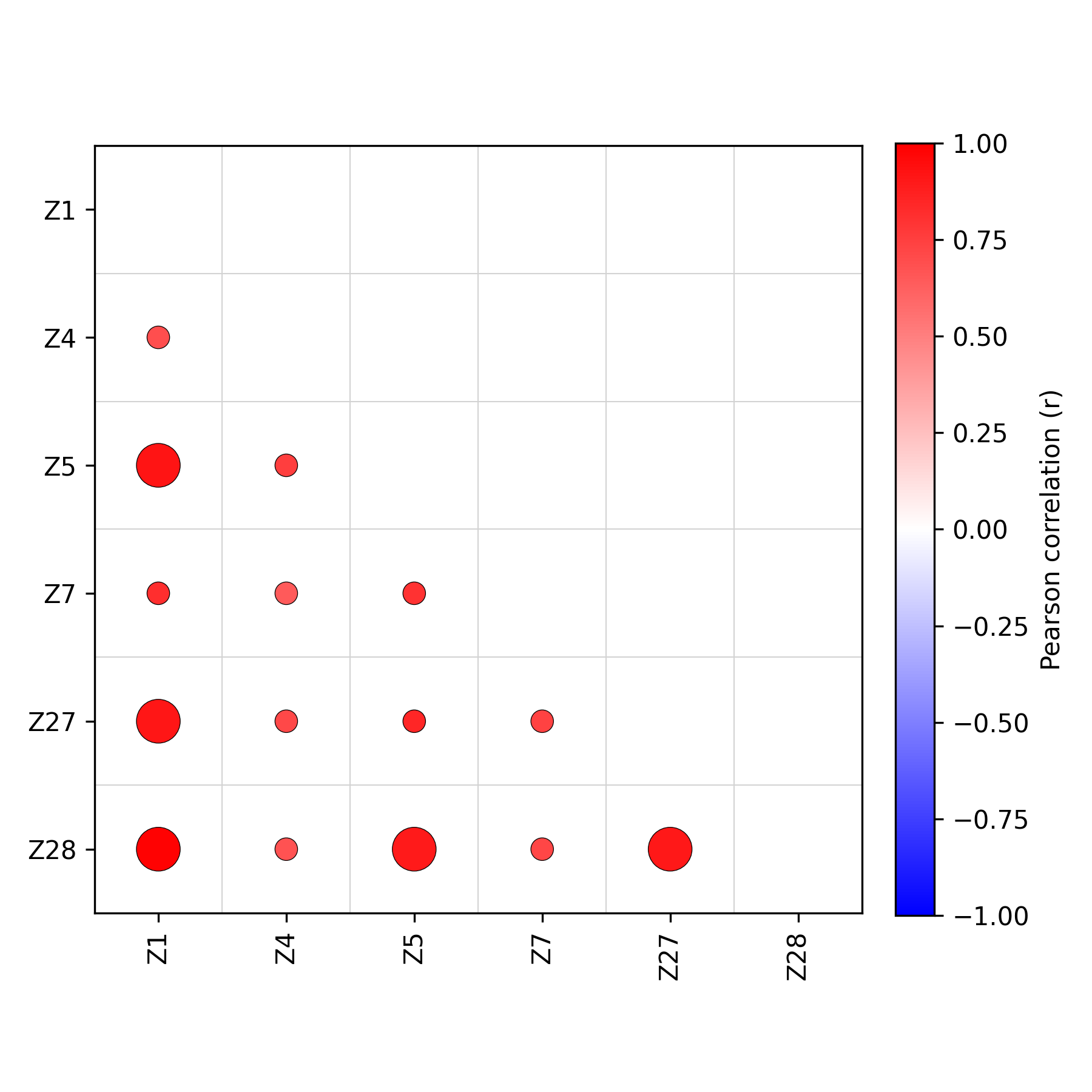

Supplement: Supplementary file 1 [file biology-15-00765-s001.zip › Supplementary/File S8/zooplankton_2023_09.png]
